# Prediction of the therapeutic efficacy of epirubicin combined with ifosfamide in patients with lung metastases from soft tissue sarcoma based on contrast-enhanced CT radiomics features

**DOI:** 10.1186/s12880-022-00859-6

**Published:** 2022-07-26

**Authors:** Lei Miao, Shu-Tao Ma, Xu Jiang, Huan-Huan Zhang, Yan-Mei Wang, Meng Li

**Affiliations:** 1 Department of Diagnostic Radiology, National Cancer Center/National Clinical Research Center for Cancer/Cancer Hospital, Chinese Academy of Medical Sciences and Peking Union Medical College, No. 17 Panjiayuan Nanli, Chaoyang District, Beijing, 100021 China; 2 Department of Medical Oncology, National Cancer Center/National Clinical Research Center for Cancer/Cancer Hospital, Chinese Academy of Medical Sciences and Peking Union Medical College, No. 17, Panjiayuan Nanli, Chaoyang District, Beijing, 100021 China; 3 GE Healthcare China, Pudong New Town, Shanghai, People’s Republic of China

**Keywords:** Radiomics, Soft tissue sarcoma, Lung metastases, Efficacy prediction

## Abstract

**Objective:**

To investigate the value of contrast-enhanced computed tomography (CECT) radiomics features in predicting the efficacy of epirubicin combined with ifosfamide in patients with pulmonary metastases from soft tissue sarcoma.

**Methods:**

A retrospective analysis of 51 patients with pulmonary metastases from soft tissue sarcoma who received the chemotherapy regimen of epirubicin combined with ifosfamide was performed, and efficacy was evaluated by Recist1.1. ROIs (1 or 2) were selected for each patient. Lung metastases were used as target lesions (86 target lesions total), and the patients were divided into a progression group (n = 29) and a non-progressive group (n = 57); the latter included a stable group (n = 34) and a partial response group (n = 23). Information on lung metastases was extracted from CECT images before chemotherapy, and all lesions were delineated by ITK-SNAP software manually or semiautomatically. The decision tree classifier had a better performance in all radiomics models. A receiver operating characteristic curve was plotted to evaluate the predictive performance of the radiomics model.

**Results:**

In total, 851 CECT radiomics features were extracted for each target lesion and finally reduced to 2 radiomics features, which were then used to construct a radiomics model. Areas under the curves of the model for predicting lesion progression were 0.917 and 0.856 in training and testing groups, respectively.

**Conclusion:**

The model established based on the radiomics features of CECT before treatment has certain predictive value for assessing the efficacy of chemotherapy for patients with soft tissue sarcoma lung metastases.

**Supplementary Information:**

The online version contains supplementary material available at 10.1186/s12880-022-00859-6.

## Key points

- The CECT-based radiomics signature may help to predict the therapeutic efficacy of epirubicin combined with ifosfamide in patients with soft tissue sarcoma lung metastases.
- Doxorubicin combined with ifosfamide is the standard first-line treatment for patients with advanced unresectable/metastatic STS.
- Several radiomics models were established, among which the model established by the decision tree classifier had a better effect.

## Introduction

Soft tissue sarcoma (STS) is a heterogeneous group of malignant tumours of mesenchymal origin, accounting for less than 1% of all adult tumours, and can be further divided into approximately 70 subtypes, each with different morphological features [1, 2]. STS can occur anywhere in the body, with the extremities (43%), trunk (10%), internal organs (19%), retroperitoneal area (15%), and head and neck (9%) being the most common primary sites. Among STS patients, 40 to 50% will develop metastases, the most common site of which is the lung, and patients with lung metastases have poor prognosis [3–6]. According to SEOM guidelines, chemotherapy is the standard systemic treatment for advanced unresectable/metastatic STS [7]. Anthracycline-based (such as doxorubicin, epirubicin, pirarubicin) chemotherapy is currently the most effective treatment for STS. At present, doxorubicin combined with ifosfamide is the standard first-line treatment for patients with advanced unresectable/metastatic STS [8]. Epirubicin, an isomer of doxorubicin, is a member of a new class of anthracycline antibiotics. Compared with doxorubicin, epirubicin has the same or slightly higher efficacy but has less cardiac toxicity. Its mechanism of action is direct intercalation between nucleobase pairs of DNA, interfering with the transcription process and preventing the formation of mRNA and thereby inhibiting synthesis of both DNA and RNA [9].

Response Evaluation Criteria in Solid Tumours version 1.1 (Recist1.1) is currently the most widely used clinical response evaluation standard for solid tumours, using complete response (CR), partial remission (PR), stable disease (SD), and progressive disease (PD) to judge disease response to treatment [10]. In a previous study, approximately 77% (2% CR, 25% PR, and 50% SD) of patients benefited from the chemotherapy cycle of doxorubicin and ifosfamide, but approximately 30% experienced disease progression or even death, with the disease progressing rapidly (median overall survival 12.8 months) [11]. In general, when lung metastatic lesions enlarge and reach the standard of PD during the chemotherapy cycle, the clinic will switch to a new regimen or other treatment. If we could predict disease progression in advance, we could choose or switch to other treatment options (such as second-line chemotherapy, localized radiotherapy, localized surgery) in a timely manner such that patients may have the greatest clinical benefit [7].

CT is the most important imaging modality for evaluating the efficacy of lung metastases in STS. However, conventional CT can only assess efficacy based on size and Recist1.1 but cannot predict efficacy. Radiomics has become a hot research topic in recent years: it can extract massive data from medical images with high throughput and analyse high-level, quantitative image features to deeply reflect the spatial heterogeneity of tumour tissue [12]. Contrast-enhanced CT (CECT)-based radiomics has been widely used in the study of various tumour efficacy predictions [13–15]. However, there is no previous study on the prediction of chemotherapy efficacy in metastases. The purpose of this study was to construct CECT-based radiomics models to predict the efficacy of epirubicin combined with ifosfamide in patients with lung metastases from STS and to help clinicians choose better treatment options.

## Materials and methods

### General information

A retrospective analysis of 51 patients with pulmonary metastases from STS from March 2014 to July 2021 was performed. All patients received epirubicin combined with ifosfamide chemotherapy (intravenous epirubicin 60 mg/m^2^, ifosfamide 3 ~ 5 g/m^2^); the treatment course was determined according to the specific treatment response. The inclusion criteria were as follows: ① diagnosed with pulmonary metastases from STS; ② received the established chemotherapy regimen of epirubicin combined with ifosfamide; ③ underwent an enhanced chest CT scan within three weeks before treatment; and ④ complete clinical, imaging and pathological data available. The exclusion criteria were as follows: ① not receiving a complete set of chemotherapy regimens; ② incomplete imaging data or enhanced CT scans; ③ other malignant tumours present; and ④ unclear pathological diagnosis of metastases.

### Efficacy evaluation and analysis methods

According to Recist1.1, the clinical efficacy of the patient's whole body was evaluated. PR was defined as at least a 30% reduction in the sum of the diameters of the target lesions compared to baseline. PD was defined as at least a 20% increase in the sum of the diameters of the target lesions over the entire study period, and the absolute value of the sum of the diameters of the target lesions must have also increased by at least 5 mm. In addition, the appearance of one or more new lesions was also considered to be PD. SD was defined as neither a decrease to PR nor an increase to PD during the study, as based on the sum of the target lesion diameters according to the minimum sum of the target lesion diameters.

**Fig. 1:**
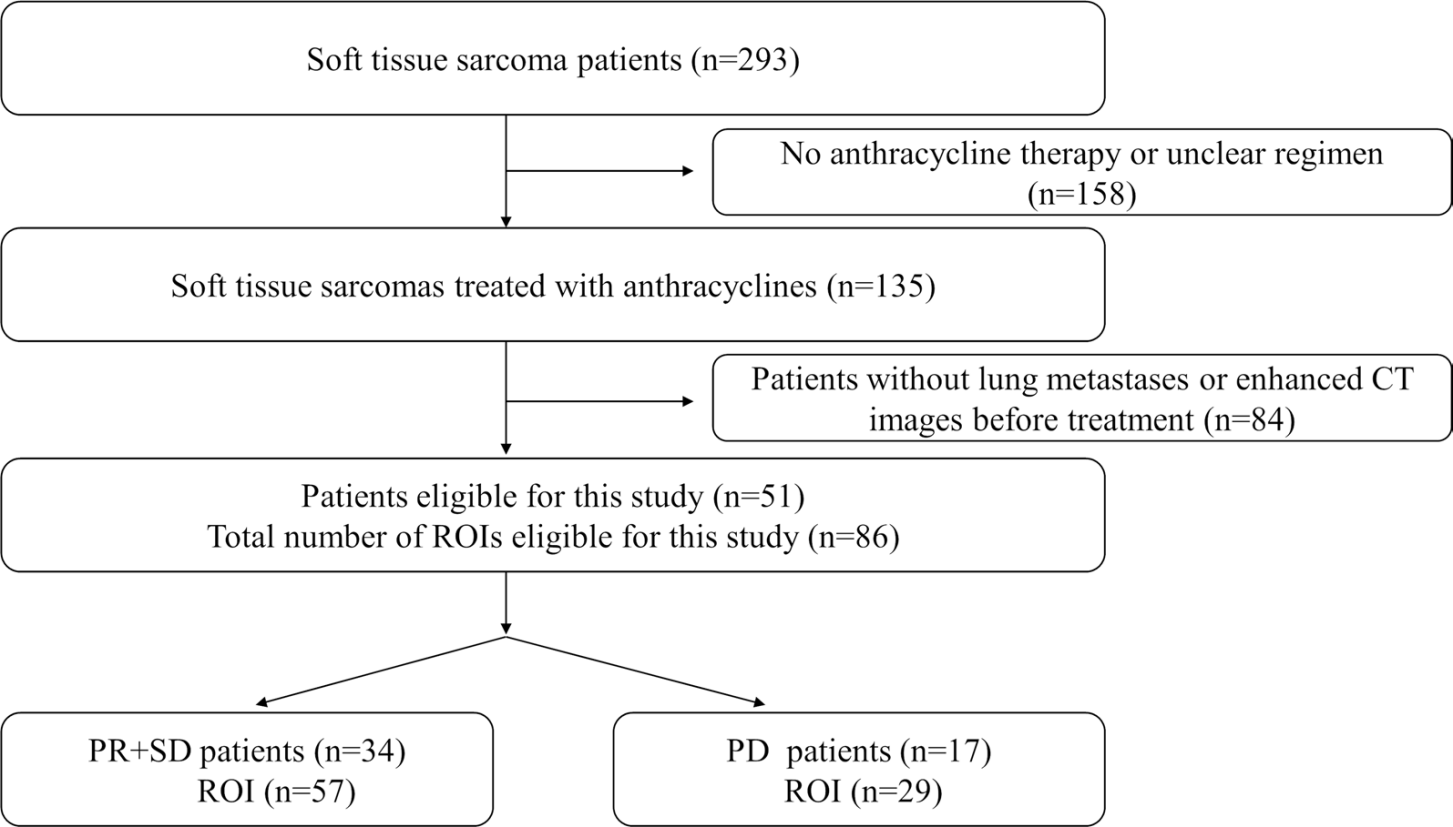
Patient Inclusion Process

The most recent enhanced CT scan before chemotherapy was selected as the imaging data of our study. For patients (n = 16) with a single lung metastasis, this metastasis was selected as the target lesion. For patients with multiple lung metastases (n = 35), 2 lung metastases were selected as the target lesions. There was a total of 86 target lesions, and the patients were divided into a progression group (n = 29) and a non-progression group (n = 57), with the non-progression group including patients with SD (n = 34) and patients with PR (n = 23). The inclusion and exclusion criteria and a flow chart of patient registration are shown in Fig. 1.

### Instruments and methods

The following CT scanning instruments were used: Optima CT660, BrightSpeed CT, Revolution CT, Discovery CT750 (GE Medical System, Milwauke, WI) and Toshiba Aquilion 64 -slice spiral CT. Before scanning, the patient performed breathing training. The patient was placed in a supine position, with the head or feet advanced, and a breath-hold scan was performed after deep inhalation. All patients underwent an enhanced CT scan; the tube voltage was 120 kVp, the tube current was 200–350 mAs, the generated images were 5 mm thick, and some images were reconstructed with 1.25 mm layer thickness. The contrast agent used for enhanced scanning was iopromide injection (iodine concentration of 300 mg/ml) at a dose of 80 to 90 ml and a flow rate of 2.5 to 3.0 ml/s. Standard algorithms and high-resolution algorithms were used for image reconstruction and parallel multiplane reconstruction. The lung window (window width 1500 HU, window level -550 HU) and mediastinal window (window width 350 HU, window level 50 HU) were selected for image observation.

### Image acquisition and segmentation

**Fig. 2:**
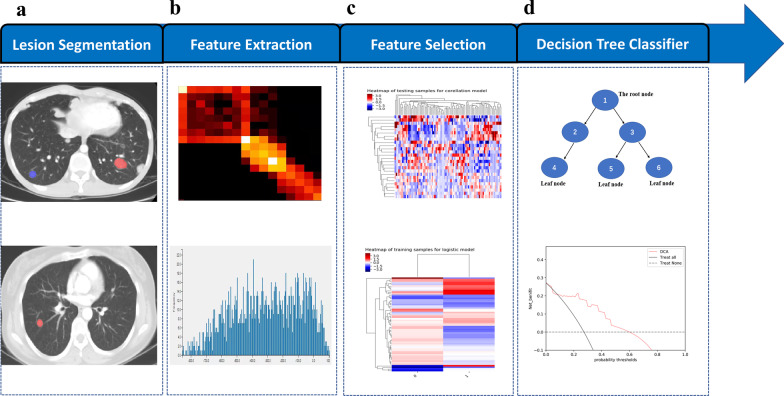
Workflow of radiomics feature analysis. DCA, decision curve analysis

DICOM images were exported from the picture archiving and communications system (PACS), and the images were preprocessed by resampling 1 × 1 × 1 before region of interest (ROI) delineation to eliminate differences between images with different slice thicknesses. Each CECT scan of each patient was normalized with Z-scores in order to get a standard normal distribution of image intensities. ITK-SANP software [16] was used for ROI segmentation. The ROI was manually or semiautomatically delineated layer by layer on the lung window image until all lung metastases were included. Lesion segmentation was completed by an imaging graduate student specializing in thoracic diagnosis, and the delineated areas of different target lesions were highlighted in the figure with specific colours. The segmentation results were then confirmed by a chief physician specializing in thoracic imaging diagnosis. In cases of disagreement, consensus was reached through consultation. Attempts were made to avoid marking beyond the edge of the lesion. Figure 2 shows the workflow of radiomics feature analysis.

### Feature selection and model building

AK software (Artificial Intelligence Kit V3.0.0. R, GE) was used to extract the radiomics features of the target lesions, whereby five kinds of features (including grey level co-occurrence matrix, grey level size zone, histogram, morphological features and run length matrix) are extracted and the image data in the segmented region are calculated. The feature calculation formula embedded in AK software follows the image biomarker standardization initiative (IBSI) standard. The calculation formula corresponding to each feature can be seen on the website [17]. The dimensionality reduction model of imaging radiomics features was built on IPMS software (Institute of Precision Medicine Statistics v2.5.2. R, GE). The patients were randomly divided into a training group (n = 60) and a test group (n = 26) according to a ratio of 7:3, and models were constructed with the data of the training group and evaluated with the data of the test group. The feature parameters were first standardized (standardization). Dimensionality reduction was performed by means of variance, multivariate logistic regression and the recursive feature elimination (RFE) strategy. The random forest classifier, logistic regression, support vector machine, naïve Bayesian classification, decision tree classifier, and K-nearest neighbour methods were used to establish radiomics models. The radiomics analysis process is provided in the Additional file 1.

### Statistical analysis

SPSS 26.0 statistical analysis software was used. An independent samples t test was used to compare age and the χ^2 ^test to compare sex and the curative effect. P < 0.05 was considered statistically significant. Model-predicted progression probabilities were compared using the evaluation results of Recist1.1. A receiver operating characteristic (ROC) curve was drawn, and the area under the curve (AUC) was calculated. The point corresponding to the maximum value of the Youden index (i.e., the point where the sum of sensitivity and specificity is the largest) was used as a cut-off to distinguish progression from non-progression, and the positive predictive value and negative predictive value were calculated. The benefit of the model was evaluated by decision curve analysis (DCA).

## Results

### General features

A total of 51 patients were included, 24 males and 27 females, ranging in age from 16 to 82 years, with an average age of 45.4 (± 14.1) years. There were no significant differences in age, sex or efficacy between the training group and the test group (P > 0.05). In this study, a given patient may have 1 or 2 ROIs, and the ROI was used as the grouping; thus, statistical descriptive indicators, including age and sex, will be different from the description of the general data. The above situation is shown in Table 1.

**Table 1:** General Information of training and testing groups.

	Age	Sex (Male/Female)	PR + SD (n = 57)	PD (n = 29)
Training (n = 60)	46.27 ± 12.73	26/34	40	20
Testing (n = 26)	44.15 ± 16.16	15/11	17	9
t/F value	− 0.65	1.49	0.23	
p value	0.517	0.23	0.91	

### Radiomics features

The decision tree classifier, which had the best effect, was selected to establish a radiomics model, and the test group was used to verify the feature performance and evaluate the data for the training group.

For each ROI, 851 features were extracted, including 18 first-order features, 14 3D shape features, 75 s-order features (GLRLM, GLSZM, GLCM, NGTDM, and GLDM), and 744 wavelet transform features (based on the transformation of previous features). Through dimensionality reduction, the final number of radiomics features was 2 (wavelet-HHH_First Order Mean and wavelet-LHL_GLRLM Long Run Low Grey Level Emphasis).

### Model performance

**Fig. 3:**
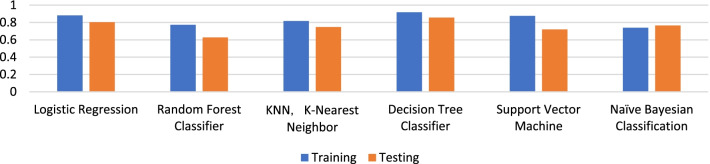
AUC Comparison of Multiple Models. The abscissa describes the model’s name. The ordinate represents the AUC value of the model. Blue represents the training group. Orange represents the testing group

**Fig. 4:**
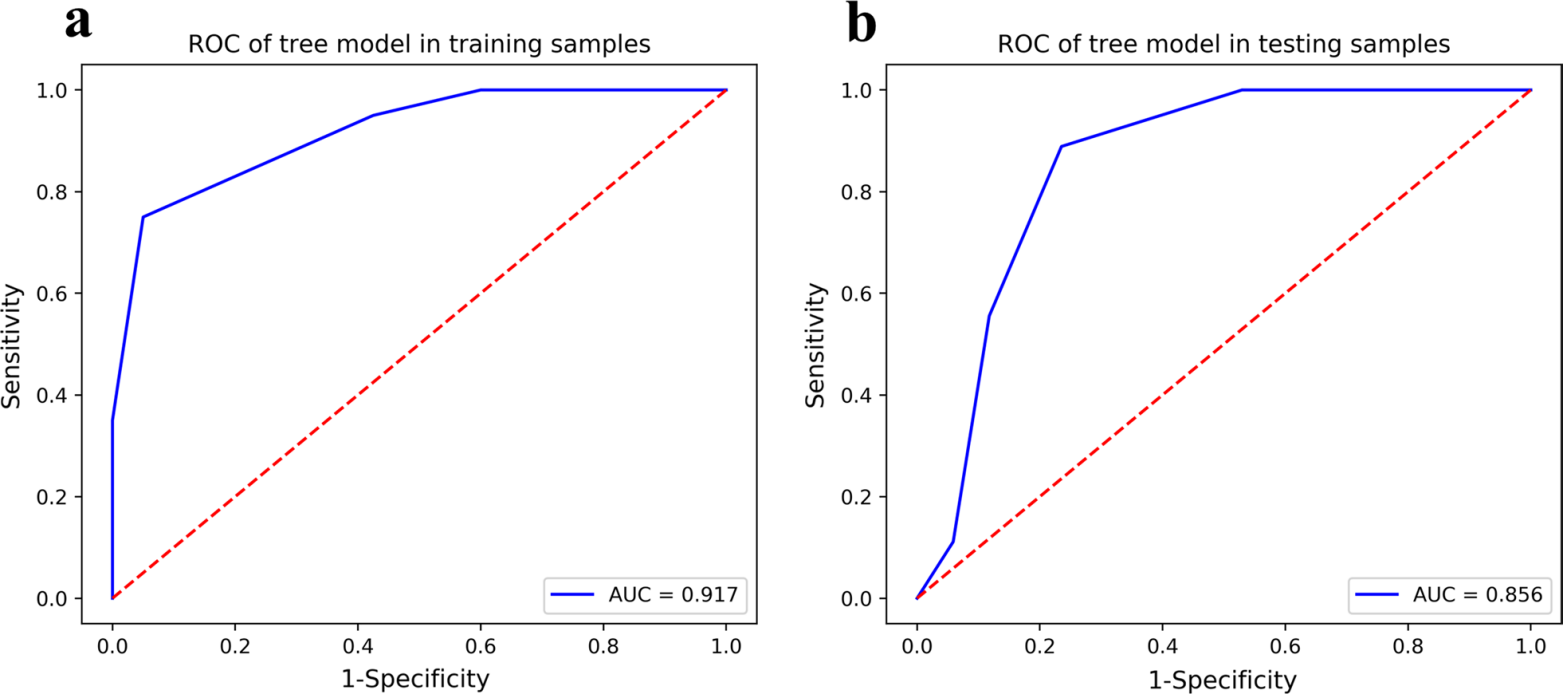
ROC Curve of the Training Group and Test Group. **a** ROC Curve of the Training Group. **b** ROC Curve of the Test Group

**Fig. 5:**
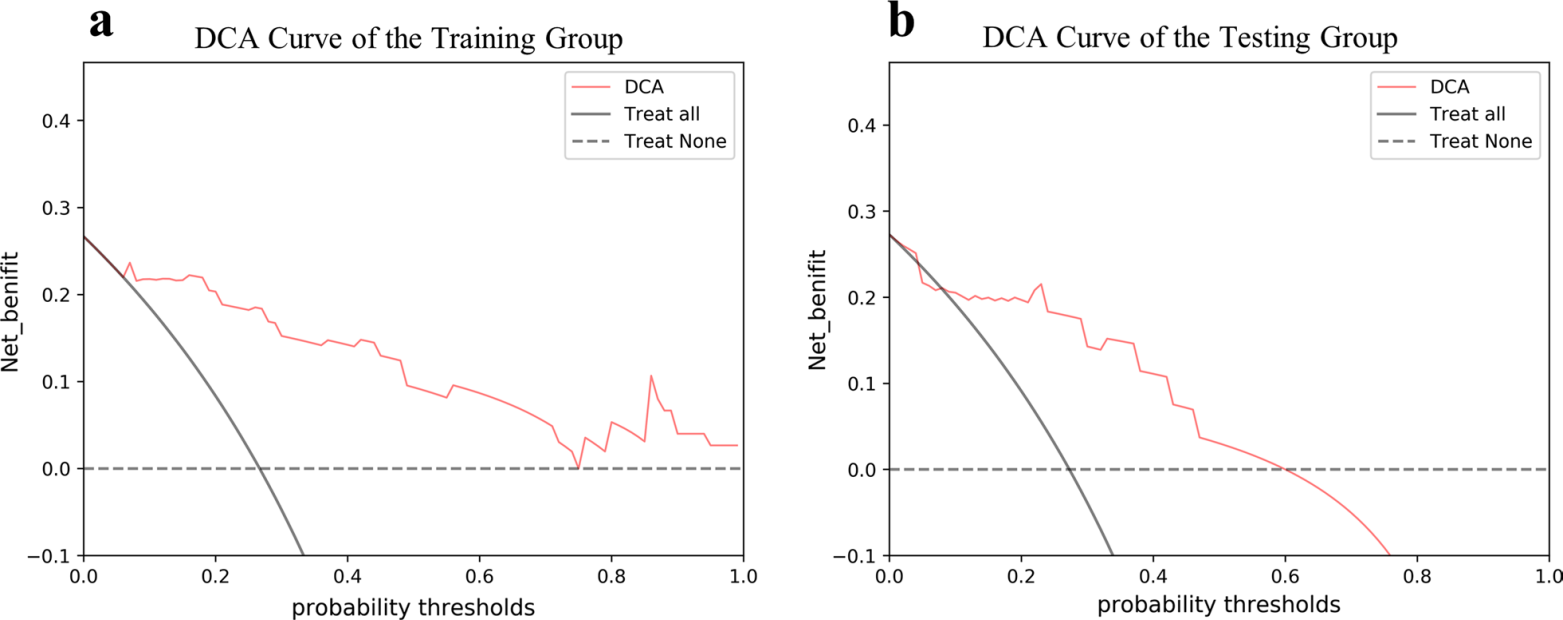
DCA Curve of the Training Group and Test Group. **a** DCA Curve of the Training Group. **b** DCA Curve of the Test Group

Comparison of the AUC values of various models in the training and test groups are illustrated in Fig. 3. According to ROC curves (Fig. 4), the AUC of the decision tree classifier model for predicting lesion progression in the training group was 0.917 (95% CI 0.858, 0.969) for the training group and 0.856 (95% CI 0.726, 0.967) for the test group. The sensitivity and specificity balance point, that is, the probability corresponding to the maximum value of the sum of the two, was used as the threshold to determine lesion progression. The confusion matrix under this balance point describes the numbers of true positive, false positive, true negative and false negative cases. The sensitivity, specificity, accuracy, positive predictive value and negative predictive value of predicting lesion progression were 75.0%, 95.0%, 88.3%, 88.2% and 88.4% in the training group and 55.6%, 88.2%, 76.9%, 71.4% and 78.9% in the test group, respectively. The diagnostic performance of the decision tree classifier in the training and test groups is given in Table 2. The DCA curve showed that the net benefits of the radiomics model in both the training group and the test group were better than those of the treat-none model and the treat-all model over a wide range of risk thresholds between 0.1 and 1.0 (Fig. 5).

**Table 2:** Diagnostic performance of the decision tree classifier in the training and testing groups

	Training group	Testing group
AUC	0.917	0.856
Sensitivity (%)	75	55.6
Specificity (%)	95	88.2
Accuracy (%)	88.3	76.9
Positive predictive value (%)	88.2	71.4
Negative predictive value (%)	88.4	78.9
95% CI	0.858 ~ 0.969	0.726 ~ 0.967

## Discussion

This study aimed to establish a variety of radiomics models to predict the treatment effect of chemotherapy for STS lung metastases. A total of 6 kinds of radiomics models were established, of which the model with the best performance in the training and test groups was the decision tree classifier. All other indicators showed good predictive value (AUC of training group and testing group: 0.917, 0.856). Our results demonstrate that CECT-based radiomics features can predict the efficacy of epirubicin combined with ifosfamide for treatment of pulmonary metastases from STS. In the clinic, epirubicin combined with ifosfamide is the first-line chemotherapy regimen for STS with lung metastases; however, there are still some patients who do not benefit from this approach and may rather benefit from other treatments and there is no detailed and broad consensus on the selection of this first-line chemotherapy regimen. The radiomics model established in this study can predict whether metastatic lesions are suitable for this treatment (epirubicin combined with ifosfamide) based on radiomics features of metastatic lesions, which adds a new judgement index for clinical regimen selection. If the predictive model suggests that there is still a high probability of disease progression, other treatment options can be chosen in a timely manner.

Radiomics has been widely used for evaluating the efficacy of neoadjuvant chemoradiotherapy for STS. Crombe first proposed the application of delta -radiomics to predict the efficacy of neoadjuvant therapy for STS, analysing its value based on T_2_-weighted sequences in predicting pCR in 65 STS inpatients before and after neoadjuvant therapy [18]. In a recent study, Peeken et al. [19] retrospectively studied 156 patients treated with neoadjuvant chemoradiotherapy and established a delta-radiomics model to predict its efficacy for STS, which confirmed the advantages of radiomics in evaluating treatment efficacy in STS. Nevertheless, radiomics has mostly been used to evaluate efficacy in primary lesions of specific diseases, and the evaluation of radiomics for the lung has largely been based on lung cancer [20]. Indeed, as there are no previous radiomics studies for predicting response with intrapulmonary metastases, the present study is both pioneering and advanced. Additionally, this study helps to fill the gap of efficacy prediction in advanced sarcoma.

Recist1.1 criteria were selected as the control criteria in this study because they are currently the most widely used clinical efficacy evaluation criteria for solid tumours [21]. Although Recist1.1 criteria are controversial in the evaluation of neoadjuvant therapy for STS, some STSs that respond biologically to radiotherapy/ chemotherapy may not shrink due to tumour enlargement due to necrosis, intratumoural haemorrhage, and/or cystic degeneration [22]; as the present study involved lung metastases, the above situation does not apply. In a previous radiomics study of multiple lesions, a single lesion in a given patient was usually selected for analysis [23]. However, in this study, the delineation of ROIs differed because patients with STS may have a single lung metastasis or multiple lung metastases. Therefore, the requirements of Recist1.1 were used in ROI delineation, and a target organ can be delineated two times at most. For each target lesion, single or multiple lesions can be selected for delineation, which not only increases the sample size of the ROI but also makes the research more in line with clinical reality.

The two radiomics features ultimately selected in this study were wavelet-HHH_First Order Mean and wavelet-LHL_ Gray Level Run Length Matrix (GLRLM) Long Run Low Grey Level Emphasis (LRLGLRE), which are both radiomics features obtained from wavelet transform. The first-order mean is expressed as the average value of the signal intensity of all pixels in the ROI, which can reflect the regularity of the image texture. GLRLM is defined as the length of the number of pixels, i.e., consecutive pixels with the same greyscale value, where LRLGLRE means measuring the joint distribution of long-run lengths with lower greyscale values, short-run dominance versus long-run dominance. The advantage reflects the smoothness and roughness of the image. Overall, the greater the advantage of the short run is, the smoother the texture of the image is; the greater the advantage of the long run is, the rougher the texture of the image is [24]. These radiomics features may more deeply reflect the heterogeneity of tumours and hence reflect sensitivity to chemotherapy regimens to predict efficacy [12].

There are certain limitations in this study. First, this was a single-centre, preliminary study with a relatively small sample size. Second, ROI segmentation was performed by manual or semiautomatic delineation, with potential errors that may cause a certain deviation. Third, some of the clinical data were incomplete because the patient was treated in various ways at other hospitals before being treated at our hospital, and such previous clinical data could not be carefully incorporated. In the future, we will conduct a prospective, multicentre, large-sample imaging study and include a complete and refined analysis of clinical factors.

In conclusion, CECT-based radiomics has certain value for noninvasively predicting the therapeutic efficacy of epirubicin combined with ifosfamide in patients with STS lung metastases. Our preliminary findings suggest that CECT radiomics has the potential to be used as a noninvasive biomarker to predict the efficacy of epirubicin combined with ifosfamide in the treatment of pulmonary metastases from STS. This study can help guide individualized treatment strategies for pulmonary metastases from STS.

## Supplementary Information

**Additional file 1.** Supplementary Material.

## Data Availability

The datasets used and/or analysed during the current study are available from the corresponding author on reasonable request.

## Abbreviations

Recist: Response evaluation criteria in solid tumours
RFE: Recursive feature elimination
ROC: Receiver operating characteristic
AUC: Area under the curve
STS: Soft tissue sarcoma
CECT: Contrast-enhanced computed tomography
CR: Complete response
PR: Partial remission
PD: Progressive disease
SD: Stable disease
PACS: Picture archiving and communications system
DCA: Decision curve analysis
LRLGLRE: Long run low grey level emphasis
GLRLM: Gray level run length matrix
