# Supplementary material for: Prediction of the therapeutic efficacy of epirubicin combined with ifosfamide in patients with lung metastases from soft tissue sarcoma based on contrast-enhanced CT radiomics features
Source: BMC Med Imaging. 2022 Jul 26;22:131. doi: 10.1186/s12880-022-00859-6 (PMC9316811; doi:10.1186/s12880-022-00859-6)

**Supplementary Material**

Catalog:

Page2-24: Radiomics analysis process of random forest classifier.

Page25-52: Radiomics analysis process of logistic regression.

Page53-76: Radiomics analysis process of support vector machine.

Page77-100: Radiomics analysis process of naïve Bayesian classification.

Page101-123: Radiomics analysis process of decision tree classifier.

Page124-147: Radiomics analysis process of K-nearest neighbor.

**Radiomics analysis process of random forest classifier.**

Summary Report

# The summary report recognized the best model from all models your training, and summarized its training process.

As the result shown, the best model was Forest, its training process including: ['select a path', 'set a seed', 'seperate a data', 'input data', 'standardize data', 'select feature', 'select feature', 'select feature', 'machine_learning']. Detailed information is shown below:

# 1. Data: C:/Users/212768837/Desktop/zhuanyi_86ROI/new/result2.csv

# 2. Random seed: 46

# 3. Seperative rate: 0.7

Seperated report:

|  | Sum | Pos | Neg |
| --- | --- | --- | --- |
| data | 86 | 29 | 57 |
| train | 60 | 20 | 40 |
| test | 26 | 9 | 17 |

# 4. Input data

The method for filling the missing data: Median

The method for dealing with outliers: Median

# 5. The method for standardizing the data: Standardization

# 6. The method for selecting features: Variance

parameters setted: {'threshold': 1.0}
num of remained features: 426
remained features:
[['original_shape_Elongation']
 ['original_shape_Flatness']
 ['original_shape_LeastAxisLength']
 ['original_shape_Maximum2DDiameterColumn']
 ['original_shape_Maximum2DDiameterRow']
 ['original_shape_Maximum2DDiameterSlice']
 ['original_shape_MeshVolume']
 ['original_shape_MinorAxisLength']
 ['original_shape_VoxelVolume']
 ['original_firstorder_10Percentile']
 ['original_firstorder_InterquartileRange']
 ['original_firstorder_Kurtosis']
 ['original_firstorder_MeanAbsoluteDeviation']
 ['original_firstorder_Median']
 ['original_firstorder_Minimum']
 ['original_firstorder_Range']
 ['original_firstorder_RootMeanSquared']
 ['original_firstorder_Skewness']
 ['original_glcm_Autocorrelation']
 ['original_glcm_ClusterProminence']
 ['original_glcm_ClusterShade']
 ['original_glcm_ClusterTendency']
 ['original_glcm_Correlation']
 ['original_glcm_DifferenceAverage']
 ['original_glcm_DifferenceEntropy']
 ['original_glcm_Idn']
 ['original_glcm_JointEntropy']
 ['original_glcm_SumSquares']
 ['original_gldm_DependenceNonUniformity']
 ['original_gldm_DependenceVariance']
 ['original_gldm_GrayLevelVariance']
 ['original_gldm_HighGrayLevelEmphasis']
 ['original_gldm_LargeDependenceEmphasis']
 ['original_gldm_LargeDependenceHighGrayLevelEmphasis']
 ['original_gldm_LowGrayLevelEmphasis']
 ['original_gldm_SmallDependenceEmphasis']
 ['original_gldm_SmallDependenceHighGrayLevelEmphasis']
 ['original_gldm_SmallDependenceLowGrayLevelEmphasis']
 ['original_glrlm_GrayLevelNonUniformityNormalized']
 ['original_glrlm_GrayLevelVariance']
 ['original_glrlm_HighGrayLevelRunEmphasis']
 ['original_glrlm_LongRunEmphasis']
 ['original_glrlm_LongRunHighGrayLevelEmphasis']
 ['original_glrlm_LongRunLowGrayLevelEmphasis']
 ['original_glrlm_LowGrayLevelRunEmphasis']
 ['original_glrlm_RunLengthNonUniformityNormalized']
 ['original_glrlm_ShortRunLowGrayLevelEmphasis']
 ['original_glszm_GrayLevelNonUniformity']
 ['original_glszm_GrayLevelVariance']
 ['original_glszm_HighGrayLevelZoneEmphasis']
 ['original_glszm_LargeAreaEmphasis']
 ['original_glszm_LargeAreaHighGrayLevelEmphasis']
 ['original_glszm_SizeZoneNonUniformityNormalized']
 ['original_glszm_SmallAreaHighGrayLevelEmphasis']
 ['original_glszm_SmallAreaLowGrayLevelEmphasis']
 ['original_glszm_ZoneEntropy']
 ['original_glszm_ZonePercentage']
 ['original_ngtdm_Coarseness']
 ['original_ngtdm_Contrast']
 ['wavelet-LLH_firstorder_10Percentile']
 ['wavelet-LLH_firstorder_90Percentile']
 ['wavelet-LLH_firstorder_Energy']
 ['wavelet-LLH_firstorder_Kurtosis']
 ['wavelet-LLH_firstorder_MeanAbsoluteDeviation']
 ['wavelet-LLH_firstorder_Mean']
 ['wavelet-LLH_firstorder_Median']
 ['wavelet-LLH_firstorder_RobustMeanAbsoluteDeviation']
 ['wavelet-LLH_firstorder_TotalEnergy']
 ['wavelet-LLH_firstorder_Uniformity']
 ['wavelet-LLH_firstorder_Variance']
 ['wavelet-LLH_glcm_ClusterTendency']
 ['wavelet-LLH_glcm_Contrast']
 ['wavelet-LLH_glcm_DifferenceEntropy']
 ['wavelet-LLH_glcm_Idm']
 ['wavelet-LLH_glcm_Idmn']
 ['wavelet-LLH_glcm_Idn']
 ['wavelet-LLH_glcm_JointAverage']
 ['wavelet-LLH_glcm_JointEnergy']
 ['wavelet-LLH_glcm_JointEntropy']
 ['wavelet-LLH_glcm_MaximumProbability']
 ['wavelet-LLH_glcm_SumAverage']
 ['wavelet-LLH_glcm_SumSquares']
 ['wavelet-LLH_gldm_GrayLevelNonUniformity']
 ['wavelet-LLH_gldm_HighGrayLevelEmphasis']
 ['wavelet-LLH_gldm_LargeDependenceEmphasis']
 ['wavelet-LLH_gldm_SmallDependenceHighGrayLevelEmphasis']
 ['wavelet-LLH_gldm_SmallDependenceLowGrayLevelEmphasis']
 ['wavelet-LLH_glrlm_GrayLevelVariance']
 ['wavelet-LLH_glrlm_LongRunEmphasis']
 ['wavelet-LLH_glrlm_LongRunHighGrayLevelEmphasis']
 ['wavelet-LLH_glrlm_LongRunLowGrayLevelEmphasis']
 ['wavelet-LLH_glrlm_RunEntropy']
 ['wavelet-LLH_glrlm_RunLengthNonUniformityNormalized']
 ['wavelet-LLH_glrlm_RunPercentage']
 ['wavelet-LLH_glrlm_ShortRunEmphasis']
 ['wavelet-LLH_glrlm_ShortRunLowGrayLevelEmphasis']
 ['wavelet-LLH_glszm_HighGrayLevelZoneEmphasis']
 ['wavelet-LLH_glszm_LargeAreaEmphasis']
 ['wavelet-LLH_glszm_LargeAreaHighGrayLevelEmphasis']
 ['wavelet-LLH_glszm_LargeAreaLowGrayLevelEmphasis']
 ['wavelet-LLH_glszm_SizeZoneNonUniformity']
 ['wavelet-LLH_glszm_SizeZoneNonUniformityNormalized']
 ['wavelet-LLH_glszm_SmallAreaHighGrayLevelEmphasis']
 ['wavelet-LLH_glszm_SmallAreaLowGrayLevelEmphasis']
 ['wavelet-LHL_firstorder_90Percentile']
 ['wavelet-LHL_firstorder_Entropy']
 ['wavelet-LHL_firstorder_Kurtosis']
 ['wavelet-LHL_firstorder_Maximum']
 ['wavelet-LHL_firstorder_MeanAbsoluteDeviation']
 ['wavelet-LHL_firstorder_Mean']
 ['wavelet-LHL_firstorder_Minimum']
 ['wavelet-LHL_firstorder_RobustMeanAbsoluteDeviation']
 ['wavelet-LHL_firstorder_Skewness']
 ['wavelet-LHL_firstorder_Uniformity']
 ['wavelet-LHL_firstorder_Variance']
 ['wavelet-LHL_glcm_Autocorrelation']
 ['wavelet-LHL_glcm_ClusterShade']
 ['wavelet-LHL_glcm_Contrast']
 ['wavelet-LHL_glcm_Correlation']
 ['wavelet-LHL_glcm_DifferenceVariance']
 ['wavelet-LHL_glcm_Idm']
 ['wavelet-LHL_glcm_JointEntropy']
 ['wavelet-LHL_glcm_MaximumProbability']
 ['wavelet-LHL_gldm_DependenceNonUniformityNormalized']
 ['wavelet-LHL_gldm_DependenceVariance']
 ['wavelet-LHL_gldm_HighGrayLevelEmphasis']
 ['wavelet-LHL_gldm_LargeDependenceEmphasis']
 ['wavelet-LHL_gldm_LargeDependenceHighGrayLevelEmphasis']
 ['wavelet-LHL_glrlm_GrayLevelNonUniformity']
 ['wavelet-LHL_glrlm_LongRunLowGrayLevelEmphasis']
 ['wavelet-LHL_glrlm_LowGrayLevelRunEmphasis']
 ['wavelet-LHL_glrlm_RunLengthNonUniformityNormalized']
 ['wavelet-LHL_glrlm_RunVariance']
 ['wavelet-LHL_glrlm_ShortRunLowGrayLevelEmphasis']
 ['wavelet-LHL_glszm_GrayLevelNonUniformity']
 ['wavelet-LHL_glszm_GrayLevelNonUniformityNormalized']
 ['wavelet-LHL_glszm_GrayLevelVariance']
 ['wavelet-LHL_glszm_LargeAreaEmphasis']
 ['wavelet-LHL_glszm_LargeAreaHighGrayLevelEmphasis']
 ['wavelet-LHL_glszm_LargeAreaLowGrayLevelEmphasis']
 ['wavelet-LHL_glszm_SizeZoneNonUniformity']
 ['wavelet-LHL_glszm_SmallAreaEmphasis']
 ['wavelet-LHL_glszm_SmallAreaHighGrayLevelEmphasis']
 ['wavelet-LHL_glszm_ZonePercentage']
 ['wavelet-LHL_ngtdm_Busyness']
 ['wavelet-LHL_ngtdm_Strength']
 ['wavelet-LHH_firstorder_10Percentile']
 ['wavelet-LHH_firstorder_Entropy']
 ['wavelet-LHH_firstorder_InterquartileRange']
 ['wavelet-LHH_firstorder_Maximum']
 ['wavelet-LHH_firstorder_MeanAbsoluteDeviation']
 ['wavelet-LHH_firstorder_Mean']
 ['wavelet-LHH_firstorder_Median']
 ['wavelet-LHH_firstorder_Minimum']
 ['wavelet-LHH_firstorder_RootMeanSquared']
 ['wavelet-LHH_firstorder_Skewness']
 ['wavelet-LHH_firstorder_Uniformity']
 ['wavelet-LHH_glcm_ClusterProminence']
 ['wavelet-LHH_glcm_DifferenceVariance']
 ['wavelet-LHH_glcm_Id']
 ['wavelet-LHH_glcm_Idn']
 ['wavelet-LHH_glcm_Imc2']
 ['wavelet-LHH_glcm_InverseVariance']
 ['wavelet-LHH_glcm_JointEnergy']
 ['wavelet-LHH_glcm_JointEntropy']
 ['wavelet-LHH_glcm_MCC']
 ['wavelet-LHH_glcm_SumSquares']
 ['wavelet-LHH_gldm_DependenceEntropy']
 ['wavelet-LHH_gldm_DependenceNonUniformity']
 ['wavelet-LHH_gldm_GrayLevelVariance']
 ['wavelet-LHH_gldm_HighGrayLevelEmphasis']
 ['wavelet-LHH_gldm_LargeDependenceEmphasis']
 ['wavelet-LHH_gldm_LargeDependenceLowGrayLevelEmphasis']
 ['wavelet-LHH_gldm_SmallDependenceHighGrayLevelEmphasis']
 ['wavelet-LHH_gldm_SmallDependenceLowGrayLevelEmphasis']
 ['wavelet-LHH_glrlm_GrayLevelNonUniformity']
 ['wavelet-LHH_glrlm_GrayLevelNonUniformityNormalized']
 ['wavelet-LHH_glrlm_GrayLevelVariance']
 ['wavelet-LHH_glrlm_HighGrayLevelRunEmphasis']
 ['wavelet-LHH_glrlm_LongRunHighGrayLevelEmphasis']
 ['wavelet-LHH_glrlm_RunEntropy']
 ['wavelet-LHH_glrlm_RunLengthNonUniformity']
 ['wavelet-LHH_glrlm_RunLengthNonUniformityNormalized']
 ['wavelet-LHH_glrlm_RunVariance']
 ['wavelet-LHH_glszm_LargeAreaHighGrayLevelEmphasis']
 ['wavelet-LHH_glszm_LowGrayLevelZoneEmphasis']
 ['wavelet-LHH_glszm_SizeZoneNonUniformity']
 ['wavelet-LHH_glszm_SmallAreaEmphasis']
 ['wavelet-LHH_ngtdm_Busyness']
 ['wavelet-LHH_ngtdm_Strength']
 ['wavelet-HLL_firstorder_90Percentile']
 ['wavelet-HLL_firstorder_Entropy']
 ['wavelet-HLL_firstorder_Maximum']
 ['wavelet-HLL_firstorder_MeanAbsoluteDeviation']
 ['wavelet-HLL_firstorder_Median']
 ['wavelet-HLL_firstorder_Minimum']
 ['wavelet-HLL_firstorder_Range']
 ['wavelet-HLL_firstorder_RobustMeanAbsoluteDeviation']
 ['wavelet-HLL_firstorder_RootMeanSquared']
 ['wavelet-HLL_firstorder_Uniformity']
 ['wavelet-HLL_glcm_ClusterProminence']
 ['wavelet-HLL_glcm_Contrast']
 ['wavelet-HLL_glcm_DifferenceAverage']
 ['wavelet-HLL_glcm_Idmn']
 ['wavelet-HLL_glcm_Idn']
 ['wavelet-HLL_glcm_Imc1']
 ['wavelet-HLL_glcm_Imc2']
 ['wavelet-HLL_glcm_JointAverage']
 ['wavelet-HLL_glcm_JointEntropy']
 ['wavelet-HLL_glcm_SumAverage']
 ['wavelet-HLL_glcm_SumEntropy']
 ['wavelet-HLL_gldm_DependenceNonUniformity']
 ['wavelet-HLL_gldm_DependenceVariance']
 ['wavelet-HLL_gldm_GrayLevelVariance']
 ['wavelet-HLL_gldm_LargeDependenceEmphasis']
 ['wavelet-HLL_gldm_LowGrayLevelEmphasis']
 ['wavelet-HLL_gldm_SmallDependenceEmphasis']
 ['wavelet-HLL_gldm_SmallDependenceLowGrayLevelEmphasis']
 ['wavelet-HLL_glrlm_GrayLevelVariance']
 ['wavelet-HLL_glrlm_HighGrayLevelRunEmphasis']
 ['wavelet-HLL_glrlm_LongRunEmphasis']
 ['wavelet-HLL_glrlm_LongRunLowGrayLevelEmphasis']
 ['wavelet-HLL_glrlm_RunPercentage']
 ['wavelet-HLL_glrlm_RunVariance']
 ['wavelet-HLL_glrlm_ShortRunLowGrayLevelEmphasis']
 ['wavelet-HLL_glszm_GrayLevelNonUniformityNormalized']
 ['wavelet-HLL_glszm_GrayLevelVariance']
 ['wavelet-HLL_glszm_LargeAreaEmphasis']
 ['wavelet-HLL_glszm_LargeAreaLowGrayLevelEmphasis']
 ['wavelet-HLL_glszm_SizeZoneNonUniformity']
 ['wavelet-HLL_glszm_SmallAreaEmphasis']
 ['wavelet-HLL_glszm_SmallAreaLowGrayLevelEmphasis']
 ['wavelet-HLL_glszm_ZoneVariance']
 ['wavelet-HLL_ngtdm_Busyness']
 ['wavelet-HLL_ngtdm_Coarseness']
 ['wavelet-HLH_firstorder_10Percentile']
 ['wavelet-HLH_firstorder_90Percentile']
 ['wavelet-HLH_firstorder_Entropy']
 ['wavelet-HLH_firstorder_InterquartileRange']
 ['wavelet-HLH_firstorder_Kurtosis']
 ['wavelet-HLH_firstorder_Mean']
 ['wavelet-HLH_firstorder_RobustMeanAbsoluteDeviation']
 ['wavelet-HLH_glcm_Autocorrelation']
 ['wavelet-HLH_glcm_ClusterShade']
 ['wavelet-HLH_glcm_Correlation']
 ['wavelet-HLH_glcm_DifferenceAverage']
 ['wavelet-HLH_glcm_DifferenceVariance']
 ['wavelet-HLH_glcm_Id']
 ['wavelet-HLH_glcm_Idmn']
 ['wavelet-HLH_glcm_JointAverage']
 ['wavelet-HLH_glcm_MCC']
 ['wavelet-HLH_glcm_MaximumProbability']
 ['wavelet-HLH_glcm_SumAverage']
 ['wavelet-HLH_glcm_SumSquares']
 ['wavelet-HLH_gldm_DependenceEntropy']
 ['wavelet-HLH_gldm_DependenceVariance']
 ['wavelet-HLH_gldm_GrayLevelNonUniformity']
 ['wavelet-HLH_gldm_LargeDependenceEmphasis']
 ['wavelet-HLH_gldm_LargeDependenceHighGrayLevelEmphasis']
 ['wavelet-HLH_gldm_LowGrayLevelEmphasis']
 ['wavelet-HLH_glrlm_GrayLevelNonUniformity']
 ['wavelet-HLH_glrlm_GrayLevelVariance']
 ['wavelet-HLH_glrlm_HighGrayLevelRunEmphasis']
 ['wavelet-HLH_glrlm_LongRunHighGrayLevelEmphasis']
 ['wavelet-HLH_glrlm_LongRunLowGrayLevelEmphasis']
 ['wavelet-HLH_glrlm_LowGrayLevelRunEmphasis']
 ['wavelet-HLH_glrlm_RunEntropy']
 ['wavelet-HLH_glrlm_RunLengthNonUniformityNormalized']
 ['wavelet-HLH_glrlm_RunPercentage']
 ['wavelet-HLH_glrlm_RunVariance']
 ['wavelet-HLH_glrlm_ShortRunLowGrayLevelEmphasis']
 ['wavelet-HLH_glszm_LargeAreaHighGrayLevelEmphasis']
 ['wavelet-HLH_glszm_LargeAreaLowGrayLevelEmphasis']
 ['wavelet-HLH_glszm_SizeZoneNonUniformity']
 ['wavelet-HLH_glszm_SmallAreaEmphasis']
 ['wavelet-HLH_glszm_SmallAreaHighGrayLevelEmphasis']
 ['wavelet-HLH_glszm_SmallAreaLowGrayLevelEmphasis']
 ['wavelet-HLH_glszm_ZoneEntropy']
 ['wavelet-HLH_glszm_ZonePercentage']
 ['wavelet-HLH_glszm_ZoneVariance']
 ['wavelet-HLH_ngtdm_Busyness']
 ['wavelet-HLH_ngtdm_Coarseness']
 ['wavelet-HLH_ngtdm_Contrast']
 ['wavelet-HHL_firstorder_10Percentile']
 ['wavelet-HHL_firstorder_90Percentile']
 ['wavelet-HHL_firstorder_InterquartileRange']
 ['wavelet-HHL_firstorder_Kurtosis']
 ['wavelet-HHL_firstorder_Mean']
 ['wavelet-HHL_firstorder_Minimum']
 ['wavelet-HHL_firstorder_RobustMeanAbsoluteDeviation']
 ['wavelet-HHL_firstorder_RootMeanSquared']
 ['wavelet-HHL_firstorder_Skewness']
 ['wavelet-HHL_firstorder_Uniformity']
 ['wavelet-HHL_glcm_ClusterShade']
 ['wavelet-HHL_glcm_ClusterTendency']
 ['wavelet-HHL_glcm_DifferenceAverage']
 ['wavelet-HHL_glcm_Id']
 ['wavelet-HHL_glcm_Idn']
 ['wavelet-HHL_glcm_Imc1']
 ['wavelet-HHL_glcm_Imc2']
 ['wavelet-HHL_glcm_JointAverage']
 ['wavelet-HHL_glcm_JointEnergy']
 ['wavelet-HHL_glcm_SumAverage']
 ['wavelet-HHL_glcm_SumSquares']
 ['wavelet-HHL_gldm_DependenceVariance']
 ['wavelet-HHL_gldm_GrayLevelNonUniformity']
 ['wavelet-HHL_gldm_GrayLevelVariance']
 ['wavelet-HHL_gldm_HighGrayLevelEmphasis']
 ['wavelet-HHL_gldm_LargeDependenceHighGrayLevelEmphasis']
 ['wavelet-HHL_gldm_LargeDependenceLowGrayLevelEmphasis']
 ['wavelet-HHL_gldm_SmallDependenceEmphasis']
 ['wavelet-HHL_gldm_SmallDependenceLowGrayLevelEmphasis']
 ['wavelet-HHL_glrlm_GrayLevelNonUniformity']
 ['wavelet-HHL_glrlm_GrayLevelNonUniformityNormalized']
 ['wavelet-HHL_glrlm_HighGrayLevelRunEmphasis']
 ['wavelet-HHL_glrlm_LongRunEmphasis']
 ['wavelet-HHL_glrlm_LongRunHighGrayLevelEmphasis']
 ['wavelet-HHL_glrlm_LongRunLowGrayLevelEmphasis']
 ['wavelet-HHL_glrlm_RunPercentage']
 ['wavelet-HHL_glrlm_RunVariance']
 ['wavelet-HHL_glrlm_ShortRunHighGrayLevelEmphasis']
 ['wavelet-HHL_glrlm_ShortRunLowGrayLevelEmphasis']
 ['wavelet-HHL_glszm_GrayLevelVariance']
 ['wavelet-HHL_glszm_HighGrayLevelZoneEmphasis']
 ['wavelet-HHL_glszm_LargeAreaEmphasis']
 ['wavelet-HHL_glszm_LargeAreaLowGrayLevelEmphasis']
 ['wavelet-HHL_glszm_LowGrayLevelZoneEmphasis']
 ['wavelet-HHL_glszm_SizeZoneNonUniformity']
 ['wavelet-HHL_glszm_SizeZoneNonUniformityNormalized']
 ['wavelet-HHL_glszm_ZoneEntropy']
 ['wavelet-HHL_glszm_ZonePercentage']
 ['wavelet-HHL_ngtdm_Busyness']
 ['wavelet-HHL_ngtdm_Coarseness']
 ['wavelet-HHL_ngtdm_Complexity']
 ['wavelet-HHL_ngtdm_Contrast']
 ['wavelet-HHL_ngtdm_Strength']
 ['wavelet-HHH_firstorder_10Percentile']
 ['wavelet-HHH_firstorder_Energy']
 ['wavelet-HHH_firstorder_Entropy']
 ['wavelet-HHH_firstorder_InterquartileRange']
 ['wavelet-HHH_firstorder_MeanAbsoluteDeviation']
 ['wavelet-HHH_firstorder_Median']
 ['wavelet-HHH_firstorder_Range']
 ['wavelet-HHH_firstorder_RootMeanSquared']
 ['wavelet-HHH_firstorder_TotalEnergy']
 ['wavelet-HHH_firstorder_Uniformity']
 ['wavelet-HHH_glcm_Autocorrelation']
 ['wavelet-HHH_glcm_Contrast']
 ['wavelet-HHH_glcm_Correlation']
 ['wavelet-HHH_glcm_DifferenceAverage']
 ['wavelet-HHH_glcm_DifferenceVariance']
 ['wavelet-HHH_glcm_Id']
 ['wavelet-HHH_glcm_Idn']
 ['wavelet-HHH_glcm_Imc1']
 ['wavelet-HHH_glcm_Imc2']
 ['wavelet-HHH_glcm_InverseVariance']
 ['wavelet-HHH_glcm_JointEntropy']
 ['wavelet-HHH_glcm_MCC']
 ['wavelet-HHH_glcm_SumEntropy']
 ['wavelet-HHH_glcm_SumSquares']
 ['wavelet-HHH_gldm_DependenceEntropy']
 ['wavelet-HHH_gldm_GrayLevelNonUniformity']
 ['wavelet-HHH_gldm_GrayLevelVariance']
 ['wavelet-HHH_gldm_HighGrayLevelEmphasis']
 ['wavelet-HHH_gldm_LowGrayLevelEmphasis']
 ['wavelet-HHH_gldm_SmallDependenceHighGrayLevelEmphasis']
 ['wavelet-HHH_glrlm_GrayLevelVariance']
 ['wavelet-HHH_glrlm_HighGrayLevelRunEmphasis']
 ['wavelet-HHH_glrlm_LongRunHighGrayLevelEmphasis']
 ['wavelet-HHH_glrlm_LongRunLowGrayLevelEmphasis']
 ['wavelet-HHH_glrlm_RunLengthNonUniformityNormalized']
 ['wavelet-HHH_glrlm_RunPercentage']
 ['wavelet-HHH_glrlm_RunVariance']
 ['wavelet-HHH_glszm_HighGrayLevelZoneEmphasis']
 ['wavelet-HHH_glszm_LargeAreaEmphasis']
 ['wavelet-HHH_glszm_SizeZoneNonUniformity']
 ['wavelet-HHH_glszm_SmallAreaEmphasis']
 ['wavelet-HHH_glszm_ZonePercentage']
 ['wavelet-HHH_ngtdm_Strength']
 ['wavelet-LLL_firstorder_Entropy']
 ['wavelet-LLL_firstorder_Maximum']
 ['wavelet-LLL_firstorder_MeanAbsoluteDeviation']
 ['wavelet-LLL_firstorder_Mean']
 ['wavelet-LLL_firstorder_Median']
 ['wavelet-LLL_firstorder_Range']
 ['wavelet-LLL_firstorder_Uniformity']
 ['wavelet-LLL_firstorder_Variance']
 ['wavelet-LLL_glcm_Autocorrelation']
 ['wavelet-LLL_glcm_ClusterTendency']
 ['wavelet-LLL_glcm_Correlation']
 ['wavelet-LLL_glcm_DifferenceEntropy']
 ['wavelet-LLL_glcm_Idmn']
 ['wavelet-LLL_glcm_Imc1']
 ['wavelet-LLL_glcm_Imc2']
 ['wavelet-LLL_glcm_InverseVariance']
 ['wavelet-LLL_glcm_JointEnergy']
 ['wavelet-LLL_glcm_JointEntropy']
 ['wavelet-LLL_glcm_MaximumProbability']
 ['wavelet-LLL_gldm_DependenceEntropy']
 ['wavelet-LLL_gldm_DependenceVariance']
 ['wavelet-LLL_gldm_GrayLevelNonUniformity']
 ['wavelet-LLL_gldm_GrayLevelVariance']
 ['wavelet-LLL_gldm_LargeDependenceHighGrayLevelEmphasis']
 ['wavelet-LLL_gldm_LargeDependenceLowGrayLevelEmphasis']
 ['wavelet-LLL_gldm_LowGrayLevelEmphasis']
 ['wavelet-LLL_gldm_SmallDependenceEmphasis']
 ['wavelet-LLL_gldm_SmallDependenceHighGrayLevelEmphasis']
 ['wavelet-LLL_glrlm_GrayLevelNonUniformity']
 ['wavelet-LLL_glrlm_HighGrayLevelRunEmphasis']
 ['wavelet-LLL_glrlm_LongRunEmphasis']
 ['wavelet-LLL_glrlm_LongRunHighGrayLevelEmphasis']
 ['wavelet-LLL_glrlm_LowGrayLevelRunEmphasis']
 ['wavelet-LLL_glrlm_RunEntropy']
 ['wavelet-LLL_glrlm_ShortRunEmphasis']
 ['wavelet-LLL_glszm_GrayLevelNonUniformity']
 ['wavelet-LLL_glszm_GrayLevelNonUniformityNormalized']
 ['wavelet-LLL_glszm_GrayLevelVariance']
 ['wavelet-LLL_glszm_LargeAreaHighGrayLevelEmphasis']
 ['wavelet-LLL_glszm_LargeAreaLowGrayLevelEmphasis']
 ['wavelet-LLL_glszm_LowGrayLevelZoneEmphasis']
 ['wavelet-LLL_glszm_SizeZoneNonUniformity']
 ['wavelet-LLL_glszm_SizeZoneNonUniformityNormalized']
 ['wavelet-LLL_glszm_SmallAreaLowGrayLevelEmphasis']
 ['wavelet-LLL_glszm_ZonePercentage']
 ['wavelet-LLL_ngtdm_Coarseness']
 ['wavelet-LLL_ngtdm_Strength']]

Heatmap of the model in the training samples:


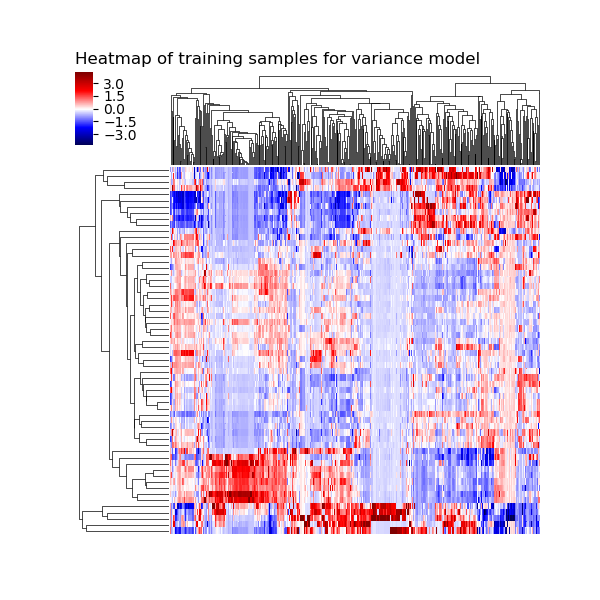


Heatmap of the model in the testing samples:


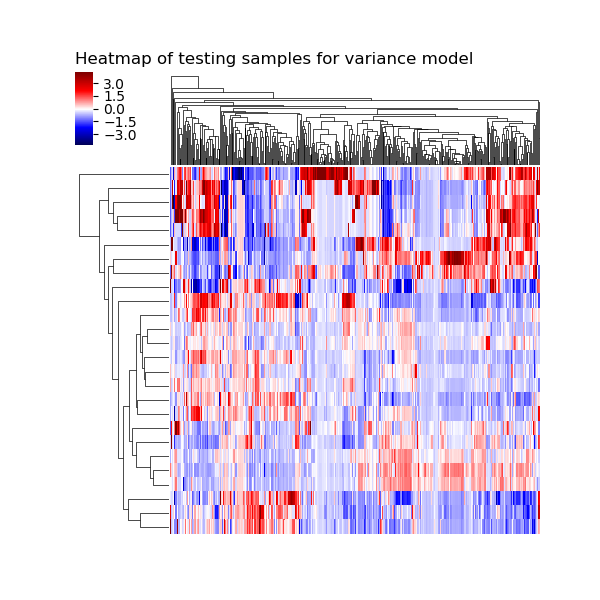


Correlation coefficient figure of the training samples

Correlation coefficient figure of the testing samples

# 7. The method for selecting features: Correlation_xx

parameters setted: {'cutoff': 0.7}
num of remained features: 76
remained features:
[['original_shape_Flatness']
 ['original_firstorder_Minimum']
 ['original_glcm_ClusterShade']
 ['original_glrlm_LongRunLowGrayLevelEmphasis']
 ['original_glszm_GrayLevelVariance']
 ['original_glszm_LargeAreaHighGrayLevelEmphasis']
 ['original_glszm_SmallAreaLowGrayLevelEmphasis']
 ['original_ngtdm_Contrast']
 ['wavelet-LLH_firstorder_10Percentile']
 ['wavelet-LLH_firstorder_90Percentile']
 ['wavelet-LLH_firstorder_Kurtosis']
 ['wavelet-LLH_firstorder_Median']
 ['wavelet-LLH_glcm_SumAverage']
 ['wavelet-LLH_glrlm_LongRunHighGrayLevelEmphasis']
 ['wavelet-LLH_glszm_SizeZoneNonUniformityNormalized']
 ['wavelet-LLH_glszm_SmallAreaHighGrayLevelEmphasis']
 ['wavelet-LLH_glszm_SmallAreaLowGrayLevelEmphasis']
 ['wavelet-LHL_firstorder_90Percentile']
 ['wavelet-LHL_firstorder_Mean']
 ['wavelet-LHL_firstorder_Skewness']
 ['wavelet-LHL_glcm_ClusterShade']
 ['wavelet-LHL_glcm_Contrast']
 ['wavelet-LHL_glcm_Correlation']
 ['wavelet-LHL_glcm_DifferenceVariance']
 ['wavelet-LHL_glrlm_LongRunLowGrayLevelEmphasis']
 ['wavelet-LHL_glszm_GrayLevelNonUniformityNormalized']
 ['wavelet-LHL_glszm_LargeAreaLowGrayLevelEmphasis']
 ['wavelet-LHH_firstorder_Maximum']
 ['wavelet-LHH_firstorder_Skewness']
 ['wavelet-LHH_glcm_InverseVariance']
 ['wavelet-LHH_gldm_LargeDependenceLowGrayLevelEmphasis']
 ['wavelet-LHH_glszm_SmallAreaEmphasis']
 ['wavelet-LHH_ngtdm_Busyness']
 ['wavelet-HLL_firstorder_90Percentile']
 ['wavelet-HLL_firstorder_Median']
 ['wavelet-HLL_glcm_ClusterProminence']
 ['wavelet-HLL_glcm_Imc2']
 ['wavelet-HLL_glszm_GrayLevelNonUniformityNormalized']
 ['wavelet-HLL_glszm_LargeAreaLowGrayLevelEmphasis']
 ['wavelet-HLL_glszm_SmallAreaEmphasis']
 ['wavelet-HLL_glszm_SmallAreaLowGrayLevelEmphasis']
 ['wavelet-HLH_firstorder_Mean']
 ['wavelet-HLH_glcm_Correlation']
 ['wavelet-HLH_glcm_MaximumProbability']
 ['wavelet-HLH_gldm_DependenceEntropy']
 ['wavelet-HLH_gldm_DependenceVariance']
 ['wavelet-HLH_glrlm_LongRunLowGrayLevelEmphasis']
 ['wavelet-HLH_glszm_SmallAreaEmphasis']
 ['wavelet-HLH_glszm_SmallAreaHighGrayLevelEmphasis']
 ['wavelet-HLH_glszm_SmallAreaLowGrayLevelEmphasis']
 ['wavelet-HLH_ngtdm_Busyness']
 ['wavelet-HHL_firstorder_Mean']
 ['wavelet-HHL_firstorder_Skewness']
 ['wavelet-HHL_glcm_ClusterShade']
 ['wavelet-HHL_glcm_ClusterTendency']
 ['wavelet-HHL_glcm_Imc2']
 ['wavelet-HHL_gldm_DependenceVariance']
 ['wavelet-HHL_gldm_LargeDependenceLowGrayLevelEmphasis']
 ['wavelet-HHL_glrlm_ShortRunHighGrayLevelEmphasis']
 ['wavelet-HHL_glszm_LargeAreaLowGrayLevelEmphasis']
 ['wavelet-HHL_glszm_SizeZoneNonUniformityNormalized']
 ['wavelet-HHL_ngtdm_Coarseness']
 ['wavelet-HHL_ngtdm_Strength']
 ['wavelet-HHH_firstorder_Median']
 ['wavelet-HHH_glcm_Correlation']
 ['wavelet-HHH_glcm_DifferenceAverage']
 ['wavelet-HHH_glcm_Idn']
 ['wavelet-HHH_glcm_MCC']
 ['wavelet-HHH_gldm_DependenceEntropy']
 ['wavelet-HHH_glszm_SizeZoneNonUniformity']
 ['wavelet-HHH_glszm_SmallAreaEmphasis']
 ['wavelet-LLL_glcm_JointEnergy']
 ['wavelet-LLL_gldm_LargeDependenceLowGrayLevelEmphasis']
 ['wavelet-LLL_glszm_LargeAreaLowGrayLevelEmphasis']
 ['wavelet-LLL_glszm_SizeZoneNonUniformityNormalized']
 ['wavelet-LLL_ngtdm_Strength']]

Heatmap of the model in the training samples:


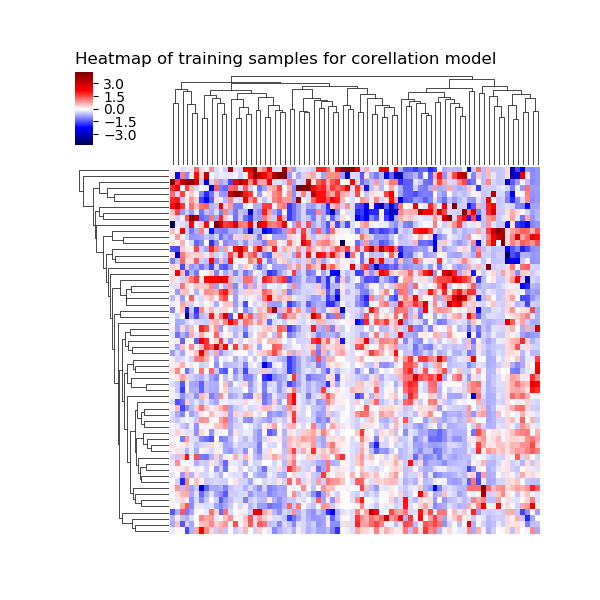


Heatmap of the model in the testing samples:


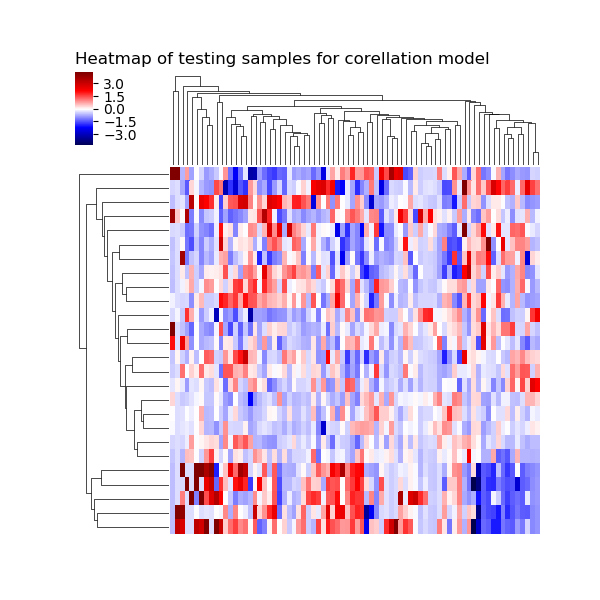


Correlation coefficient figure of the training samples

Correlation coefficient figure of the testing samples

# 8. The method for selecting features: MultiVariate_Logistic

parameters setted: {'P value for threshold in': 0.05, 'P value for threshold out': 0.1}
num of remained features: 2
remained features:
[['wavelet-LHL_glrlm_LongRunLowGrayLevelEmphasis']
 ['wavelet-LLH_firstorder_10Percentile']]

Statistical analysis of logistic multivariate analysis:

| feature | OR | 0.025 | 0.975 | P_value |
| --- | --- | --- | --- | --- |
| const | 0.44 | 0.238 | 0.814 | nan |
| wavelet-LHL_glrlm_LongRunLowGrayLevelEmphasis | 2.492 | 1.308 | 4.748 | 0.006 |
| wavelet-LLH_firstorder_10Percentile | 0.437 | 0.227 | 0.842 | 0.013 |

Heatmap of the model in the training samples:


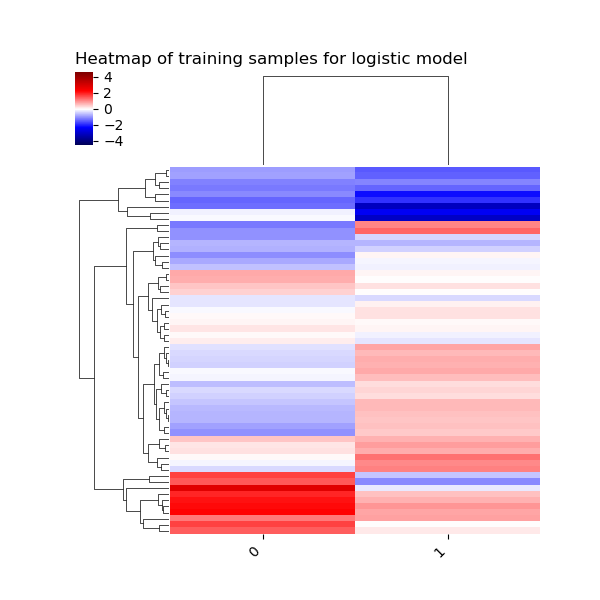


Heatmap of the model in the testing samples:


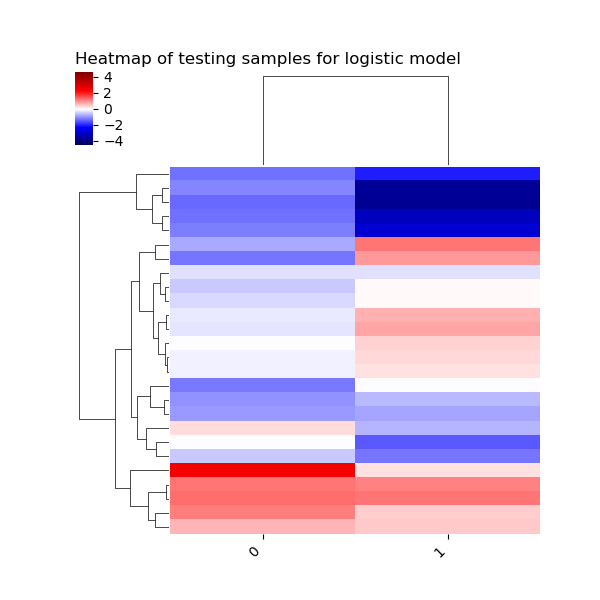


Correlation coefficient figure of the training samples


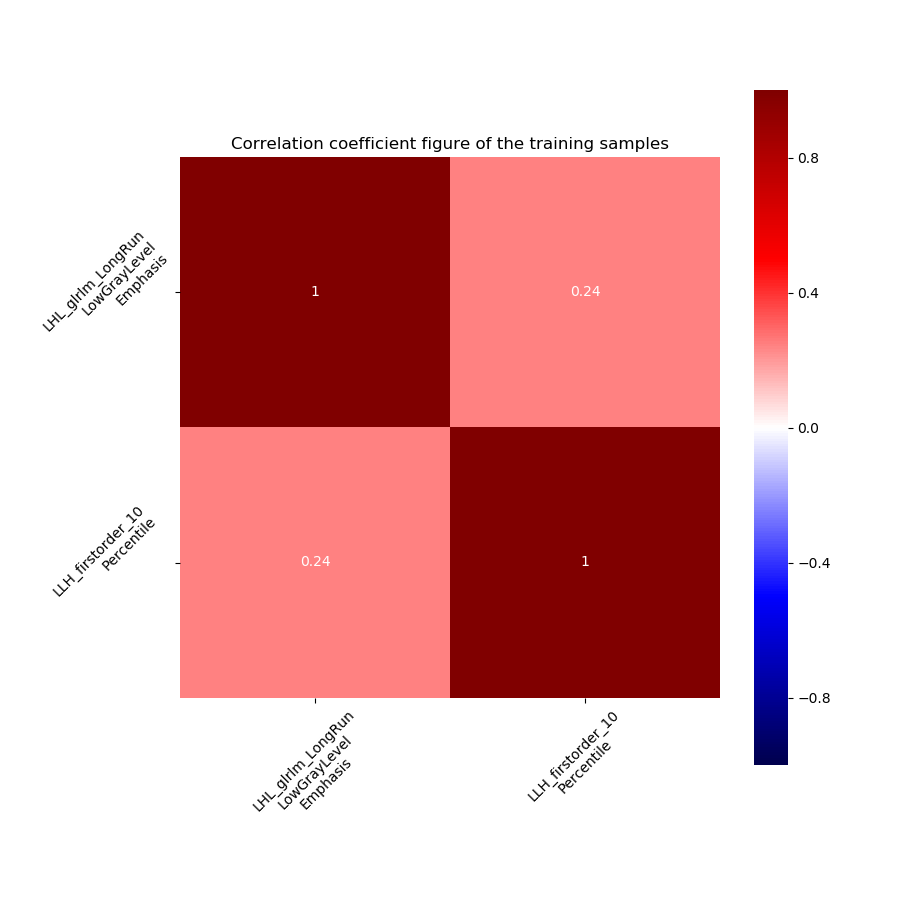


Correlation coefficient figure of the testing samples


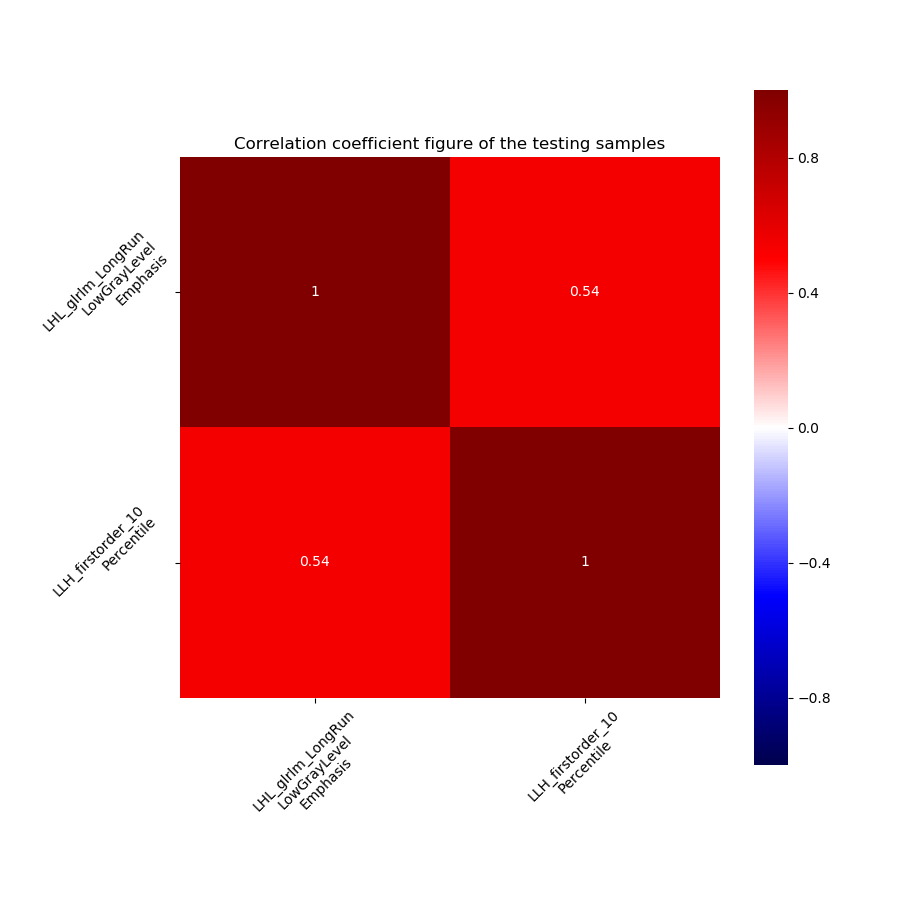


# 9. The best machine learning method: Forest

the mechine learning method: Forest

the parameters selection method and selected parameters:
 {'Method': 'auto', 'max_depth': 1, 'min_samples_split': 2, 'n_estimators': 15}

the separate scores and total mean scores of model in each validation fold:

【Note】the validation cross process has not been operated.

evaluation of the Forest model in the training and testing samples:

| Item | Train | Test |
| --- | --- | --- |
| Accuracy | 0.75 | 0.692 |
| f1_score | 0.483 | 0.2 |
| Recall | 0.35 | 0.111 |
| Precision | 0.778 | 1.0 |
| AUC | 0.773 (0.659, 0.878) | 0.627 (0.441, 0.823) |
| Sensitivity | 0.35 | 0.111 |
| Specificity | 0.95 | 1.0 |
| positive prediction | 0.778 | 1.0 |
| negative prediction | 0.745 | 0.68 |
| positive llr | 7.0 | inf |
| negatice llr | 0.684 | 0.889 |

ROC of the Forest model in the training samples:


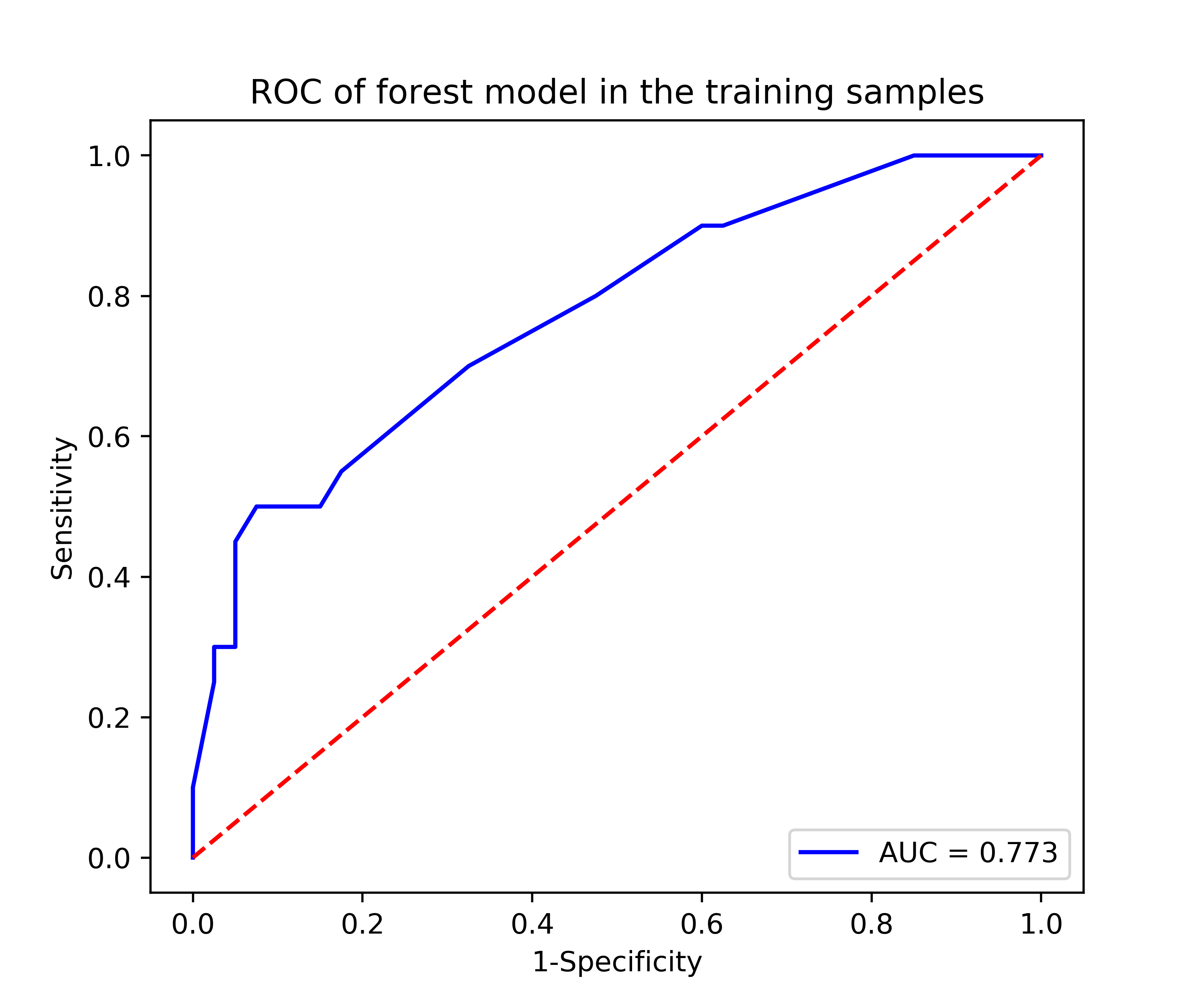


ROC of the Forest model in the testing samples:


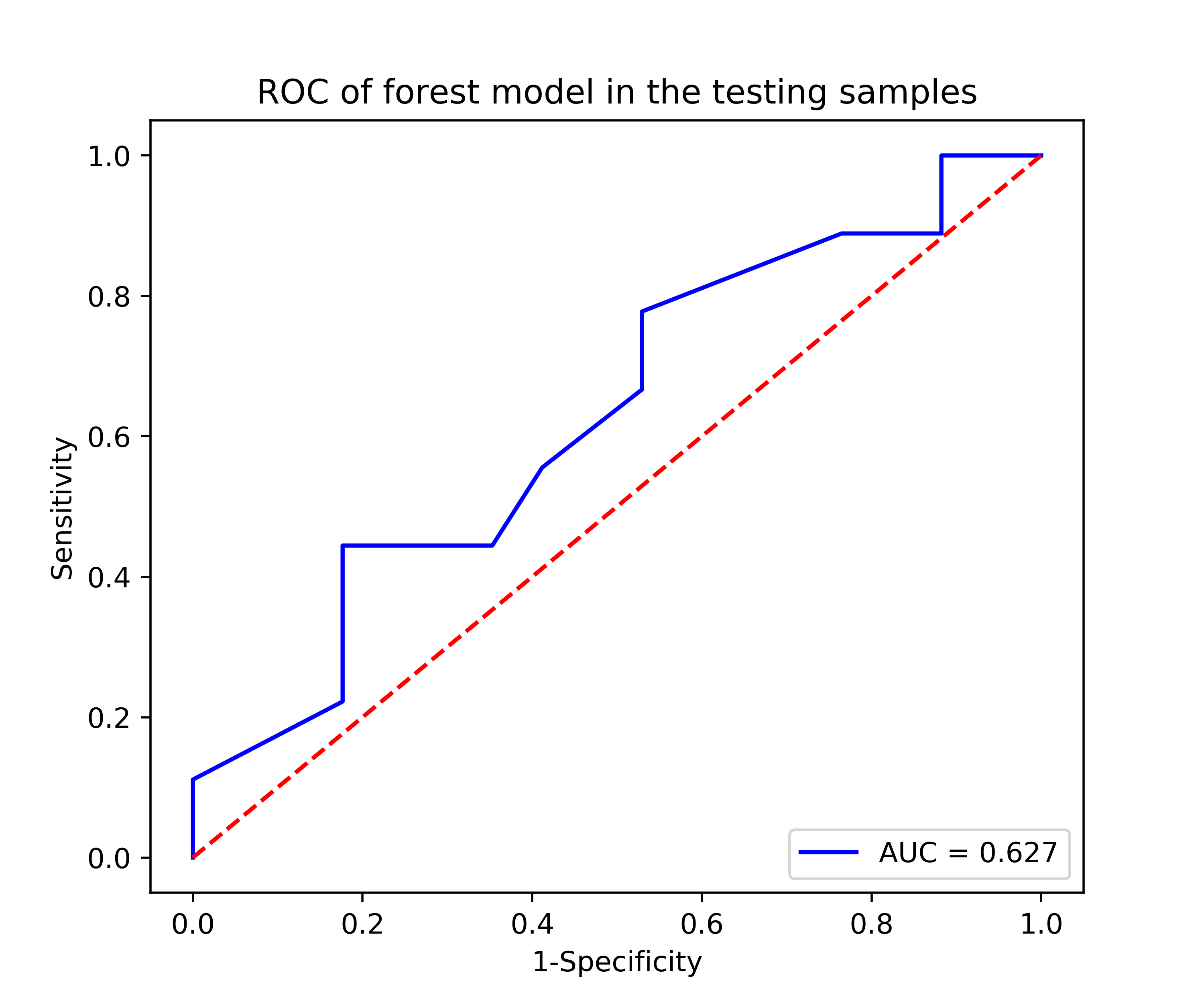


Decision Curve of Forest model in training samples:


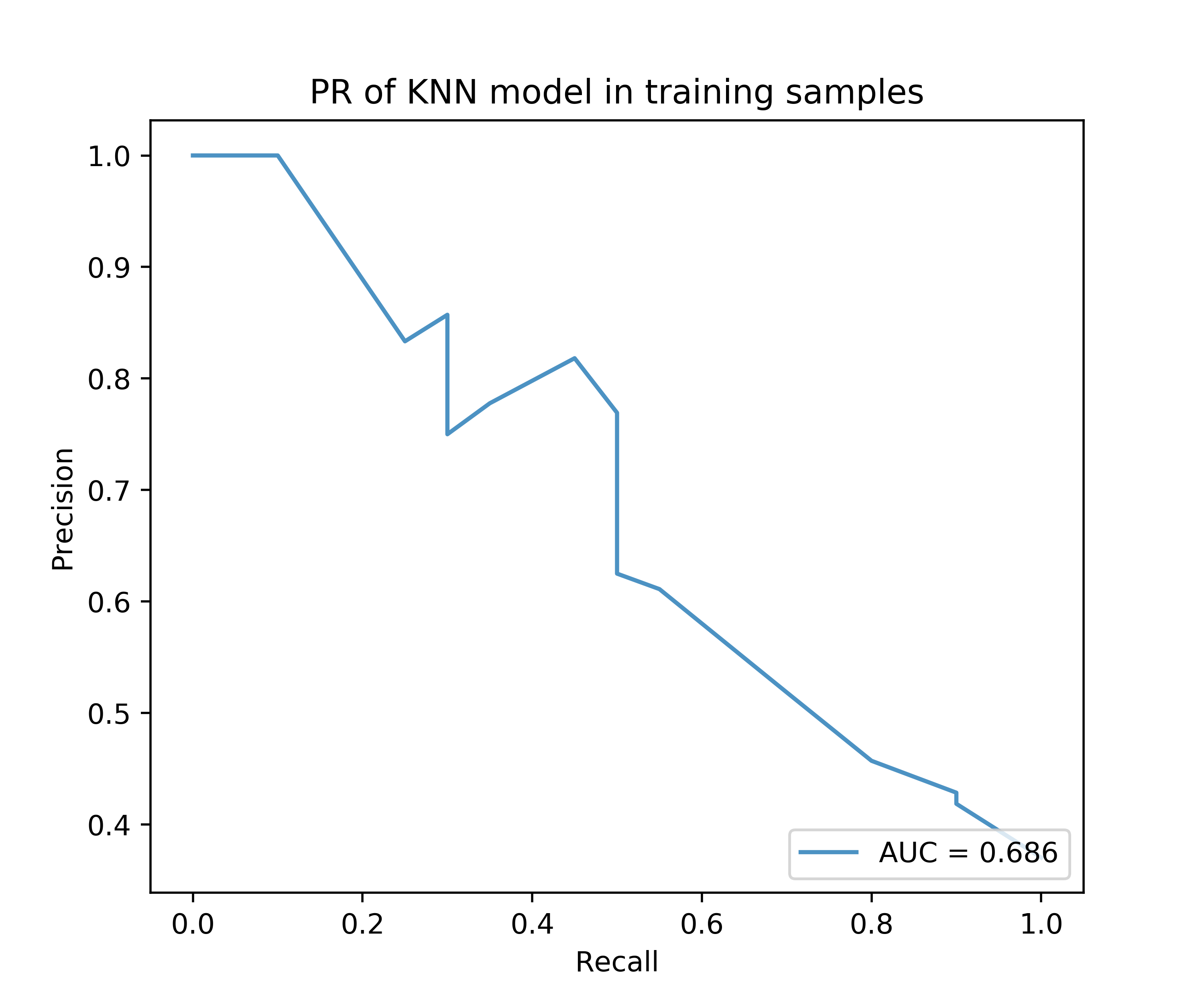


Decision Curve of Forest model in testing samples:


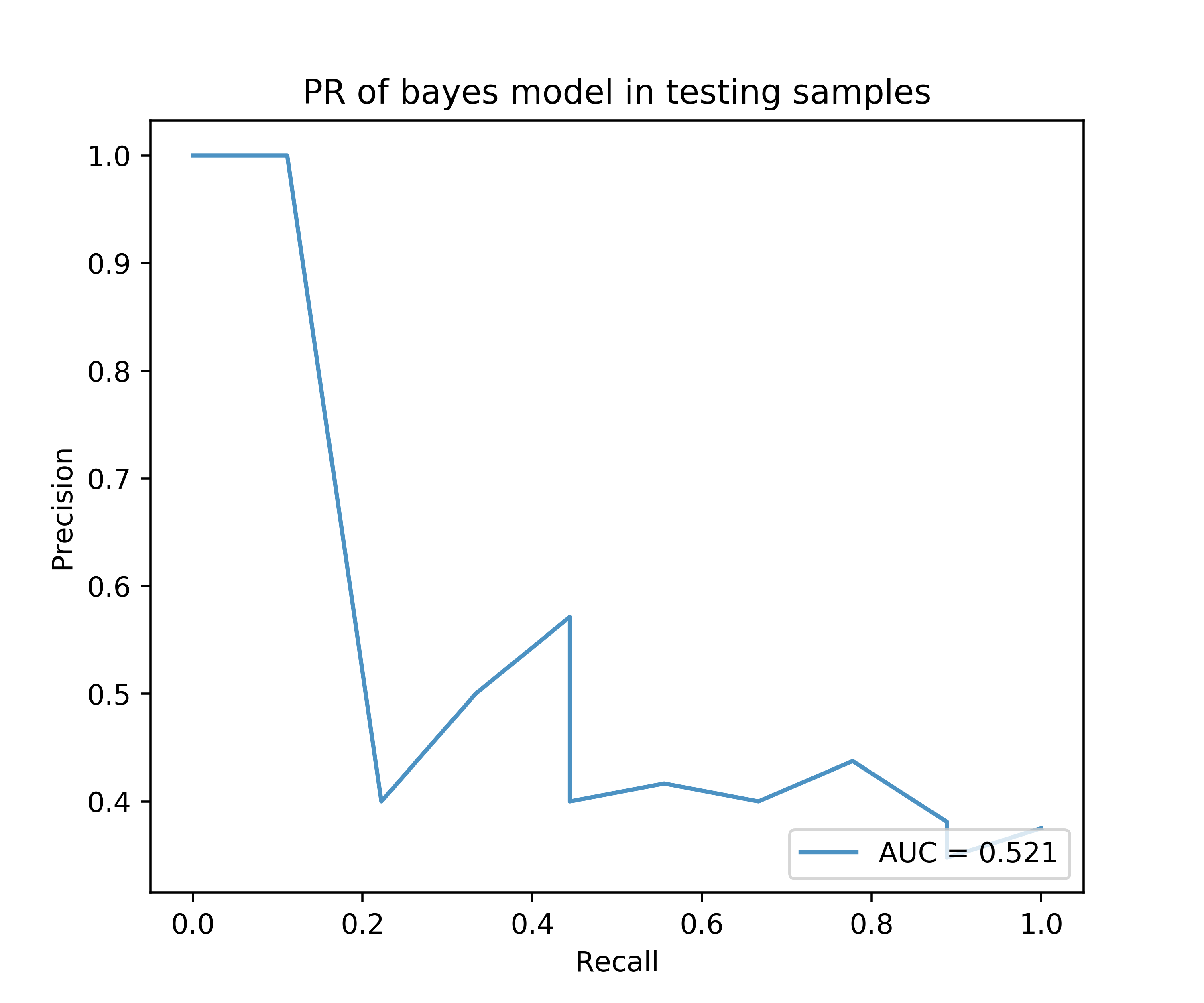


**Radiomics analysis process of logistic regression.**

Summary Report

# The summary report recognized the best model from all models your training, and summarized its training process.

As the result shown, the best model was Logistic, its training process including: ['select a path', 'set a seed', 'seperate a data', 'input data', 'standardize data', 'select feature', 'select feature', 'select feature', 'machine_learning']. Detailed information is shown below:

# 1. Data: C:/Users/212768837/Desktop/zhuanyi_86ROI/new/result2.csv

# 2. Random seed: 68

# 3. Seperative rate: 0.7

Seperated report:

|  | Sum | Pos | Neg |
| --- | --- | --- | --- |
| data | 86 | 29 | 57 |
| train | 60 | 20 | 40 |
| test | 26 | 9 | 17 |

# 4. Input data

The method for filling the missing data: Median

The method for dealing with outliers: Median

# 5. The method for standardizing the data: Standardization

# 6. The method for selecting features: Variance

parameters setted: {'threshold': 1.0}
num of remained features: 419
remained features:
[['original_shape_Elongation']
 ['original_shape_Flatness']
 ['original_shape_LeastAxisLength']
 ['original_shape_MajorAxisLength']
 ['original_shape_Maximum2DDiameterColumn']
 ['original_shape_Maximum3DDiameter']
 ['original_shape_MinorAxisLength']
 ['original_shape_SurfaceVolumeRatio']
 ['original_shape_VoxelVolume']
 ['original_firstorder_90Percentile']
 ['original_firstorder_Entropy']
 ['original_firstorder_Kurtosis']
 ['original_firstorder_Maximum']
 ['original_firstorder_Minimum']
 ['original_firstorder_Uniformity']
 ['original_glcm_Autocorrelation']
 ['original_glcm_ClusterProminence']
 ['original_glcm_ClusterShade']
 ['original_glcm_ClusterTendency']
 ['original_glcm_DifferenceEntropy']
 ['original_glcm_DifferenceVariance']
 ['original_glcm_Idm']
 ['original_glcm_Idmn']
 ['original_glcm_Idn']
 ['original_glcm_Imc1']
 ['original_glcm_InverseVariance']
 ['original_glcm_MCC']
 ['original_gldm_DependenceEntropy']
 ['original_gldm_DependenceVariance']
 ['original_gldm_GrayLevelNonUniformity']
 ['original_gldm_HighGrayLevelEmphasis']
 ['original_gldm_LargeDependenceEmphasis']
 ['original_gldm_LargeDependenceLowGrayLevelEmphasis']
 ['original_gldm_LowGrayLevelEmphasis']
 ['original_gldm_SmallDependenceEmphasis']
 ['original_gldm_SmallDependenceHighGrayLevelEmphasis']
 ['original_glrlm_GrayLevelVariance']
 ['original_glrlm_LongRunHighGrayLevelEmphasis']
 ['original_glrlm_LongRunLowGrayLevelEmphasis']
 ['original_glrlm_LowGrayLevelRunEmphasis']
 ['original_glrlm_RunEntropy']
 ['original_glrlm_ShortRunEmphasis']
 ['original_glszm_GrayLevelNonUniformityNormalized']
 ['original_glszm_GrayLevelVariance']
 ['original_glszm_HighGrayLevelZoneEmphasis']
 ['original_glszm_SizeZoneNonUniformity']
 ['original_glszm_SizeZoneNonUniformityNormalized']
 ['original_glszm_SmallAreaEmphasis']
 ['original_glszm_SmallAreaLowGrayLevelEmphasis']
 ['original_glszm_ZonePercentage']
 ['original_ngtdm_Busyness']
 ['original_ngtdm_Coarseness']
 ['original_ngtdm_Complexity']
 ['original_ngtdm_Contrast']
 ['original_ngtdm_Strength']
 ['wavelet-LLH_firstorder_10Percentile']
 ['wavelet-LLH_firstorder_90Percentile']
 ['wavelet-LLH_firstorder_Energy']
 ['wavelet-LLH_firstorder_Entropy']
 ['wavelet-LLH_firstorder_InterquartileRange']
 ['wavelet-LLH_firstorder_Kurtosis']
 ['wavelet-LLH_firstorder_Median']
 ['wavelet-LLH_firstorder_Skewness']
 ['wavelet-LLH_firstorder_TotalEnergy']
 ['wavelet-LLH_glcm_ClusterProminence']
 ['wavelet-LLH_glcm_Contrast']
 ['wavelet-LLH_glcm_Correlation']
 ['wavelet-LLH_glcm_DifferenceAverage']
 ['wavelet-LLH_glcm_DifferenceVariance']
 ['wavelet-LLH_glcm_Id']
 ['wavelet-LLH_glcm_Idm']
 ['wavelet-LLH_glcm_Imc1']
 ['wavelet-LLH_glcm_Imc2']
 ['wavelet-LLH_glcm_JointEnergy']
 ['wavelet-LLH_glcm_MaximumProbability']
 ['wavelet-LLH_glcm_SumEntropy']
 ['wavelet-LLH_glcm_SumSquares']
 ['wavelet-LLH_gldm_DependenceEntropy']
 ['wavelet-LLH_gldm_DependenceNonUniformityNormalized']
 ['wavelet-LLH_gldm_DependenceVariance']
 ['wavelet-LLH_gldm_LargeDependenceEmphasis']
 ['wavelet-LLH_gldm_SmallDependenceHighGrayLevelEmphasis']
 ['wavelet-LLH_glrlm_GrayLevelNonUniformityNormalized']
 ['wavelet-LLH_glrlm_GrayLevelVariance']
 ['wavelet-LLH_glrlm_LowGrayLevelRunEmphasis']
 ['wavelet-LLH_glrlm_RunEntropy']
 ['wavelet-LLH_glrlm_RunLengthNonUniformity']
 ['wavelet-LLH_glrlm_RunLengthNonUniformityNormalized']
 ['wavelet-LLH_glrlm_RunPercentage']
 ['wavelet-LLH_glrlm_RunVariance']
 ['wavelet-LLH_glrlm_ShortRunEmphasis']
 ['wavelet-LLH_glszm_GrayLevelNonUniformity']
 ['wavelet-LLH_glszm_LargeAreaEmphasis']
 ['wavelet-LLH_glszm_LargeAreaHighGrayLevelEmphasis']
 ['wavelet-LLH_glszm_LowGrayLevelZoneEmphasis']
 ['wavelet-LLH_glszm_SizeZoneNonUniformityNormalized']
 ['wavelet-LLH_glszm_SmallAreaHighGrayLevelEmphasis']
 ['wavelet-LLH_glszm_ZoneVariance']
 ['wavelet-LLH_ngtdm_Contrast']
 ['wavelet-LHL_firstorder_10Percentile']
 ['wavelet-LHL_firstorder_Energy']
 ['wavelet-LHL_firstorder_Kurtosis']
 ['wavelet-LHL_firstorder_Maximum']
 ['wavelet-LHL_firstorder_Median']
 ['wavelet-LHL_firstorder_RobustMeanAbsoluteDeviation']
 ['wavelet-LHL_firstorder_RootMeanSquared']
 ['wavelet-LHL_firstorder_TotalEnergy']
 ['wavelet-LHL_glcm_Autocorrelation']
 ['wavelet-LHL_glcm_ClusterProminence']
 ['wavelet-LHL_glcm_Contrast']
 ['wavelet-LHL_glcm_DifferenceAverage']
 ['wavelet-LHL_glcm_DifferenceEntropy']
 ['wavelet-LHL_glcm_DifferenceVariance']
 ['wavelet-LHL_glcm_Idm']
 ['wavelet-LHL_glcm_Idmn']
 ['wavelet-LHL_glcm_Imc2']
 ['wavelet-LHL_glcm_MCC']
 ['wavelet-LHL_glcm_MaximumProbability']
 ['wavelet-LHL_glcm_SumEntropy']
 ['wavelet-LHL_glcm_SumSquares']
 ['wavelet-LHL_gldm_DependenceEntropy']
 ['wavelet-LHL_gldm_DependenceNonUniformity']
 ['wavelet-LHL_gldm_DependenceNonUniformityNormalized']
 ['wavelet-LHL_gldm_GrayLevelNonUniformity']
 ['wavelet-LHL_gldm_GrayLevelVariance']
 ['wavelet-LHL_gldm_LargeDependenceEmphasis']
 ['wavelet-LHL_gldm_LargeDependenceHighGrayLevelEmphasis']
 ['wavelet-LHL_gldm_LargeDependenceLowGrayLevelEmphasis']
 ['wavelet-LHL_gldm_LowGrayLevelEmphasis']
 ['wavelet-LHL_glrlm_GrayLevelNonUniformityNormalized']
 ['wavelet-LHL_glrlm_LongRunEmphasis']
 ['wavelet-LHL_glrlm_LowGrayLevelRunEmphasis']
 ['wavelet-LHL_glrlm_RunEntropy']
 ['wavelet-LHL_glrlm_RunLengthNonUniformity']
 ['wavelet-LHL_glrlm_RunLengthNonUniformityNormalized']
 ['wavelet-LHL_glrlm_RunVariance']
 ['wavelet-LHL_glrlm_ShortRunEmphasis']
 ['wavelet-LHL_glrlm_ShortRunLowGrayLevelEmphasis']
 ['wavelet-LHL_glszm_GrayLevelVariance']
 ['wavelet-LHL_glszm_HighGrayLevelZoneEmphasis']
 ['wavelet-LHL_glszm_LargeAreaEmphasis']
 ['wavelet-LHL_glszm_LargeAreaLowGrayLevelEmphasis']
 ['wavelet-LHL_glszm_SizeZoneNonUniformity']
 ['wavelet-LHL_glszm_SmallAreaHighGrayLevelEmphasis']
 ['wavelet-LHL_glszm_SmallAreaLowGrayLevelEmphasis']
 ['wavelet-LHL_glszm_ZoneEntropy']
 ['wavelet-LHL_glszm_ZonePercentage']
 ['wavelet-LHL_glszm_ZoneVariance']
 ['wavelet-LHL_ngtdm_Busyness']
 ['wavelet-LHL_ngtdm_Coarseness']
 ['wavelet-LHL_ngtdm_Complexity']
 ['wavelet-LHL_ngtdm_Contrast']
 ['wavelet-LHL_ngtdm_Strength']
 ['wavelet-LHH_firstorder_90Percentile']
 ['wavelet-LHH_firstorder_Energy']
 ['wavelet-LHH_firstorder_InterquartileRange']
 ['wavelet-LHH_firstorder_Kurtosis']
 ['wavelet-LHH_firstorder_Maximum']
 ['wavelet-LHH_firstorder_MeanAbsoluteDeviation']
 ['wavelet-LHH_firstorder_Mean']
 ['wavelet-LHH_firstorder_Range']
 ['wavelet-LHH_firstorder_RobustMeanAbsoluteDeviation']
 ['wavelet-LHH_firstorder_TotalEnergy']
 ['wavelet-LHH_firstorder_Uniformity']
 ['wavelet-LHH_firstorder_Variance']
 ['wavelet-LHH_glcm_ClusterShade']
 ['wavelet-LHH_glcm_Contrast']
 ['wavelet-LHH_glcm_DifferenceVariance']
 ['wavelet-LHH_glcm_Idmn']
 ['wavelet-LHH_glcm_Idn']
 ['wavelet-LHH_glcm_JointAverage']
 ['wavelet-LHH_glcm_JointEnergy']
 ['wavelet-LHH_glcm_JointEntropy']
 ['wavelet-LHH_glcm_MCC']
 ['wavelet-LHH_glcm_MaximumProbability']
 ['wavelet-LHH_glcm_SumAverage']
 ['wavelet-LHH_glcm_SumEntropy']
 ['wavelet-LHH_glcm_SumSquares']
 ['wavelet-LHH_gldm_DependenceNonUniformity']
 ['wavelet-LHH_gldm_DependenceVariance']
 ['wavelet-LHH_gldm_GrayLevelVariance']
 ['wavelet-LHH_gldm_HighGrayLevelEmphasis']
 ['wavelet-LHH_gldm_LargeDependenceEmphasis']
 ['wavelet-LHH_gldm_LargeDependenceHighGrayLevelEmphasis']
 ['wavelet-LHH_gldm_LargeDependenceLowGrayLevelEmphasis']
 ['wavelet-LHH_gldm_LowGrayLevelEmphasis']
 ['wavelet-LHH_gldm_SmallDependenceHighGrayLevelEmphasis']
 ['wavelet-LHH_glrlm_GrayLevelNonUniformity']
 ['wavelet-LHH_glrlm_GrayLevelVariance']
 ['wavelet-LHH_glrlm_HighGrayLevelRunEmphasis']
 ['wavelet-LHH_glrlm_LongRunEmphasis']
 ['wavelet-LHH_glrlm_LongRunHighGrayLevelEmphasis']
 ['wavelet-LHH_glrlm_LowGrayLevelRunEmphasis']
 ['wavelet-LHH_glrlm_RunEntropy']
 ['wavelet-LHH_glrlm_RunLengthNonUniformityNormalized']
 ['wavelet-LHH_glrlm_RunVariance']
 ['wavelet-LHH_glrlm_ShortRunEmphasis']
 ['wavelet-LHH_glrlm_ShortRunLowGrayLevelEmphasis']
 ['wavelet-LHH_glszm_GrayLevelVariance']
 ['wavelet-LHH_glszm_HighGrayLevelZoneEmphasis']
 ['wavelet-LHH_glszm_LargeAreaHighGrayLevelEmphasis']
 ['wavelet-LHH_glszm_LargeAreaLowGrayLevelEmphasis']
 ['wavelet-LHH_glszm_SizeZoneNonUniformity']
 ['wavelet-LHH_glszm_SizeZoneNonUniformityNormalized']
 ['wavelet-LHH_glszm_SmallAreaEmphasis']
 ['wavelet-LHH_glszm_SmallAreaHighGrayLevelEmphasis']
 ['wavelet-LHH_glszm_ZonePercentage']
 ['wavelet-HLL_firstorder_InterquartileRange']
 ['wavelet-HLL_firstorder_Kurtosis']
 ['wavelet-HLL_firstorder_Maximum']
 ['wavelet-HLL_firstorder_MeanAbsoluteDeviation']
 ['wavelet-HLL_firstorder_Mean']
 ['wavelet-HLL_firstorder_Median']
 ['wavelet-HLL_firstorder_Range']
 ['wavelet-HLL_firstorder_Skewness']
 ['wavelet-HLL_firstorder_Uniformity']
 ['wavelet-HLL_firstorder_Variance']
 ['wavelet-HLL_glcm_Autocorrelation']
 ['wavelet-HLL_glcm_ClusterProminence']
 ['wavelet-HLL_glcm_Contrast']
 ['wavelet-HLL_glcm_Correlation']
 ['wavelet-HLL_glcm_DifferenceEntropy']
 ['wavelet-HLL_glcm_DifferenceVariance']
 ['wavelet-HLL_glcm_Id']
 ['wavelet-HLL_glcm_Idmn']
 ['wavelet-HLL_glcm_Imc2']
 ['wavelet-HLL_glcm_InverseVariance']
 ['wavelet-HLL_glcm_SumEntropy']
 ['wavelet-HLL_gldm_DependenceEntropy']
 ['wavelet-HLL_gldm_DependenceNonUniformity']
 ['wavelet-HLL_gldm_GrayLevelNonUniformity']
 ['wavelet-HLL_gldm_HighGrayLevelEmphasis']
 ['wavelet-HLL_gldm_LargeDependenceLowGrayLevelEmphasis']
 ['wavelet-HLL_gldm_LowGrayLevelEmphasis']
 ['wavelet-HLL_gldm_SmallDependenceEmphasis']
 ['wavelet-HLL_gldm_SmallDependenceHighGrayLevelEmphasis']
 ['wavelet-HLL_glrlm_GrayLevelNonUniformity']
 ['wavelet-HLL_glrlm_GrayLevelVariance']
 ['wavelet-HLL_glrlm_HighGrayLevelRunEmphasis']
 ['wavelet-HLL_glrlm_LongRunEmphasis']
 ['wavelet-HLL_glrlm_LongRunHighGrayLevelEmphasis']
 ['wavelet-HLL_glrlm_RunLengthNonUniformity']
 ['wavelet-HLL_glrlm_RunLengthNonUniformityNormalized']
 ['wavelet-HLL_glrlm_ShortRunLowGrayLevelEmphasis']
 ['wavelet-HLL_glszm_GrayLevelNonUniformityNormalized']
 ['wavelet-HLL_glszm_GrayLevelVariance']
 ['wavelet-HLL_glszm_HighGrayLevelZoneEmphasis']
 ['wavelet-HLL_glszm_LargeAreaHighGrayLevelEmphasis']
 ['wavelet-HLL_glszm_SizeZoneNonUniformity']
 ['wavelet-HLL_ngtdm_Complexity']
 ['wavelet-HLL_ngtdm_Strength']
 ['wavelet-HLH_firstorder_10Percentile']
 ['wavelet-HLH_firstorder_Entropy']
 ['wavelet-HLH_firstorder_Mean']
 ['wavelet-HLH_firstorder_Median']
 ['wavelet-HLH_firstorder_Minimum']
 ['wavelet-HLH_firstorder_Range']
 ['wavelet-HLH_firstorder_RootMeanSquared']
 ['wavelet-HLH_firstorder_Skewness']
 ['wavelet-HLH_firstorder_Uniformity']
 ['wavelet-HLH_glcm_ClusterProminence']
 ['wavelet-HLH_glcm_ClusterTendency']
 ['wavelet-HLH_glcm_Contrast']
 ['wavelet-HLH_glcm_DifferenceAverage']
 ['wavelet-HLH_glcm_DifferenceEntropy']
 ['wavelet-HLH_glcm_Idn']
 ['wavelet-HLH_glcm_JointAverage']
 ['wavelet-HLH_glcm_MCC']
 ['wavelet-HLH_glcm_MaximumProbability']
 ['wavelet-HLH_glcm_SumAverage']
 ['wavelet-HLH_glcm_SumSquares']
 ['wavelet-HLH_gldm_DependenceEntropy']
 ['wavelet-HLH_gldm_DependenceVariance']
 ['wavelet-HLH_gldm_GrayLevelNonUniformity']
 ['wavelet-HLH_gldm_GrayLevelVariance']
 ['wavelet-HLH_gldm_HighGrayLevelEmphasis']
 ['wavelet-HLH_gldm_LargeDependenceEmphasis']
 ['wavelet-HLH_gldm_LargeDependenceHighGrayLevelEmphasis']
 ['wavelet-HLH_glrlm_GrayLevelNonUniformity']
 ['wavelet-HLH_glrlm_LongRunEmphasis']
 ['wavelet-HLH_glrlm_LongRunLowGrayLevelEmphasis']
 ['wavelet-HLH_glrlm_RunLengthNonUniformityNormalized']
 ['wavelet-HLH_glrlm_ShortRunEmphasis']
 ['wavelet-HLH_glrlm_ShortRunHighGrayLevelEmphasis']
 ['wavelet-HLH_glszm_GrayLevelNonUniformityNormalized']
 ['wavelet-HLH_glszm_GrayLevelVariance']
 ['wavelet-HLH_glszm_HighGrayLevelZoneEmphasis']
 ['wavelet-HLH_glszm_LargeAreaEmphasis']
 ['wavelet-HLH_glszm_LargeAreaLowGrayLevelEmphasis']
 ['wavelet-HLH_glszm_SmallAreaEmphasis']
 ['wavelet-HLH_glszm_ZoneVariance']
 ['wavelet-HLH_ngtdm_Coarseness']
 ['wavelet-HHL_firstorder_90Percentile']
 ['wavelet-HHL_firstorder_Maximum']
 ['wavelet-HHL_firstorder_Mean']
 ['wavelet-HHL_firstorder_Median']
 ['wavelet-HHL_firstorder_Range']
 ['wavelet-HHL_firstorder_RootMeanSquared']
 ['wavelet-HHL_firstorder_Variance']
 ['wavelet-HHL_glcm_Autocorrelation']
 ['wavelet-HHL_glcm_DifferenceAverage']
 ['wavelet-HHL_glcm_Idm']
 ['wavelet-HHL_glcm_Idmn']
 ['wavelet-HHL_glcm_JointAverage']
 ['wavelet-HHL_glcm_JointEntropy']
 ['wavelet-HHL_glcm_MCC']
 ['wavelet-HHL_glcm_SumAverage']
 ['wavelet-HHL_gldm_DependenceEntropy']
 ['wavelet-HHL_gldm_DependenceNonUniformity']
 ['wavelet-HHL_gldm_DependenceNonUniformityNormalized']
 ['wavelet-HHL_gldm_GrayLevelVariance']
 ['wavelet-HHL_gldm_HighGrayLevelEmphasis']
 ['wavelet-HHL_gldm_SmallDependenceEmphasis']
 ['wavelet-HHL_gldm_SmallDependenceLowGrayLevelEmphasis']
 ['wavelet-HHL_glrlm_GrayLevelNonUniformity']
 ['wavelet-HHL_glrlm_GrayLevelNonUniformityNormalized']
 ['wavelet-HHL_glrlm_HighGrayLevelRunEmphasis']
 ['wavelet-HHL_glrlm_LongRunEmphasis']
 ['wavelet-HHL_glrlm_LongRunHighGrayLevelEmphasis']
 ['wavelet-HHL_glrlm_LongRunLowGrayLevelEmphasis']
 ['wavelet-HHL_glrlm_RunLengthNonUniformity']
 ['wavelet-HHL_glrlm_RunPercentage']
 ['wavelet-HHL_glrlm_ShortRunEmphasis']
 ['wavelet-HHL_glszm_GrayLevelNonUniformity']
 ['wavelet-HHL_glszm_GrayLevelNonUniformityNormalized']
 ['wavelet-HHL_glszm_GrayLevelVariance']
 ['wavelet-HHL_glszm_HighGrayLevelZoneEmphasis']
 ['wavelet-HHL_glszm_LargeAreaEmphasis']
 ['wavelet-HHL_glszm_LargeAreaLowGrayLevelEmphasis']
 ['wavelet-HHL_glszm_LowGrayLevelZoneEmphasis']
 ['wavelet-HHL_glszm_SmallAreaEmphasis']
 ['wavelet-HHL_glszm_SmallAreaHighGrayLevelEmphasis']
 ['wavelet-HHL_glszm_SmallAreaLowGrayLevelEmphasis']
 ['wavelet-HHL_glszm_ZonePercentage']
 ['wavelet-HHL_ngtdm_Busyness']
 ['wavelet-HHL_ngtdm_Complexity']
 ['wavelet-HHL_ngtdm_Strength']
 ['wavelet-HHH_firstorder_10Percentile']
 ['wavelet-HHH_firstorder_Energy']
 ['wavelet-HHH_firstorder_Entropy']
 ['wavelet-HHH_firstorder_Kurtosis']
 ['wavelet-HHH_firstorder_MeanAbsoluteDeviation']
 ['wavelet-HHH_firstorder_Mean']
 ['wavelet-HHH_firstorder_Median']
 ['wavelet-HHH_firstorder_Minimum']
 ['wavelet-HHH_firstorder_RootMeanSquared']
 ['wavelet-HHH_firstorder_TotalEnergy']
 ['wavelet-HHH_firstorder_Variance']
 ['wavelet-HHH_glcm_ClusterTendency']
 ['wavelet-HHH_glcm_Contrast']
 ['wavelet-HHH_glcm_DifferenceEntropy']
 ['wavelet-HHH_glcm_Id']
 ['wavelet-HHH_glcm_Idmn']
 ['wavelet-HHH_glcm_Idn']
 ['wavelet-HHH_glcm_JointAverage']
 ['wavelet-HHH_glcm_JointEnergy']
 ['wavelet-HHH_glcm_JointEntropy']
 ['wavelet-HHH_glcm_SumAverage']
 ['wavelet-HHH_gldm_DependenceVariance']
 ['wavelet-HHH_gldm_GrayLevelNonUniformity']
 ['wavelet-HHH_gldm_GrayLevelVariance']
 ['wavelet-HHH_gldm_LargeDependenceHighGrayLevelEmphasis']
 ['wavelet-HHH_gldm_LargeDependenceLowGrayLevelEmphasis']
 ['wavelet-HHH_gldm_SmallDependenceEmphasis']
 ['wavelet-HHH_gldm_SmallDependenceHighGrayLevelEmphasis']
 ['wavelet-HHH_gldm_SmallDependenceLowGrayLevelEmphasis']
 ['wavelet-HHH_glrlm_GrayLevelNonUniformity']
 ['wavelet-HHH_glrlm_GrayLevelNonUniformityNormalized']
 ['wavelet-HHH_glrlm_LongRunLowGrayLevelEmphasis']
 ['wavelet-HHH_glrlm_RunLengthNonUniformity']
 ['wavelet-HHH_glrlm_RunPercentage']
 ['wavelet-HHH_glrlm_ShortRunHighGrayLevelEmphasis']
 ['wavelet-HHH_glszm_LargeAreaHighGrayLevelEmphasis']
 ['wavelet-HHH_glszm_SizeZoneNonUniformityNormalized']
 ['wavelet-HHH_glszm_SmallAreaEmphasis']
 ['wavelet-HHH_glszm_ZoneVariance']
 ['wavelet-HHH_ngtdm_Coarseness']
 ['wavelet-HHH_ngtdm_Contrast']
 ['wavelet-LLL_firstorder_Entropy']
 ['wavelet-LLL_firstorder_Kurtosis']
 ['wavelet-LLL_firstorder_Maximum']
 ['wavelet-LLL_firstorder_RobustMeanAbsoluteDeviation']
 ['wavelet-LLL_firstorder_Uniformity']
 ['wavelet-LLL_glcm_ClusterShade']
 ['wavelet-LLL_glcm_Idm']
 ['wavelet-LLL_glcm_Idn']
 ['wavelet-LLL_glcm_Imc1']
 ['wavelet-LLL_glcm_Imc2']
 ['wavelet-LLL_glcm_InverseVariance']
 ['wavelet-LLL_glcm_JointAverage']
 ['wavelet-LLL_glcm_MaximumProbability']
 ['wavelet-LLL_glcm_SumAverage']
 ['wavelet-LLL_glcm_SumEntropy']
 ['wavelet-LLL_gldm_DependenceEntropy']
 ['wavelet-LLL_gldm_DependenceNonUniformity']
 ['wavelet-LLL_gldm_GrayLevelVariance']
 ['wavelet-LLL_gldm_LargeDependenceEmphasis']
 ['wavelet-LLL_gldm_LargeDependenceHighGrayLevelEmphasis']
 ['wavelet-LLL_gldm_LargeDependenceLowGrayLevelEmphasis']
 ['wavelet-LLL_gldm_LowGrayLevelEmphasis']
 ['wavelet-LLL_glrlm_GrayLevelVariance']
 ['wavelet-LLL_glrlm_HighGrayLevelRunEmphasis']
 ['wavelet-LLL_glrlm_LongRunEmphasis']
 ['wavelet-LLL_glrlm_LongRunHighGrayLevelEmphasis']
 ['wavelet-LLL_glrlm_RunLengthNonUniformity']
 ['wavelet-LLL_glrlm_RunPercentage']
 ['wavelet-LLL_glrlm_ShortRunEmphasis']
 ['wavelet-LLL_glrlm_ShortRunLowGrayLevelEmphasis']
 ['wavelet-LLL_glszm_GrayLevelNonUniformityNormalized']
 ['wavelet-LLL_glszm_GrayLevelVariance']
 ['wavelet-LLL_glszm_HighGrayLevelZoneEmphasis']
 ['wavelet-LLL_glszm_LargeAreaLowGrayLevelEmphasis']
 ['wavelet-LLL_glszm_SizeZoneNonUniformity']
 ['wavelet-LLL_glszm_ZonePercentage']
 ['wavelet-LLL_ngtdm_Busyness']
 ['wavelet-LLL_ngtdm_Coarseness']
 ['wavelet-LLL_ngtdm_Complexity']
 ['wavelet-LLL_ngtdm_Contrast']
 ['wavelet-LLL_ngtdm_Strength']]

Heatmap of the model in the training samples:


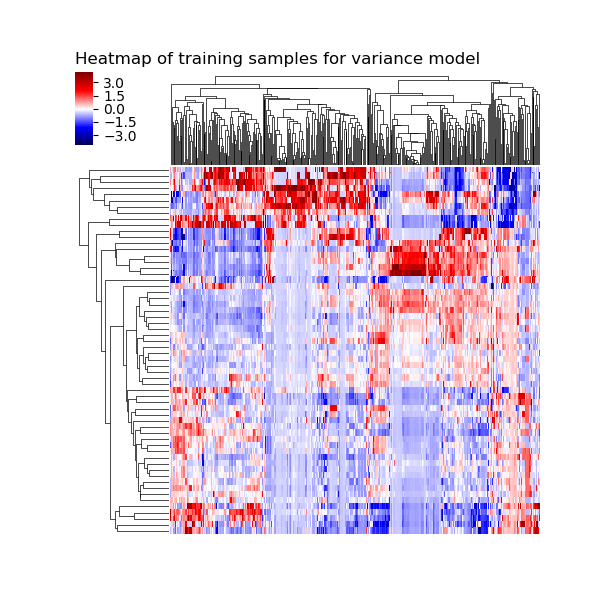


Heatmap of the model in the testing samples:


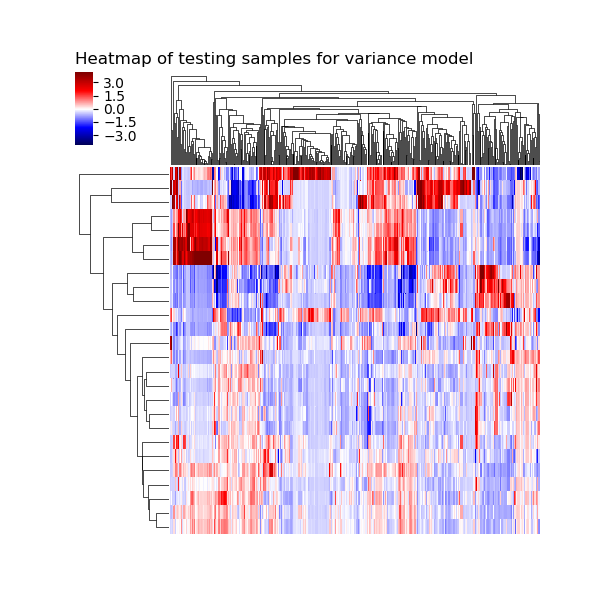


Correlation coefficient figure of the training samples

Correlation coefficient figure of the testing samples

# 7. The method for selecting features: Correlation_xx

parameters setted: {'cutoff': 0.7}
num of remained features: 93
remained features:
[['original_shape_Flatness']
 ['original_firstorder_Maximum']
 ['original_firstorder_Minimum']
 ['original_glcm_ClusterShade']
 ['original_glcm_DifferenceEntropy']
 ['original_glcm_Imc1']
 ['original_glcm_InverseVariance']
 ['original_gldm_LargeDependenceLowGrayLevelEmphasis']
 ['original_glszm_SmallAreaEmphasis']
 ['wavelet-LLH_firstorder_90Percentile']
 ['wavelet-LLH_firstorder_Kurtosis']
 ['wavelet-LLH_firstorder_Median']
 ['wavelet-LLH_firstorder_Skewness']
 ['wavelet-LLH_firstorder_TotalEnergy']
 ['wavelet-LLH_glcm_Correlation']
 ['wavelet-LLH_glcm_Imc1']
 ['wavelet-LLH_gldm_DependenceNonUniformityNormalized']
 ['wavelet-LLH_gldm_SmallDependenceHighGrayLevelEmphasis']
 ['wavelet-LLH_glrlm_LowGrayLevelRunEmphasis']
 ['wavelet-LLH_glszm_LargeAreaHighGrayLevelEmphasis']
 ['wavelet-LLH_glszm_LowGrayLevelZoneEmphasis']
 ['wavelet-LLH_glszm_SizeZoneNonUniformityNormalized']
 ['wavelet-LLH_glszm_SmallAreaHighGrayLevelEmphasis']
 ['wavelet-LLH_ngtdm_Contrast']
 ['wavelet-LHL_firstorder_Kurtosis']
 ['wavelet-LHL_glcm_Contrast']
 ['wavelet-LHL_glcm_MCC']
 ['wavelet-LHL_gldm_LargeDependenceLowGrayLevelEmphasis']
 ['wavelet-LHL_glrlm_LowGrayLevelRunEmphasis']
 ['wavelet-LHL_glrlm_RunEntropy']
 ['wavelet-LHL_glszm_LargeAreaLowGrayLevelEmphasis']
 ['wavelet-LHL_glszm_SmallAreaLowGrayLevelEmphasis']
 ['wavelet-LHL_ngtdm_Complexity']
 ['wavelet-LHL_ngtdm_Strength']
 ['wavelet-LHH_firstorder_Mean']
 ['wavelet-LHH_glcm_ClusterShade']
 ['wavelet-LHH_gldm_DependenceVariance']
 ['wavelet-LHH_gldm_LargeDependenceLowGrayLevelEmphasis']
 ['wavelet-LHH_glszm_SizeZoneNonUniformity']
 ['wavelet-LHH_glszm_SizeZoneNonUniformityNormalized']
 ['wavelet-LHH_glszm_SmallAreaEmphasis']
 ['wavelet-HLL_firstorder_Kurtosis']
 ['wavelet-HLL_firstorder_Skewness']
 ['wavelet-HLL_glcm_ClusterProminence']
 ['wavelet-HLL_glcm_Correlation']
 ['wavelet-HLL_glcm_DifferenceVariance']
 ['wavelet-HLL_glcm_Imc2']
 ['wavelet-HLL_glcm_InverseVariance']
 ['wavelet-HLL_gldm_DependenceEntropy']
 ['wavelet-HLL_glrlm_LongRunHighGrayLevelEmphasis']
 ['wavelet-HLL_glszm_GrayLevelNonUniformityNormalized']
 ['wavelet-HLL_glszm_LargeAreaHighGrayLevelEmphasis']
 ['wavelet-HLL_ngtdm_Complexity']
 ['wavelet-HLL_ngtdm_Strength']
 ['wavelet-HLH_firstorder_Median']
 ['wavelet-HLH_firstorder_Skewness']
 ['wavelet-HLH_glcm_MaximumProbability']
 ['wavelet-HLH_gldm_DependenceEntropy']
 ['wavelet-HLH_gldm_DependenceVariance']
 ['wavelet-HLH_glrlm_LongRunLowGrayLevelEmphasis']
 ['wavelet-HLH_glszm_SmallAreaEmphasis']
 ['wavelet-HHL_firstorder_Mean']
 ['wavelet-HHL_firstorder_Median']
 ['wavelet-HHL_firstorder_Variance']
 ['wavelet-HHL_glcm_Autocorrelation']
 ['wavelet-HHL_glcm_MCC']
 ['wavelet-HHL_gldm_DependenceEntropy']
 ['wavelet-HHL_gldm_DependenceNonUniformityNormalized']
 ['wavelet-HHL_glrlm_LongRunHighGrayLevelEmphasis']
 ['wavelet-HHL_glrlm_LongRunLowGrayLevelEmphasis']
 ['wavelet-HHL_glszm_GrayLevelNonUniformityNormalized']
 ['wavelet-HHL_glszm_LargeAreaLowGrayLevelEmphasis']
 ['wavelet-HHL_glszm_SmallAreaEmphasis']
 ['wavelet-HHL_glszm_SmallAreaLowGrayLevelEmphasis']
 ['wavelet-HHH_firstorder_Kurtosis']
 ['wavelet-HHH_firstorder_Mean']
 ['wavelet-HHH_firstorder_Median']
 ['wavelet-HHH_glcm_Contrast']
 ['wavelet-HHH_glcm_Id']
 ['wavelet-HHH_gldm_DependenceVariance']
 ['wavelet-HHH_gldm_SmallDependenceEmphasis']
 ['wavelet-HHH_gldm_SmallDependenceHighGrayLevelEmphasis']
 ['wavelet-HHH_gldm_SmallDependenceLowGrayLevelEmphasis']
 ['wavelet-HHH_glszm_SizeZoneNonUniformityNormalized']
 ['wavelet-HHH_glszm_SmallAreaEmphasis']
 ['wavelet-HHH_glszm_ZoneVariance']
 ['wavelet-LLL_firstorder_Entropy']
 ['wavelet-LLL_firstorder_Kurtosis']
 ['wavelet-LLL_gldm_LargeDependenceLowGrayLevelEmphasis']
 ['wavelet-LLL_glszm_LargeAreaLowGrayLevelEmphasis']
 ['wavelet-LLL_ngtdm_Busyness']
 ['wavelet-LLL_ngtdm_Complexity']
 ['wavelet-LLL_ngtdm_Contrast']]

Heatmap of the model in the training samples:


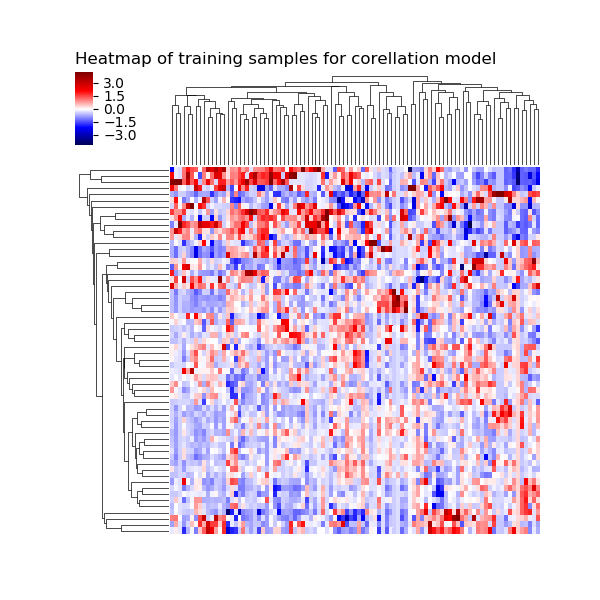


Heatmap of the model in the testing samples:


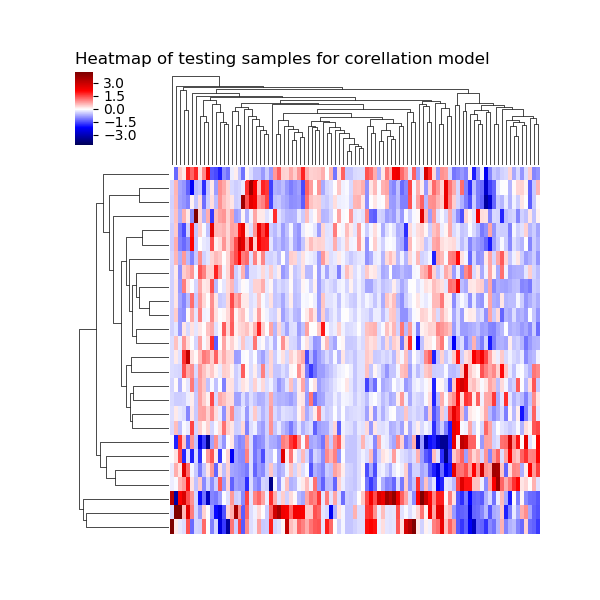


Correlation coefficient figure of the training samples

Correlation coefficient figure of the testing samples

# 8. The method for selecting features: MultiVariate_Logistic

parameters setted: {'P value for threshold in': 0.05, 'P value for threshold out': 0.1}
num of remained features: 4
remained features:
[['wavelet-HHH_glszm_SizeZoneNonUniformityNormalized']
 ['wavelet-LLH_firstorder_TotalEnergy']
 ['wavelet-HHL_glszm_SmallAreaLowGrayLevelEmphasis']
 ['wavelet-HLH_firstorder_Skewness']]

Statistical analysis of logistic multivariate analysis:

| feature | OR | 0.025 | 0.975 | P_value |
| --- | --- | --- | --- | --- |
| const | 0.267 | 0.108 | 0.66 | nan |
| wavelet-HHH_glszm_SizeZoneNonUniformityNormalized | 5.766 | 1.672 | 19.889 | 0.006 |
| wavelet-LLH_firstorder_TotalEnergy | 4.592 | 1.55 | 13.601 | 0.006 |
| wavelet-HHL_glszm_SmallAreaLowGrayLevelEmphasis | 3.147 | 1.319 | 7.512 | 0.01 |
| wavelet-HLH_firstorder_Skewness | 2.703 | 1.076 | 6.792 | 0.034 |

Heatmap of the model in the training samples:


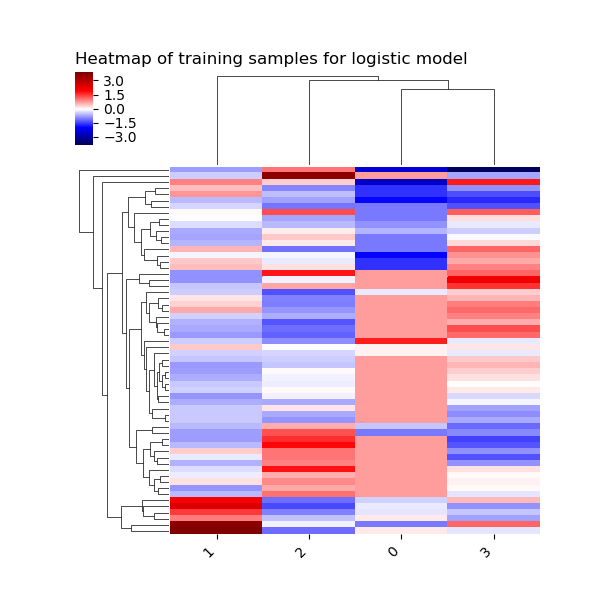


Heatmap of the model in the testing samples:


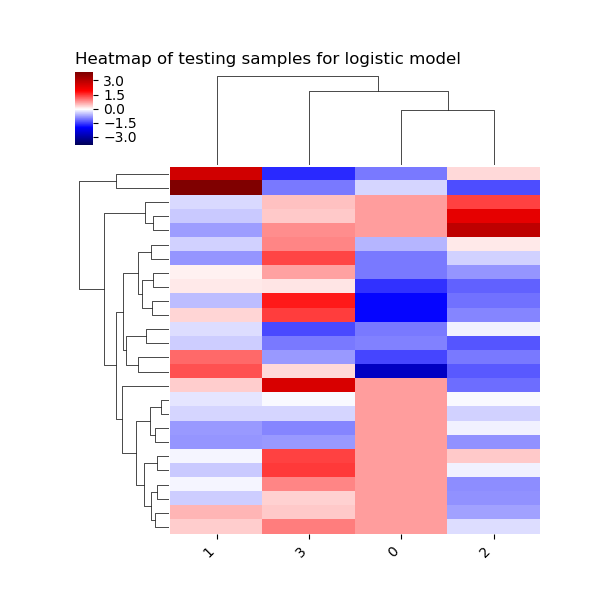


Correlation coefficient figure of the training samples


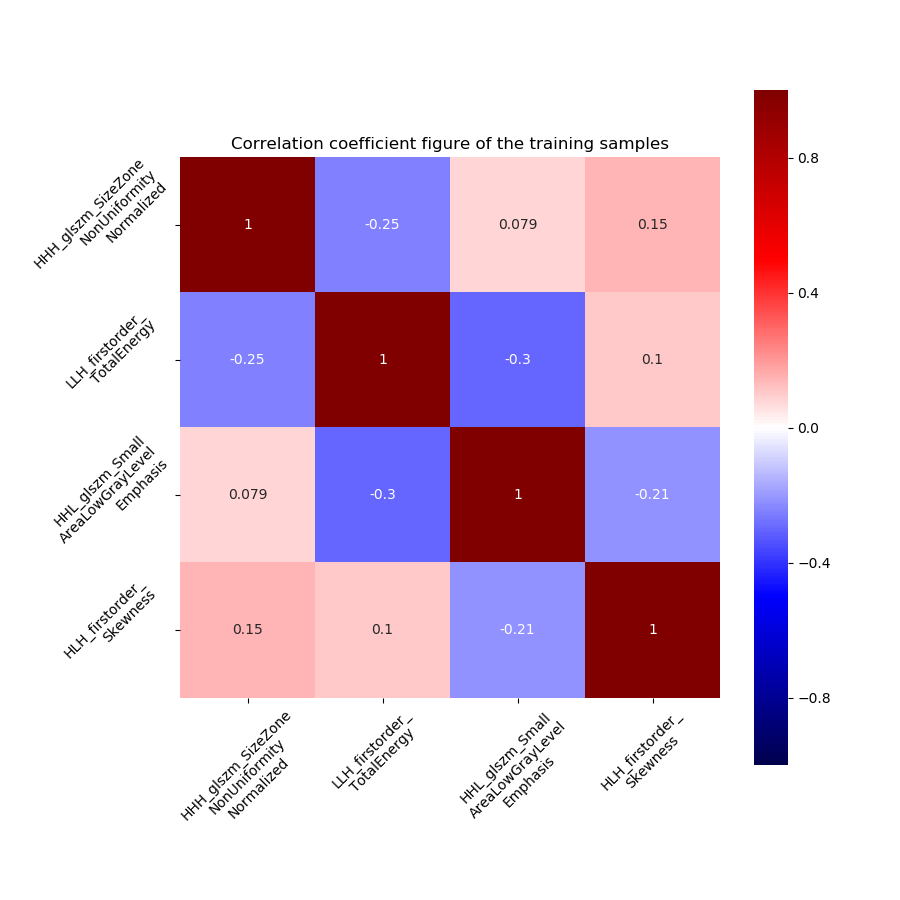


Correlation coefficient figure of the testing samples


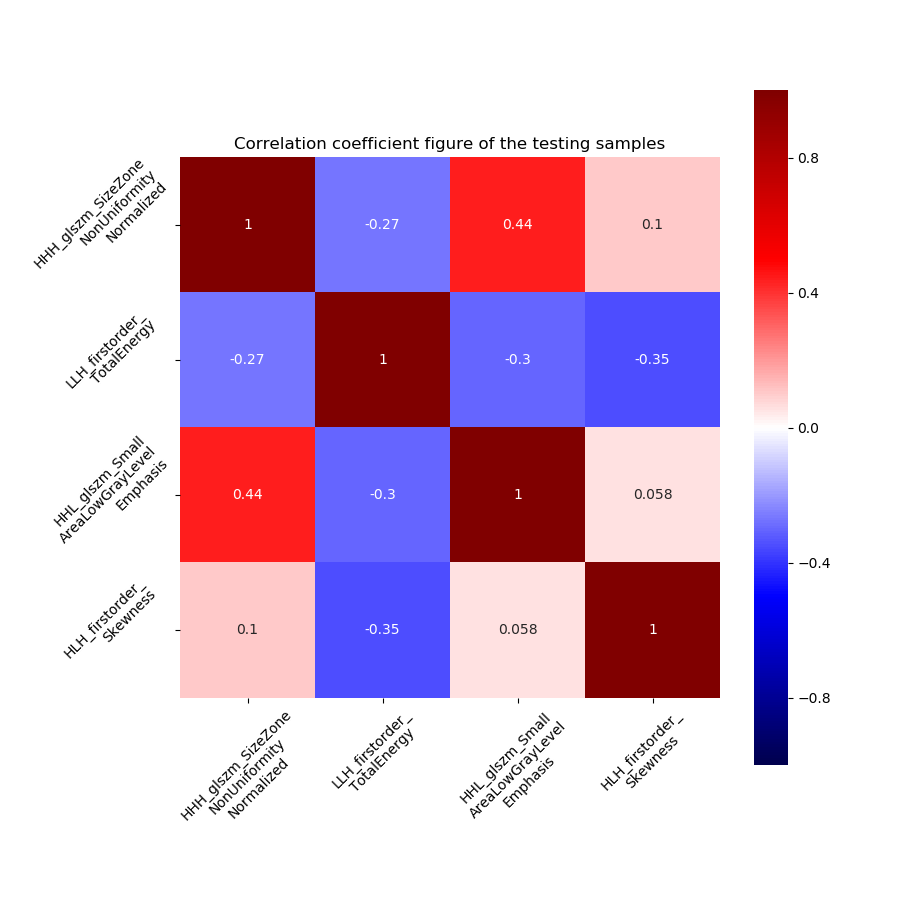


# 9. The best machine learning method: Logistic

the parameters selection method and selected parameters:
 {' ': ''}

the separate scores and total mean scores of model in each validation fold:
 {'scores': [0.8333333333333334, 0.6666666666666666, 0.8333333333333334, 0.6666666666666666, 0.75], 'mean_score': 0.75}

The best cutoff value of logistic model :

| best cutoff value |
| --- |
| 0.415 |

evaluation of logistic model in the training and testing samples:

| Item | Train | Test |
| --- | --- | --- |
| Accuracy | 0.833 | 0.654 |
| f1_score | 0.75 | 0.6 |
| Recall | 0.75 | 0.556 |
| Precision | 0.75 | 0.5 |
| AUC | 0.881 (0.806, 0.947) | 0.804 (0.653, 0.943) |
| Sensitivity | 0.75 | 0.556 |
| Specificity | 0.875 | 0.706 |
| positive prediction | 0.75 | 0.5 |
| negative prediction | 0.875 | 0.75 |
| positive llr | 6.0 | 1.889 |
| negatice llr | 0.286 | 0.63 |

The p_value of HL-test:

| P_value(HL-test) |
| --- |
| 0.709 |

the statistic information summary of Logistic models:

| Model: | Logit | Pseudo R-squared: | 0.342 |
| --- | --- | --- | --- |
| Dependent Variable: | y | AIC: | 60.2212 |
| Date: | 2022-01-17 10:08 | BIC: | 70.6930 |
| No. Observations: | 60 | Log-Likelihood: | -25.111 |
| Df Model: | 4 | LL-Null: | -38.191 |
| Df Residuals: | 55 | LLR p-value: | 2.9372e-05 |
| Converged: | 1.0000 | Scale: | 1.0000 |
| No. Iterations: | 10.0000 |  |  |

| variables | Coef. | Std.Err. | z | P>\|z\| | [0.025 | 0.975] |
| --- | --- | --- | --- | --- | --- | --- |
| intercept | -1.3214 | 0.4622 | -2.8587 | 0.0043 | -2.2274 | -0.4154 |
| wavelet-HHH_glszm_SizeZoneNonUniformityNormalized | 1.7520 | 0.6317 | 2.7733 | 0.0055 | 0.5138 | 2.9902 |
| wavelet-LLH_firstorder_TotalEnergy | 1.5243 | 0.5540 | 2.7516 | 0.0059 | 0.4386 | 2.6101 |
| wavelet-HHL_glszm_SmallAreaLowGrayLevelEmphasis | 1.1466 | 0.4438 | 2.5834 | 0.0098 | 0.2767 | 2.0165 |
| wavelet-HLH_firstorder_Skewness | 0.9943 | 0.4701 | 2.1149 | 0.0344 | 0.0728 | 1.9158 |

ROC of logistic model in the training samples:


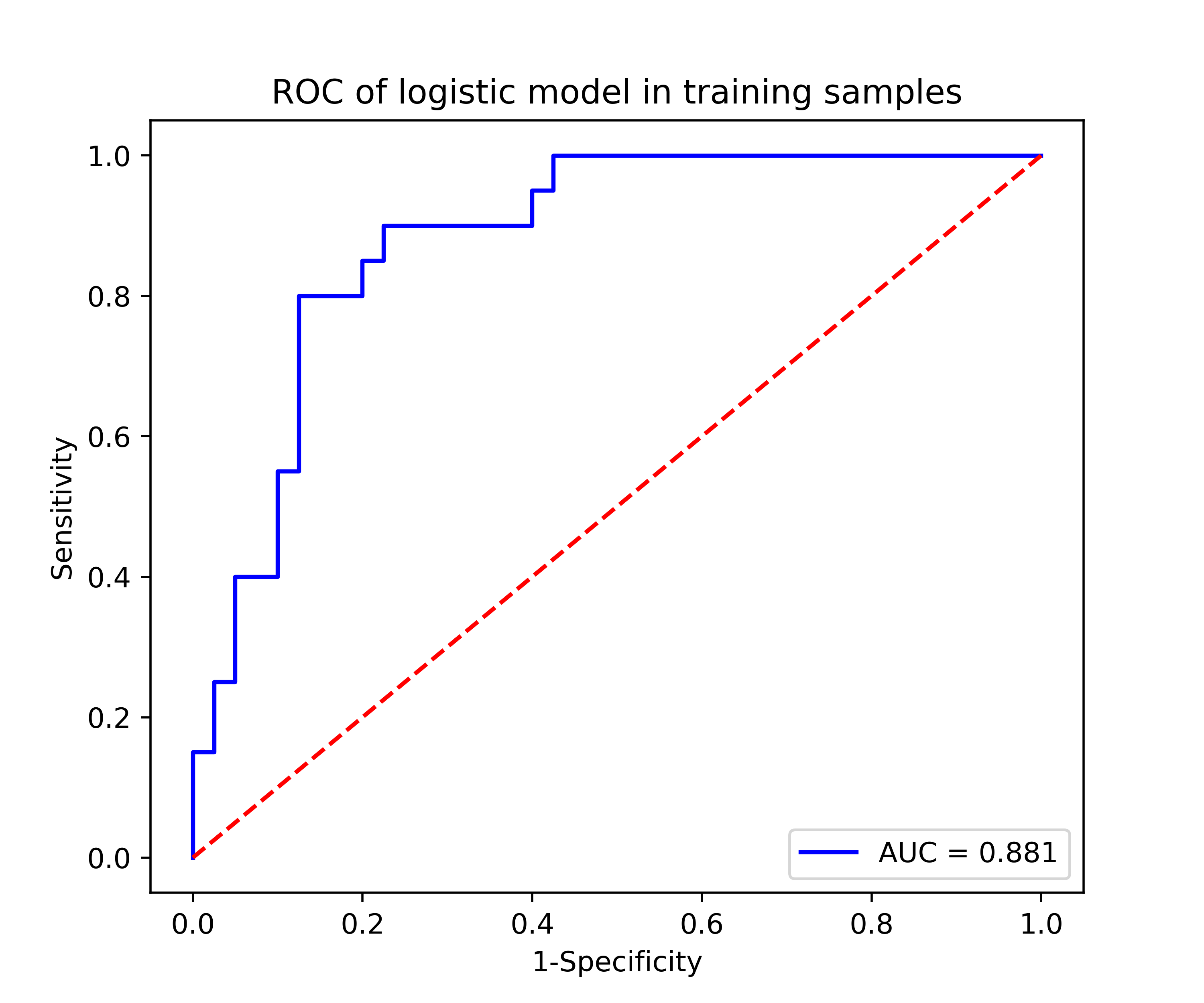


ROC of logistic model in the testing samples:


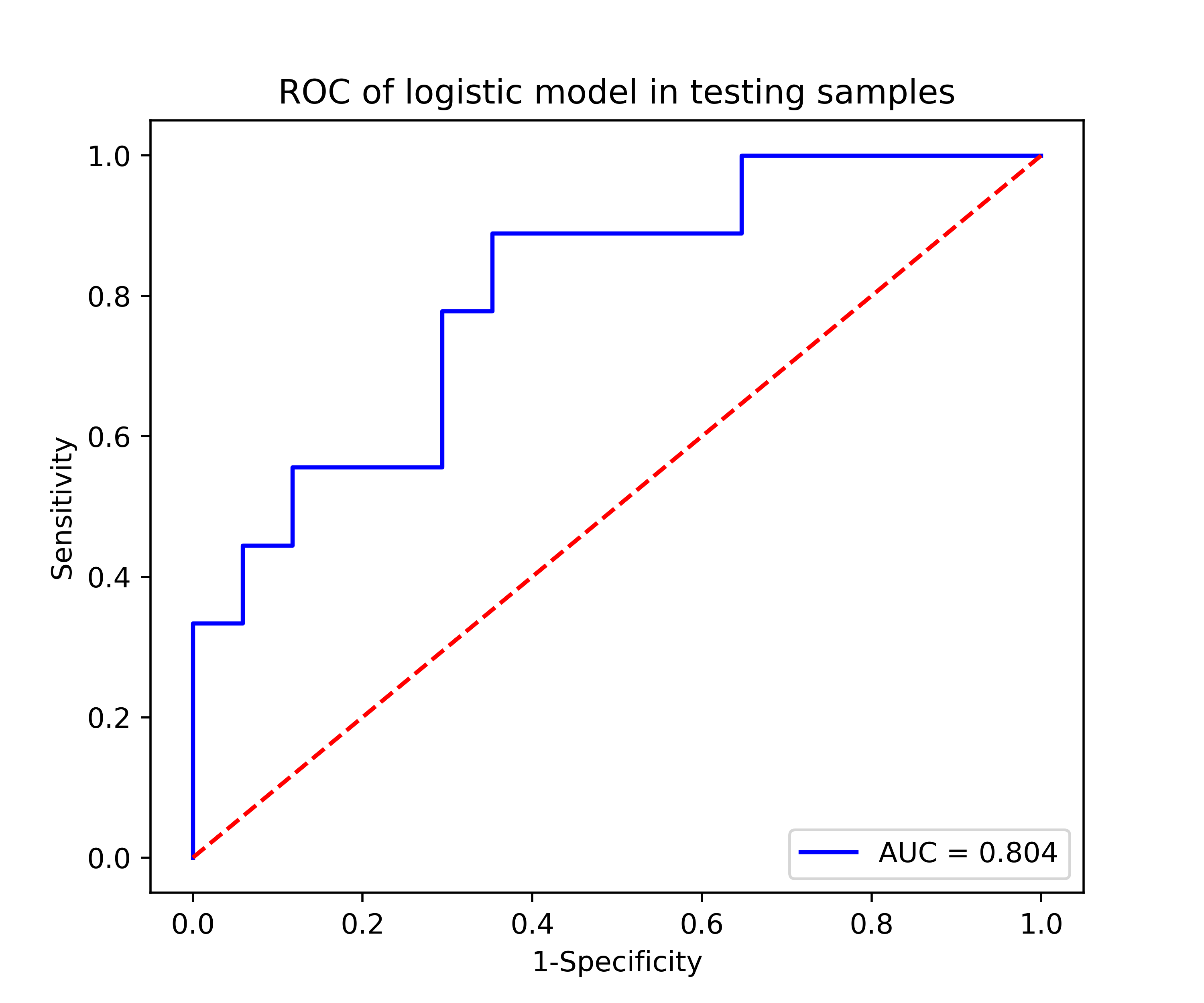


Calibration plot of logistic model in training samples:


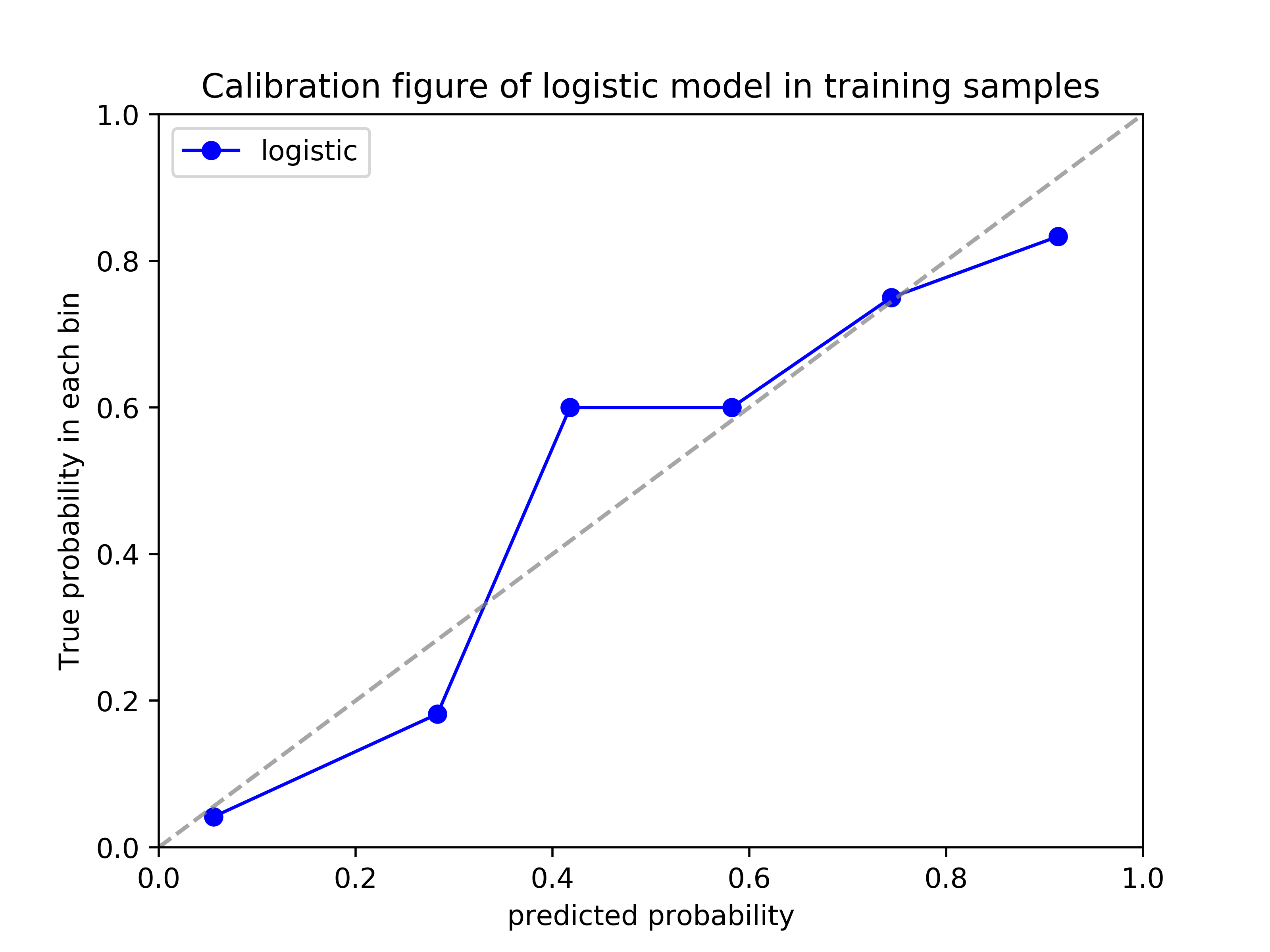


Calibration plot of logistic model in testing samples:


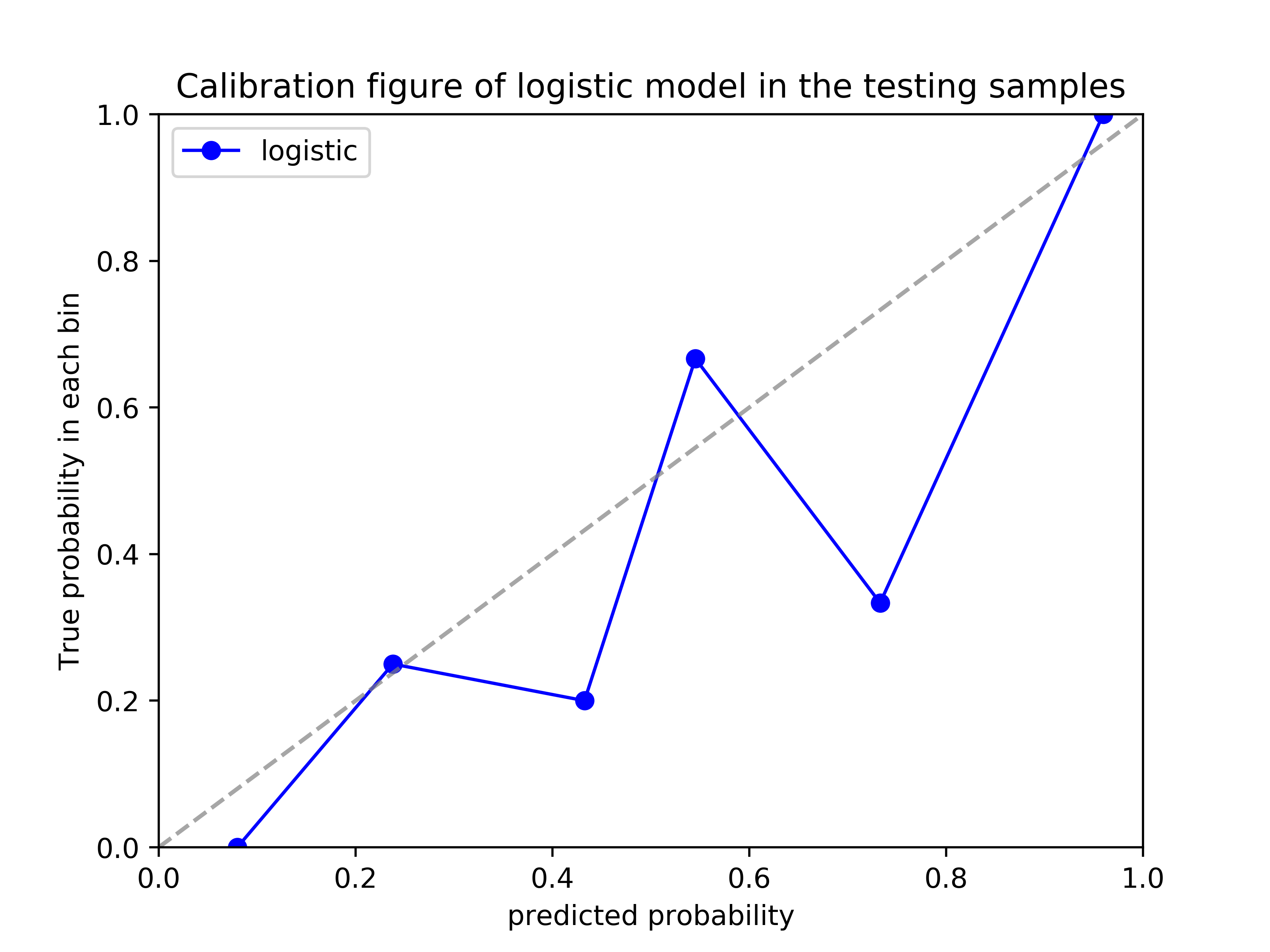


Rad score plot of logistic model in the training samples:


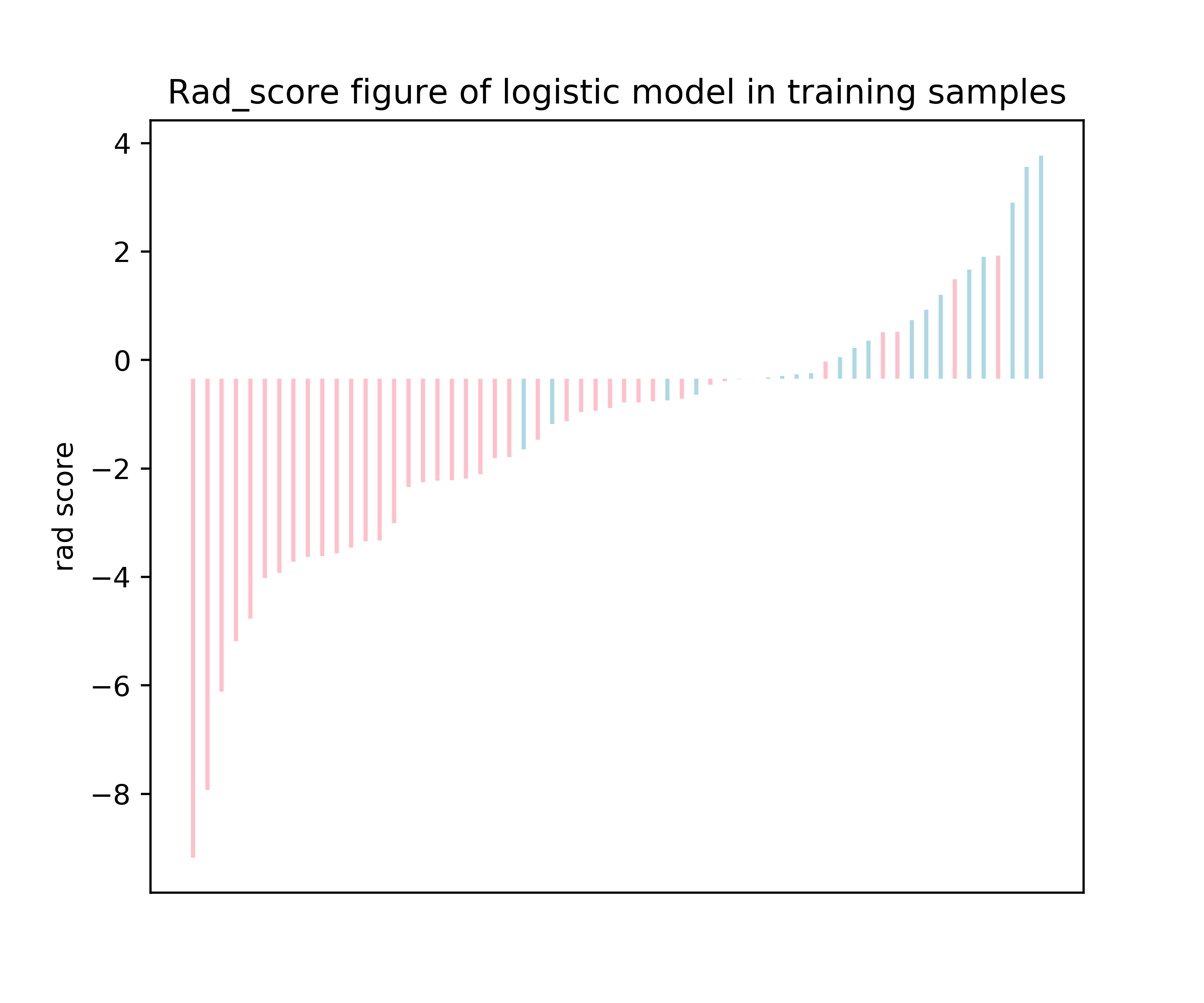


Rad score plot of logistic model in the testing samples:


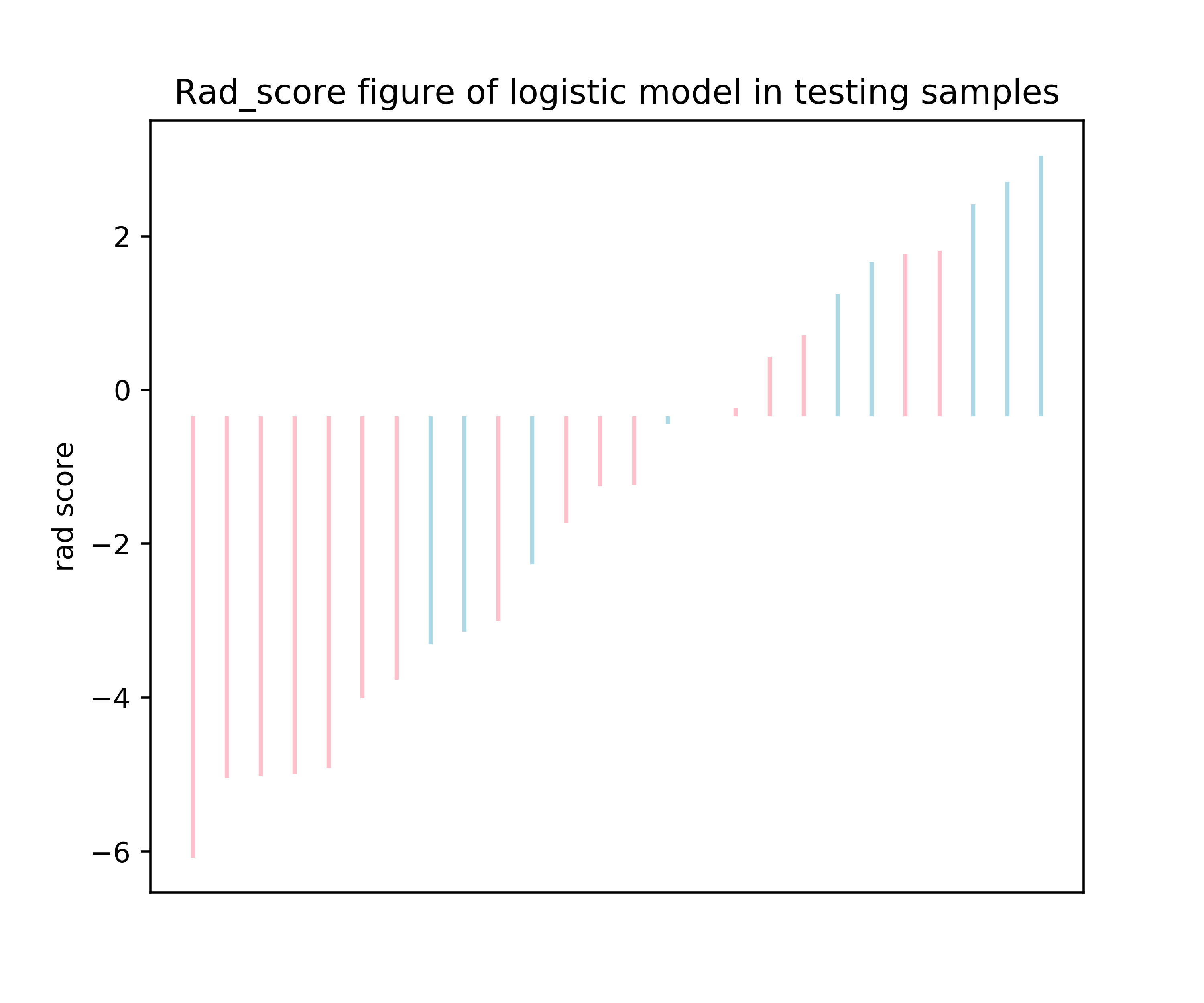


Decision Curve of logistic model in training samples:


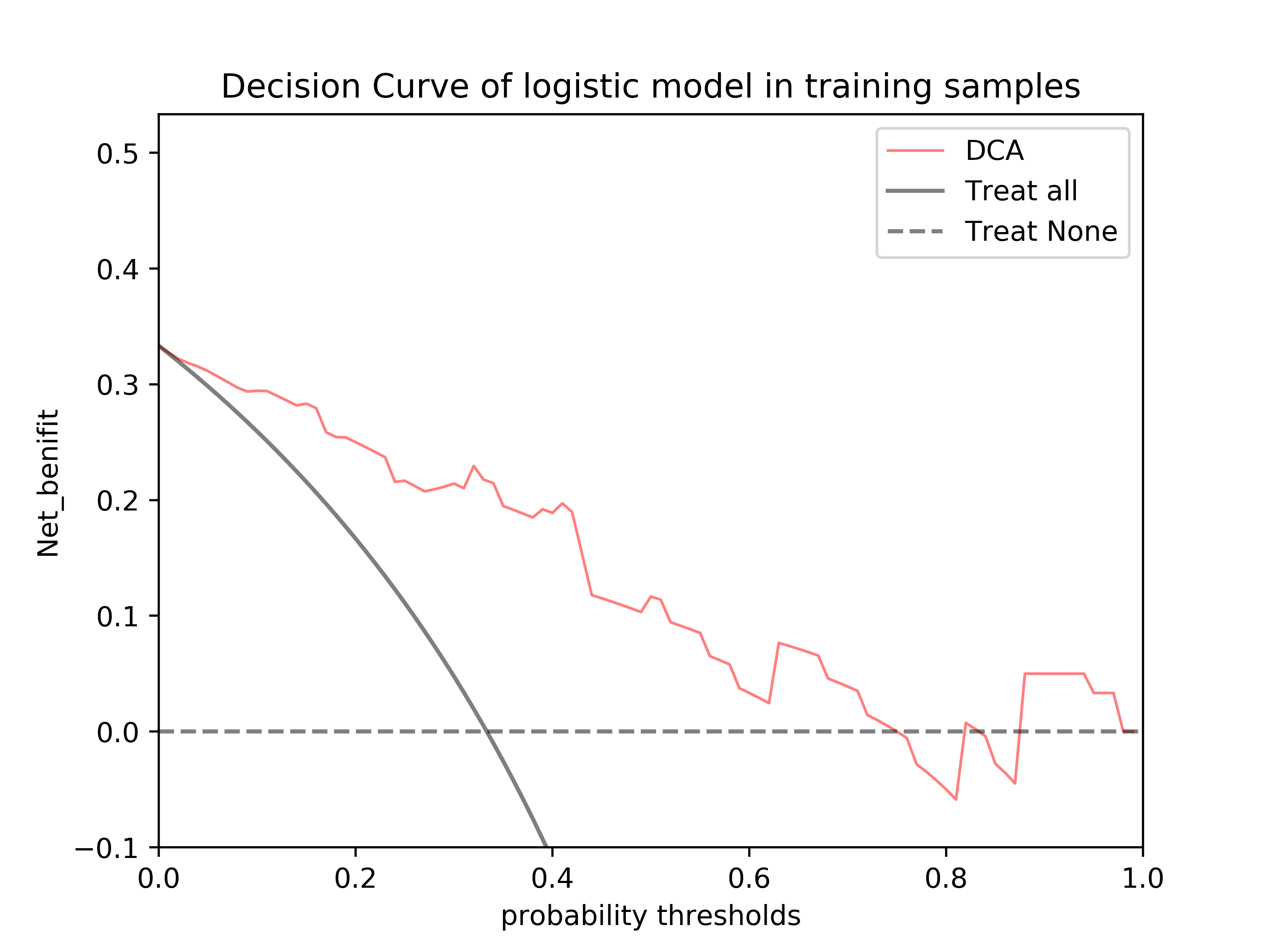


Decision Curve of logistic model in testing samples:


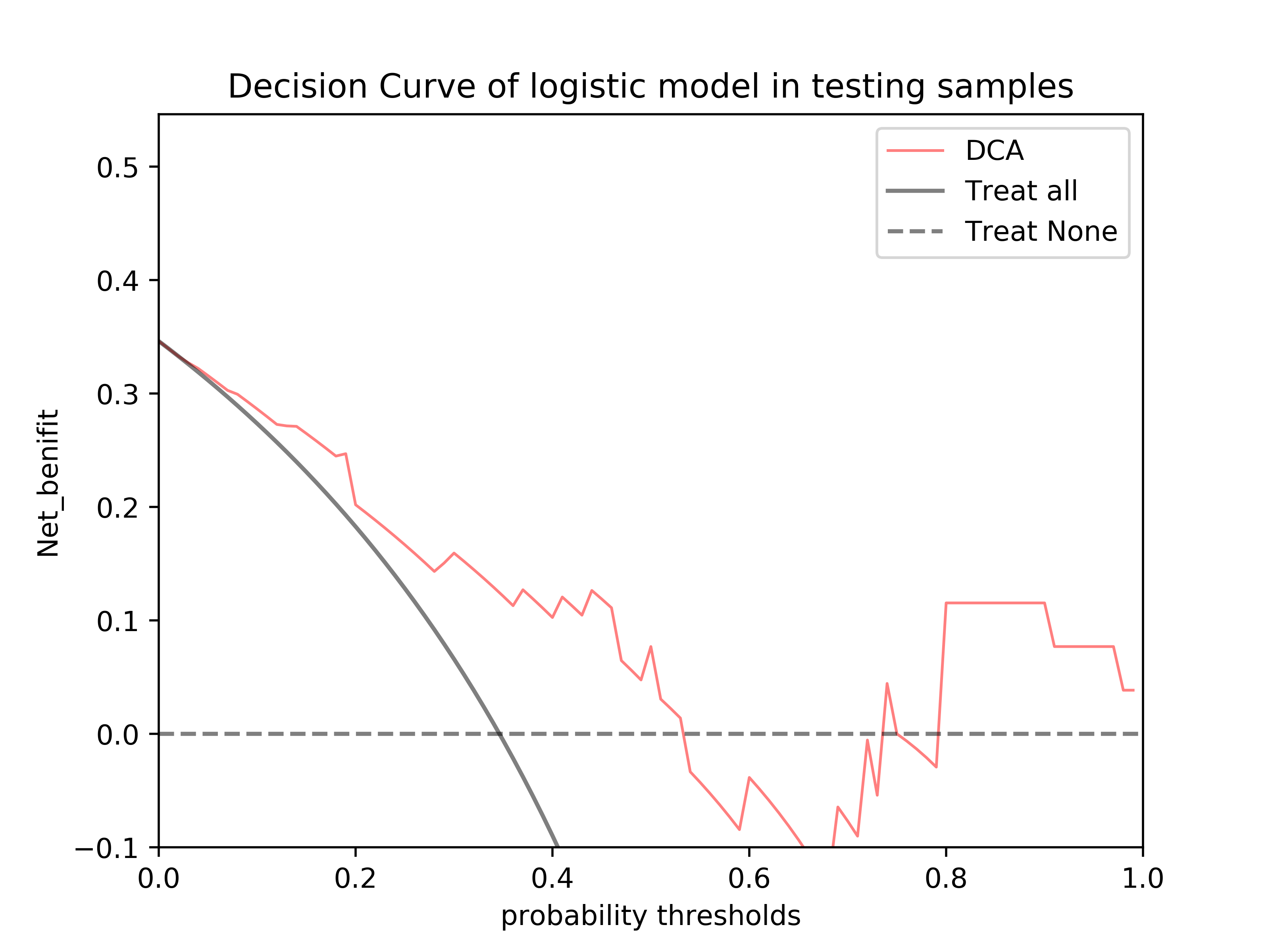


PR Curve of logistic model in training samples:


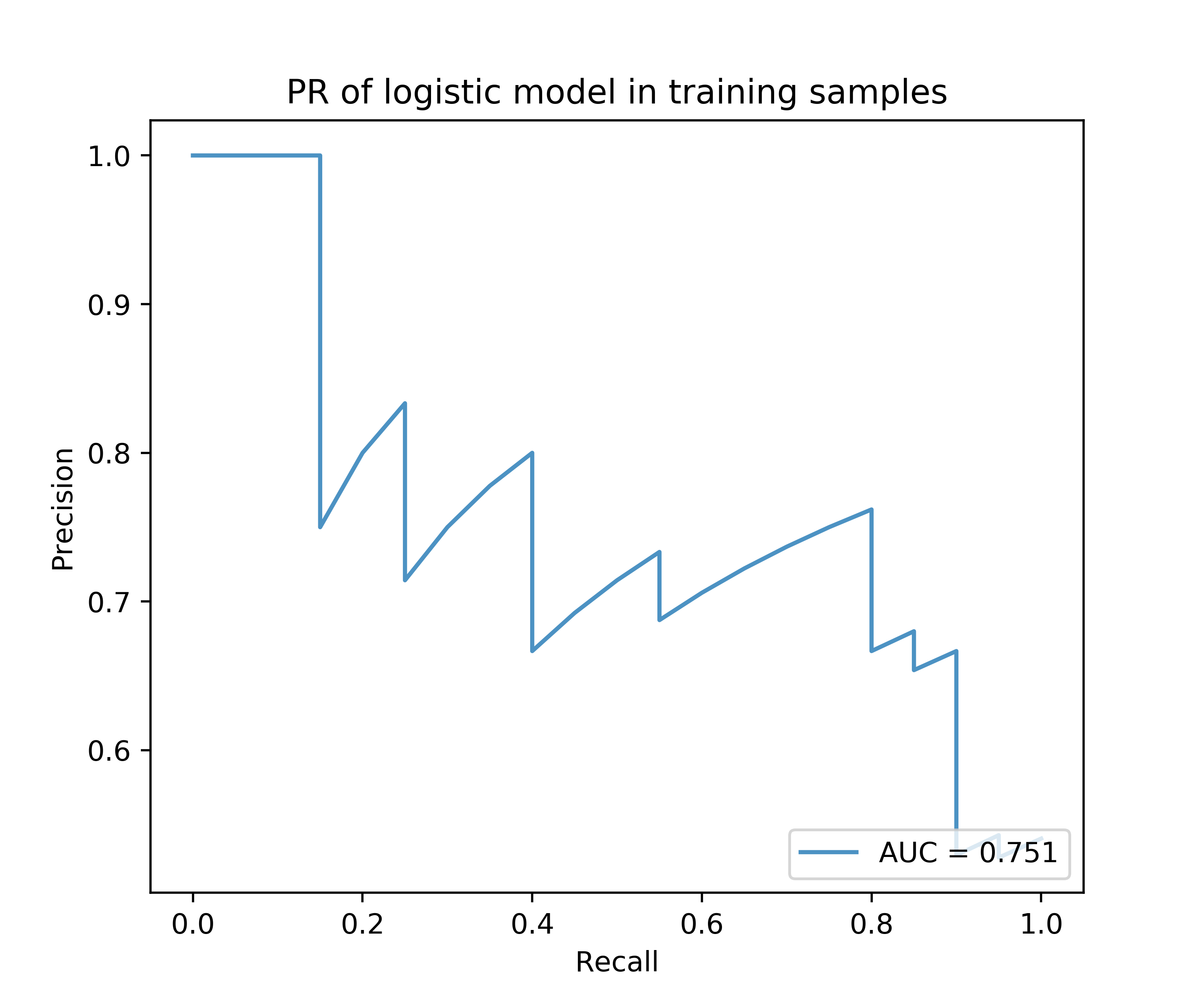


PR Curve of logistic model in testing samples:


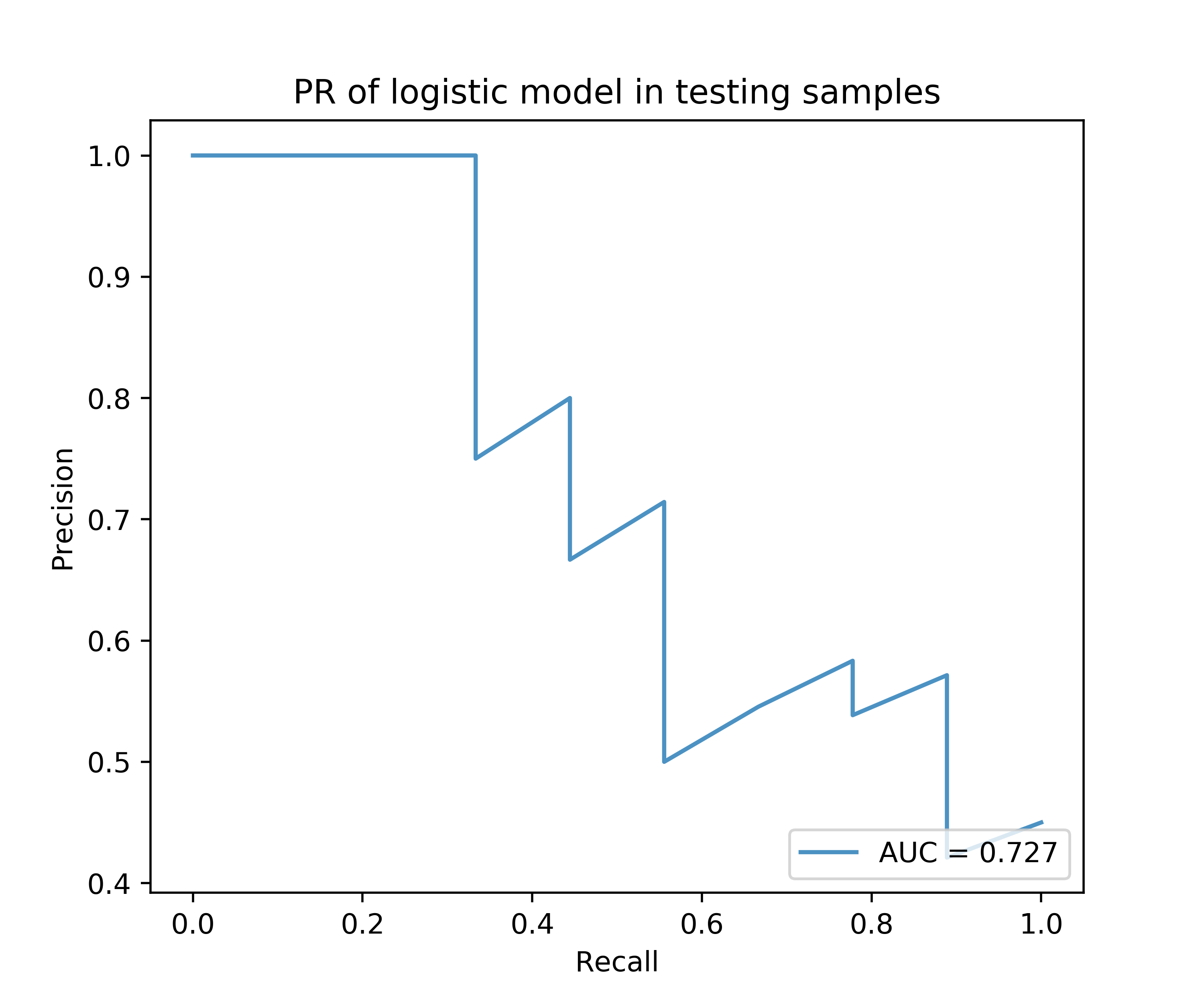


Nomograme plot of the Logistic model in the training samples:

You had not made a nomogram

**Radiomics analysis process of support vector machine.**

Summary Report

# The summary report recognized the best model from all models your training, and summarized its training process.

As the result shown, the best model was SVM, its training process including: ['select a path', 'set a seed', 'seperate a data', 'input data', 'standardize data', 'select feature', 'select feature', 'select feature', 'machine_learning']. Detailed information is shown below:

# 1. Data: C:/Users/212768837/Desktop/zhuanyi_86ROI/new/result2.csv

# 2. Random seed: 68

# 3. Seperative rate: 0.7

Seperated report:

|  | Sum | Pos | Neg |
| --- | --- | --- | --- |
| data | 86 | 29 | 57 |
| train | 60 | 20 | 40 |
| test | 26 | 9 | 17 |

# 4. Input data

The method for filling the missing data: Median

The method for dealing with outliers: Median

# 5. The method for standardizing the data: Standardization

# 6. The method for selecting features: Variance

parameters setted: {'threshold': 1.0}
num of remained features: 419
remained features:
[['original_shape_Elongation']
 ['original_shape_Flatness']
 ['original_shape_LeastAxisLength']
 ['original_shape_MajorAxisLength']
 ['original_shape_Maximum2DDiameterColumn']
 ['original_shape_Maximum3DDiameter']
 ['original_shape_MinorAxisLength']
 ['original_shape_SurfaceVolumeRatio']
 ['original_shape_VoxelVolume']
 ['original_firstorder_90Percentile']
 ['original_firstorder_Entropy']
 ['original_firstorder_Kurtosis']
 ['original_firstorder_Maximum']
 ['original_firstorder_Minimum']
 ['original_firstorder_Uniformity']
 ['original_glcm_Autocorrelation']
 ['original_glcm_ClusterProminence']
 ['original_glcm_ClusterShade']
 ['original_glcm_ClusterTendency']
 ['original_glcm_DifferenceEntropy']
 ['original_glcm_DifferenceVariance']
 ['original_glcm_Idm']
 ['original_glcm_Idmn']
 ['original_glcm_Idn']
 ['original_glcm_Imc1']
 ['original_glcm_InverseVariance']
 ['original_glcm_MCC']
 ['original_gldm_DependenceEntropy']
 ['original_gldm_DependenceVariance']
 ['original_gldm_GrayLevelNonUniformity']
 ['original_gldm_HighGrayLevelEmphasis']
 ['original_gldm_LargeDependenceEmphasis']
 ['original_gldm_LargeDependenceLowGrayLevelEmphasis']
 ['original_gldm_LowGrayLevelEmphasis']
 ['original_gldm_SmallDependenceEmphasis']
 ['original_gldm_SmallDependenceHighGrayLevelEmphasis']
 ['original_glrlm_GrayLevelVariance']
 ['original_glrlm_LongRunHighGrayLevelEmphasis']
 ['original_glrlm_LongRunLowGrayLevelEmphasis']
 ['original_glrlm_LowGrayLevelRunEmphasis']
 ['original_glrlm_RunEntropy']
 ['original_glrlm_ShortRunEmphasis']
 ['original_glszm_GrayLevelNonUniformityNormalized']
 ['original_glszm_GrayLevelVariance']
 ['original_glszm_HighGrayLevelZoneEmphasis']
 ['original_glszm_SizeZoneNonUniformity']
 ['original_glszm_SizeZoneNonUniformityNormalized']
 ['original_glszm_SmallAreaEmphasis']
 ['original_glszm_SmallAreaLowGrayLevelEmphasis']
 ['original_glszm_ZonePercentage']
 ['original_ngtdm_Busyness']
 ['original_ngtdm_Coarseness']
 ['original_ngtdm_Complexity']
 ['original_ngtdm_Contrast']
 ['original_ngtdm_Strength']
 ['wavelet-LLH_firstorder_10Percentile']
 ['wavelet-LLH_firstorder_90Percentile']
 ['wavelet-LLH_firstorder_Energy']
 ['wavelet-LLH_firstorder_Entropy']
 ['wavelet-LLH_firstorder_InterquartileRange']
 ['wavelet-LLH_firstorder_Kurtosis']
 ['wavelet-LLH_firstorder_Median']
 ['wavelet-LLH_firstorder_Skewness']
 ['wavelet-LLH_firstorder_TotalEnergy']
 ['wavelet-LLH_glcm_ClusterProminence']
 ['wavelet-LLH_glcm_Contrast']
 ['wavelet-LLH_glcm_Correlation']
 ['wavelet-LLH_glcm_DifferenceAverage']
 ['wavelet-LLH_glcm_DifferenceVariance']
 ['wavelet-LLH_glcm_Id']
 ['wavelet-LLH_glcm_Idm']
 ['wavelet-LLH_glcm_Imc1']
 ['wavelet-LLH_glcm_Imc2']
 ['wavelet-LLH_glcm_JointEnergy']
 ['wavelet-LLH_glcm_MaximumProbability']
 ['wavelet-LLH_glcm_SumEntropy']
 ['wavelet-LLH_glcm_SumSquares']
 ['wavelet-LLH_gldm_DependenceEntropy']
 ['wavelet-LLH_gldm_DependenceNonUniformityNormalized']
 ['wavelet-LLH_gldm_DependenceVariance']
 ['wavelet-LLH_gldm_LargeDependenceEmphasis']
 ['wavelet-LLH_gldm_SmallDependenceHighGrayLevelEmphasis']
 ['wavelet-LLH_glrlm_GrayLevelNonUniformityNormalized']
 ['wavelet-LLH_glrlm_GrayLevelVariance']
 ['wavelet-LLH_glrlm_LowGrayLevelRunEmphasis']
 ['wavelet-LLH_glrlm_RunEntropy']
 ['wavelet-LLH_glrlm_RunLengthNonUniformity']
 ['wavelet-LLH_glrlm_RunLengthNonUniformityNormalized']
 ['wavelet-LLH_glrlm_RunPercentage']
 ['wavelet-LLH_glrlm_RunVariance']
 ['wavelet-LLH_glrlm_ShortRunEmphasis']
 ['wavelet-LLH_glszm_GrayLevelNonUniformity']
 ['wavelet-LLH_glszm_LargeAreaEmphasis']
 ['wavelet-LLH_glszm_LargeAreaHighGrayLevelEmphasis']
 ['wavelet-LLH_glszm_LowGrayLevelZoneEmphasis']
 ['wavelet-LLH_glszm_SizeZoneNonUniformityNormalized']
 ['wavelet-LLH_glszm_SmallAreaHighGrayLevelEmphasis']
 ['wavelet-LLH_glszm_ZoneVariance']
 ['wavelet-LLH_ngtdm_Contrast']
 ['wavelet-LHL_firstorder_10Percentile']
 ['wavelet-LHL_firstorder_Energy']
 ['wavelet-LHL_firstorder_Kurtosis']
 ['wavelet-LHL_firstorder_Maximum']
 ['wavelet-LHL_firstorder_Median']
 ['wavelet-LHL_firstorder_RobustMeanAbsoluteDeviation']
 ['wavelet-LHL_firstorder_RootMeanSquared']
 ['wavelet-LHL_firstorder_TotalEnergy']
 ['wavelet-LHL_glcm_Autocorrelation']
 ['wavelet-LHL_glcm_ClusterProminence']
 ['wavelet-LHL_glcm_Contrast']
 ['wavelet-LHL_glcm_DifferenceAverage']
 ['wavelet-LHL_glcm_DifferenceEntropy']
 ['wavelet-LHL_glcm_DifferenceVariance']
 ['wavelet-LHL_glcm_Idm']
 ['wavelet-LHL_glcm_Idmn']
 ['wavelet-LHL_glcm_Imc2']
 ['wavelet-LHL_glcm_MCC']
 ['wavelet-LHL_glcm_MaximumProbability']
 ['wavelet-LHL_glcm_SumEntropy']
 ['wavelet-LHL_glcm_SumSquares']
 ['wavelet-LHL_gldm_DependenceEntropy']
 ['wavelet-LHL_gldm_DependenceNonUniformity']
 ['wavelet-LHL_gldm_DependenceNonUniformityNormalized']
 ['wavelet-LHL_gldm_GrayLevelNonUniformity']
 ['wavelet-LHL_gldm_GrayLevelVariance']
 ['wavelet-LHL_gldm_LargeDependenceEmphasis']
 ['wavelet-LHL_gldm_LargeDependenceHighGrayLevelEmphasis']
 ['wavelet-LHL_gldm_LargeDependenceLowGrayLevelEmphasis']
 ['wavelet-LHL_gldm_LowGrayLevelEmphasis']
 ['wavelet-LHL_glrlm_GrayLevelNonUniformityNormalized']
 ['wavelet-LHL_glrlm_LongRunEmphasis']
 ['wavelet-LHL_glrlm_LowGrayLevelRunEmphasis']
 ['wavelet-LHL_glrlm_RunEntropy']
 ['wavelet-LHL_glrlm_RunLengthNonUniformity']
 ['wavelet-LHL_glrlm_RunLengthNonUniformityNormalized']
 ['wavelet-LHL_glrlm_RunVariance']
 ['wavelet-LHL_glrlm_ShortRunEmphasis']
 ['wavelet-LHL_glrlm_ShortRunLowGrayLevelEmphasis']
 ['wavelet-LHL_glszm_GrayLevelVariance']
 ['wavelet-LHL_glszm_HighGrayLevelZoneEmphasis']
 ['wavelet-LHL_glszm_LargeAreaEmphasis']
 ['wavelet-LHL_glszm_LargeAreaLowGrayLevelEmphasis']
 ['wavelet-LHL_glszm_SizeZoneNonUniformity']
 ['wavelet-LHL_glszm_SmallAreaHighGrayLevelEmphasis']
 ['wavelet-LHL_glszm_SmallAreaLowGrayLevelEmphasis']
 ['wavelet-LHL_glszm_ZoneEntropy']
 ['wavelet-LHL_glszm_ZonePercentage']
 ['wavelet-LHL_glszm_ZoneVariance']
 ['wavelet-LHL_ngtdm_Busyness']
 ['wavelet-LHL_ngtdm_Coarseness']
 ['wavelet-LHL_ngtdm_Complexity']
 ['wavelet-LHL_ngtdm_Contrast']
 ['wavelet-LHL_ngtdm_Strength']
 ['wavelet-LHH_firstorder_90Percentile']
 ['wavelet-LHH_firstorder_Energy']
 ['wavelet-LHH_firstorder_InterquartileRange']
 ['wavelet-LHH_firstorder_Kurtosis']
 ['wavelet-LHH_firstorder_Maximum']
 ['wavelet-LHH_firstorder_MeanAbsoluteDeviation']
 ['wavelet-LHH_firstorder_Mean']
 ['wavelet-LHH_firstorder_Range']
 ['wavelet-LHH_firstorder_RobustMeanAbsoluteDeviation']
 ['wavelet-LHH_firstorder_TotalEnergy']
 ['wavelet-LHH_firstorder_Uniformity']
 ['wavelet-LHH_firstorder_Variance']
 ['wavelet-LHH_glcm_ClusterShade']
 ['wavelet-LHH_glcm_Contrast']
 ['wavelet-LHH_glcm_DifferenceVariance']
 ['wavelet-LHH_glcm_Idmn']
 ['wavelet-LHH_glcm_Idn']
 ['wavelet-LHH_glcm_JointAverage']
 ['wavelet-LHH_glcm_JointEnergy']
 ['wavelet-LHH_glcm_JointEntropy']
 ['wavelet-LHH_glcm_MCC']
 ['wavelet-LHH_glcm_MaximumProbability']
 ['wavelet-LHH_glcm_SumAverage']
 ['wavelet-LHH_glcm_SumEntropy']
 ['wavelet-LHH_glcm_SumSquares']
 ['wavelet-LHH_gldm_DependenceNonUniformity']
 ['wavelet-LHH_gldm_DependenceVariance']
 ['wavelet-LHH_gldm_GrayLevelVariance']
 ['wavelet-LHH_gldm_HighGrayLevelEmphasis']
 ['wavelet-LHH_gldm_LargeDependenceEmphasis']
 ['wavelet-LHH_gldm_LargeDependenceHighGrayLevelEmphasis']
 ['wavelet-LHH_gldm_LargeDependenceLowGrayLevelEmphasis']
 ['wavelet-LHH_gldm_LowGrayLevelEmphasis']
 ['wavelet-LHH_gldm_SmallDependenceHighGrayLevelEmphasis']
 ['wavelet-LHH_glrlm_GrayLevelNonUniformity']
 ['wavelet-LHH_glrlm_GrayLevelVariance']
 ['wavelet-LHH_glrlm_HighGrayLevelRunEmphasis']
 ['wavelet-LHH_glrlm_LongRunEmphasis']
 ['wavelet-LHH_glrlm_LongRunHighGrayLevelEmphasis']
 ['wavelet-LHH_glrlm_LowGrayLevelRunEmphasis']
 ['wavelet-LHH_glrlm_RunEntropy']
 ['wavelet-LHH_glrlm_RunLengthNonUniformityNormalized']
 ['wavelet-LHH_glrlm_RunVariance']
 ['wavelet-LHH_glrlm_ShortRunEmphasis']
 ['wavelet-LHH_glrlm_ShortRunLowGrayLevelEmphasis']
 ['wavelet-LHH_glszm_GrayLevelVariance']
 ['wavelet-LHH_glszm_HighGrayLevelZoneEmphasis']
 ['wavelet-LHH_glszm_LargeAreaHighGrayLevelEmphasis']
 ['wavelet-LHH_glszm_LargeAreaLowGrayLevelEmphasis']
 ['wavelet-LHH_glszm_SizeZoneNonUniformity']
 ['wavelet-LHH_glszm_SizeZoneNonUniformityNormalized']
 ['wavelet-LHH_glszm_SmallAreaEmphasis']
 ['wavelet-LHH_glszm_SmallAreaHighGrayLevelEmphasis']
 ['wavelet-LHH_glszm_ZonePercentage']
 ['wavelet-HLL_firstorder_InterquartileRange']
 ['wavelet-HLL_firstorder_Kurtosis']
 ['wavelet-HLL_firstorder_Maximum']
 ['wavelet-HLL_firstorder_MeanAbsoluteDeviation']
 ['wavelet-HLL_firstorder_Mean']
 ['wavelet-HLL_firstorder_Median']
 ['wavelet-HLL_firstorder_Range']
 ['wavelet-HLL_firstorder_Skewness']
 ['wavelet-HLL_firstorder_Uniformity']
 ['wavelet-HLL_firstorder_Variance']
 ['wavelet-HLL_glcm_Autocorrelation']
 ['wavelet-HLL_glcm_ClusterProminence']
 ['wavelet-HLL_glcm_Contrast']
 ['wavelet-HLL_glcm_Correlation']
 ['wavelet-HLL_glcm_DifferenceEntropy']
 ['wavelet-HLL_glcm_DifferenceVariance']
 ['wavelet-HLL_glcm_Id']
 ['wavelet-HLL_glcm_Idmn']
 ['wavelet-HLL_glcm_Imc2']
 ['wavelet-HLL_glcm_InverseVariance']
 ['wavelet-HLL_glcm_SumEntropy']
 ['wavelet-HLL_gldm_DependenceEntropy']
 ['wavelet-HLL_gldm_DependenceNonUniformity']
 ['wavelet-HLL_gldm_GrayLevelNonUniformity']
 ['wavelet-HLL_gldm_HighGrayLevelEmphasis']
 ['wavelet-HLL_gldm_LargeDependenceLowGrayLevelEmphasis']
 ['wavelet-HLL_gldm_LowGrayLevelEmphasis']
 ['wavelet-HLL_gldm_SmallDependenceEmphasis']
 ['wavelet-HLL_gldm_SmallDependenceHighGrayLevelEmphasis']
 ['wavelet-HLL_glrlm_GrayLevelNonUniformity']
 ['wavelet-HLL_glrlm_GrayLevelVariance']
 ['wavelet-HLL_glrlm_HighGrayLevelRunEmphasis']
 ['wavelet-HLL_glrlm_LongRunEmphasis']
 ['wavelet-HLL_glrlm_LongRunHighGrayLevelEmphasis']
 ['wavelet-HLL_glrlm_RunLengthNonUniformity']
 ['wavelet-HLL_glrlm_RunLengthNonUniformityNormalized']
 ['wavelet-HLL_glrlm_ShortRunLowGrayLevelEmphasis']
 ['wavelet-HLL_glszm_GrayLevelNonUniformityNormalized']
 ['wavelet-HLL_glszm_GrayLevelVariance']
 ['wavelet-HLL_glszm_HighGrayLevelZoneEmphasis']
 ['wavelet-HLL_glszm_LargeAreaHighGrayLevelEmphasis']
 ['wavelet-HLL_glszm_SizeZoneNonUniformity']
 ['wavelet-HLL_ngtdm_Complexity']
 ['wavelet-HLL_ngtdm_Strength']
 ['wavelet-HLH_firstorder_10Percentile']
 ['wavelet-HLH_firstorder_Entropy']
 ['wavelet-HLH_firstorder_Mean']
 ['wavelet-HLH_firstorder_Median']
 ['wavelet-HLH_firstorder_Minimum']
 ['wavelet-HLH_firstorder_Range']
 ['wavelet-HLH_firstorder_RootMeanSquared']
 ['wavelet-HLH_firstorder_Skewness']
 ['wavelet-HLH_firstorder_Uniformity']
 ['wavelet-HLH_glcm_ClusterProminence']
 ['wavelet-HLH_glcm_ClusterTendency']
 ['wavelet-HLH_glcm_Contrast']
 ['wavelet-HLH_glcm_DifferenceAverage']
 ['wavelet-HLH_glcm_DifferenceEntropy']
 ['wavelet-HLH_glcm_Idn']
 ['wavelet-HLH_glcm_JointAverage']
 ['wavelet-HLH_glcm_MCC']
 ['wavelet-HLH_glcm_MaximumProbability']
 ['wavelet-HLH_glcm_SumAverage']
 ['wavelet-HLH_glcm_SumSquares']
 ['wavelet-HLH_gldm_DependenceEntropy']
 ['wavelet-HLH_gldm_DependenceVariance']
 ['wavelet-HLH_gldm_GrayLevelNonUniformity']
 ['wavelet-HLH_gldm_GrayLevelVariance']
 ['wavelet-HLH_gldm_HighGrayLevelEmphasis']
 ['wavelet-HLH_gldm_LargeDependenceEmphasis']
 ['wavelet-HLH_gldm_LargeDependenceHighGrayLevelEmphasis']
 ['wavelet-HLH_glrlm_GrayLevelNonUniformity']
 ['wavelet-HLH_glrlm_LongRunEmphasis']
 ['wavelet-HLH_glrlm_LongRunLowGrayLevelEmphasis']
 ['wavelet-HLH_glrlm_RunLengthNonUniformityNormalized']
 ['wavelet-HLH_glrlm_ShortRunEmphasis']
 ['wavelet-HLH_glrlm_ShortRunHighGrayLevelEmphasis']
 ['wavelet-HLH_glszm_GrayLevelNonUniformityNormalized']
 ['wavelet-HLH_glszm_GrayLevelVariance']
 ['wavelet-HLH_glszm_HighGrayLevelZoneEmphasis']
 ['wavelet-HLH_glszm_LargeAreaEmphasis']
 ['wavelet-HLH_glszm_LargeAreaLowGrayLevelEmphasis']
 ['wavelet-HLH_glszm_SmallAreaEmphasis']
 ['wavelet-HLH_glszm_ZoneVariance']
 ['wavelet-HLH_ngtdm_Coarseness']
 ['wavelet-HHL_firstorder_90Percentile']
 ['wavelet-HHL_firstorder_Maximum']
 ['wavelet-HHL_firstorder_Mean']
 ['wavelet-HHL_firstorder_Median']
 ['wavelet-HHL_firstorder_Range']
 ['wavelet-HHL_firstorder_RootMeanSquared']
 ['wavelet-HHL_firstorder_Variance']
 ['wavelet-HHL_glcm_Autocorrelation']
 ['wavelet-HHL_glcm_DifferenceAverage']
 ['wavelet-HHL_glcm_Idm']
 ['wavelet-HHL_glcm_Idmn']
 ['wavelet-HHL_glcm_JointAverage']
 ['wavelet-HHL_glcm_JointEntropy']
 ['wavelet-HHL_glcm_MCC']
 ['wavelet-HHL_glcm_SumAverage']
 ['wavelet-HHL_gldm_DependenceEntropy']
 ['wavelet-HHL_gldm_DependenceNonUniformity']
 ['wavelet-HHL_gldm_DependenceNonUniformityNormalized']
 ['wavelet-HHL_gldm_GrayLevelVariance']
 ['wavelet-HHL_gldm_HighGrayLevelEmphasis']
 ['wavelet-HHL_gldm_SmallDependenceEmphasis']
 ['wavelet-HHL_gldm_SmallDependenceLowGrayLevelEmphasis']
 ['wavelet-HHL_glrlm_GrayLevelNonUniformity']
 ['wavelet-HHL_glrlm_GrayLevelNonUniformityNormalized']
 ['wavelet-HHL_glrlm_HighGrayLevelRunEmphasis']
 ['wavelet-HHL_glrlm_LongRunEmphasis']
 ['wavelet-HHL_glrlm_LongRunHighGrayLevelEmphasis']
 ['wavelet-HHL_glrlm_LongRunLowGrayLevelEmphasis']
 ['wavelet-HHL_glrlm_RunLengthNonUniformity']
 ['wavelet-HHL_glrlm_RunPercentage']
 ['wavelet-HHL_glrlm_ShortRunEmphasis']
 ['wavelet-HHL_glszm_GrayLevelNonUniformity']
 ['wavelet-HHL_glszm_GrayLevelNonUniformityNormalized']
 ['wavelet-HHL_glszm_GrayLevelVariance']
 ['wavelet-HHL_glszm_HighGrayLevelZoneEmphasis']
 ['wavelet-HHL_glszm_LargeAreaEmphasis']
 ['wavelet-HHL_glszm_LargeAreaLowGrayLevelEmphasis']
 ['wavelet-HHL_glszm_LowGrayLevelZoneEmphasis']
 ['wavelet-HHL_glszm_SmallAreaEmphasis']
 ['wavelet-HHL_glszm_SmallAreaHighGrayLevelEmphasis']
 ['wavelet-HHL_glszm_SmallAreaLowGrayLevelEmphasis']
 ['wavelet-HHL_glszm_ZonePercentage']
 ['wavelet-HHL_ngtdm_Busyness']
 ['wavelet-HHL_ngtdm_Complexity']
 ['wavelet-HHL_ngtdm_Strength']
 ['wavelet-HHH_firstorder_10Percentile']
 ['wavelet-HHH_firstorder_Energy']
 ['wavelet-HHH_firstorder_Entropy']
 ['wavelet-HHH_firstorder_Kurtosis']
 ['wavelet-HHH_firstorder_MeanAbsoluteDeviation']
 ['wavelet-HHH_firstorder_Mean']
 ['wavelet-HHH_firstorder_Median']
 ['wavelet-HHH_firstorder_Minimum']
 ['wavelet-HHH_firstorder_RootMeanSquared']
 ['wavelet-HHH_firstorder_TotalEnergy']
 ['wavelet-HHH_firstorder_Variance']
 ['wavelet-HHH_glcm_ClusterTendency']
 ['wavelet-HHH_glcm_Contrast']
 ['wavelet-HHH_glcm_DifferenceEntropy']
 ['wavelet-HHH_glcm_Id']
 ['wavelet-HHH_glcm_Idmn']
 ['wavelet-HHH_glcm_Idn']
 ['wavelet-HHH_glcm_JointAverage']
 ['wavelet-HHH_glcm_JointEnergy']
 ['wavelet-HHH_glcm_JointEntropy']
 ['wavelet-HHH_glcm_SumAverage']
 ['wavelet-HHH_gldm_DependenceVariance']
 ['wavelet-HHH_gldm_GrayLevelNonUniformity']
 ['wavelet-HHH_gldm_GrayLevelVariance']
 ['wavelet-HHH_gldm_LargeDependenceHighGrayLevelEmphasis']
 ['wavelet-HHH_gldm_LargeDependenceLowGrayLevelEmphasis']
 ['wavelet-HHH_gldm_SmallDependenceEmphasis']
 ['wavelet-HHH_gldm_SmallDependenceHighGrayLevelEmphasis']
 ['wavelet-HHH_gldm_SmallDependenceLowGrayLevelEmphasis']
 ['wavelet-HHH_glrlm_GrayLevelNonUniformity']
 ['wavelet-HHH_glrlm_GrayLevelNonUniformityNormalized']
 ['wavelet-HHH_glrlm_LongRunLowGrayLevelEmphasis']
 ['wavelet-HHH_glrlm_RunLengthNonUniformity']
 ['wavelet-HHH_glrlm_RunPercentage']
 ['wavelet-HHH_glrlm_ShortRunHighGrayLevelEmphasis']
 ['wavelet-HHH_glszm_LargeAreaHighGrayLevelEmphasis']
 ['wavelet-HHH_glszm_SizeZoneNonUniformityNormalized']
 ['wavelet-HHH_glszm_SmallAreaEmphasis']
 ['wavelet-HHH_glszm_ZoneVariance']
 ['wavelet-HHH_ngtdm_Coarseness']
 ['wavelet-HHH_ngtdm_Contrast']
 ['wavelet-LLL_firstorder_Entropy']
 ['wavelet-LLL_firstorder_Kurtosis']
 ['wavelet-LLL_firstorder_Maximum']
 ['wavelet-LLL_firstorder_RobustMeanAbsoluteDeviation']
 ['wavelet-LLL_firstorder_Uniformity']
 ['wavelet-LLL_glcm_ClusterShade']
 ['wavelet-LLL_glcm_Idm']
 ['wavelet-LLL_glcm_Idn']
 ['wavelet-LLL_glcm_Imc1']
 ['wavelet-LLL_glcm_Imc2']
 ['wavelet-LLL_glcm_InverseVariance']
 ['wavelet-LLL_glcm_JointAverage']
 ['wavelet-LLL_glcm_MaximumProbability']
 ['wavelet-LLL_glcm_SumAverage']
 ['wavelet-LLL_glcm_SumEntropy']
 ['wavelet-LLL_gldm_DependenceEntropy']
 ['wavelet-LLL_gldm_DependenceNonUniformity']
 ['wavelet-LLL_gldm_GrayLevelVariance']
 ['wavelet-LLL_gldm_LargeDependenceEmphasis']
 ['wavelet-LLL_gldm_LargeDependenceHighGrayLevelEmphasis']
 ['wavelet-LLL_gldm_LargeDependenceLowGrayLevelEmphasis']
 ['wavelet-LLL_gldm_LowGrayLevelEmphasis']
 ['wavelet-LLL_glrlm_GrayLevelVariance']
 ['wavelet-LLL_glrlm_HighGrayLevelRunEmphasis']
 ['wavelet-LLL_glrlm_LongRunEmphasis']
 ['wavelet-LLL_glrlm_LongRunHighGrayLevelEmphasis']
 ['wavelet-LLL_glrlm_RunLengthNonUniformity']
 ['wavelet-LLL_glrlm_RunPercentage']
 ['wavelet-LLL_glrlm_ShortRunEmphasis']
 ['wavelet-LLL_glrlm_ShortRunLowGrayLevelEmphasis']
 ['wavelet-LLL_glszm_GrayLevelNonUniformityNormalized']
 ['wavelet-LLL_glszm_GrayLevelVariance']
 ['wavelet-LLL_glszm_HighGrayLevelZoneEmphasis']
 ['wavelet-LLL_glszm_LargeAreaLowGrayLevelEmphasis']
 ['wavelet-LLL_glszm_SizeZoneNonUniformity']
 ['wavelet-LLL_glszm_ZonePercentage']
 ['wavelet-LLL_ngtdm_Busyness']
 ['wavelet-LLL_ngtdm_Coarseness']
 ['wavelet-LLL_ngtdm_Complexity']
 ['wavelet-LLL_ngtdm_Contrast']
 ['wavelet-LLL_ngtdm_Strength']]

Heatmap of the model in the training samples:


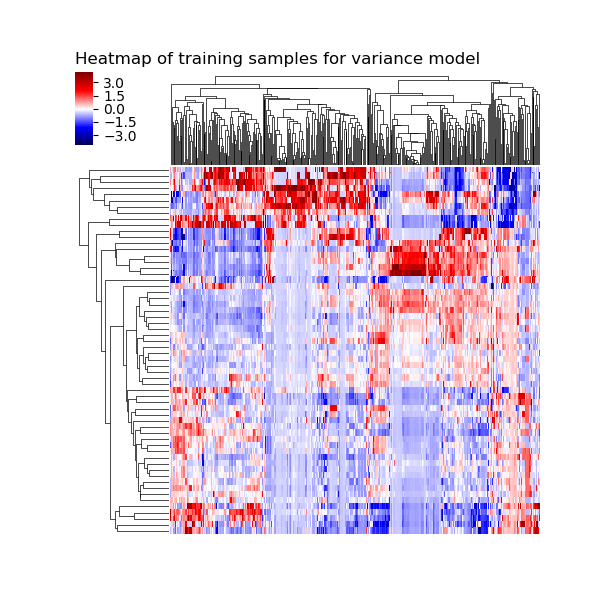


Heatmap of the model in the testing samples:


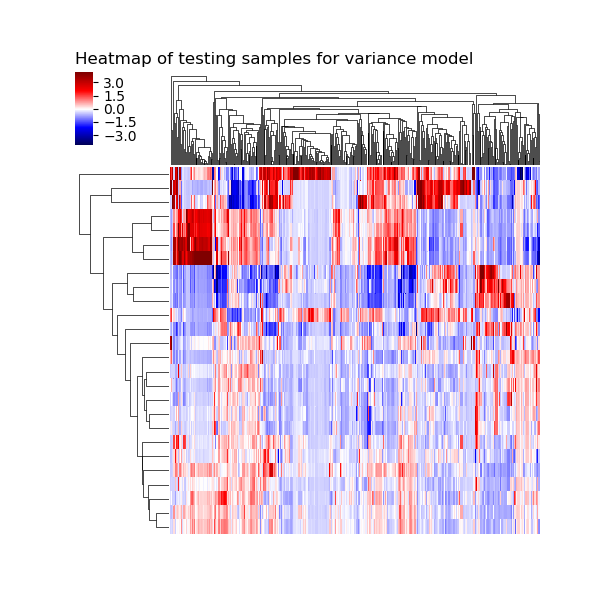


Correlation coefficient figure of the training samples

Correlation coefficient figure of the testing samples

# 7. The method for selecting features: Correlation_xx

parameters setted: {'cutoff': 0.7}
num of remained features: 93
remained features:
[['original_shape_Flatness']
 ['original_firstorder_Maximum']
 ['original_firstorder_Minimum']
 ['original_glcm_ClusterShade']
 ['original_glcm_DifferenceEntropy']
 ['original_glcm_Imc1']
 ['original_glcm_InverseVariance']
 ['original_gldm_LargeDependenceLowGrayLevelEmphasis']
 ['original_glszm_SmallAreaEmphasis']
 ['wavelet-LLH_firstorder_90Percentile']
 ['wavelet-LLH_firstorder_Kurtosis']
 ['wavelet-LLH_firstorder_Median']
 ['wavelet-LLH_firstorder_Skewness']
 ['wavelet-LLH_firstorder_TotalEnergy']
 ['wavelet-LLH_glcm_Correlation']
 ['wavelet-LLH_glcm_Imc1']
 ['wavelet-LLH_gldm_DependenceNonUniformityNormalized']
 ['wavelet-LLH_gldm_SmallDependenceHighGrayLevelEmphasis']
 ['wavelet-LLH_glrlm_LowGrayLevelRunEmphasis']
 ['wavelet-LLH_glszm_LargeAreaHighGrayLevelEmphasis']
 ['wavelet-LLH_glszm_LowGrayLevelZoneEmphasis']
 ['wavelet-LLH_glszm_SizeZoneNonUniformityNormalized']
 ['wavelet-LLH_glszm_SmallAreaHighGrayLevelEmphasis']
 ['wavelet-LLH_ngtdm_Contrast']
 ['wavelet-LHL_firstorder_Kurtosis']
 ['wavelet-LHL_glcm_Contrast']
 ['wavelet-LHL_glcm_MCC']
 ['wavelet-LHL_gldm_LargeDependenceLowGrayLevelEmphasis']
 ['wavelet-LHL_glrlm_LowGrayLevelRunEmphasis']
 ['wavelet-LHL_glrlm_RunEntropy']
 ['wavelet-LHL_glszm_LargeAreaLowGrayLevelEmphasis']
 ['wavelet-LHL_glszm_SmallAreaLowGrayLevelEmphasis']
 ['wavelet-LHL_ngtdm_Complexity']
 ['wavelet-LHL_ngtdm_Strength']
 ['wavelet-LHH_firstorder_Mean']
 ['wavelet-LHH_glcm_ClusterShade']
 ['wavelet-LHH_gldm_DependenceVariance']
 ['wavelet-LHH_gldm_LargeDependenceLowGrayLevelEmphasis']
 ['wavelet-LHH_glszm_SizeZoneNonUniformity']
 ['wavelet-LHH_glszm_SizeZoneNonUniformityNormalized']
 ['wavelet-LHH_glszm_SmallAreaEmphasis']
 ['wavelet-HLL_firstorder_Kurtosis']
 ['wavelet-HLL_firstorder_Skewness']
 ['wavelet-HLL_glcm_ClusterProminence']
 ['wavelet-HLL_glcm_Correlation']
 ['wavelet-HLL_glcm_DifferenceVariance']
 ['wavelet-HLL_glcm_Imc2']
 ['wavelet-HLL_glcm_InverseVariance']
 ['wavelet-HLL_gldm_DependenceEntropy']
 ['wavelet-HLL_glrlm_LongRunHighGrayLevelEmphasis']
 ['wavelet-HLL_glszm_GrayLevelNonUniformityNormalized']
 ['wavelet-HLL_glszm_LargeAreaHighGrayLevelEmphasis']
 ['wavelet-HLL_ngtdm_Complexity']
 ['wavelet-HLL_ngtdm_Strength']
 ['wavelet-HLH_firstorder_Median']
 ['wavelet-HLH_firstorder_Skewness']
 ['wavelet-HLH_glcm_MaximumProbability']
 ['wavelet-HLH_gldm_DependenceEntropy']
 ['wavelet-HLH_gldm_DependenceVariance']
 ['wavelet-HLH_glrlm_LongRunLowGrayLevelEmphasis']
 ['wavelet-HLH_glszm_SmallAreaEmphasis']
 ['wavelet-HHL_firstorder_Mean']
 ['wavelet-HHL_firstorder_Median']
 ['wavelet-HHL_firstorder_Variance']
 ['wavelet-HHL_glcm_Autocorrelation']
 ['wavelet-HHL_glcm_MCC']
 ['wavelet-HHL_gldm_DependenceEntropy']
 ['wavelet-HHL_gldm_DependenceNonUniformityNormalized']
 ['wavelet-HHL_glrlm_LongRunHighGrayLevelEmphasis']
 ['wavelet-HHL_glrlm_LongRunLowGrayLevelEmphasis']
 ['wavelet-HHL_glszm_GrayLevelNonUniformityNormalized']
 ['wavelet-HHL_glszm_LargeAreaLowGrayLevelEmphasis']
 ['wavelet-HHL_glszm_SmallAreaEmphasis']
 ['wavelet-HHL_glszm_SmallAreaLowGrayLevelEmphasis']
 ['wavelet-HHH_firstorder_Kurtosis']
 ['wavelet-HHH_firstorder_Mean']
 ['wavelet-HHH_firstorder_Median']
 ['wavelet-HHH_glcm_Contrast']
 ['wavelet-HHH_glcm_Id']
 ['wavelet-HHH_gldm_DependenceVariance']
 ['wavelet-HHH_gldm_SmallDependenceEmphasis']
 ['wavelet-HHH_gldm_SmallDependenceHighGrayLevelEmphasis']
 ['wavelet-HHH_gldm_SmallDependenceLowGrayLevelEmphasis']
 ['wavelet-HHH_glszm_SizeZoneNonUniformityNormalized']
 ['wavelet-HHH_glszm_SmallAreaEmphasis']
 ['wavelet-HHH_glszm_ZoneVariance']
 ['wavelet-LLL_firstorder_Entropy']
 ['wavelet-LLL_firstorder_Kurtosis']
 ['wavelet-LLL_gldm_LargeDependenceLowGrayLevelEmphasis']
 ['wavelet-LLL_glszm_LargeAreaLowGrayLevelEmphasis']
 ['wavelet-LLL_ngtdm_Busyness']
 ['wavelet-LLL_ngtdm_Complexity']
 ['wavelet-LLL_ngtdm_Contrast']]

Heatmap of the model in the training samples:


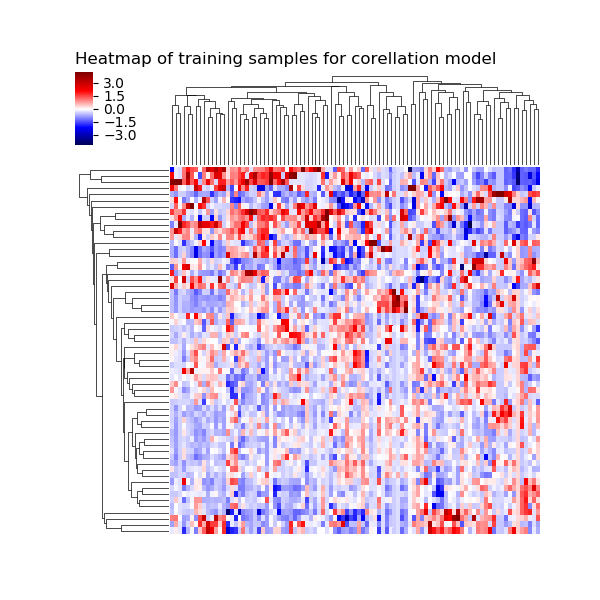


Heatmap of the model in the testing samples:


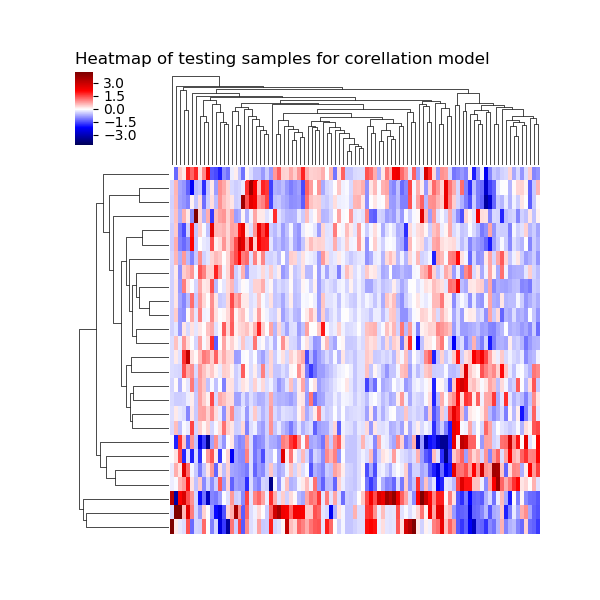


Correlation coefficient figure of the training samples

Correlation coefficient figure of the testing samples

# 8. The method for selecting features: MultiVariate_Logistic

parameters setted: {'P value for threshold in': 0.05, 'P value for threshold out': 0.1}
num of remained features: 4
remained features:
[['wavelet-HHH_glszm_SizeZoneNonUniformityNormalized']
 ['wavelet-LLH_firstorder_TotalEnergy']
 ['wavelet-HHL_glszm_SmallAreaLowGrayLevelEmphasis']
 ['wavelet-HLH_firstorder_Skewness']]

Statistical analysis of logistic multivariate analysis:

| feature | OR | 0.025 | 0.975 | P_value |
| --- | --- | --- | --- | --- |
| const | 0.267 | 0.108 | 0.66 | nan |
| wavelet-HHH_glszm_SizeZoneNonUniformityNormalized | 5.766 | 1.672 | 19.889 | 0.006 |
| wavelet-LLH_firstorder_TotalEnergy | 4.592 | 1.55 | 13.601 | 0.006 |
| wavelet-HHL_glszm_SmallAreaLowGrayLevelEmphasis | 3.147 | 1.319 | 7.512 | 0.01 |
| wavelet-HLH_firstorder_Skewness | 2.703 | 1.076 | 6.792 | 0.034 |

Heatmap of the model in the training samples:


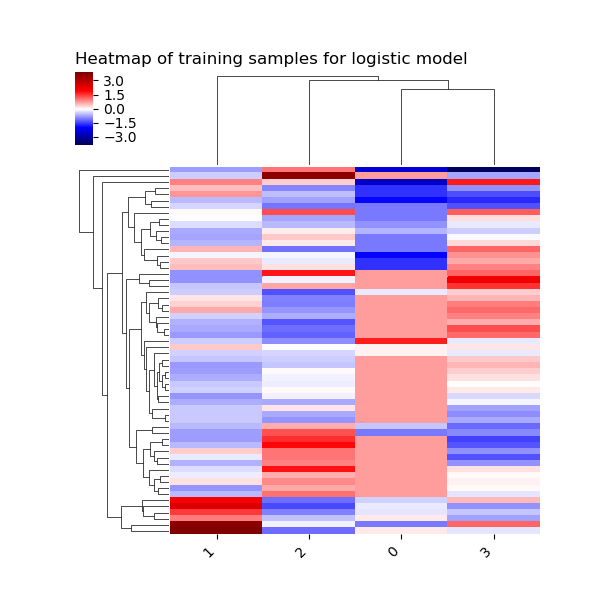


Heatmap of the model in the testing samples:


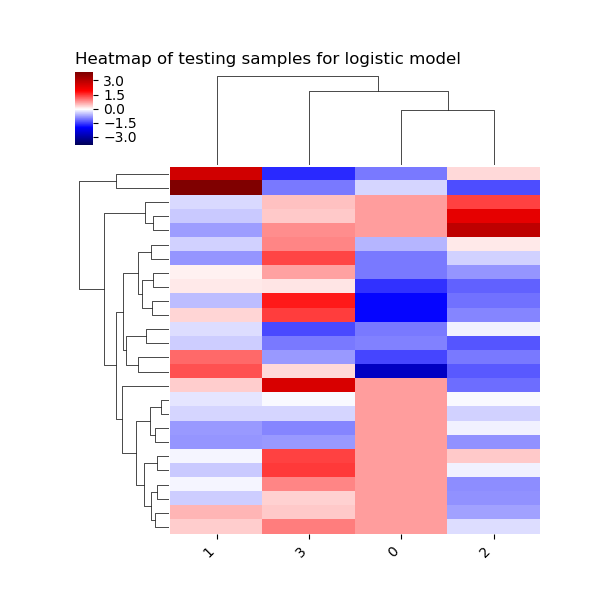


Correlation coefficient figure of the training samples


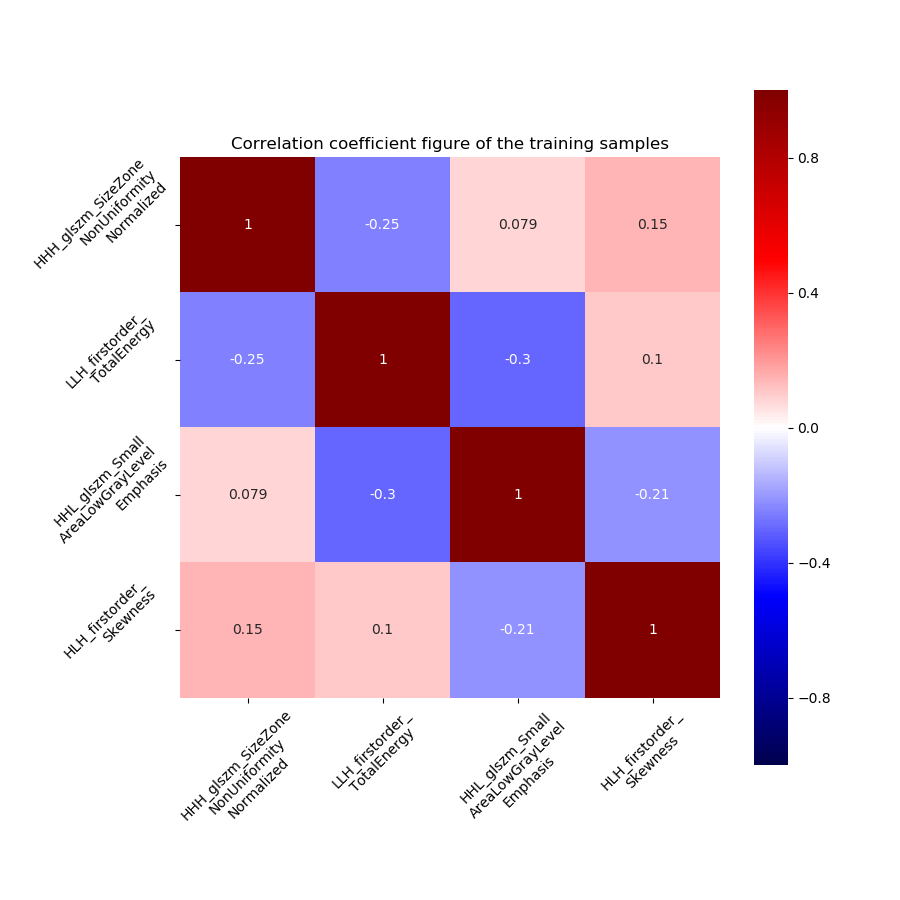


Correlation coefficient figure of the testing samples


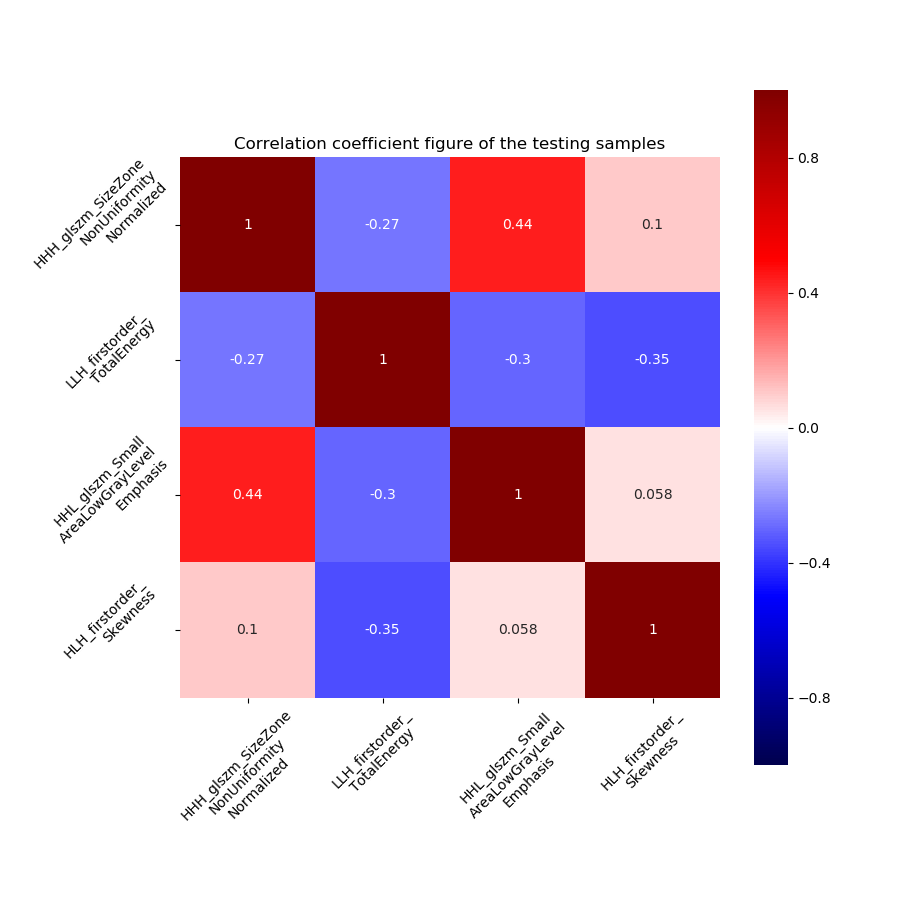


# 9. The best machine learning method: SVM

the mechine learning method: SVM

the parameters selection method and selected parameters:
 {'Method': 'auto', 'C': 95.93742017977509, 'gamma': 0.017078776762856695, 'kernel': 'rbf'}

the separate scores and total mean scores of model in each validation fold:
 {'scores': array([0.75 , 0.75 , 0.75 , 0.58333333, 0.83333333]), 'mean_score': 0.7333333333333334}

evaluation of the SVM model in the training and testing samples:

| Item | Train | Test |
| --- | --- | --- |
| Accuracy | 0.783 | 0.692 |
| f1_score | 0.606 | 0.556 |
| Recall | 0.5 | 0.556 |
| Precision | 0.769 | 0.556 |
| AUC | 0.876 (0.799, 0.946) | 0.719 (0.541, 0.882) |
| Sensitivity | 0.5 | 0.556 |
| Specificity | 0.925 | 0.765 |
| positive prediction | 0.769 | 0.556 |
| negative prediction | 0.787 | 0.765 |
| positive llr | 6.667 | 2.361 |
| negatice llr | 0.541 | 0.581 |

ROC of the SVM model in the training samples:


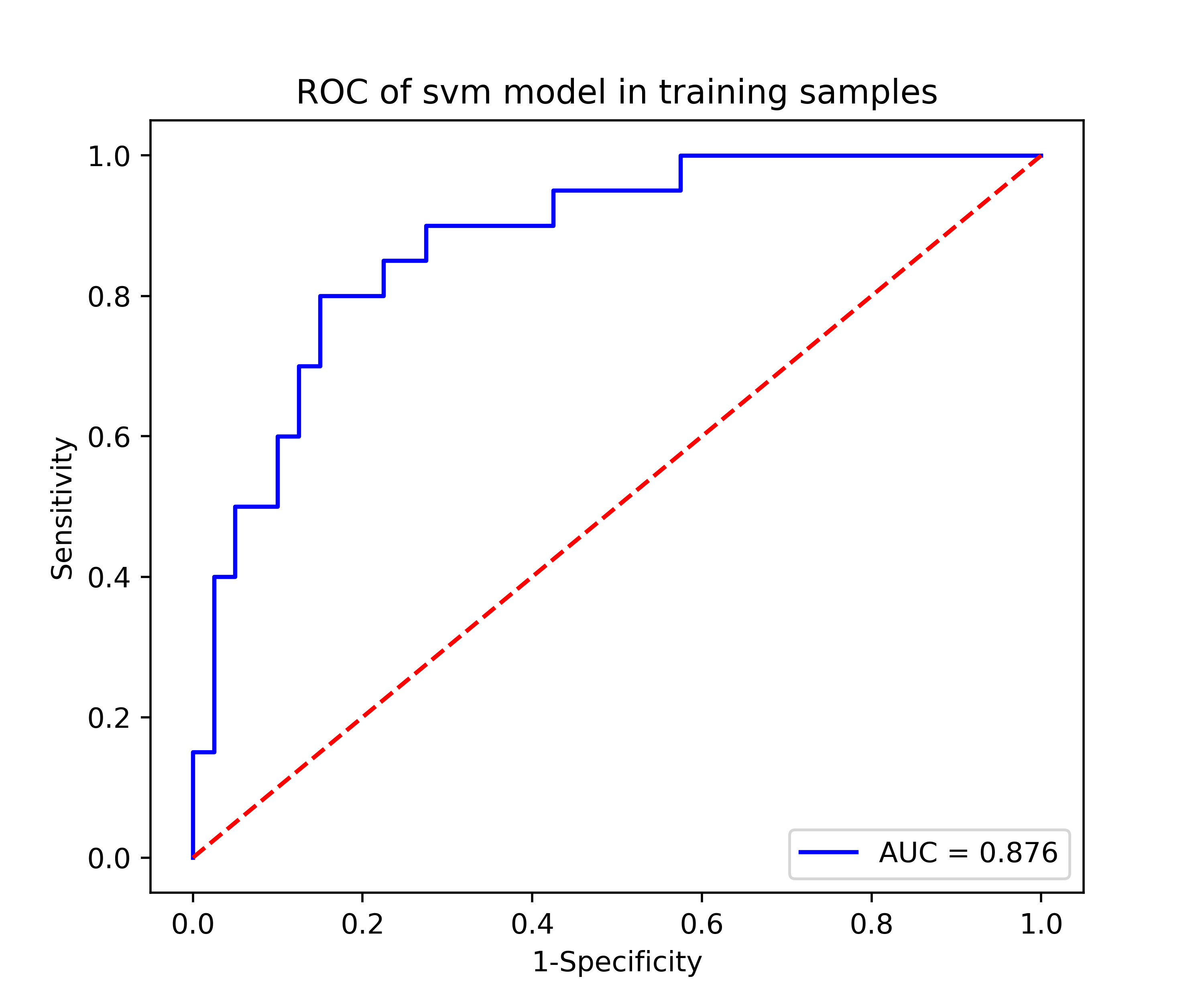


ROC of the SVM model in the testing samples:


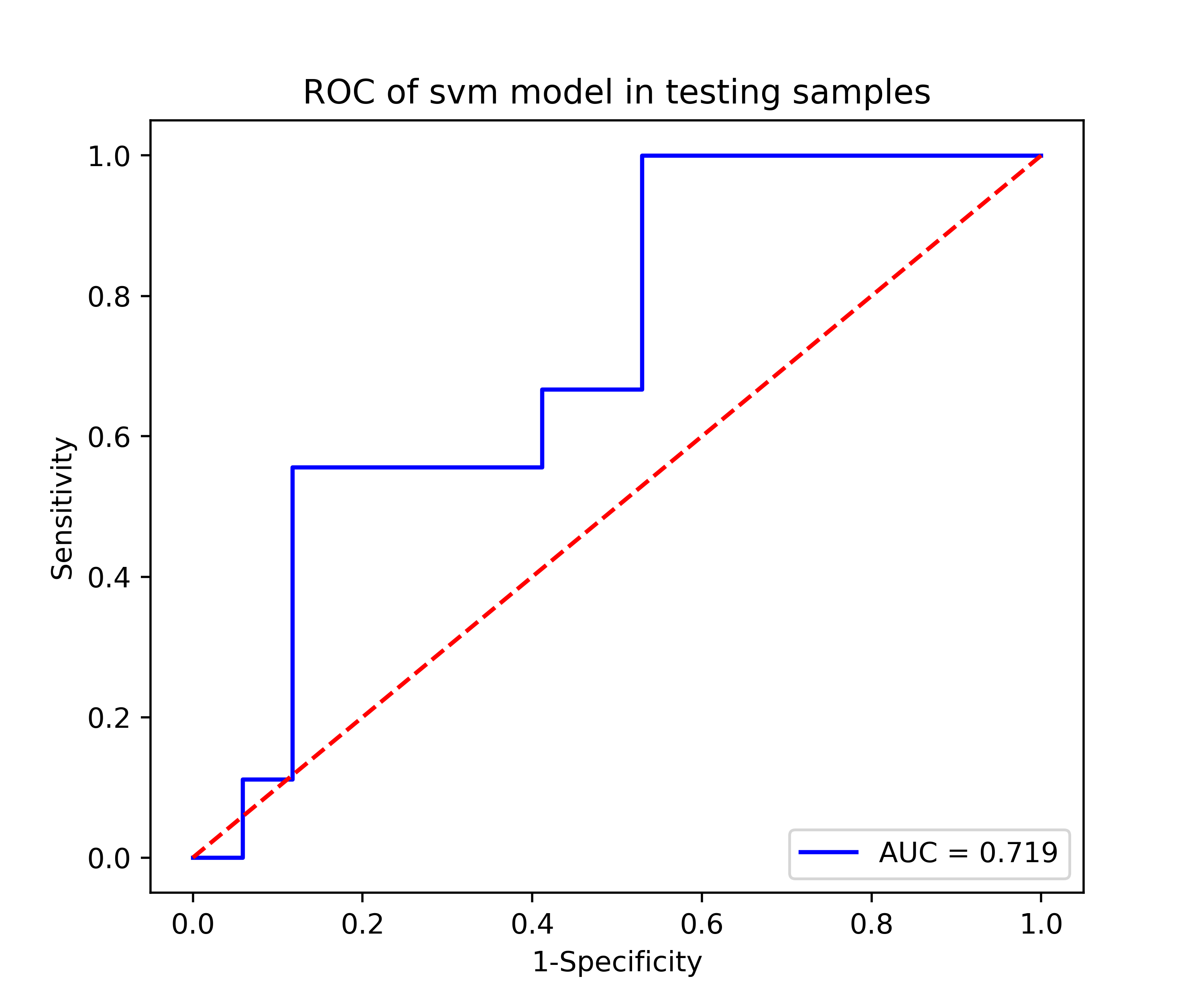


Decision Curve of SVM model in training samples:


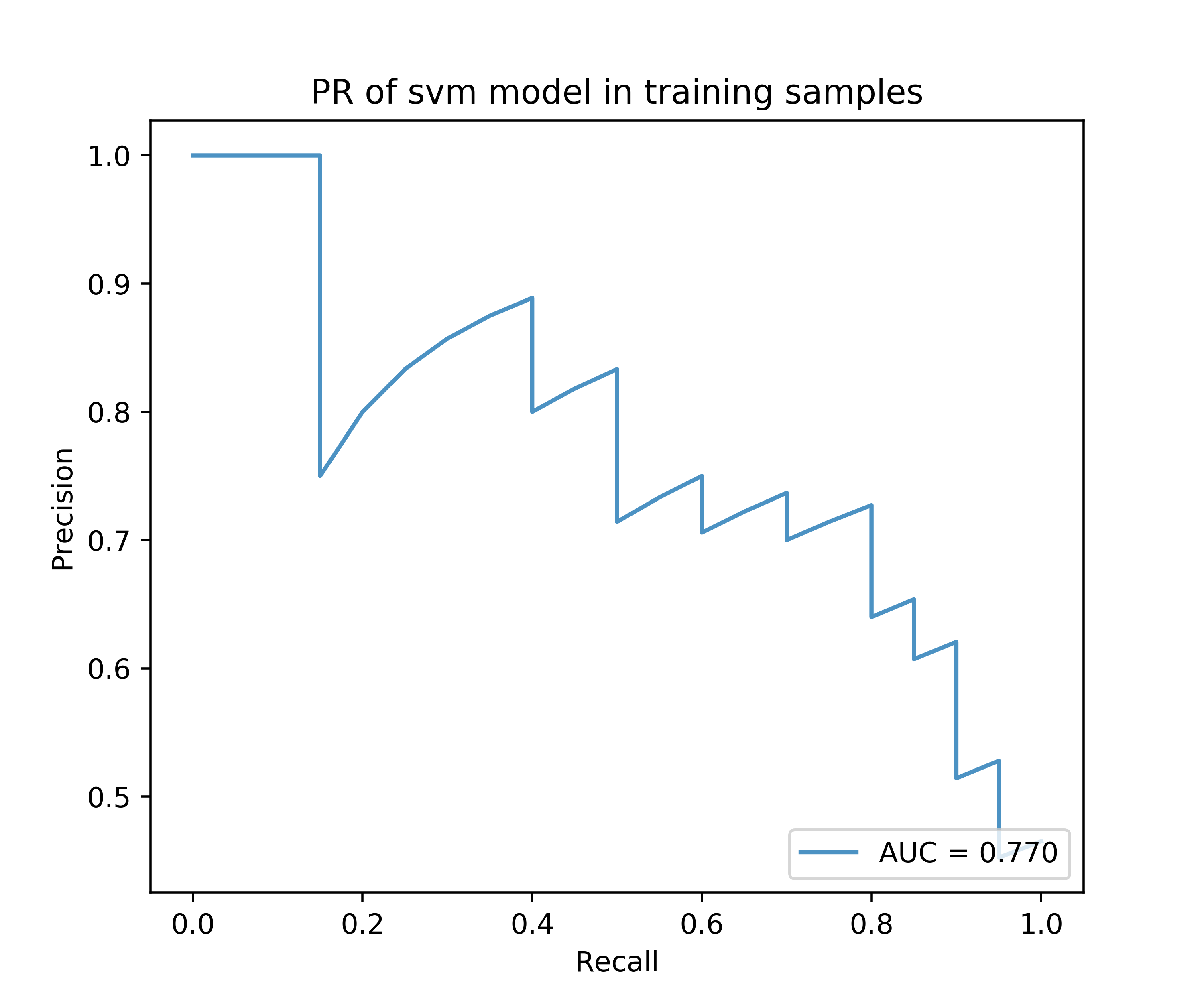


Decision Curve of SVM model in testing samples:


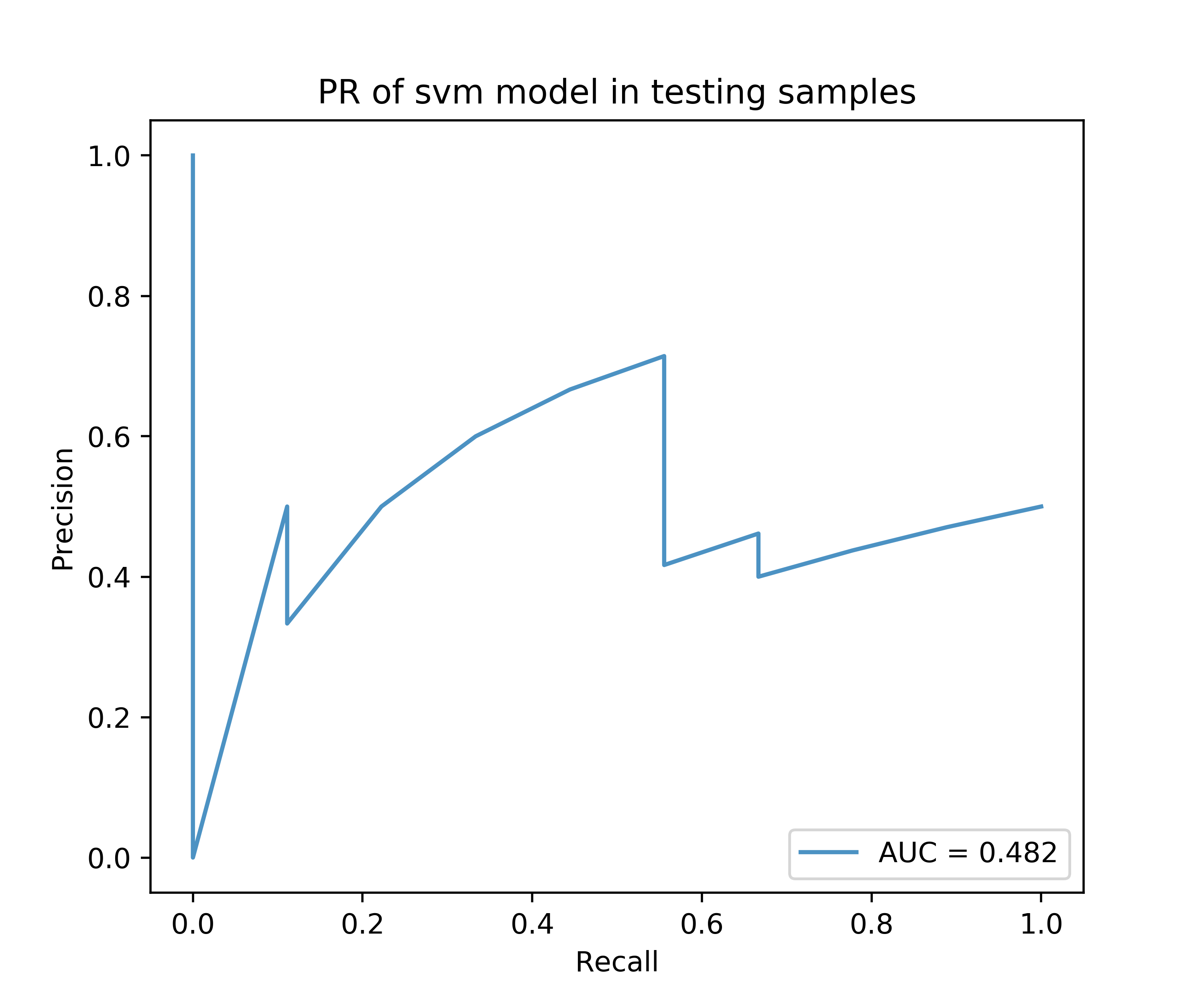


**Radiomics analysis process of naïve Bayesian classification.**

Summary Report

# The summary report recognized the best model from all models your training, and summarized its training process.

As the result shown, the best model was Bayes, its training process including: ['select a path', 'set a seed', 'seperate a data', 'input data', 'standardize data', 'select feature', 'select feature', 'select feature', 'machine_learning']. Detailed information is shown below:

# 1. Data: C:/Users/212768837/Desktop/zhuanyi_86ROI/new/result2.csv

# 2. Random seed: 53

# 3. Seperative rate: 0.7

Seperated report:

|  | Sum | Pos | Neg |
| --- | --- | --- | --- |
| data | 86 | 29 | 57 |
| train | 60 | 20 | 40 |
| test | 26 | 9 | 17 |

# 4. Input data

The method for filling the missing data: Median

The method for dealing with outliers: Median

# 5. The method for standardizing the data: Standardization

# 6. The method for selecting features: Variance

parameters setted: {'threshold': 1.0}
num of remained features: 434
remained features:
[['original_shape_Elongation']
 ['original_shape_Maximum2DDiameterColumn']
 ['original_shape_Maximum2DDiameterSlice']
 ['original_shape_Sphericity']
 ['original_shape_SurfaceVolumeRatio']
 ['original_firstorder_Maximum']
 ['original_firstorder_Mean']
 ['original_firstorder_Median']
 ['original_firstorder_RobustMeanAbsoluteDeviation']
 ['original_firstorder_Variance']
 ['original_glcm_Autocorrelation']
 ['original_glcm_ClusterShade']
 ['original_glcm_Contrast']
 ['original_glcm_DifferenceAverage']
 ['original_glcm_DifferenceVariance']
 ['original_glcm_Id']
 ['original_glcm_Idm']
 ['original_glcm_Idmn']
 ['original_glcm_Imc1']
 ['original_glcm_InverseVariance']
 ['original_glcm_JointAverage']
 ['original_glcm_JointEntropy']
 ['original_glcm_SumAverage']
 ['original_gldm_DependenceEntropy']
 ['original_gldm_DependenceNonUniformity']
 ['original_gldm_DependenceNonUniformityNormalized']
 ['original_gldm_GrayLevelVariance']
 ['original_gldm_HighGrayLevelEmphasis']
 ['original_gldm_LargeDependenceEmphasis']
 ['original_gldm_LargeDependenceLowGrayLevelEmphasis']
 ['original_gldm_SmallDependenceHighGrayLevelEmphasis']
 ['original_glrlm_GrayLevelNonUniformity']
 ['original_glrlm_HighGrayLevelRunEmphasis']
 ['original_glrlm_LongRunEmphasis']
 ['original_glrlm_LongRunHighGrayLevelEmphasis']
 ['original_glrlm_LowGrayLevelRunEmphasis']
 ['original_glrlm_RunEntropy']
 ['original_glrlm_RunLengthNonUniformity']
 ['original_glrlm_ShortRunHighGrayLevelEmphasis']
 ['original_glszm_GrayLevelNonUniformityNormalized']
 ['original_glszm_GrayLevelVariance']
 ['original_glszm_HighGrayLevelZoneEmphasis']
 ['original_glszm_LargeAreaEmphasis']
 ['original_glszm_SizeZoneNonUniformity']
 ['original_glszm_SmallAreaHighGrayLevelEmphasis']
 ['original_glszm_SmallAreaLowGrayLevelEmphasis']
 ['original_glszm_ZoneEntropy']
 ['original_glszm_ZonePercentage']
 ['original_glszm_ZoneVariance']
 ['original_ngtdm_Coarseness']
 ['original_ngtdm_Complexity']
 ['original_ngtdm_Contrast']
 ['wavelet-LLH_firstorder_10Percentile']
 ['wavelet-LLH_firstorder_Entropy']
 ['wavelet-LLH_firstorder_InterquartileRange']
 ['wavelet-LLH_firstorder_Mean']
 ['wavelet-LLH_firstorder_Median']
 ['wavelet-LLH_firstorder_Range']
 ['wavelet-LLH_firstorder_RootMeanSquared']
 ['wavelet-LLH_firstorder_Uniformity']
 ['wavelet-LLH_glcm_ClusterShade']
 ['wavelet-LLH_glcm_ClusterTendency']
 ['wavelet-LLH_glcm_Correlation']
 ['wavelet-LLH_glcm_DifferenceEntropy']
 ['wavelet-LLH_glcm_Id']
 ['wavelet-LLH_glcm_Idmn']
 ['wavelet-LLH_glcm_Idn']
 ['wavelet-LLH_glcm_MCC']
 ['wavelet-LLH_glcm_SumEntropy']
 ['wavelet-LLH_gldm_DependenceNonUniformity']
 ['wavelet-LLH_gldm_DependenceVariance']
 ['wavelet-LLH_gldm_GrayLevelNonUniformity']
 ['wavelet-LLH_gldm_HighGrayLevelEmphasis']
 ['wavelet-LLH_gldm_LargeDependenceLowGrayLevelEmphasis']
 ['wavelet-LLH_gldm_LowGrayLevelEmphasis']
 ['wavelet-LLH_gldm_SmallDependenceEmphasis']
 ['wavelet-LLH_gldm_SmallDependenceHighGrayLevelEmphasis']
 ['wavelet-LLH_gldm_SmallDependenceLowGrayLevelEmphasis']
 ['wavelet-LLH_glrlm_LongRunLowGrayLevelEmphasis']
 ['wavelet-LLH_glrlm_LowGrayLevelRunEmphasis']
 ['wavelet-LLH_glrlm_RunLengthNonUniformityNormalized']
 ['wavelet-LLH_glrlm_RunVariance']
 ['wavelet-LLH_glrlm_ShortRunHighGrayLevelEmphasis']
 ['wavelet-LLH_glszm_GrayLevelVariance']
 ['wavelet-LLH_glszm_LargeAreaHighGrayLevelEmphasis']
 ['wavelet-LLH_glszm_LowGrayLevelZoneEmphasis']
 ['wavelet-LLH_glszm_SizeZoneNonUniformity']
 ['wavelet-LLH_glszm_SmallAreaEmphasis']
 ['wavelet-LLH_glszm_SmallAreaHighGrayLevelEmphasis']
 ['wavelet-LLH_glszm_SmallAreaLowGrayLevelEmphasis']
 ['wavelet-LLH_glszm_ZonePercentage']
 ['wavelet-LLH_glszm_ZoneVariance']
 ['wavelet-LLH_ngtdm_Complexity']
 ['wavelet-LHL_firstorder_10Percentile']
 ['wavelet-LHL_firstorder_90Percentile']
 ['wavelet-LHL_firstorder_Entropy']
 ['wavelet-LHL_firstorder_Maximum']
 ['wavelet-LHL_firstorder_Median']
 ['wavelet-LHL_firstorder_Range']
 ['wavelet-LHL_firstorder_RobustMeanAbsoluteDeviation']
 ['wavelet-LHL_firstorder_RootMeanSquared']
 ['wavelet-LHL_firstorder_Skewness']
 ['wavelet-LHL_firstorder_Uniformity']
 ['wavelet-LHL_firstorder_Variance']
 ['wavelet-LHL_glcm_Autocorrelation']
 ['wavelet-LHL_glcm_ClusterShade']
 ['wavelet-LHL_glcm_ClusterTendency']
 ['wavelet-LHL_glcm_Contrast']
 ['wavelet-LHL_glcm_DifferenceAverage']
 ['wavelet-LHL_glcm_DifferenceEntropy']
 ['wavelet-LHL_glcm_DifferenceVariance']
 ['wavelet-LHL_glcm_Idm']
 ['wavelet-LHL_glcm_Imc1']
 ['wavelet-LHL_glcm_InverseVariance']
 ['wavelet-LHL_glcm_JointEnergy']
 ['wavelet-LHL_glcm_JointEntropy']
 ['wavelet-LHL_glcm_SumEntropy']
 ['wavelet-LHL_glcm_SumSquares']
 ['wavelet-LHL_gldm_DependenceNonUniformityNormalized']
 ['wavelet-LHL_gldm_DependenceVariance']
 ['wavelet-LHL_gldm_GrayLevelNonUniformity']
 ['wavelet-LHL_gldm_SmallDependenceEmphasis']
 ['wavelet-LHL_gldm_SmallDependenceHighGrayLevelEmphasis']
 ['wavelet-LHL_glrlm_GrayLevelVariance']
 ['wavelet-LHL_glrlm_LongRunHighGrayLevelEmphasis']
 ['wavelet-LHL_glrlm_LongRunLowGrayLevelEmphasis']
 ['wavelet-LHL_glrlm_LowGrayLevelRunEmphasis']
 ['wavelet-LHL_glrlm_RunEntropy']
 ['wavelet-LHL_glrlm_RunLengthNonUniformityNormalized']
 ['wavelet-LHL_glrlm_RunPercentage']
 ['wavelet-LHL_glrlm_RunVariance']
 ['wavelet-LHL_glrlm_ShortRunHighGrayLevelEmphasis']
 ['wavelet-LHL_glszm_GrayLevelNonUniformityNormalized']
 ['wavelet-LHL_glszm_GrayLevelVariance']
 ['wavelet-LHL_glszm_HighGrayLevelZoneEmphasis']
 ['wavelet-LHL_glszm_SizeZoneNonUniformity']
 ['wavelet-LHL_glszm_SmallAreaEmphasis']
 ['wavelet-LHL_glszm_SmallAreaHighGrayLevelEmphasis']
 ['wavelet-LHL_glszm_SmallAreaLowGrayLevelEmphasis']
 ['wavelet-LHL_glszm_ZonePercentage']
 ['wavelet-LHL_glszm_ZoneVariance']
 ['wavelet-LHL_ngtdm_Busyness']
 ['wavelet-LHL_ngtdm_Complexity']
 ['wavelet-LHL_ngtdm_Contrast']
 ['wavelet-LHH_firstorder_10Percentile']
 ['wavelet-LHH_firstorder_Maximum']
 ['wavelet-LHH_firstorder_MeanAbsoluteDeviation']
 ['wavelet-LHH_firstorder_Mean']
 ['wavelet-LHH_firstorder_Median']
 ['wavelet-LHH_firstorder_Minimum']
 ['wavelet-LHH_firstorder_Range']
 ['wavelet-LHH_firstorder_RootMeanSquared']
 ['wavelet-LHH_firstorder_Skewness']
 ['wavelet-LHH_firstorder_Variance']
 ['wavelet-LHH_glcm_Autocorrelation']
 ['wavelet-LHH_glcm_ClusterProminence']
 ['wavelet-LHH_glcm_ClusterTendency']
 ['wavelet-LHH_glcm_Contrast']
 ['wavelet-LHH_glcm_Correlation']
 ['wavelet-LHH_glcm_DifferenceAverage']
 ['wavelet-LHH_glcm_Idn']
 ['wavelet-LHH_glcm_JointAverage']
 ['wavelet-LHH_glcm_JointEntropy']
 ['wavelet-LHH_glcm_MaximumProbability']
 ['wavelet-LHH_glcm_SumAverage']
 ['wavelet-LHH_gldm_DependenceEntropy']
 ['wavelet-LHH_gldm_DependenceNonUniformityNormalized']
 ['wavelet-LHH_gldm_GrayLevelNonUniformity']
 ['wavelet-LHH_gldm_GrayLevelVariance']
 ['wavelet-LHH_gldm_LargeDependenceEmphasis']
 ['wavelet-LHH_gldm_LargeDependenceLowGrayLevelEmphasis']
 ['wavelet-LHH_gldm_SmallDependenceHighGrayLevelEmphasis']
 ['wavelet-LHH_gldm_SmallDependenceLowGrayLevelEmphasis']
 ['wavelet-LHH_glrlm_GrayLevelNonUniformity']
 ['wavelet-LHH_glrlm_GrayLevelNonUniformityNormalized']
 ['wavelet-LHH_glrlm_HighGrayLevelRunEmphasis']
 ['wavelet-LHH_glrlm_LongRunLowGrayLevelEmphasis']
 ['wavelet-LHH_glrlm_LowGrayLevelRunEmphasis']
 ['wavelet-LHH_glrlm_RunVariance']
 ['wavelet-LHH_glrlm_ShortRunEmphasis']
 ['wavelet-LHH_glrlm_ShortRunHighGrayLevelEmphasis']
 ['wavelet-LHH_glrlm_ShortRunLowGrayLevelEmphasis']
 ['wavelet-LHH_glszm_GrayLevelNonUniformity']
 ['wavelet-LHH_glszm_GrayLevelNonUniformityNormalized']
 ['wavelet-LHH_glszm_HighGrayLevelZoneEmphasis']
 ['wavelet-LHH_glszm_LargeAreaEmphasis']
 ['wavelet-LHH_glszm_LargeAreaHighGrayLevelEmphasis']
 ['wavelet-LHH_glszm_LowGrayLevelZoneEmphasis']
 ['wavelet-LHH_glszm_SizeZoneNonUniformity']
 ['wavelet-LHH_glszm_SmallAreaEmphasis']
 ['wavelet-LHH_glszm_SmallAreaHighGrayLevelEmphasis']
 ['wavelet-LHH_glszm_SmallAreaLowGrayLevelEmphasis']
 ['wavelet-LHH_ngtdm_Complexity']
 ['wavelet-LHH_ngtdm_Strength']
 ['wavelet-HLL_firstorder_InterquartileRange']
 ['wavelet-HLL_firstorder_MeanAbsoluteDeviation']
 ['wavelet-HLL_firstorder_Minimum']
 ['wavelet-HLL_firstorder_Range']
 ['wavelet-HLL_firstorder_RobustMeanAbsoluteDeviation']
 ['wavelet-HLL_firstorder_RootMeanSquared']
 ['wavelet-HLL_firstorder_Uniformity']
 ['wavelet-HLL_firstorder_Variance']
 ['wavelet-HLL_glcm_Autocorrelation']
 ['wavelet-HLL_glcm_ClusterTendency']
 ['wavelet-HLL_glcm_Contrast']
 ['wavelet-HLL_glcm_Correlation']
 ['wavelet-HLL_glcm_DifferenceAverage']
 ['wavelet-HLL_glcm_DifferenceEntropy']
 ['wavelet-HLL_glcm_DifferenceVariance']
 ['wavelet-HLL_glcm_Id']
 ['wavelet-HLL_glcm_Idm']
 ['wavelet-HLL_glcm_Idmn']
 ['wavelet-HLL_glcm_InverseVariance']
 ['wavelet-HLL_glcm_SumEntropy']
 ['wavelet-HLL_glcm_SumSquares']
 ['wavelet-HLL_gldm_DependenceEntropy']
 ['wavelet-HLL_gldm_DependenceNonUniformityNormalized']
 ['wavelet-HLL_gldm_GrayLevelNonUniformity']
 ['wavelet-HLL_gldm_GrayLevelVariance']
 ['wavelet-HLL_gldm_LargeDependenceEmphasis']
 ['wavelet-HLL_gldm_LargeDependenceLowGrayLevelEmphasis']
 ['wavelet-HLL_gldm_LowGrayLevelEmphasis']
 ['wavelet-HLL_gldm_SmallDependenceEmphasis']
 ['wavelet-HLL_gldm_SmallDependenceHighGrayLevelEmphasis']
 ['wavelet-HLL_glrlm_GrayLevelNonUniformity']
 ['wavelet-HLL_glrlm_GrayLevelNonUniformityNormalized']
 ['wavelet-HLL_glrlm_HighGrayLevelRunEmphasis']
 ['wavelet-HLL_glrlm_LongRunEmphasis']
 ['wavelet-HLL_glrlm_LongRunLowGrayLevelEmphasis']
 ['wavelet-HLL_glrlm_RunLengthNonUniformity']
 ['wavelet-HLL_glrlm_RunPercentage']
 ['wavelet-HLL_glrlm_RunVariance']
 ['wavelet-HLL_glrlm_ShortRunEmphasis']
 ['wavelet-HLL_glszm_GrayLevelNonUniformityNormalized']
 ['wavelet-HLL_glszm_SmallAreaEmphasis']
 ['wavelet-HLL_glszm_SmallAreaLowGrayLevelEmphasis']
 ['wavelet-HLL_ngtdm_Strength']
 ['wavelet-HLH_firstorder_10Percentile']
 ['wavelet-HLH_firstorder_Energy']
 ['wavelet-HLH_firstorder_Kurtosis']
 ['wavelet-HLH_firstorder_MeanAbsoluteDeviation']
 ['wavelet-HLH_firstorder_Mean']
 ['wavelet-HLH_firstorder_Median']
 ['wavelet-HLH_firstorder_RobustMeanAbsoluteDeviation']
 ['wavelet-HLH_firstorder_RootMeanSquared']
 ['wavelet-HLH_firstorder_Skewness']
 ['wavelet-HLH_firstorder_TotalEnergy']
 ['wavelet-HLH_firstorder_Uniformity']
 ['wavelet-HLH_firstorder_Variance']
 ['wavelet-HLH_glcm_Autocorrelation']
 ['wavelet-HLH_glcm_ClusterTendency']
 ['wavelet-HLH_glcm_Contrast']
 ['wavelet-HLH_glcm_Correlation']
 ['wavelet-HLH_glcm_DifferenceVariance']
 ['wavelet-HLH_glcm_Id']
 ['wavelet-HLH_glcm_Idm']
 ['wavelet-HLH_glcm_Idn']
 ['wavelet-HLH_glcm_Imc1']
 ['wavelet-HLH_glcm_InverseVariance']
 ['wavelet-HLH_glcm_SumEntropy']
 ['wavelet-HLH_gldm_DependenceNonUniformity']
 ['wavelet-HLH_gldm_DependenceNonUniformityNormalized']
 ['wavelet-HLH_gldm_DependenceVariance']
 ['wavelet-HLH_gldm_HighGrayLevelEmphasis']
 ['wavelet-HLH_gldm_LargeDependenceEmphasis']
 ['wavelet-HLH_gldm_LargeDependenceHighGrayLevelEmphasis']
 ['wavelet-HLH_gldm_LowGrayLevelEmphasis']
 ['wavelet-HLH_gldm_SmallDependenceEmphasis']
 ['wavelet-HLH_glrlm_GrayLevelNonUniformity']
 ['wavelet-HLH_glrlm_GrayLevelNonUniformityNormalized']
 ['wavelet-HLH_glrlm_HighGrayLevelRunEmphasis']
 ['wavelet-HLH_glrlm_LongRunEmphasis']
 ['wavelet-HLH_glrlm_LongRunLowGrayLevelEmphasis']
 ['wavelet-HLH_glrlm_RunEntropy']
 ['wavelet-HLH_glrlm_RunLengthNonUniformity']
 ['wavelet-HLH_glrlm_RunLengthNonUniformityNormalized']
 ['wavelet-HLH_glrlm_RunPercentage']
 ['wavelet-HLH_glrlm_ShortRunEmphasis']
 ['wavelet-HLH_glrlm_ShortRunHighGrayLevelEmphasis']
 ['wavelet-HLH_glrlm_ShortRunLowGrayLevelEmphasis']
 ['wavelet-HLH_glszm_GrayLevelNonUniformity']
 ['wavelet-HLH_glszm_GrayLevelVariance']
 ['wavelet-HLH_glszm_HighGrayLevelZoneEmphasis']
 ['wavelet-HLH_glszm_LargeAreaEmphasis']
 ['wavelet-HLH_glszm_LargeAreaHighGrayLevelEmphasis']
 ['wavelet-HLH_glszm_LowGrayLevelZoneEmphasis']
 ['wavelet-HLH_glszm_SizeZoneNonUniformity']
 ['wavelet-HLH_glszm_SizeZoneNonUniformityNormalized']
 ['wavelet-HLH_glszm_SmallAreaHighGrayLevelEmphasis']
 ['wavelet-HLH_glszm_SmallAreaLowGrayLevelEmphasis']
 ['wavelet-HLH_glszm_ZoneVariance']
 ['wavelet-HHL_firstorder_InterquartileRange']
 ['wavelet-HHL_firstorder_Kurtosis']
 ['wavelet-HHL_firstorder_Maximum']
 ['wavelet-HHL_firstorder_Median']
 ['wavelet-HHL_firstorder_Minimum']
 ['wavelet-HHL_firstorder_RobustMeanAbsoluteDeviation']
 ['wavelet-HHL_firstorder_Skewness']
 ['wavelet-HHL_firstorder_Uniformity']
 ['wavelet-HHL_firstorder_Variance']
 ['wavelet-HHL_glcm_Autocorrelation']
 ['wavelet-HHL_glcm_ClusterShade']
 ['wavelet-HHL_glcm_ClusterTendency']
 ['wavelet-HHL_glcm_Correlation']
 ['wavelet-HHL_glcm_DifferenceEntropy']
 ['wavelet-HHL_glcm_DifferenceVariance']
 ['wavelet-HHL_glcm_Id']
 ['wavelet-HHL_glcm_Imc1']
 ['wavelet-HHL_glcm_InverseVariance']
 ['wavelet-HHL_glcm_JointAverage']
 ['wavelet-HHL_glcm_JointEntropy']
 ['wavelet-HHL_glcm_MCC']
 ['wavelet-HHL_glcm_MaximumProbability']
 ['wavelet-HHL_glcm_SumAverage']
 ['wavelet-HHL_glcm_SumEntropy']
 ['wavelet-HHL_glcm_SumSquares']
 ['wavelet-HHL_gldm_DependenceEntropy']
 ['wavelet-HHL_gldm_DependenceNonUniformity']
 ['wavelet-HHL_gldm_DependenceNonUniformityNormalized']
 ['wavelet-HHL_gldm_LargeDependenceEmphasis']
 ['wavelet-HHL_gldm_LargeDependenceHighGrayLevelEmphasis']
 ['wavelet-HHL_gldm_LowGrayLevelEmphasis']
 ['wavelet-HHL_glrlm_LongRunHighGrayLevelEmphasis']
 ['wavelet-HHL_glrlm_LongRunLowGrayLevelEmphasis']
 ['wavelet-HHL_glrlm_RunLengthNonUniformityNormalized']
 ['wavelet-HHL_glrlm_ShortRunEmphasis']
 ['wavelet-HHL_glrlm_ShortRunLowGrayLevelEmphasis']
 ['wavelet-HHL_glszm_GrayLevelNonUniformity']
 ['wavelet-HHL_glszm_HighGrayLevelZoneEmphasis']
 ['wavelet-HHL_glszm_LowGrayLevelZoneEmphasis']
 ['wavelet-HHL_glszm_SizeZoneNonUniformity']
 ['wavelet-HHL_glszm_SizeZoneNonUniformityNormalized']
 ['wavelet-HHL_glszm_SmallAreaHighGrayLevelEmphasis']
 ['wavelet-HHL_glszm_SmallAreaLowGrayLevelEmphasis']
 ['wavelet-HHL_glszm_ZoneEntropy']
 ['wavelet-HHL_glszm_ZoneVariance']
 ['wavelet-HHL_ngtdm_Busyness']
 ['wavelet-HHL_ngtdm_Complexity']
 ['wavelet-HHL_ngtdm_Contrast']
 ['wavelet-HHL_ngtdm_Strength']
 ['wavelet-HHH_firstorder_10Percentile']
 ['wavelet-HHH_firstorder_Entropy']
 ['wavelet-HHH_firstorder_InterquartileRange']
 ['wavelet-HHH_firstorder_Kurtosis']
 ['wavelet-HHH_firstorder_Maximum']
 ['wavelet-HHH_firstorder_Mean']
 ['wavelet-HHH_firstorder_Range']
 ['wavelet-HHH_firstorder_Uniformity']
 ['wavelet-HHH_firstorder_Variance']
 ['wavelet-HHH_glcm_Autocorrelation']
 ['wavelet-HHH_glcm_ClusterProminence']
 ['wavelet-HHH_glcm_ClusterShade']
 ['wavelet-HHH_glcm_ClusterTendency']
 ['wavelet-HHH_glcm_DifferenceEntropy']
 ['wavelet-HHH_glcm_DifferenceVariance']
 ['wavelet-HHH_glcm_Id']
 ['wavelet-HHH_glcm_Idm']
 ['wavelet-HHH_glcm_Idn']
 ['wavelet-HHH_glcm_Imc1']
 ['wavelet-HHH_glcm_Imc2']
 ['wavelet-HHH_glcm_InverseVariance']
 ['wavelet-HHH_glcm_SumEntropy']
 ['wavelet-HHH_glcm_SumSquares']
 ['wavelet-HHH_gldm_DependenceEntropy']
 ['wavelet-HHH_gldm_GrayLevelVariance']
 ['wavelet-HHH_gldm_HighGrayLevelEmphasis']
 ['wavelet-HHH_gldm_LargeDependenceHighGrayLevelEmphasis']
 ['wavelet-HHH_gldm_LowGrayLevelEmphasis']
 ['wavelet-HHH_gldm_SmallDependenceLowGrayLevelEmphasis']
 ['wavelet-HHH_glrlm_GrayLevelNonUniformity']
 ['wavelet-HHH_glrlm_GrayLevelVariance']
 ['wavelet-HHH_glrlm_LongRunEmphasis']
 ['wavelet-HHH_glrlm_LongRunHighGrayLevelEmphasis']
 ['wavelet-HHH_glrlm_LongRunLowGrayLevelEmphasis']
 ['wavelet-HHH_glrlm_RunLengthNonUniformityNormalized']
 ['wavelet-HHH_glrlm_RunVariance']
 ['wavelet-HHH_glrlm_ShortRunEmphasis']
 ['wavelet-HHH_glrlm_ShortRunHighGrayLevelEmphasis']
 ['wavelet-HHH_glrlm_ShortRunLowGrayLevelEmphasis']
 ['wavelet-HHH_glszm_GrayLevelNonUniformity']
 ['wavelet-HHH_glszm_HighGrayLevelZoneEmphasis']
 ['wavelet-HHH_glszm_LargeAreaHighGrayLevelEmphasis']
 ['wavelet-HHH_glszm_LargeAreaLowGrayLevelEmphasis']
 ['wavelet-HHH_glszm_SizeZoneNonUniformity']
 ['wavelet-HHH_glszm_SmallAreaHighGrayLevelEmphasis']
 ['wavelet-HHH_glszm_SmallAreaLowGrayLevelEmphasis']
 ['wavelet-HHH_glszm_ZoneEntropy']
 ['wavelet-HHH_glszm_ZonePercentage']
 ['wavelet-HHH_glszm_ZoneVariance']
 ['wavelet-HHH_ngtdm_Busyness']
 ['wavelet-HHH_ngtdm_Coarseness']
 ['wavelet-HHH_ngtdm_Contrast']
 ['wavelet-HHH_ngtdm_Strength']
 ['wavelet-LLL_firstorder_90Percentile']
 ['wavelet-LLL_firstorder_Energy']
 ['wavelet-LLL_firstorder_Entropy']
 ['wavelet-LLL_firstorder_Maximum']
 ['wavelet-LLL_firstorder_MeanAbsoluteDeviation']
 ['wavelet-LLL_firstorder_Median']
 ['wavelet-LLL_firstorder_RobustMeanAbsoluteDeviation']
 ['wavelet-LLL_firstorder_RootMeanSquared']
 ['wavelet-LLL_firstorder_TotalEnergy']
 ['wavelet-LLL_firstorder_Uniformity']
 ['wavelet-LLL_firstorder_Variance']
 ['wavelet-LLL_glcm_ClusterProminence']
 ['wavelet-LLL_glcm_ClusterShade']
 ['wavelet-LLL_glcm_DifferenceEntropy']
 ['wavelet-LLL_glcm_Imc1']
 ['wavelet-LLL_glcm_SumSquares']
 ['wavelet-LLL_gldm_DependenceEntropy']
 ['wavelet-LLL_gldm_DependenceNonUniformity']
 ['wavelet-LLL_gldm_DependenceNonUniformityNormalized']
 ['wavelet-LLL_gldm_GrayLevelNonUniformity']
 ['wavelet-LLL_gldm_HighGrayLevelEmphasis']
 ['wavelet-LLL_gldm_LowGrayLevelEmphasis']
 ['wavelet-LLL_gldm_SmallDependenceEmphasis']
 ['wavelet-LLL_gldm_SmallDependenceHighGrayLevelEmphasis']
 ['wavelet-LLL_gldm_SmallDependenceLowGrayLevelEmphasis']
 ['wavelet-LLL_glrlm_GrayLevelNonUniformity']
 ['wavelet-LLL_glrlm_LongRunLowGrayLevelEmphasis']
 ['wavelet-LLL_glrlm_LowGrayLevelRunEmphasis']
 ['wavelet-LLL_glrlm_RunLengthNonUniformity']
 ['wavelet-LLL_glrlm_RunLengthNonUniformityNormalized']
 ['wavelet-LLL_glrlm_RunVariance']
 ['wavelet-LLL_glrlm_ShortRunEmphasis']
 ['wavelet-LLL_glrlm_ShortRunHighGrayLevelEmphasis']
 ['wavelet-LLL_glszm_GrayLevelNonUniformity']
 ['wavelet-LLL_glszm_GrayLevelVariance']
 ['wavelet-LLL_glszm_HighGrayLevelZoneEmphasis']
 ['wavelet-LLL_glszm_LargeAreaHighGrayLevelEmphasis']
 ['wavelet-LLL_glszm_LowGrayLevelZoneEmphasis']
 ['wavelet-LLL_glszm_SmallAreaEmphasis']
 ['wavelet-LLL_glszm_SmallAreaHighGrayLevelEmphasis']
 ['wavelet-LLL_glszm_ZoneVariance']]

Heatmap of the model in the training samples:


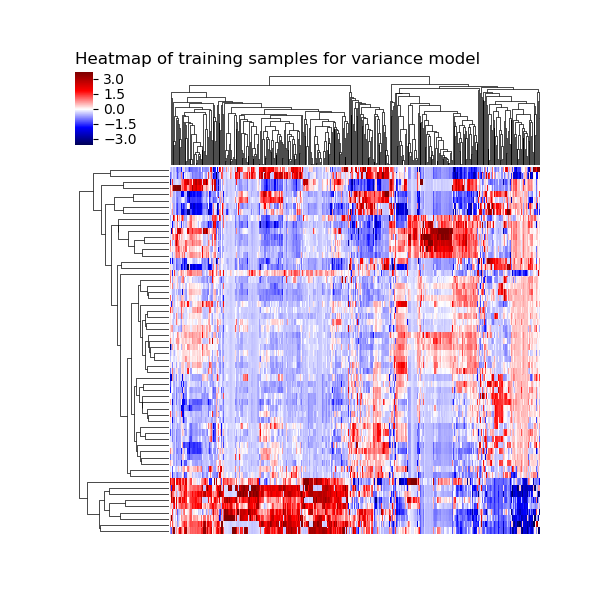


Heatmap of the model in the testing samples:


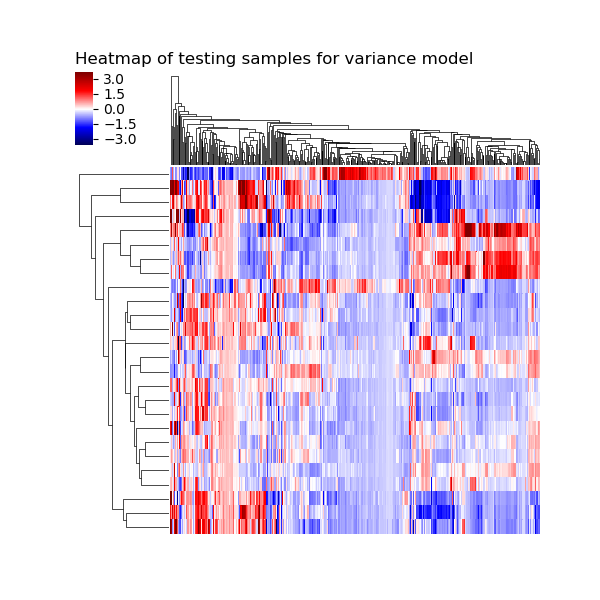


Correlation coefficient figure of the training samples

Correlation coefficient figure of the testing samples

# 7. The method for selecting features: Correlation_xx

parameters setted: {'cutoff': 0.7}
num of remained features: 87
remained features:
[['original_shape_Elongation']
 ['original_shape_Sphericity']
 ['original_glcm_Imc1']
 ['original_glcm_InverseVariance']
 ['original_gldm_LargeDependenceLowGrayLevelEmphasis']
 ['original_glszm_GrayLevelNonUniformityNormalized']
 ['original_glszm_GrayLevelVariance']
 ['original_glszm_SmallAreaLowGrayLevelEmphasis']
 ['wavelet-LLH_firstorder_Median']
 ['wavelet-LLH_glcm_ClusterShade']
 ['wavelet-LLH_glcm_Correlation']
 ['wavelet-LLH_glcm_MCC']
 ['wavelet-LLH_gldm_DependenceVariance']
 ['wavelet-LLH_gldm_HighGrayLevelEmphasis']
 ['wavelet-LLH_gldm_LowGrayLevelEmphasis']
 ['wavelet-LLH_glrlm_LongRunLowGrayLevelEmphasis']
 ['wavelet-LLH_glszm_LargeAreaHighGrayLevelEmphasis']
 ['wavelet-LLH_glszm_SmallAreaLowGrayLevelEmphasis']
 ['wavelet-LHL_firstorder_90Percentile']
 ['wavelet-LHL_firstorder_Median']
 ['wavelet-LHL_firstorder_Skewness']
 ['wavelet-LHL_glcm_ClusterShade']
 ['wavelet-LHL_glcm_Imc1']
 ['wavelet-LHL_glrlm_LowGrayLevelRunEmphasis']
 ['wavelet-LHL_glszm_SmallAreaLowGrayLevelEmphasis']
 ['wavelet-LHH_firstorder_Mean']
 ['wavelet-LHH_firstorder_Median']
 ['wavelet-LHH_firstorder_Skewness']
 ['wavelet-LHH_glcm_Contrast']
 ['wavelet-LHH_glcm_Correlation']
 ['wavelet-LHH_gldm_DependenceNonUniformityNormalized']
 ['wavelet-LHH_gldm_SmallDependenceLowGrayLevelEmphasis']
 ['wavelet-LHH_glszm_SmallAreaEmphasis']
 ['wavelet-LHH_glszm_SmallAreaLowGrayLevelEmphasis']
 ['wavelet-HLL_glcm_Correlation']
 ['wavelet-HLL_glcm_DifferenceVariance']
 ['wavelet-HLL_gldm_DependenceEntropy']
 ['wavelet-HLL_gldm_LargeDependenceLowGrayLevelEmphasis']
 ['wavelet-HLL_glrlm_GrayLevelNonUniformityNormalized']
 ['wavelet-HLL_glszm_GrayLevelNonUniformityNormalized']
 ['wavelet-HLL_glszm_SmallAreaEmphasis']
 ['wavelet-HLL_glszm_SmallAreaLowGrayLevelEmphasis']
 ['wavelet-HLL_ngtdm_Strength']
 ['wavelet-HLH_firstorder_Median']
 ['wavelet-HLH_firstorder_Skewness']
 ['wavelet-HLH_glcm_Correlation']
 ['wavelet-HLH_glcm_Imc1']
 ['wavelet-HLH_gldm_DependenceNonUniformityNormalized']
 ['wavelet-HLH_gldm_SmallDependenceEmphasis']
 ['wavelet-HLH_glrlm_LongRunLowGrayLevelEmphasis']
 ['wavelet-HLH_glszm_SizeZoneNonUniformityNormalized']
 ['wavelet-HLH_glszm_SmallAreaLowGrayLevelEmphasis']
 ['wavelet-HHL_firstorder_Median']
 ['wavelet-HHL_firstorder_Skewness']
 ['wavelet-HHL_glcm_ClusterShade']
 ['wavelet-HHL_glcm_Correlation']
 ['wavelet-HHL_glcm_Imc1']
 ['wavelet-HHL_glcm_InverseVariance']
 ['wavelet-HHL_glcm_MCC']
 ['wavelet-HHL_glcm_MaximumProbability']
 ['wavelet-HHL_gldm_DependenceEntropy']
 ['wavelet-HHL_gldm_DependenceNonUniformityNormalized']
 ['wavelet-HHL_gldm_LargeDependenceHighGrayLevelEmphasis']
 ['wavelet-HHL_glrlm_LongRunHighGrayLevelEmphasis']
 ['wavelet-HHL_glrlm_LongRunLowGrayLevelEmphasis']
 ['wavelet-HHL_glszm_SizeZoneNonUniformityNormalized']
 ['wavelet-HHL_glszm_SmallAreaLowGrayLevelEmphasis']
 ['wavelet-HHL_ngtdm_Contrast']
 ['wavelet-HHL_ngtdm_Strength']
 ['wavelet-HHH_firstorder_Kurtosis']
 ['wavelet-HHH_firstorder_Mean']
 ['wavelet-HHH_glcm_ClusterProminence']
 ['wavelet-HHH_glcm_ClusterShade']
 ['wavelet-HHH_glcm_Idm']
 ['wavelet-HHH_glcm_Imc1']
 ['wavelet-HHH_glcm_InverseVariance']
 ['wavelet-HHH_glcm_SumSquares']
 ['wavelet-HHH_gldm_SmallDependenceLowGrayLevelEmphasis']
 ['wavelet-HHH_glszm_SizeZoneNonUniformity']
 ['wavelet-HHH_glszm_SmallAreaLowGrayLevelEmphasis']
 ['wavelet-HHH_glszm_ZonePercentage']
 ['wavelet-HHH_glszm_ZoneVariance']
 ['wavelet-HHH_ngtdm_Strength']
 ['wavelet-LLL_firstorder_Entropy']
 ['wavelet-LLL_glcm_DifferenceEntropy']
 ['wavelet-LLL_glcm_Imc1']
 ['wavelet-LLL_glszm_ZoneVariance']]

Heatmap of the model in the training samples:


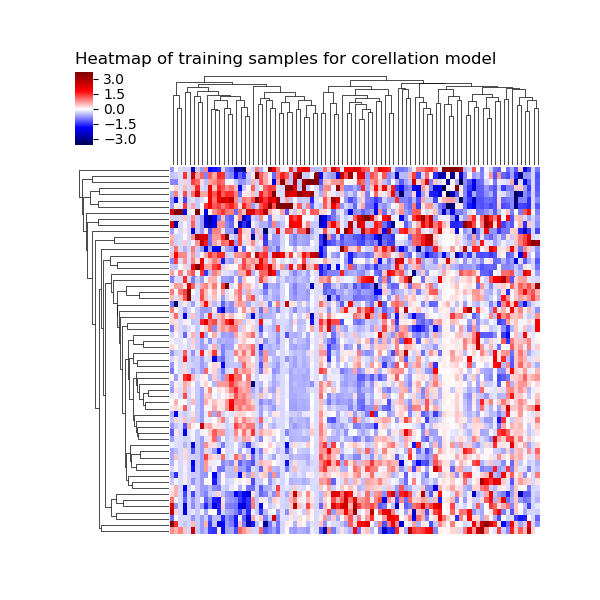


Heatmap of the model in the testing samples:


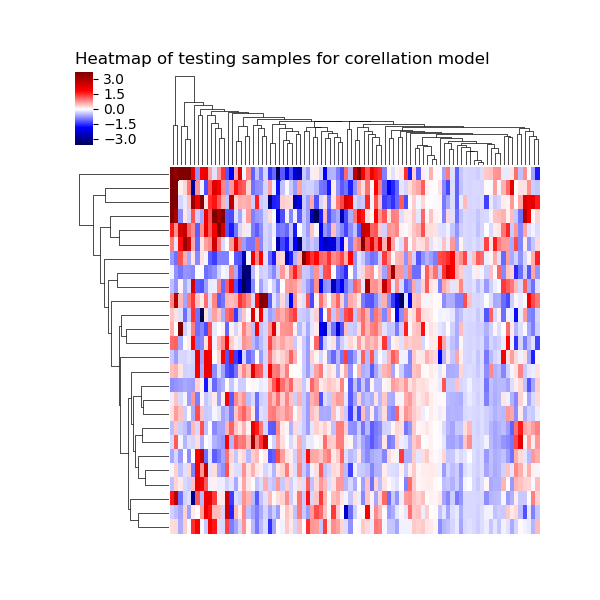


Correlation coefficient figure of the training samples

Correlation coefficient figure of the testing samples

# 8. The method for selecting features: MultiVariate_Logistic

parameters setted: {'P value for threshold in': 0.05, 'P value for threshold out': 0.1}
num of remained features: 3
remained features:
[['wavelet-HHH_firstorder_Mean']
 ['wavelet-HHL_glrlm_LongRunLowGrayLevelEmphasis']
 ['wavelet-HLH_glcm_Correlation']]

Statistical analysis of logistic multivariate analysis:

| feature | OR | 0.025 | 0.975 | P_value |
| --- | --- | --- | --- | --- |
| const | 0.402 | 0.211 | 0.767 | nan |
| wavelet-HHH_firstorder_Mean | 0.336 | 0.137 | 0.824 | 0.017 |
| wavelet-HHL_glrlm_LongRunLowGrayLevelEmphasis | 1.944 | 1.042 | 3.626 | 0.037 |
| wavelet-HLH_glcm_Correlation | 1.912 | 1.01 | 3.619 | 0.046 |

Heatmap of the model in the training samples:


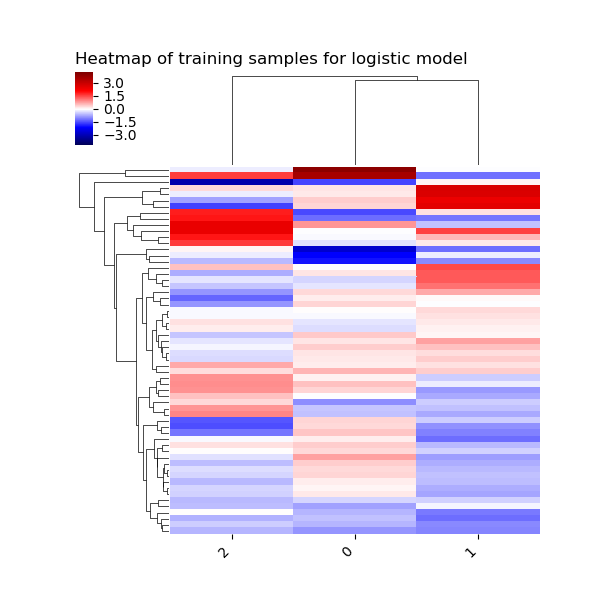


Heatmap of the model in the testing samples:


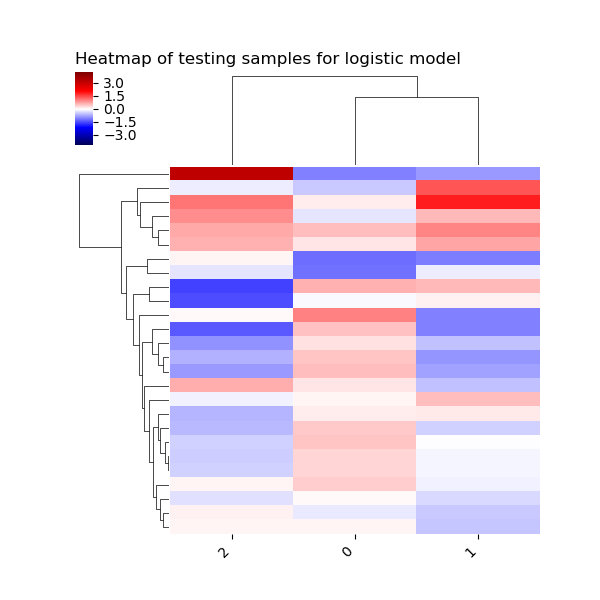


Correlation coefficient figure of the training samples


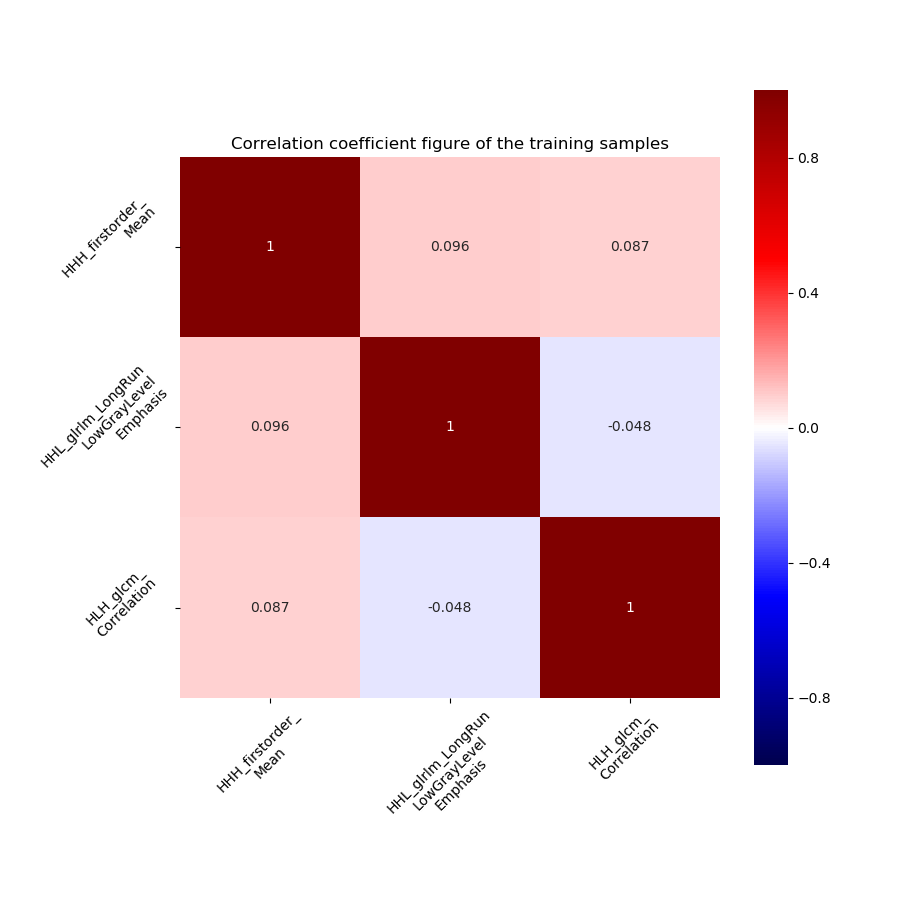


Correlation coefficient figure of the testing samples


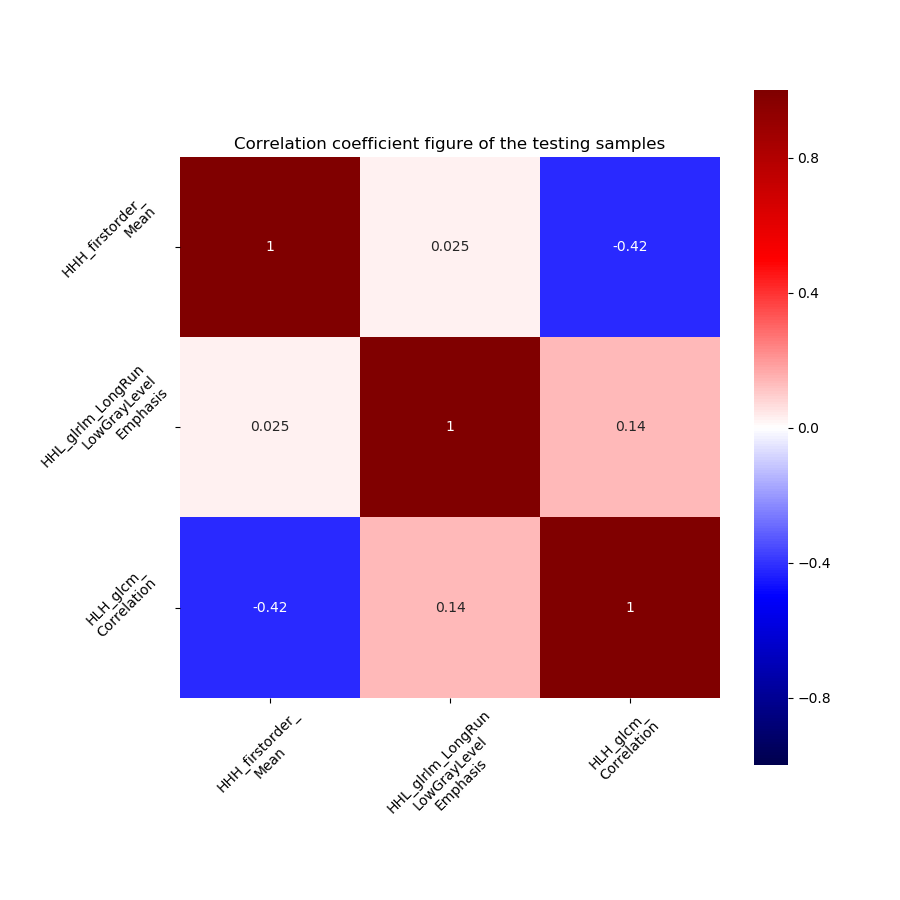


# 9. The best machine learning method: Bayes

the mechine learning method: Bayes

the parameters selection method and selected parameters:
 {'Method': 'auto', 'Model': 'GaussianNB'}

the separate scores and total mean scores of model in each validation fold:
 {'scores': array([0.66666667, 0.75 , 0.58333333, 0.66666667, 0.5 ]), 'mean_score': 0.6333333333333333}

evaluation of the Bayes model in the training and testing samples:

| Item | Train | Test |
| --- | --- | --- |
| Accuracy | 0.75 | 0.615 |
| f1_score | 0.545 | 0.167 |
| Recall | 0.45 | 0.111 |
| Precision | 0.692 | 0.333 |
| AUC | 0.739 (0.626, 0.838) | 0.765 (0.594, 0.922) |
| Sensitivity | 0.45 | 0.111 |
| Specificity | 0.9 | 0.882 |
| positive prediction | 0.692 | 0.333 |
| negative prediction | 0.766 | 0.652 |
| positive llr | 4.5 | 0.944 |
| negatice llr | 0.611 | 1.007 |

ROC of the Bayes model in the training samples:


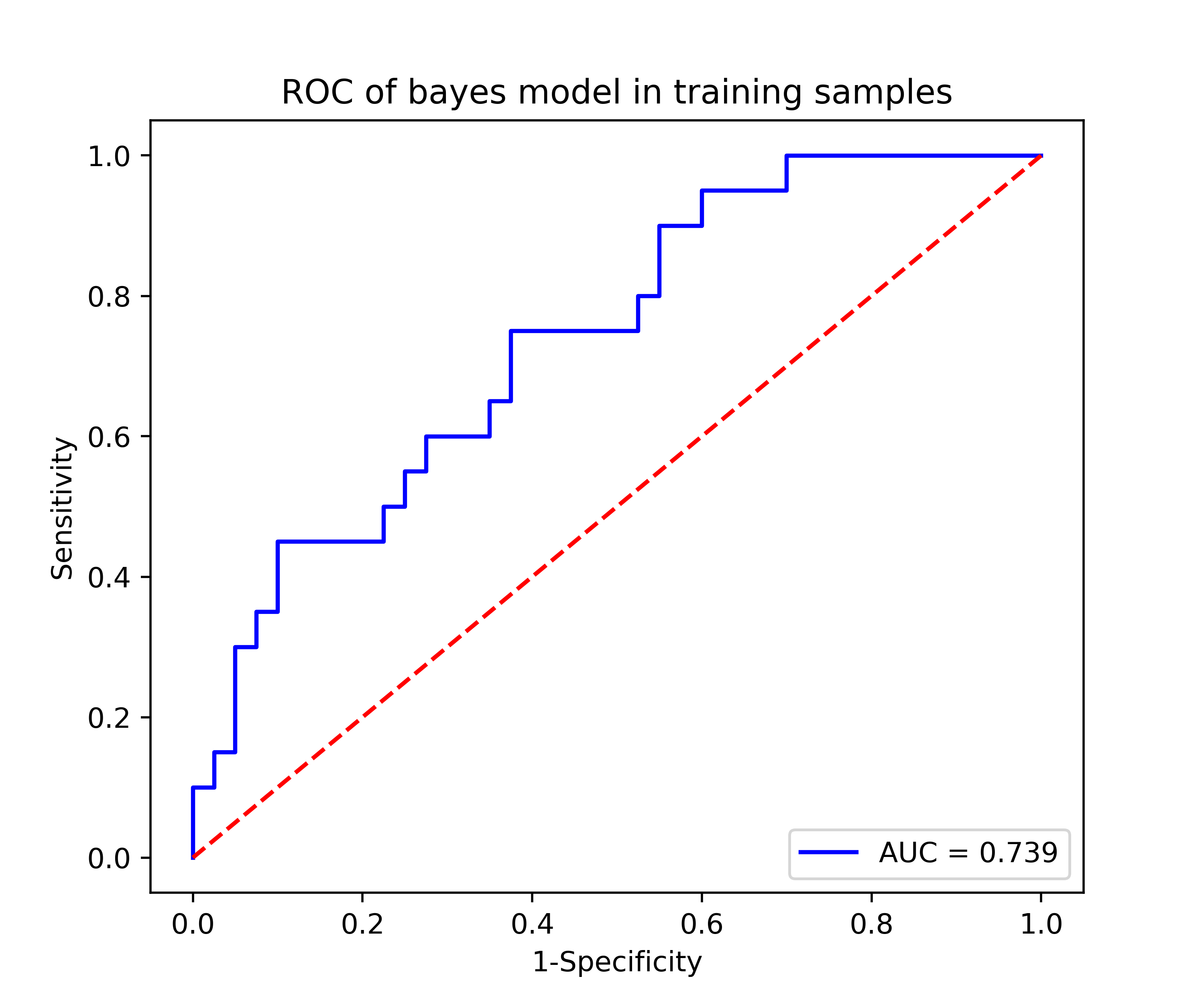


ROC of the Bayes model in the testing samples:


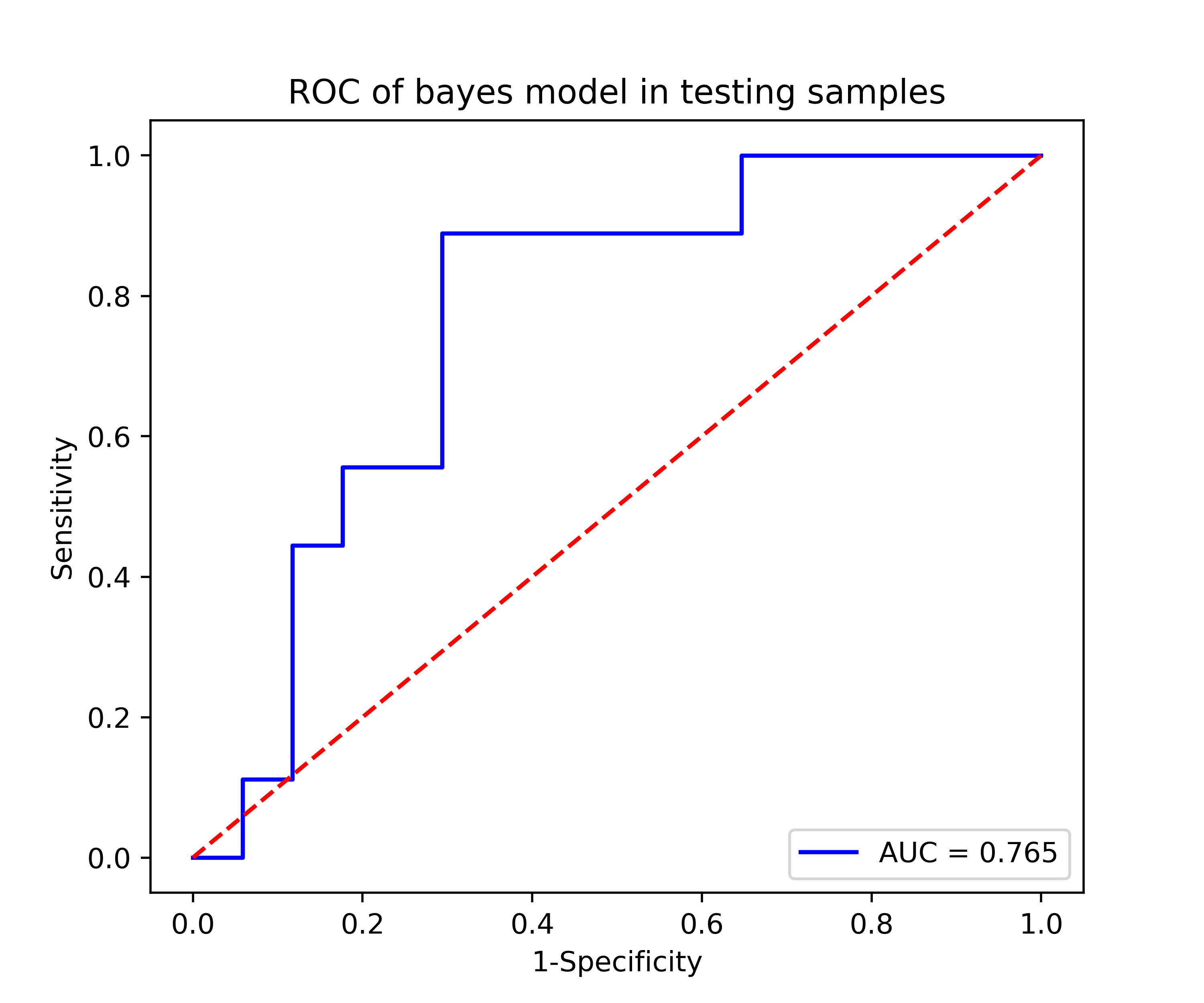


Decision Curve of Bayes model in training samples:


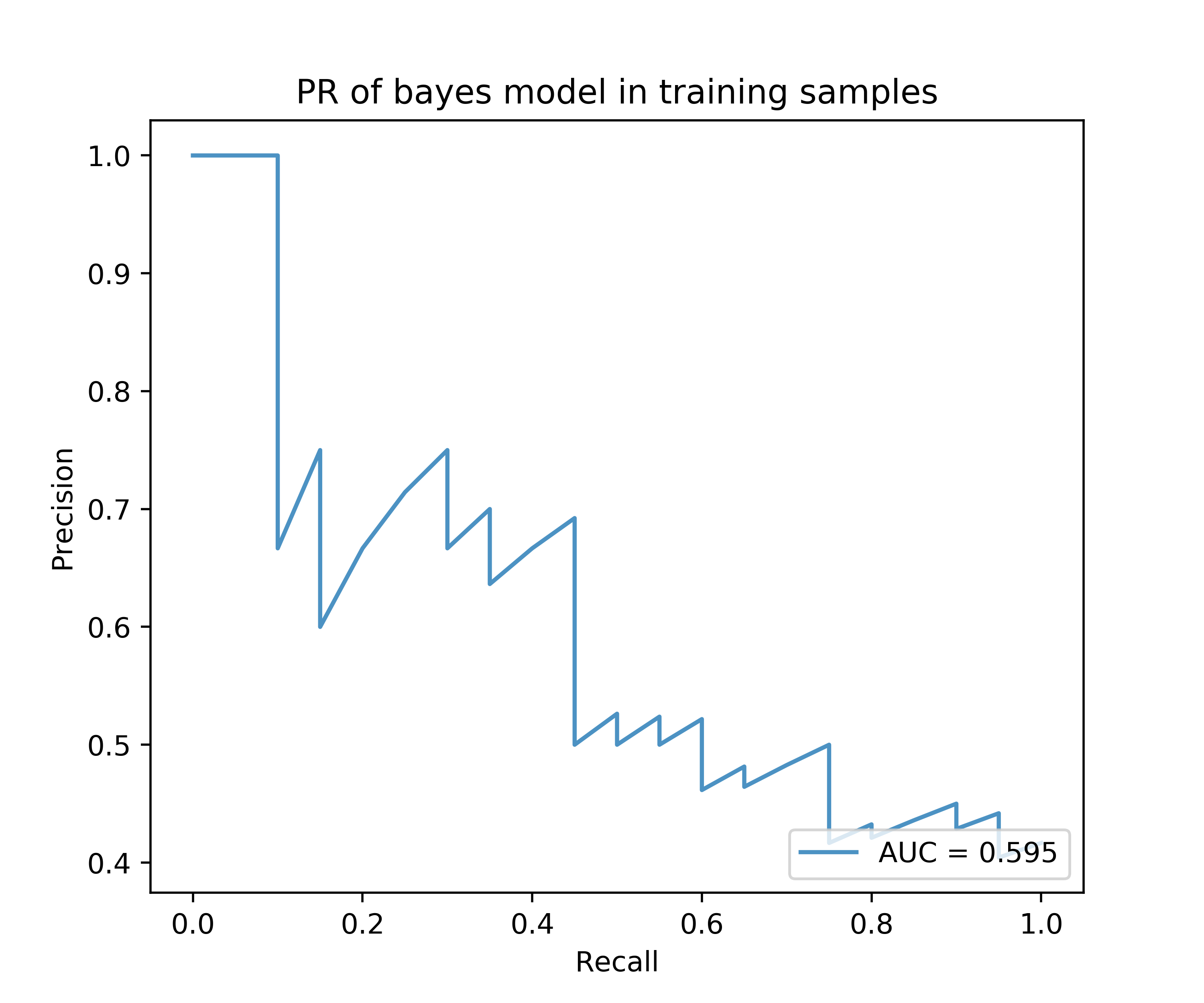


Decision Curve of Bayes model in testing samples:


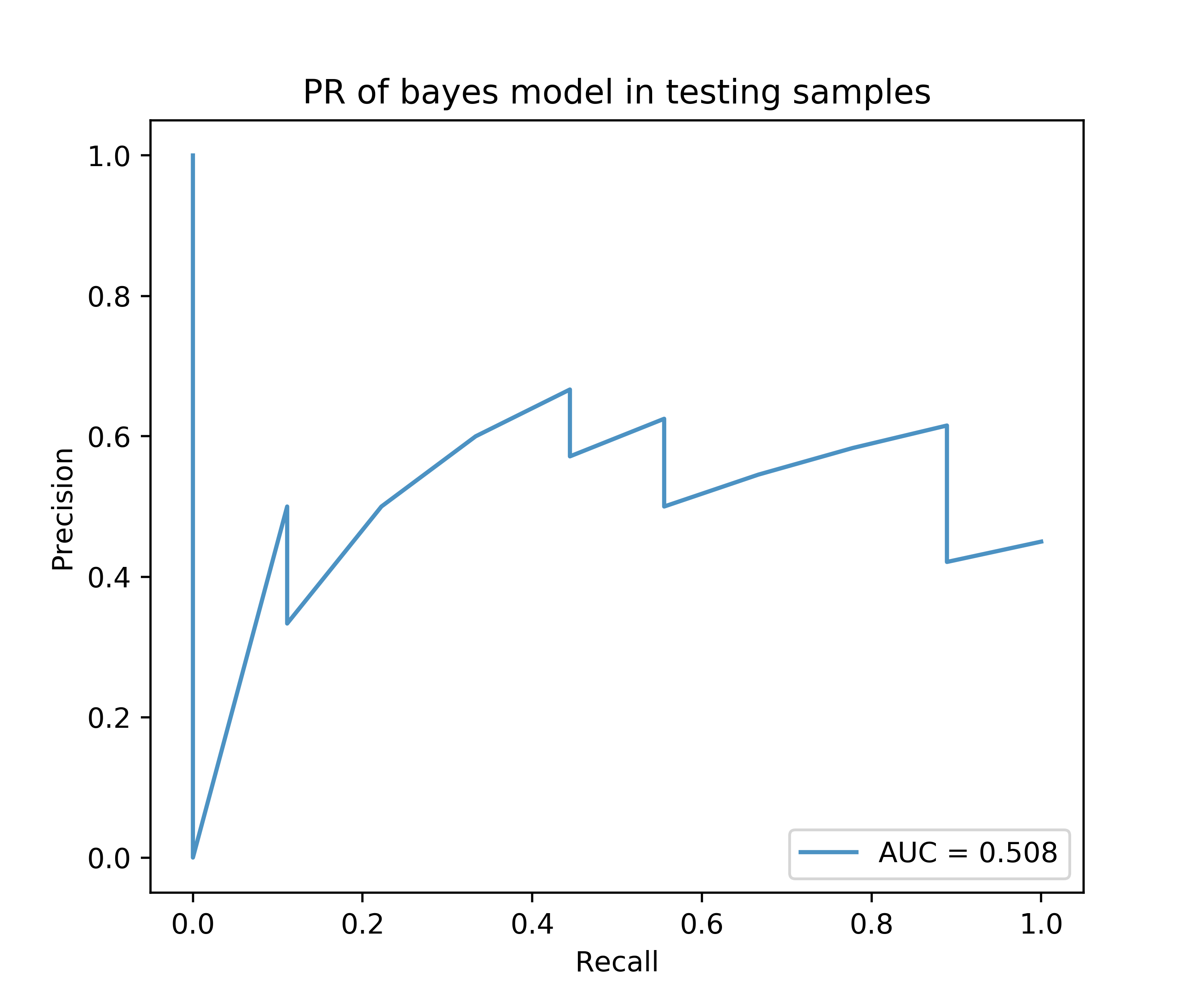


**Radiomics analysis process of decision tree classifier.**

Summary Report

# The summary report recognized the best model from all models your training, and summarized its training process.

As the result shown, the best model was Forest, its training process including: ['select a path', 'set a seed', 'seperate a data', 'input data', 'standardize data', 'select feature', 'select feature', 'select feature', 'machine_learning']. Detailed information is shown below:

# 1. Data: C:/Users/212768837/Desktop/zhuanyi_86ROI/new/result2.csv

# 2. Random seed: 46

# 3. Seperative rate: 0.7

Seperated report:

|  | Sum | Pos | Neg |
| --- | --- | --- | --- |
| data | 86 | 29 | 57 |
| train | 60 | 20 | 40 |
| test | 26 | 9 | 17 |

# 4. Input data

The method for filling the missing data: Median

The method for dealing with outliers: Median

# 5. The method for standardizing the data: Standardization

# 6. The method for selecting features: Variance

parameters setted: {'threshold': 1.0}
num of remained features: 426
remained features:
[['original_shape_Elongation']
 ['original_shape_Flatness']
 ['original_shape_LeastAxisLength']
 ['original_shape_Maximum2DDiameterColumn']
 ['original_shape_Maximum2DDiameterRow']
 ['original_shape_Maximum2DDiameterSlice']
 ['original_shape_MeshVolume']
 ['original_shape_MinorAxisLength']
 ['original_shape_VoxelVolume']
 ['original_firstorder_10Percentile']
 ['original_firstorder_InterquartileRange']
 ['original_firstorder_Kurtosis']
 ['original_firstorder_MeanAbsoluteDeviation']
 ['original_firstorder_Median']
 ['original_firstorder_Minimum']
 ['original_firstorder_Range']
 ['original_firstorder_RootMeanSquared']
 ['original_firstorder_Skewness']
 ['original_glcm_Autocorrelation']
 ['original_glcm_ClusterProminence']
 ['original_glcm_ClusterShade']
 ['original_glcm_ClusterTendency']
 ['original_glcm_Correlation']
 ['original_glcm_DifferenceAverage']
 ['original_glcm_DifferenceEntropy']
 ['original_glcm_Idn']
 ['original_glcm_JointEntropy']
 ['original_glcm_SumSquares']
 ['original_gldm_DependenceNonUniformity']
 ['original_gldm_DependenceVariance']
 ['original_gldm_GrayLevelVariance']
 ['original_gldm_HighGrayLevelEmphasis']
 ['original_gldm_LargeDependenceEmphasis']
 ['original_gldm_LargeDependenceHighGrayLevelEmphasis']
 ['original_gldm_LowGrayLevelEmphasis']
 ['original_gldm_SmallDependenceEmphasis']
 ['original_gldm_SmallDependenceHighGrayLevelEmphasis']
 ['original_gldm_SmallDependenceLowGrayLevelEmphasis']
 ['original_glrlm_GrayLevelNonUniformityNormalized']
 ['original_glrlm_GrayLevelVariance']
 ['original_glrlm_HighGrayLevelRunEmphasis']
 ['original_glrlm_LongRunEmphasis']
 ['original_glrlm_LongRunHighGrayLevelEmphasis']
 ['original_glrlm_LongRunLowGrayLevelEmphasis']
 ['original_glrlm_LowGrayLevelRunEmphasis']
 ['original_glrlm_RunLengthNonUniformityNormalized']
 ['original_glrlm_ShortRunLowGrayLevelEmphasis']
 ['original_glszm_GrayLevelNonUniformity']
 ['original_glszm_GrayLevelVariance']
 ['original_glszm_HighGrayLevelZoneEmphasis']
 ['original_glszm_LargeAreaEmphasis']
 ['original_glszm_LargeAreaHighGrayLevelEmphasis']
 ['original_glszm_SizeZoneNonUniformityNormalized']
 ['original_glszm_SmallAreaHighGrayLevelEmphasis']
 ['original_glszm_SmallAreaLowGrayLevelEmphasis']
 ['original_glszm_ZoneEntropy']
 ['original_glszm_ZonePercentage']
 ['original_ngtdm_Coarseness']
 ['original_ngtdm_Contrast']
 ['wavelet-LLH_firstorder_10Percentile']
 ['wavelet-LLH_firstorder_90Percentile']
 ['wavelet-LLH_firstorder_Energy']
 ['wavelet-LLH_firstorder_Kurtosis']
 ['wavelet-LLH_firstorder_MeanAbsoluteDeviation']
 ['wavelet-LLH_firstorder_Mean']
 ['wavelet-LLH_firstorder_Median']
 ['wavelet-LLH_firstorder_RobustMeanAbsoluteDeviation']
 ['wavelet-LLH_firstorder_TotalEnergy']
 ['wavelet-LLH_firstorder_Uniformity']
 ['wavelet-LLH_firstorder_Variance']
 ['wavelet-LLH_glcm_ClusterTendency']
 ['wavelet-LLH_glcm_Contrast']
 ['wavelet-LLH_glcm_DifferenceEntropy']
 ['wavelet-LLH_glcm_Idm']
 ['wavelet-LLH_glcm_Idmn']
 ['wavelet-LLH_glcm_Idn']
 ['wavelet-LLH_glcm_JointAverage']
 ['wavelet-LLH_glcm_JointEnergy']
 ['wavelet-LLH_glcm_JointEntropy']
 ['wavelet-LLH_glcm_MaximumProbability']
 ['wavelet-LLH_glcm_SumAverage']
 ['wavelet-LLH_glcm_SumSquares']
 ['wavelet-LLH_gldm_GrayLevelNonUniformity']
 ['wavelet-LLH_gldm_HighGrayLevelEmphasis']
 ['wavelet-LLH_gldm_LargeDependenceEmphasis']
 ['wavelet-LLH_gldm_SmallDependenceHighGrayLevelEmphasis']
 ['wavelet-LLH_gldm_SmallDependenceLowGrayLevelEmphasis']
 ['wavelet-LLH_glrlm_GrayLevelVariance']
 ['wavelet-LLH_glrlm_LongRunEmphasis']
 ['wavelet-LLH_glrlm_LongRunHighGrayLevelEmphasis']
 ['wavelet-LLH_glrlm_LongRunLowGrayLevelEmphasis']
 ['wavelet-LLH_glrlm_RunEntropy']
 ['wavelet-LLH_glrlm_RunLengthNonUniformityNormalized']
 ['wavelet-LLH_glrlm_RunPercentage']
 ['wavelet-LLH_glrlm_ShortRunEmphasis']
 ['wavelet-LLH_glrlm_ShortRunLowGrayLevelEmphasis']
 ['wavelet-LLH_glszm_HighGrayLevelZoneEmphasis']
 ['wavelet-LLH_glszm_LargeAreaEmphasis']
 ['wavelet-LLH_glszm_LargeAreaHighGrayLevelEmphasis']
 ['wavelet-LLH_glszm_LargeAreaLowGrayLevelEmphasis']
 ['wavelet-LLH_glszm_SizeZoneNonUniformity']
 ['wavelet-LLH_glszm_SizeZoneNonUniformityNormalized']
 ['wavelet-LLH_glszm_SmallAreaHighGrayLevelEmphasis']
 ['wavelet-LLH_glszm_SmallAreaLowGrayLevelEmphasis']
 ['wavelet-LHL_firstorder_90Percentile']
 ['wavelet-LHL_firstorder_Entropy']
 ['wavelet-LHL_firstorder_Kurtosis']
 ['wavelet-LHL_firstorder_Maximum']
 ['wavelet-LHL_firstorder_MeanAbsoluteDeviation']
 ['wavelet-LHL_firstorder_Mean']
 ['wavelet-LHL_firstorder_Minimum']
 ['wavelet-LHL_firstorder_RobustMeanAbsoluteDeviation']
 ['wavelet-LHL_firstorder_Skewness']
 ['wavelet-LHL_firstorder_Uniformity']
 ['wavelet-LHL_firstorder_Variance']
 ['wavelet-LHL_glcm_Autocorrelation']
 ['wavelet-LHL_glcm_ClusterShade']
 ['wavelet-LHL_glcm_Contrast']
 ['wavelet-LHL_glcm_Correlation']
 ['wavelet-LHL_glcm_DifferenceVariance']
 ['wavelet-LHL_glcm_Idm']
 ['wavelet-LHL_glcm_JointEntropy']
 ['wavelet-LHL_glcm_MaximumProbability']
 ['wavelet-LHL_gldm_DependenceNonUniformityNormalized']
 ['wavelet-LHL_gldm_DependenceVariance']
 ['wavelet-LHL_gldm_HighGrayLevelEmphasis']
 ['wavelet-LHL_gldm_LargeDependenceEmphasis']
 ['wavelet-LHL_gldm_LargeDependenceHighGrayLevelEmphasis']
 ['wavelet-LHL_glrlm_GrayLevelNonUniformity']
 ['wavelet-LHL_glrlm_LongRunLowGrayLevelEmphasis']
 ['wavelet-LHL_glrlm_LowGrayLevelRunEmphasis']
 ['wavelet-LHL_glrlm_RunLengthNonUniformityNormalized']
 ['wavelet-LHL_glrlm_RunVariance']
 ['wavelet-LHL_glrlm_ShortRunLowGrayLevelEmphasis']
 ['wavelet-LHL_glszm_GrayLevelNonUniformity']
 ['wavelet-LHL_glszm_GrayLevelNonUniformityNormalized']
 ['wavelet-LHL_glszm_GrayLevelVariance']
 ['wavelet-LHL_glszm_LargeAreaEmphasis']
 ['wavelet-LHL_glszm_LargeAreaHighGrayLevelEmphasis']
 ['wavelet-LHL_glszm_LargeAreaLowGrayLevelEmphasis']
 ['wavelet-LHL_glszm_SizeZoneNonUniformity']
 ['wavelet-LHL_glszm_SmallAreaEmphasis']
 ['wavelet-LHL_glszm_SmallAreaHighGrayLevelEmphasis']
 ['wavelet-LHL_glszm_ZonePercentage']
 ['wavelet-LHL_ngtdm_Busyness']
 ['wavelet-LHL_ngtdm_Strength']
 ['wavelet-LHH_firstorder_10Percentile']
 ['wavelet-LHH_firstorder_Entropy']
 ['wavelet-LHH_firstorder_InterquartileRange']
 ['wavelet-LHH_firstorder_Maximum']
 ['wavelet-LHH_firstorder_MeanAbsoluteDeviation']
 ['wavelet-LHH_firstorder_Mean']
 ['wavelet-LHH_firstorder_Median']
 ['wavelet-LHH_firstorder_Minimum']
 ['wavelet-LHH_firstorder_RootMeanSquared']
 ['wavelet-LHH_firstorder_Skewness']
 ['wavelet-LHH_firstorder_Uniformity']
 ['wavelet-LHH_glcm_ClusterProminence']
 ['wavelet-LHH_glcm_DifferenceVariance']
 ['wavelet-LHH_glcm_Id']
 ['wavelet-LHH_glcm_Idn']
 ['wavelet-LHH_glcm_Imc2']
 ['wavelet-LHH_glcm_InverseVariance']
 ['wavelet-LHH_glcm_JointEnergy']
 ['wavelet-LHH_glcm_JointEntropy']
 ['wavelet-LHH_glcm_MCC']
 ['wavelet-LHH_glcm_SumSquares']
 ['wavelet-LHH_gldm_DependenceEntropy']
 ['wavelet-LHH_gldm_DependenceNonUniformity']
 ['wavelet-LHH_gldm_GrayLevelVariance']
 ['wavelet-LHH_gldm_HighGrayLevelEmphasis']
 ['wavelet-LHH_gldm_LargeDependenceEmphasis']
 ['wavelet-LHH_gldm_LargeDependenceLowGrayLevelEmphasis']
 ['wavelet-LHH_gldm_SmallDependenceHighGrayLevelEmphasis']
 ['wavelet-LHH_gldm_SmallDependenceLowGrayLevelEmphasis']
 ['wavelet-LHH_glrlm_GrayLevelNonUniformity']
 ['wavelet-LHH_glrlm_GrayLevelNonUniformityNormalized']
 ['wavelet-LHH_glrlm_GrayLevelVariance']
 ['wavelet-LHH_glrlm_HighGrayLevelRunEmphasis']
 ['wavelet-LHH_glrlm_LongRunHighGrayLevelEmphasis']
 ['wavelet-LHH_glrlm_RunEntropy']
 ['wavelet-LHH_glrlm_RunLengthNonUniformity']
 ['wavelet-LHH_glrlm_RunLengthNonUniformityNormalized']
 ['wavelet-LHH_glrlm_RunVariance']
 ['wavelet-LHH_glszm_LargeAreaHighGrayLevelEmphasis']
 ['wavelet-LHH_glszm_LowGrayLevelZoneEmphasis']
 ['wavelet-LHH_glszm_SizeZoneNonUniformity']
 ['wavelet-LHH_glszm_SmallAreaEmphasis']
 ['wavelet-LHH_ngtdm_Busyness']
 ['wavelet-LHH_ngtdm_Strength']
 ['wavelet-HLL_firstorder_90Percentile']
 ['wavelet-HLL_firstorder_Entropy']
 ['wavelet-HLL_firstorder_Maximum']
 ['wavelet-HLL_firstorder_MeanAbsoluteDeviation']
 ['wavelet-HLL_firstorder_Median']
 ['wavelet-HLL_firstorder_Minimum']
 ['wavelet-HLL_firstorder_Range']
 ['wavelet-HLL_firstorder_RobustMeanAbsoluteDeviation']
 ['wavelet-HLL_firstorder_RootMeanSquared']
 ['wavelet-HLL_firstorder_Uniformity']
 ['wavelet-HLL_glcm_ClusterProminence']
 ['wavelet-HLL_glcm_Contrast']
 ['wavelet-HLL_glcm_DifferenceAverage']
 ['wavelet-HLL_glcm_Idmn']
 ['wavelet-HLL_glcm_Idn']
 ['wavelet-HLL_glcm_Imc1']
 ['wavelet-HLL_glcm_Imc2']
 ['wavelet-HLL_glcm_JointAverage']
 ['wavelet-HLL_glcm_JointEntropy']
 ['wavelet-HLL_glcm_SumAverage']
 ['wavelet-HLL_glcm_SumEntropy']
 ['wavelet-HLL_gldm_DependenceNonUniformity']
 ['wavelet-HLL_gldm_DependenceVariance']
 ['wavelet-HLL_gldm_GrayLevelVariance']
 ['wavelet-HLL_gldm_LargeDependenceEmphasis']
 ['wavelet-HLL_gldm_LowGrayLevelEmphasis']
 ['wavelet-HLL_gldm_SmallDependenceEmphasis']
 ['wavelet-HLL_gldm_SmallDependenceLowGrayLevelEmphasis']
 ['wavelet-HLL_glrlm_GrayLevelVariance']
 ['wavelet-HLL_glrlm_HighGrayLevelRunEmphasis']
 ['wavelet-HLL_glrlm_LongRunEmphasis']
 ['wavelet-HLL_glrlm_LongRunLowGrayLevelEmphasis']
 ['wavelet-HLL_glrlm_RunPercentage']
 ['wavelet-HLL_glrlm_RunVariance']
 ['wavelet-HLL_glrlm_ShortRunLowGrayLevelEmphasis']
 ['wavelet-HLL_glszm_GrayLevelNonUniformityNormalized']
 ['wavelet-HLL_glszm_GrayLevelVariance']
 ['wavelet-HLL_glszm_LargeAreaEmphasis']
 ['wavelet-HLL_glszm_LargeAreaLowGrayLevelEmphasis']
 ['wavelet-HLL_glszm_SizeZoneNonUniformity']
 ['wavelet-HLL_glszm_SmallAreaEmphasis']
 ['wavelet-HLL_glszm_SmallAreaLowGrayLevelEmphasis']
 ['wavelet-HLL_glszm_ZoneVariance']
 ['wavelet-HLL_ngtdm_Busyness']
 ['wavelet-HLL_ngtdm_Coarseness']
 ['wavelet-HLH_firstorder_10Percentile']
 ['wavelet-HLH_firstorder_90Percentile']
 ['wavelet-HLH_firstorder_Entropy']
 ['wavelet-HLH_firstorder_InterquartileRange']
 ['wavelet-HLH_firstorder_Kurtosis']
 ['wavelet-HLH_firstorder_Mean']
 ['wavelet-HLH_firstorder_RobustMeanAbsoluteDeviation']
 ['wavelet-HLH_glcm_Autocorrelation']
 ['wavelet-HLH_glcm_ClusterShade']
 ['wavelet-HLH_glcm_Correlation']
 ['wavelet-HLH_glcm_DifferenceAverage']
 ['wavelet-HLH_glcm_DifferenceVariance']
 ['wavelet-HLH_glcm_Id']
 ['wavelet-HLH_glcm_Idmn']
 ['wavelet-HLH_glcm_JointAverage']
 ['wavelet-HLH_glcm_MCC']
 ['wavelet-HLH_glcm_MaximumProbability']
 ['wavelet-HLH_glcm_SumAverage']
 ['wavelet-HLH_glcm_SumSquares']
 ['wavelet-HLH_gldm_DependenceEntropy']
 ['wavelet-HLH_gldm_DependenceVariance']
 ['wavelet-HLH_gldm_GrayLevelNonUniformity']
 ['wavelet-HLH_gldm_LargeDependenceEmphasis']
 ['wavelet-HLH_gldm_LargeDependenceHighGrayLevelEmphasis']
 ['wavelet-HLH_gldm_LowGrayLevelEmphasis']
 ['wavelet-HLH_glrlm_GrayLevelNonUniformity']
 ['wavelet-HLH_glrlm_GrayLevelVariance']
 ['wavelet-HLH_glrlm_HighGrayLevelRunEmphasis']
 ['wavelet-HLH_glrlm_LongRunHighGrayLevelEmphasis']
 ['wavelet-HLH_glrlm_LongRunLowGrayLevelEmphasis']
 ['wavelet-HLH_glrlm_LowGrayLevelRunEmphasis']
 ['wavelet-HLH_glrlm_RunEntropy']
 ['wavelet-HLH_glrlm_RunLengthNonUniformityNormalized']
 ['wavelet-HLH_glrlm_RunPercentage']
 ['wavelet-HLH_glrlm_RunVariance']
 ['wavelet-HLH_glrlm_ShortRunLowGrayLevelEmphasis']
 ['wavelet-HLH_glszm_LargeAreaHighGrayLevelEmphasis']
 ['wavelet-HLH_glszm_LargeAreaLowGrayLevelEmphasis']
 ['wavelet-HLH_glszm_SizeZoneNonUniformity']
 ['wavelet-HLH_glszm_SmallAreaEmphasis']
 ['wavelet-HLH_glszm_SmallAreaHighGrayLevelEmphasis']
 ['wavelet-HLH_glszm_SmallAreaLowGrayLevelEmphasis']
 ['wavelet-HLH_glszm_ZoneEntropy']
 ['wavelet-HLH_glszm_ZonePercentage']
 ['wavelet-HLH_glszm_ZoneVariance']
 ['wavelet-HLH_ngtdm_Busyness']
 ['wavelet-HLH_ngtdm_Coarseness']
 ['wavelet-HLH_ngtdm_Contrast']
 ['wavelet-HHL_firstorder_10Percentile']
 ['wavelet-HHL_firstorder_90Percentile']
 ['wavelet-HHL_firstorder_InterquartileRange']
 ['wavelet-HHL_firstorder_Kurtosis']
 ['wavelet-HHL_firstorder_Mean']
 ['wavelet-HHL_firstorder_Minimum']
 ['wavelet-HHL_firstorder_RobustMeanAbsoluteDeviation']
 ['wavelet-HHL_firstorder_RootMeanSquared']
 ['wavelet-HHL_firstorder_Skewness']
 ['wavelet-HHL_firstorder_Uniformity']
 ['wavelet-HHL_glcm_ClusterShade']
 ['wavelet-HHL_glcm_ClusterTendency']
 ['wavelet-HHL_glcm_DifferenceAverage']
 ['wavelet-HHL_glcm_Id']
 ['wavelet-HHL_glcm_Idn']
 ['wavelet-HHL_glcm_Imc1']
 ['wavelet-HHL_glcm_Imc2']
 ['wavelet-HHL_glcm_JointAverage']
 ['wavelet-HHL_glcm_JointEnergy']
 ['wavelet-HHL_glcm_SumAverage']
 ['wavelet-HHL_glcm_SumSquares']
 ['wavelet-HHL_gldm_DependenceVariance']
 ['wavelet-HHL_gldm_GrayLevelNonUniformity']
 ['wavelet-HHL_gldm_GrayLevelVariance']
 ['wavelet-HHL_gldm_HighGrayLevelEmphasis']
 ['wavelet-HHL_gldm_LargeDependenceHighGrayLevelEmphasis']
 ['wavelet-HHL_gldm_LargeDependenceLowGrayLevelEmphasis']
 ['wavelet-HHL_gldm_SmallDependenceEmphasis']
 ['wavelet-HHL_gldm_SmallDependenceLowGrayLevelEmphasis']
 ['wavelet-HHL_glrlm_GrayLevelNonUniformity']
 ['wavelet-HHL_glrlm_GrayLevelNonUniformityNormalized']
 ['wavelet-HHL_glrlm_HighGrayLevelRunEmphasis']
 ['wavelet-HHL_glrlm_LongRunEmphasis']
 ['wavelet-HHL_glrlm_LongRunHighGrayLevelEmphasis']
 ['wavelet-HHL_glrlm_LongRunLowGrayLevelEmphasis']
 ['wavelet-HHL_glrlm_RunPercentage']
 ['wavelet-HHL_glrlm_RunVariance']
 ['wavelet-HHL_glrlm_ShortRunHighGrayLevelEmphasis']
 ['wavelet-HHL_glrlm_ShortRunLowGrayLevelEmphasis']
 ['wavelet-HHL_glszm_GrayLevelVariance']
 ['wavelet-HHL_glszm_HighGrayLevelZoneEmphasis']
 ['wavelet-HHL_glszm_LargeAreaEmphasis']
 ['wavelet-HHL_glszm_LargeAreaLowGrayLevelEmphasis']
 ['wavelet-HHL_glszm_LowGrayLevelZoneEmphasis']
 ['wavelet-HHL_glszm_SizeZoneNonUniformity']
 ['wavelet-HHL_glszm_SizeZoneNonUniformityNormalized']
 ['wavelet-HHL_glszm_ZoneEntropy']
 ['wavelet-HHL_glszm_ZonePercentage']
 ['wavelet-HHL_ngtdm_Busyness']
 ['wavelet-HHL_ngtdm_Coarseness']
 ['wavelet-HHL_ngtdm_Complexity']
 ['wavelet-HHL_ngtdm_Contrast']
 ['wavelet-HHL_ngtdm_Strength']
 ['wavelet-HHH_firstorder_10Percentile']
 ['wavelet-HHH_firstorder_Energy']
 ['wavelet-HHH_firstorder_Entropy']
 ['wavelet-HHH_firstorder_InterquartileRange']
 ['wavelet-HHH_firstorder_MeanAbsoluteDeviation']
 ['wavelet-HHH_firstorder_Median']
 ['wavelet-HHH_firstorder_Range']
 ['wavelet-HHH_firstorder_RootMeanSquared']
 ['wavelet-HHH_firstorder_TotalEnergy']
 ['wavelet-HHH_firstorder_Uniformity']
 ['wavelet-HHH_glcm_Autocorrelation']
 ['wavelet-HHH_glcm_Contrast']
 ['wavelet-HHH_glcm_Correlation']
 ['wavelet-HHH_glcm_DifferenceAverage']
 ['wavelet-HHH_glcm_DifferenceVariance']
 ['wavelet-HHH_glcm_Id']
 ['wavelet-HHH_glcm_Idn']
 ['wavelet-HHH_glcm_Imc1']
 ['wavelet-HHH_glcm_Imc2']
 ['wavelet-HHH_glcm_InverseVariance']
 ['wavelet-HHH_glcm_JointEntropy']
 ['wavelet-HHH_glcm_MCC']
 ['wavelet-HHH_glcm_SumEntropy']
 ['wavelet-HHH_glcm_SumSquares']
 ['wavelet-HHH_gldm_DependenceEntropy']
 ['wavelet-HHH_gldm_GrayLevelNonUniformity']
 ['wavelet-HHH_gldm_GrayLevelVariance']
 ['wavelet-HHH_gldm_HighGrayLevelEmphasis']
 ['wavelet-HHH_gldm_LowGrayLevelEmphasis']
 ['wavelet-HHH_gldm_SmallDependenceHighGrayLevelEmphasis']
 ['wavelet-HHH_glrlm_GrayLevelVariance']
 ['wavelet-HHH_glrlm_HighGrayLevelRunEmphasis']
 ['wavelet-HHH_glrlm_LongRunHighGrayLevelEmphasis']
 ['wavelet-HHH_glrlm_LongRunLowGrayLevelEmphasis']
 ['wavelet-HHH_glrlm_RunLengthNonUniformityNormalized']
 ['wavelet-HHH_glrlm_RunPercentage']
 ['wavelet-HHH_glrlm_RunVariance']
 ['wavelet-HHH_glszm_HighGrayLevelZoneEmphasis']
 ['wavelet-HHH_glszm_LargeAreaEmphasis']
 ['wavelet-HHH_glszm_SizeZoneNonUniformity']
 ['wavelet-HHH_glszm_SmallAreaEmphasis']
 ['wavelet-HHH_glszm_ZonePercentage']
 ['wavelet-HHH_ngtdm_Strength']
 ['wavelet-LLL_firstorder_Entropy']
 ['wavelet-LLL_firstorder_Maximum']
 ['wavelet-LLL_firstorder_MeanAbsoluteDeviation']
 ['wavelet-LLL_firstorder_Mean']
 ['wavelet-LLL_firstorder_Median']
 ['wavelet-LLL_firstorder_Range']
 ['wavelet-LLL_firstorder_Uniformity']
 ['wavelet-LLL_firstorder_Variance']
 ['wavelet-LLL_glcm_Autocorrelation']
 ['wavelet-LLL_glcm_ClusterTendency']
 ['wavelet-LLL_glcm_Correlation']
 ['wavelet-LLL_glcm_DifferenceEntropy']
 ['wavelet-LLL_glcm_Idmn']
 ['wavelet-LLL_glcm_Imc1']
 ['wavelet-LLL_glcm_Imc2']
 ['wavelet-LLL_glcm_InverseVariance']
 ['wavelet-LLL_glcm_JointEnergy']
 ['wavelet-LLL_glcm_JointEntropy']
 ['wavelet-LLL_glcm_MaximumProbability']
 ['wavelet-LLL_gldm_DependenceEntropy']
 ['wavelet-LLL_gldm_DependenceVariance']
 ['wavelet-LLL_gldm_GrayLevelNonUniformity']
 ['wavelet-LLL_gldm_GrayLevelVariance']
 ['wavelet-LLL_gldm_LargeDependenceHighGrayLevelEmphasis']
 ['wavelet-LLL_gldm_LargeDependenceLowGrayLevelEmphasis']
 ['wavelet-LLL_gldm_LowGrayLevelEmphasis']
 ['wavelet-LLL_gldm_SmallDependenceEmphasis']
 ['wavelet-LLL_gldm_SmallDependenceHighGrayLevelEmphasis']
 ['wavelet-LLL_glrlm_GrayLevelNonUniformity']
 ['wavelet-LLL_glrlm_HighGrayLevelRunEmphasis']
 ['wavelet-LLL_glrlm_LongRunEmphasis']
 ['wavelet-LLL_glrlm_LongRunHighGrayLevelEmphasis']
 ['wavelet-LLL_glrlm_LowGrayLevelRunEmphasis']
 ['wavelet-LLL_glrlm_RunEntropy']
 ['wavelet-LLL_glrlm_ShortRunEmphasis']
 ['wavelet-LLL_glszm_GrayLevelNonUniformity']
 ['wavelet-LLL_glszm_GrayLevelNonUniformityNormalized']
 ['wavelet-LLL_glszm_GrayLevelVariance']
 ['wavelet-LLL_glszm_LargeAreaHighGrayLevelEmphasis']
 ['wavelet-LLL_glszm_LargeAreaLowGrayLevelEmphasis']
 ['wavelet-LLL_glszm_LowGrayLevelZoneEmphasis']
 ['wavelet-LLL_glszm_SizeZoneNonUniformity']
 ['wavelet-LLL_glszm_SizeZoneNonUniformityNormalized']
 ['wavelet-LLL_glszm_SmallAreaLowGrayLevelEmphasis']
 ['wavelet-LLL_glszm_ZonePercentage']
 ['wavelet-LLL_ngtdm_Coarseness']
 ['wavelet-LLL_ngtdm_Strength']]

Heatmap of the model in the training samples:


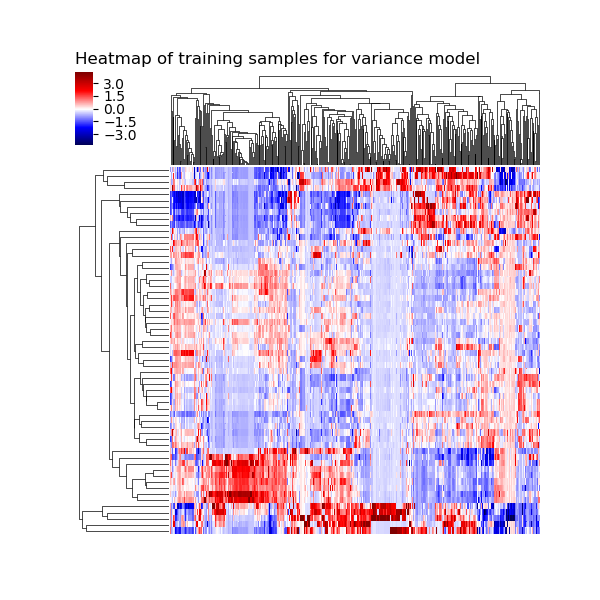


Heatmap of the model in the testing samples:


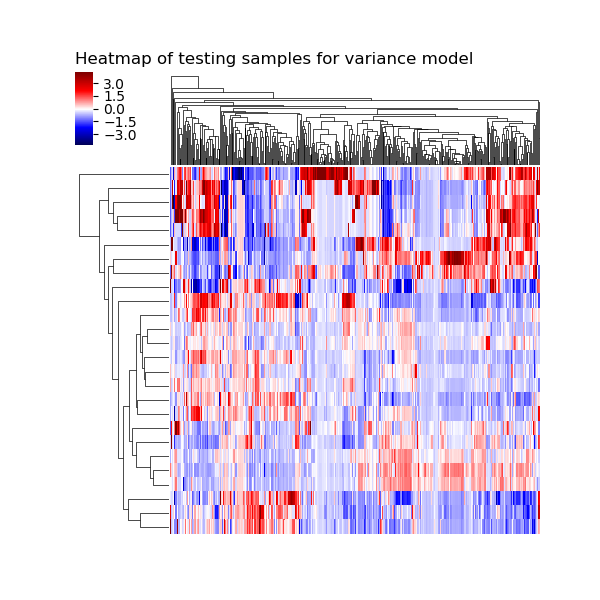


Correlation coefficient figure of the training samples

Correlation coefficient figure of the testing samples

# 7. The method for selecting features: Correlation_xx

parameters setted: {'cutoff': 0.7}
num of remained features: 76
remained features:
[['original_shape_Flatness']
 ['original_firstorder_Minimum']
 ['original_glcm_ClusterShade']
 ['original_glrlm_LongRunLowGrayLevelEmphasis']
 ['original_glszm_GrayLevelVariance']
 ['original_glszm_LargeAreaHighGrayLevelEmphasis']
 ['original_glszm_SmallAreaLowGrayLevelEmphasis']
 ['original_ngtdm_Contrast']
 ['wavelet-LLH_firstorder_10Percentile']
 ['wavelet-LLH_firstorder_90Percentile']
 ['wavelet-LLH_firstorder_Kurtosis']
 ['wavelet-LLH_firstorder_Median']
 ['wavelet-LLH_glcm_SumAverage']
 ['wavelet-LLH_glrlm_LongRunHighGrayLevelEmphasis']
 ['wavelet-LLH_glszm_SizeZoneNonUniformityNormalized']
 ['wavelet-LLH_glszm_SmallAreaHighGrayLevelEmphasis']
 ['wavelet-LLH_glszm_SmallAreaLowGrayLevelEmphasis']
 ['wavelet-LHL_firstorder_90Percentile']
 ['wavelet-LHL_firstorder_Mean']
 ['wavelet-LHL_firstorder_Skewness']
 ['wavelet-LHL_glcm_ClusterShade']
 ['wavelet-LHL_glcm_Contrast']
 ['wavelet-LHL_glcm_Correlation']
 ['wavelet-LHL_glcm_DifferenceVariance']
 ['wavelet-LHL_glrlm_LongRunLowGrayLevelEmphasis']
 ['wavelet-LHL_glszm_GrayLevelNonUniformityNormalized']
 ['wavelet-LHL_glszm_LargeAreaLowGrayLevelEmphasis']
 ['wavelet-LHH_firstorder_Maximum']
 ['wavelet-LHH_firstorder_Skewness']
 ['wavelet-LHH_glcm_InverseVariance']
 ['wavelet-LHH_gldm_LargeDependenceLowGrayLevelEmphasis']
 ['wavelet-LHH_glszm_SmallAreaEmphasis']
 ['wavelet-LHH_ngtdm_Busyness']
 ['wavelet-HLL_firstorder_90Percentile']
 ['wavelet-HLL_firstorder_Median']
 ['wavelet-HLL_glcm_ClusterProminence']
 ['wavelet-HLL_glcm_Imc2']
 ['wavelet-HLL_glszm_GrayLevelNonUniformityNormalized']
 ['wavelet-HLL_glszm_LargeAreaLowGrayLevelEmphasis']
 ['wavelet-HLL_glszm_SmallAreaEmphasis']
 ['wavelet-HLL_glszm_SmallAreaLowGrayLevelEmphasis']
 ['wavelet-HLH_firstorder_Mean']
 ['wavelet-HLH_glcm_Correlation']
 ['wavelet-HLH_glcm_MaximumProbability']
 ['wavelet-HLH_gldm_DependenceEntropy']
 ['wavelet-HLH_gldm_DependenceVariance']
 ['wavelet-HLH_glrlm_LongRunLowGrayLevelEmphasis']
 ['wavelet-HLH_glszm_SmallAreaEmphasis']
 ['wavelet-HLH_glszm_SmallAreaHighGrayLevelEmphasis']
 ['wavelet-HLH_glszm_SmallAreaLowGrayLevelEmphasis']
 ['wavelet-HLH_ngtdm_Busyness']
 ['wavelet-HHL_firstorder_Mean']
 ['wavelet-HHL_firstorder_Skewness']
 ['wavelet-HHL_glcm_ClusterShade']
 ['wavelet-HHL_glcm_ClusterTendency']
 ['wavelet-HHL_glcm_Imc2']
 ['wavelet-HHL_gldm_DependenceVariance']
 ['wavelet-HHL_gldm_LargeDependenceLowGrayLevelEmphasis']
 ['wavelet-HHL_glrlm_ShortRunHighGrayLevelEmphasis']
 ['wavelet-HHL_glszm_LargeAreaLowGrayLevelEmphasis']
 ['wavelet-HHL_glszm_SizeZoneNonUniformityNormalized']
 ['wavelet-HHL_ngtdm_Coarseness']
 ['wavelet-HHL_ngtdm_Strength']
 ['wavelet-HHH_firstorder_Median']
 ['wavelet-HHH_glcm_Correlation']
 ['wavelet-HHH_glcm_DifferenceAverage']
 ['wavelet-HHH_glcm_Idn']
 ['wavelet-HHH_glcm_MCC']
 ['wavelet-HHH_gldm_DependenceEntropy']
 ['wavelet-HHH_glszm_SizeZoneNonUniformity']
 ['wavelet-HHH_glszm_SmallAreaEmphasis']
 ['wavelet-LLL_glcm_JointEnergy']
 ['wavelet-LLL_gldm_LargeDependenceLowGrayLevelEmphasis']
 ['wavelet-LLL_glszm_LargeAreaLowGrayLevelEmphasis']
 ['wavelet-LLL_glszm_SizeZoneNonUniformityNormalized']
 ['wavelet-LLL_ngtdm_Strength']]

Heatmap of the model in the training samples:


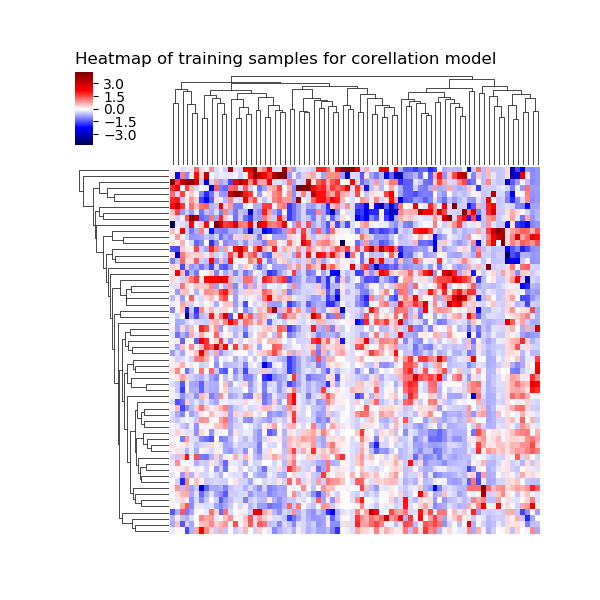


Heatmap of the model in the testing samples:


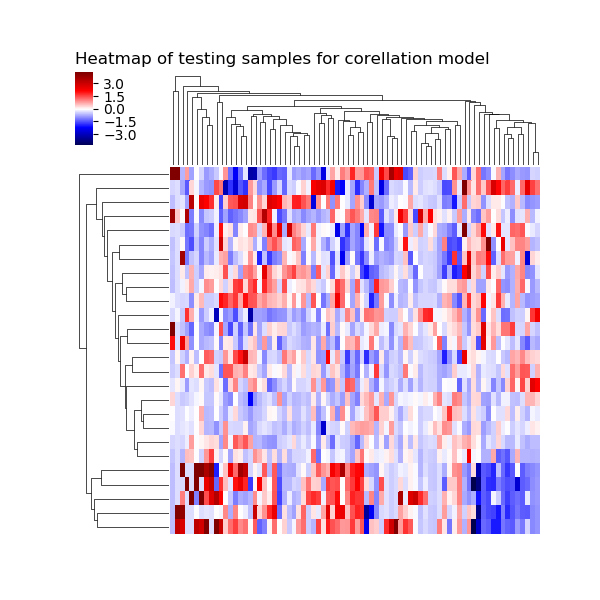


Correlation coefficient figure of the training samples

Correlation coefficient figure of the testing samples

# 8. The method for selecting features: MultiVariate_Logistic

parameters setted: {'P value for threshold in': 0.05, 'P value for threshold out': 0.1}
num of remained features: 2
remained features:
[['wavelet-LHL_glrlm_LongRunLowGrayLevelEmphasis']
 ['wavelet-LLH_firstorder_10Percentile']]

Statistical analysis of logistic multivariate analysis:

| feature | OR | 0.025 | 0.975 | P_value |
| --- | --- | --- | --- | --- |
| const | 0.44 | 0.238 | 0.814 | nan |
| wavelet-LHL_glrlm_LongRunLowGrayLevelEmphasis | 2.492 | 1.308 | 4.748 | 0.006 |
| wavelet-LLH_firstorder_10Percentile | 0.437 | 0.227 | 0.842 | 0.013 |

Heatmap of the model in the training samples:


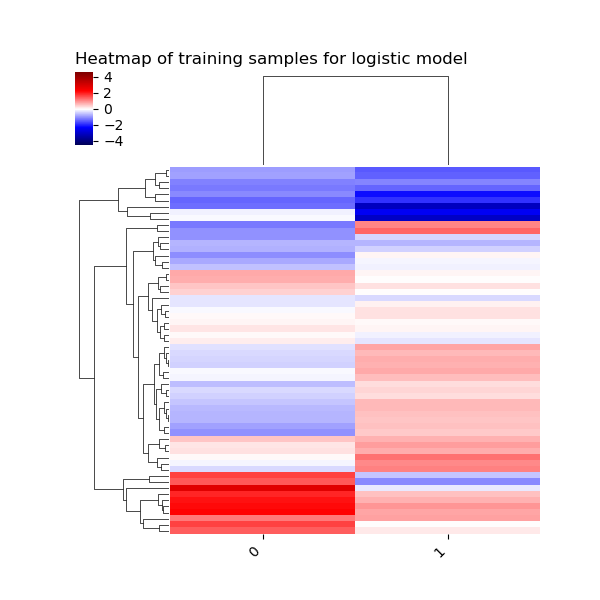


Heatmap of the model in the testing samples:


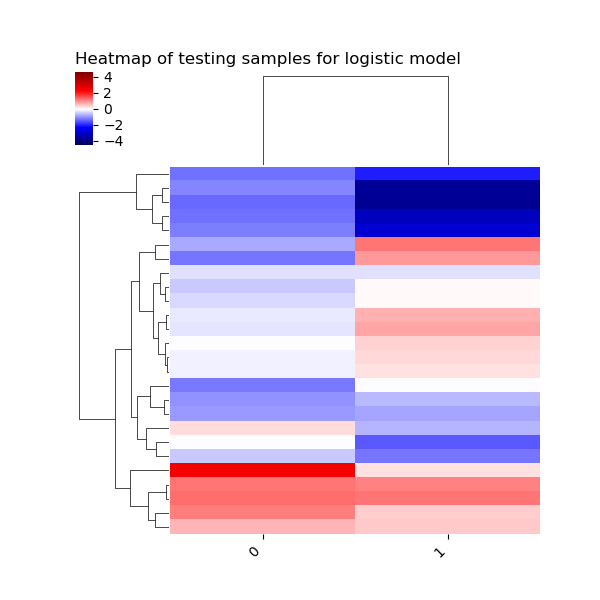


Correlation coefficient figure of the training samples


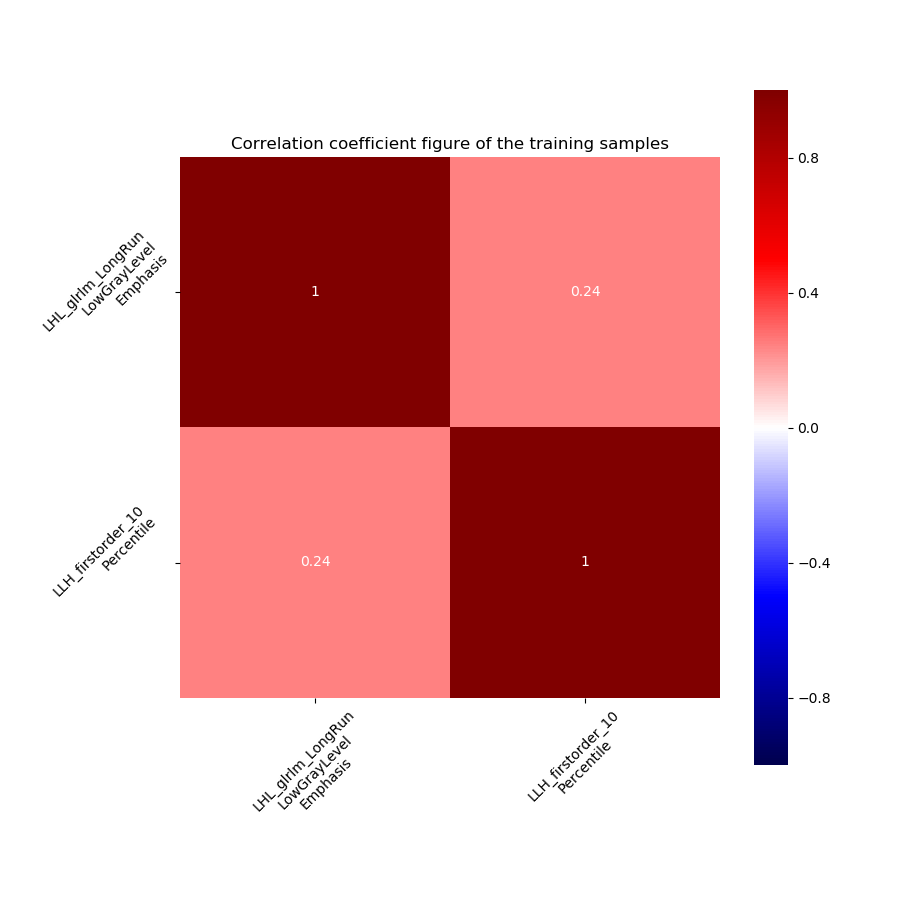


Correlation coefficient figure of the testing samples


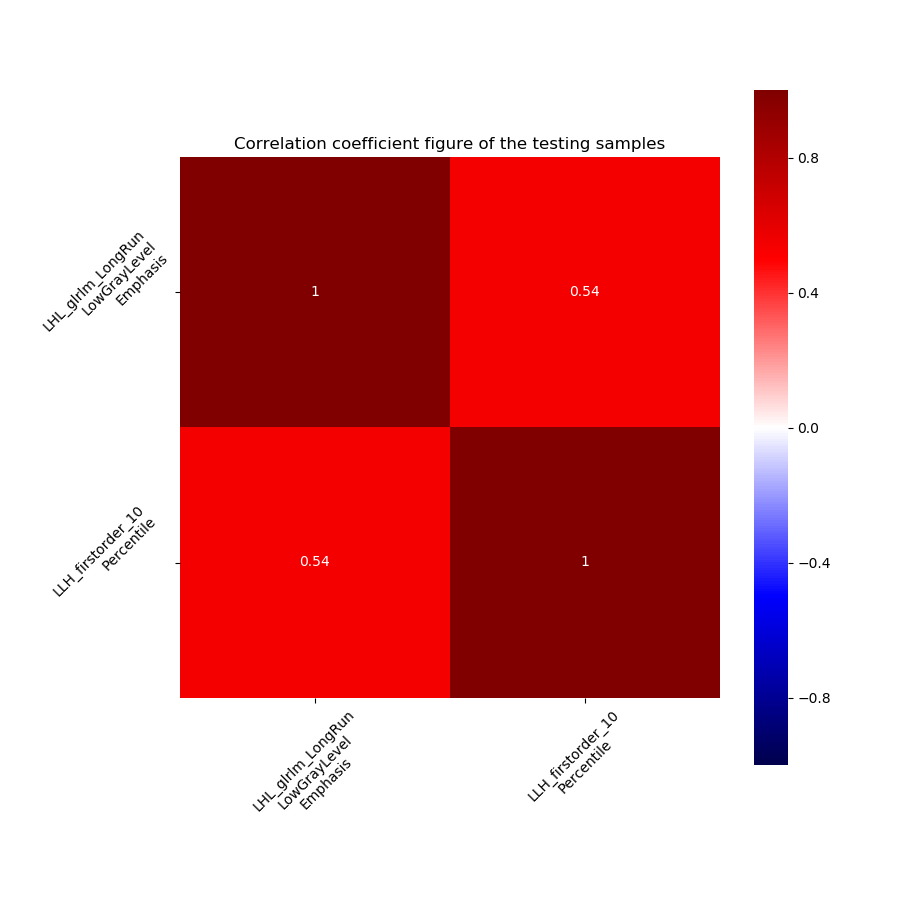


# 9. The best machine learning method: Forest

the mechine learning method: Forest

the parameters selection method and selected parameters:
 {'Method': 'auto', 'max_depth': 1, 'min_samples_split': 2, 'n_estimators': 15}

the separate scores and total mean scores of model in each validation fold:

【Note】the validation cross process has not been operated.

evaluation of the Forest model in the training and testing samples:

| Item | Train | Test |
| --- | --- | --- |
| Accuracy | 0.75 | 0.692 |
| f1_score | 0.483 | 0.2 |
| Recall | 0.35 | 0.111 |
| Precision | 0.778 | 1.0 |
| AUC | 0.773 (0.659, 0.878) | 0.627 (0.441, 0.823) |
| Sensitivity | 0.35 | 0.111 |
| Specificity | 0.95 | 1.0 |
| positive prediction | 0.778 | 1.0 |
| negative prediction | 0.745 | 0.68 |
| positive llr | 7.0 | inf |
| negatice llr | 0.684 | 0.889 |

ROC of the Forest model in the training samples:


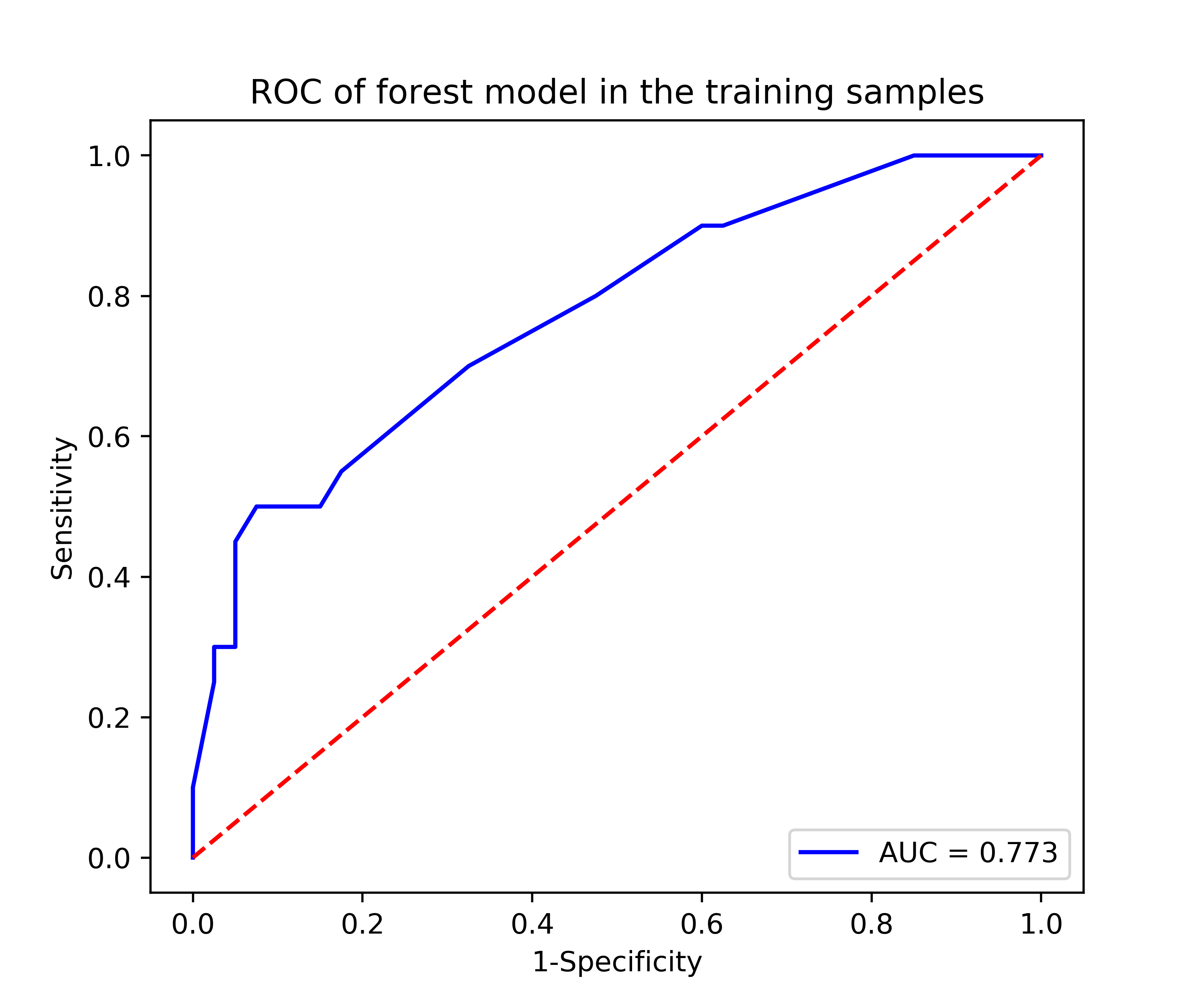


ROC of the Forest model in the testing samples:


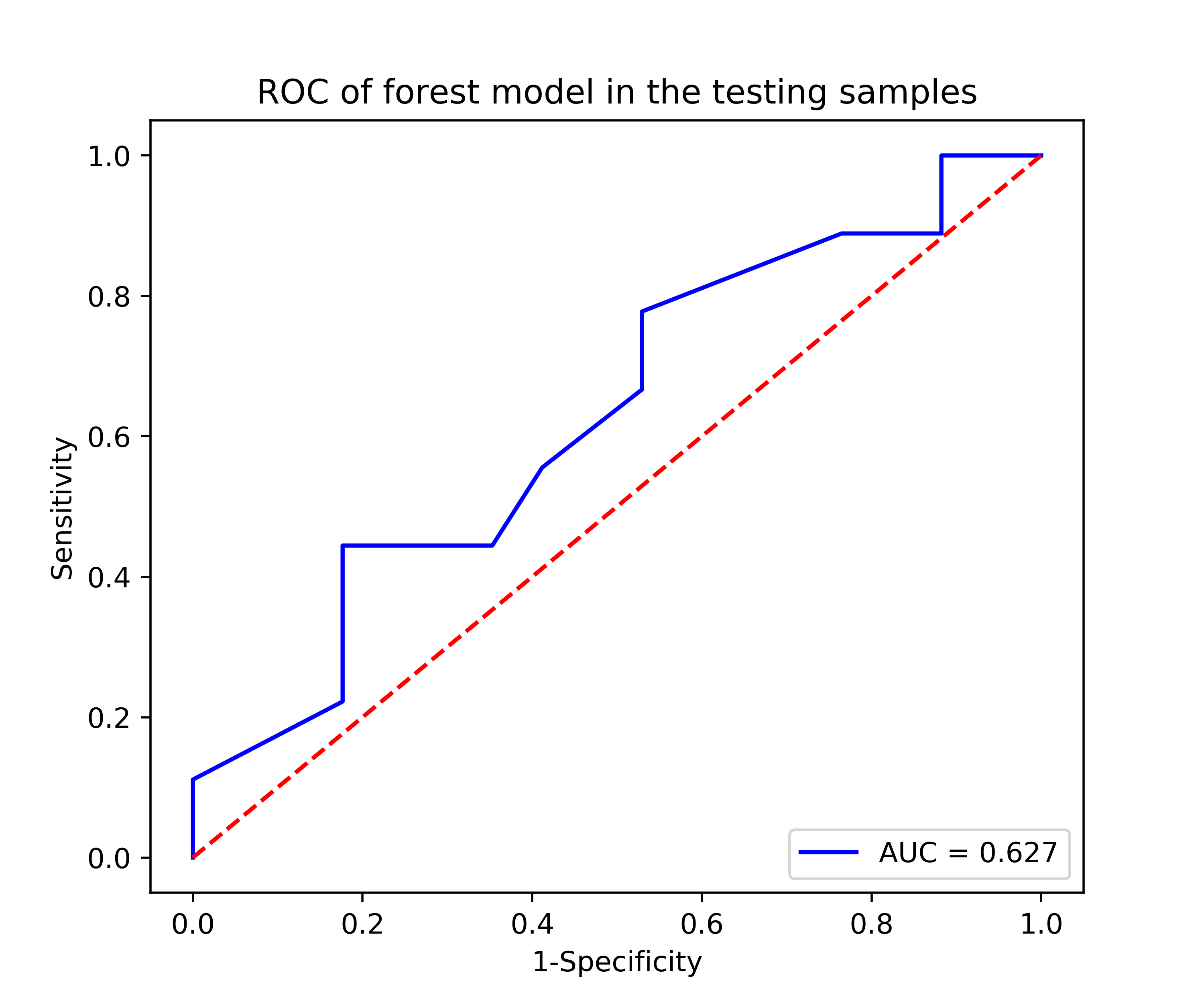


Decision Curve of Forest model in training samples:


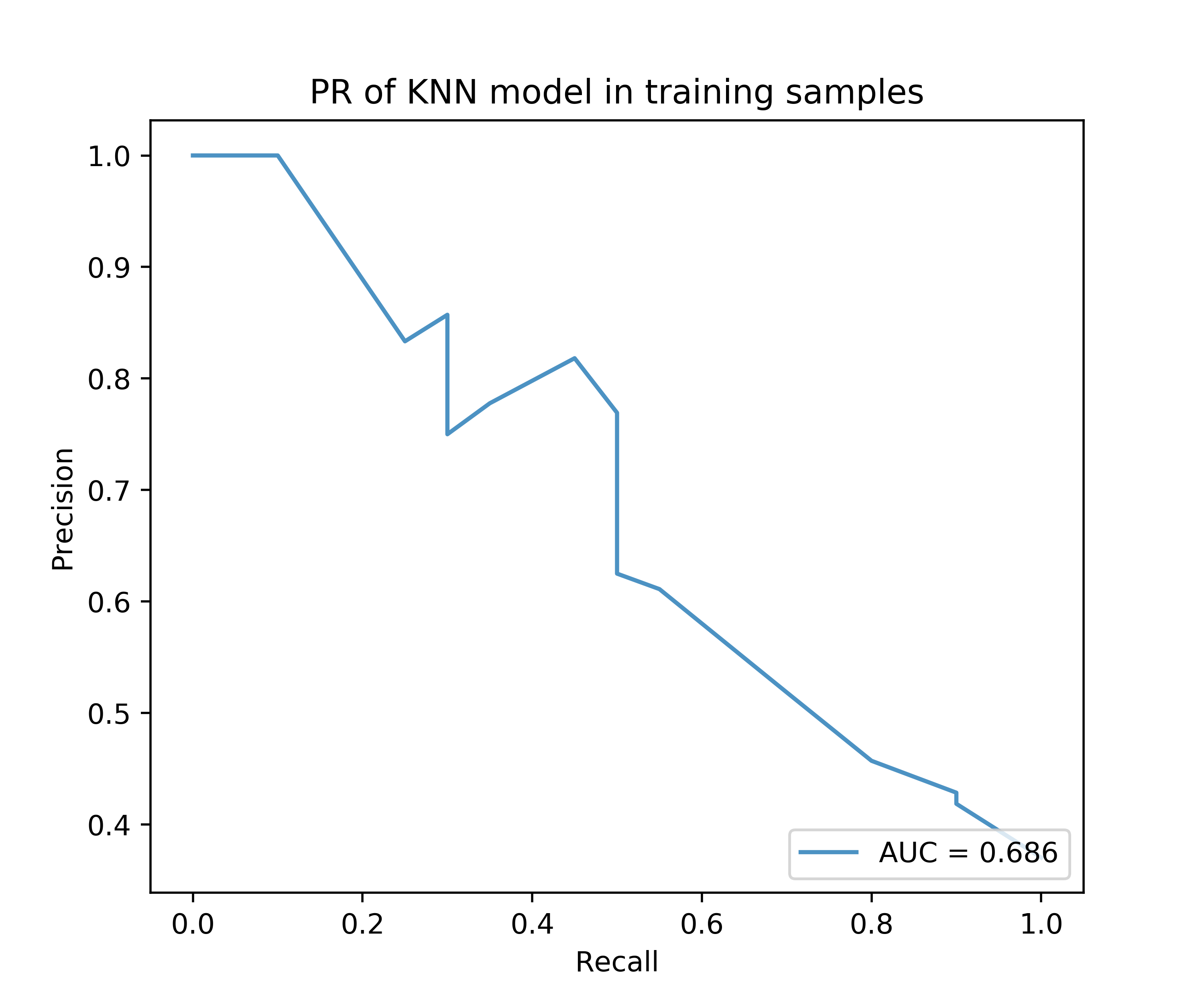


Decision Curve of Forest model in testing samples:


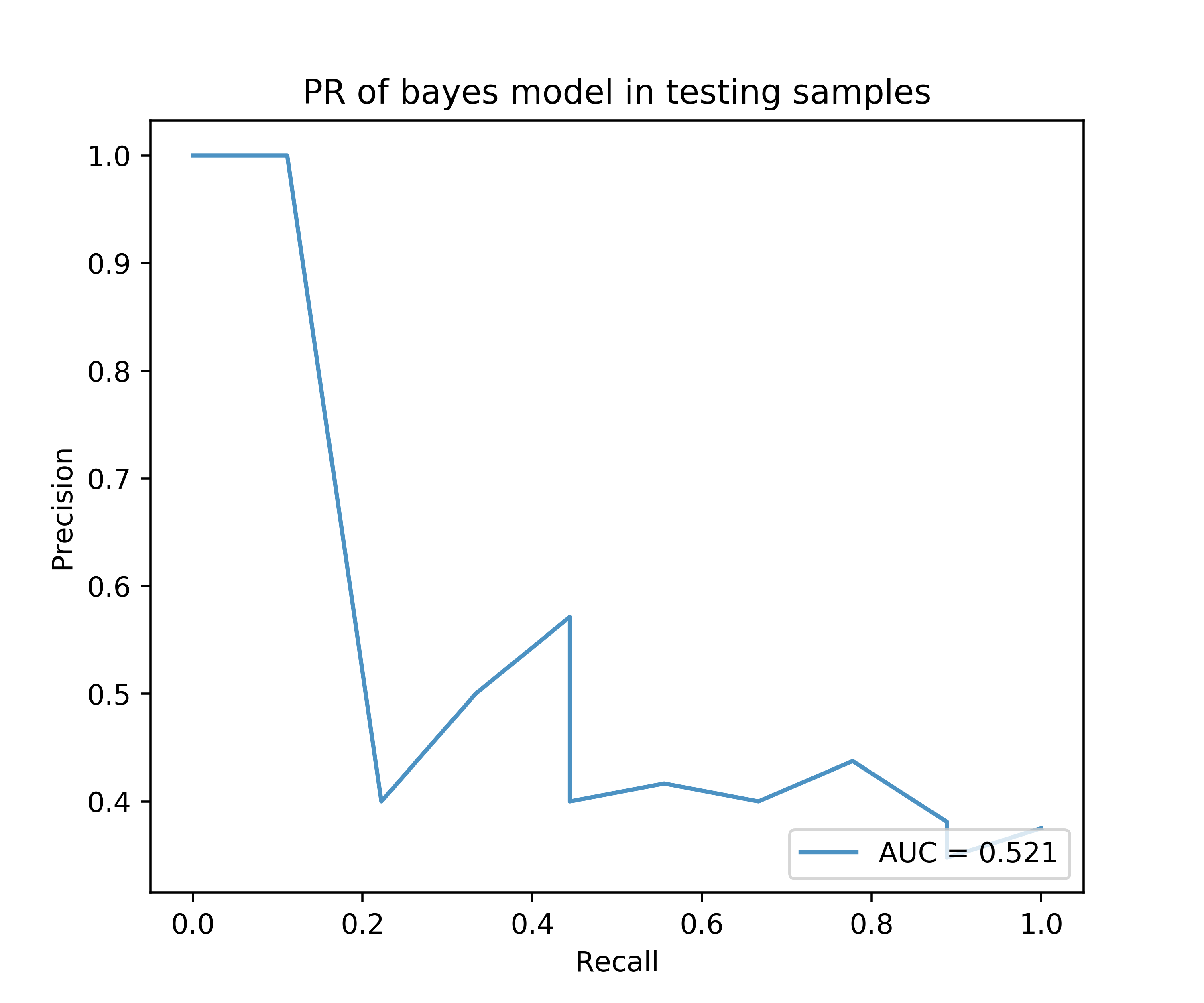


**Radiomics analysis process of K-nearest neighbor.**

Summary Report

# The summary report recognized the best model from all models your training, and summarized its training process.

As the result shown, the best model was KNN, its training process including: ['select a path', 'set a seed', 'seperate a data', 'input data', 'standardize data', 'select feature', 'select feature', 'select feature', 'machine_learning']. Detailed information is shown below:

# 1. Data: C:/Users/212768837/Desktop/zhuanyi_86ROI/new/result2.csv

# 2. Random seed: 18

# 3. Seperative rate: 0.7

Seperated report:

|  | Sum | Pos | Neg |
| --- | --- | --- | --- |
| data | 86 | 29 | 57 |
| train | 60 | 20 | 40 |
| test | 26 | 9 | 17 |

# 4. Input data

The method for filling the missing data: Median

The method for dealing with outliers: Median

# 5. The method for standardizing the data: Standardization

# 6. The method for selecting features: Variance

parameters setted: {'threshold': 1.0}
num of remained features: 426
remained features:
[['original_shape_Flatness']
 ['original_shape_LeastAxisLength']
 ['original_shape_Maximum2DDiameterSlice']
 ['original_shape_MeshVolume']
 ['original_shape_Sphericity']
 ['original_shape_SurfaceArea']
 ['original_shape_SurfaceVolumeRatio']
 ['original_firstorder_10Percentile']
 ['original_firstorder_90Percentile']
 ['original_firstorder_Energy']
 ['original_firstorder_Kurtosis']
 ['original_firstorder_Maximum']
 ['original_firstorder_MeanAbsoluteDeviation']
 ['original_firstorder_Median']
 ['original_firstorder_RobustMeanAbsoluteDeviation']
 ['original_firstorder_TotalEnergy']
 ['original_glcm_Autocorrelation']
 ['original_glcm_ClusterProminence']
 ['original_glcm_ClusterShade']
 ['original_glcm_Correlation']
 ['original_glcm_DifferenceVariance']
 ['original_glcm_Id']
 ['original_glcm_InverseVariance']
 ['original_glcm_JointAverage']
 ['original_glcm_JointEnergy']
 ['original_glcm_JointEntropy']
 ['original_glcm_MCC']
 ['original_glcm_SumAverage']
 ['original_glcm_SumEntropy']
 ['original_gldm_DependenceVariance']
 ['original_gldm_GrayLevelNonUniformity']
 ['original_gldm_GrayLevelVariance']
 ['original_gldm_LargeDependenceHighGrayLevelEmphasis']
 ['original_glrlm_GrayLevelNonUniformity']
 ['original_glrlm_GrayLevelNonUniformityNormalized']
 ['original_glrlm_HighGrayLevelRunEmphasis']
 ['original_glrlm_RunEntropy']
 ['original_glrlm_RunLengthNonUniformity']
 ['original_glrlm_RunPercentage']
 ['original_glrlm_ShortRunHighGrayLevelEmphasis']
 ['original_glrlm_ShortRunLowGrayLevelEmphasis']
 ['original_glszm_GrayLevelNonUniformity']
 ['original_glszm_GrayLevelNonUniformityNormalized']
 ['original_glszm_HighGrayLevelZoneEmphasis']
 ['original_glszm_LargeAreaEmphasis']
 ['original_glszm_LargeAreaHighGrayLevelEmphasis']
 ['original_glszm_LargeAreaLowGrayLevelEmphasis']
 ['original_glszm_LowGrayLevelZoneEmphasis']
 ['original_glszm_SizeZoneNonUniformity']
 ['original_glszm_SmallAreaEmphasis']
 ['original_glszm_SmallAreaHighGrayLevelEmphasis']
 ['original_glszm_SmallAreaLowGrayLevelEmphasis']
 ['original_glszm_ZonePercentage']
 ['original_ngtdm_Coarseness']
 ['original_ngtdm_Complexity']
 ['original_ngtdm_Contrast']
 ['original_ngtdm_Strength']
 ['wavelet-LLH_firstorder_10Percentile']
 ['wavelet-LLH_firstorder_90Percentile']
 ['wavelet-LLH_firstorder_Energy']
 ['wavelet-LLH_firstorder_Kurtosis']
 ['wavelet-LLH_firstorder_Maximum']
 ['wavelet-LLH_firstorder_Minimum']
 ['wavelet-LLH_firstorder_Range']
 ['wavelet-LLH_firstorder_TotalEnergy']
 ['wavelet-LLH_firstorder_Uniformity']
 ['wavelet-LLH_firstorder_Variance']
 ['wavelet-LLH_glcm_Autocorrelation']
 ['wavelet-LLH_glcm_Contrast']
 ['wavelet-LLH_glcm_Correlation']
 ['wavelet-LLH_glcm_Id']
 ['wavelet-LLH_glcm_Idm']
 ['wavelet-LLH_glcm_Imc1']
 ['wavelet-LLH_glcm_Imc2']
 ['wavelet-LLH_glcm_InverseVariance']
 ['wavelet-LLH_glcm_JointAverage']
 ['wavelet-LLH_glcm_JointEnergy']
 ['wavelet-LLH_glcm_JointEntropy']
 ['wavelet-LLH_glcm_SumAverage']
 ['wavelet-LLH_glcm_SumSquares']
 ['wavelet-LLH_gldm_DependenceNonUniformity']
 ['wavelet-LLH_gldm_DependenceNonUniformityNormalized']
 ['wavelet-LLH_gldm_DependenceVariance']
 ['wavelet-LLH_gldm_LargeDependenceEmphasis']
 ['wavelet-LLH_gldm_LargeDependenceHighGrayLevelEmphasis']
 ['wavelet-LLH_gldm_LowGrayLevelEmphasis']
 ['wavelet-LLH_gldm_SmallDependenceEmphasis']
 ['wavelet-LLH_glrlm_GrayLevelNonUniformity']
 ['wavelet-LLH_glrlm_GrayLevelNonUniformityNormalized']
 ['wavelet-LLH_glrlm_HighGrayLevelRunEmphasis']
 ['wavelet-LLH_glrlm_LongRunEmphasis']
 ['wavelet-LLH_glrlm_LongRunLowGrayLevelEmphasis']
 ['wavelet-LLH_glrlm_RunLengthNonUniformityNormalized']
 ['wavelet-LLH_glrlm_RunVariance']
 ['wavelet-LLH_glrlm_ShortRunEmphasis']
 ['wavelet-LLH_glrlm_ShortRunHighGrayLevelEmphasis']
 ['wavelet-LLH_glszm_HighGrayLevelZoneEmphasis']
 ['wavelet-LLH_glszm_LargeAreaEmphasis']
 ['wavelet-LLH_glszm_LargeAreaHighGrayLevelEmphasis']
 ['wavelet-LLH_glszm_SizeZoneNonUniformity']
 ['wavelet-LLH_glszm_SizeZoneNonUniformityNormalized']
 ['wavelet-LLH_glszm_SmallAreaEmphasis']
 ['wavelet-LLH_glszm_SmallAreaHighGrayLevelEmphasis']
 ['wavelet-LLH_ngtdm_Busyness']
 ['wavelet-LLH_ngtdm_Coarseness']
 ['wavelet-LLH_ngtdm_Complexity']
 ['wavelet-LLH_ngtdm_Contrast']
 ['wavelet-LHL_firstorder_10Percentile']
 ['wavelet-LHL_firstorder_90Percentile']
 ['wavelet-LHL_firstorder_Kurtosis']
 ['wavelet-LHL_firstorder_Median']
 ['wavelet-LHL_firstorder_RobustMeanAbsoluteDeviation']
 ['wavelet-LHL_firstorder_Uniformity']
 ['wavelet-LHL_glcm_Autocorrelation']
 ['wavelet-LHL_glcm_Correlation']
 ['wavelet-LHL_glcm_Id']
 ['wavelet-LHL_glcm_Idmn']
 ['wavelet-LHL_glcm_Imc1']
 ['wavelet-LHL_glcm_Imc2']
 ['wavelet-LHL_glcm_InverseVariance']
 ['wavelet-LHL_glcm_MCC']
 ['wavelet-LHL_glcm_SumEntropy']
 ['wavelet-LHL_glcm_SumSquares']
 ['wavelet-LHL_gldm_DependenceEntropy']
 ['wavelet-LHL_gldm_DependenceNonUniformity']
 ['wavelet-LHL_gldm_DependenceVariance']
 ['wavelet-LHL_gldm_GrayLevelVariance']
 ['wavelet-LHL_gldm_HighGrayLevelEmphasis']
 ['wavelet-LHL_gldm_LargeDependenceEmphasis']
 ['wavelet-LHL_gldm_SmallDependenceEmphasis']
 ['wavelet-LHL_gldm_SmallDependenceHighGrayLevelEmphasis']
 ['wavelet-LHL_gldm_SmallDependenceLowGrayLevelEmphasis']
 ['wavelet-LHL_glrlm_GrayLevelNonUniformity']
 ['wavelet-LHL_glrlm_GrayLevelNonUniformityNormalized']
 ['wavelet-LHL_glrlm_GrayLevelVariance']
 ['wavelet-LHL_glrlm_LongRunLowGrayLevelEmphasis']
 ['wavelet-LHL_glrlm_LowGrayLevelRunEmphasis']
 ['wavelet-LHL_glrlm_RunEntropy']
 ['wavelet-LHL_glrlm_RunLengthNonUniformity']
 ['wavelet-LHL_glrlm_ShortRunEmphasis']
 ['wavelet-LHL_glszm_GrayLevelVariance']
 ['wavelet-LHL_glszm_HighGrayLevelZoneEmphasis']
 ['wavelet-LHL_glszm_LargeAreaEmphasis']
 ['wavelet-LHL_glszm_LargeAreaHighGrayLevelEmphasis']
 ['wavelet-LHL_glszm_LowGrayLevelZoneEmphasis']
 ['wavelet-LHL_glszm_SizeZoneNonUniformityNormalized']
 ['wavelet-LHL_glszm_SmallAreaLowGrayLevelEmphasis']
 ['wavelet-LHL_glszm_ZoneEntropy']
 ['wavelet-LHL_glszm_ZonePercentage']
 ['wavelet-LHL_glszm_ZoneVariance']
 ['wavelet-LHL_ngtdm_Coarseness']
 ['wavelet-LHH_firstorder_10Percentile']
 ['wavelet-LHH_firstorder_90Percentile']
 ['wavelet-LHH_firstorder_Energy']
 ['wavelet-LHH_firstorder_InterquartileRange']
 ['wavelet-LHH_firstorder_Kurtosis']
 ['wavelet-LHH_firstorder_MeanAbsoluteDeviation']
 ['wavelet-LHH_firstorder_Median']
 ['wavelet-LHH_firstorder_Range']
 ['wavelet-LHH_firstorder_RobustMeanAbsoluteDeviation']
 ['wavelet-LHH_firstorder_TotalEnergy']
 ['wavelet-LHH_firstorder_Uniformity']
 ['wavelet-LHH_firstorder_Variance']
 ['wavelet-LHH_glcm_Autocorrelation']
 ['wavelet-LHH_glcm_ClusterTendency']
 ['wavelet-LHH_glcm_Correlation']
 ['wavelet-LHH_glcm_DifferenceEntropy']
 ['wavelet-LHH_glcm_DifferenceVariance']
 ['wavelet-LHH_glcm_Idm']
 ['wavelet-LHH_glcm_Imc2']
 ['wavelet-LHH_glcm_JointEnergy']
 ['wavelet-LHH_glcm_JointEntropy']
 ['wavelet-LHH_glcm_MCC']
 ['wavelet-LHH_glcm_MaximumProbability']
 ['wavelet-LHH_glcm_SumSquares']
 ['wavelet-LHH_gldm_DependenceEntropy']
 ['wavelet-LHH_gldm_DependenceNonUniformityNormalized']
 ['wavelet-LHH_gldm_DependenceVariance']
 ['wavelet-LHH_gldm_GrayLevelVariance']
 ['wavelet-LHH_gldm_HighGrayLevelEmphasis']
 ['wavelet-LHH_gldm_LargeDependenceEmphasis']
 ['wavelet-LHH_gldm_LargeDependenceHighGrayLevelEmphasis']
 ['wavelet-LHH_gldm_SmallDependenceLowGrayLevelEmphasis']
 ['wavelet-LHH_glrlm_GrayLevelNonUniformityNormalized']
 ['wavelet-LHH_glrlm_GrayLevelVariance']
 ['wavelet-LHH_glrlm_LongRunHighGrayLevelEmphasis']
 ['wavelet-LHH_glrlm_LongRunLowGrayLevelEmphasis']
 ['wavelet-LHH_glrlm_RunEntropy']
 ['wavelet-LHH_glrlm_RunVariance']
 ['wavelet-LHH_glrlm_ShortRunEmphasis']
 ['wavelet-LHH_glrlm_ShortRunHighGrayLevelEmphasis']
 ['wavelet-LHH_glszm_GrayLevelNonUniformity']
 ['wavelet-LHH_glszm_GrayLevelNonUniformityNormalized']
 ['wavelet-LHH_glszm_LargeAreaLowGrayLevelEmphasis']
 ['wavelet-LHH_glszm_LowGrayLevelZoneEmphasis']
 ['wavelet-LHH_glszm_SizeZoneNonUniformityNormalized']
 ['wavelet-LHH_glszm_SmallAreaHighGrayLevelEmphasis']
 ['wavelet-LHH_glszm_SmallAreaLowGrayLevelEmphasis']
 ['wavelet-LHH_glszm_ZonePercentage']
 ['wavelet-LHH_glszm_ZoneVariance']
 ['wavelet-LHH_ngtdm_Busyness']
 ['wavelet-LHH_ngtdm_Coarseness']
 ['wavelet-LHH_ngtdm_Contrast']
 ['wavelet-HLL_firstorder_90Percentile']
 ['wavelet-HLL_firstorder_InterquartileRange']
 ['wavelet-HLL_firstorder_MeanAbsoluteDeviation']
 ['wavelet-HLL_firstorder_Mean']
 ['wavelet-HLL_firstorder_Median']
 ['wavelet-HLL_firstorder_Minimum']
 ['wavelet-HLL_firstorder_Range']
 ['wavelet-HLL_firstorder_RootMeanSquared']
 ['wavelet-HLL_firstorder_Variance']
 ['wavelet-HLL_glcm_ClusterProminence']
 ['wavelet-HLL_glcm_ClusterShade']
 ['wavelet-HLL_glcm_Contrast']
 ['wavelet-HLL_glcm_Correlation']
 ['wavelet-HLL_glcm_DifferenceAverage']
 ['wavelet-HLL_glcm_DifferenceVariance']
 ['wavelet-HLL_glcm_Id']
 ['wavelet-HLL_glcm_Idm']
 ['wavelet-HLL_glcm_Idmn']
 ['wavelet-HLL_glcm_InverseVariance']
 ['wavelet-HLL_glcm_JointEnergy']
 ['wavelet-HLL_glcm_JointEntropy']
 ['wavelet-HLL_glcm_MaximumProbability']
 ['wavelet-HLL_glcm_SumEntropy']
 ['wavelet-HLL_gldm_DependenceNonUniformity']
 ['wavelet-HLL_gldm_DependenceVariance']
 ['wavelet-HLL_gldm_GrayLevelVariance']
 ['wavelet-HLL_gldm_HighGrayLevelEmphasis']
 ['wavelet-HLL_gldm_LargeDependenceEmphasis']
 ['wavelet-HLL_gldm_LargeDependenceHighGrayLevelEmphasis']
 ['wavelet-HLL_gldm_SmallDependenceEmphasis']
 ['wavelet-HLL_gldm_SmallDependenceHighGrayLevelEmphasis']
 ['wavelet-HLL_gldm_SmallDependenceLowGrayLevelEmphasis']
 ['wavelet-HLL_glrlm_GrayLevelNonUniformity']
 ['wavelet-HLL_glrlm_GrayLevelNonUniformityNormalized']
 ['wavelet-HLL_glrlm_GrayLevelVariance']
 ['wavelet-HLL_glrlm_HighGrayLevelRunEmphasis']
 ['wavelet-HLL_glrlm_LongRunEmphasis']
 ['wavelet-HLL_glrlm_LongRunHighGrayLevelEmphasis']
 ['wavelet-HLL_glrlm_RunEntropy']
 ['wavelet-HLL_glrlm_RunPercentage']
 ['wavelet-HLL_glszm_GrayLevelVariance']
 ['wavelet-HLL_glszm_LargeAreaEmphasis']
 ['wavelet-HLL_glszm_LargeAreaHighGrayLevelEmphasis']
 ['wavelet-HLL_glszm_SmallAreaEmphasis']
 ['wavelet-HLL_glszm_SmallAreaLowGrayLevelEmphasis']
 ['wavelet-HLL_glszm_ZoneEntropy']
 ['wavelet-HLL_glszm_ZoneVariance']
 ['wavelet-HLL_ngtdm_Strength']
 ['wavelet-HLH_firstorder_10Percentile']
 ['wavelet-HLH_firstorder_Energy']
 ['wavelet-HLH_firstorder_InterquartileRange']
 ['wavelet-HLH_firstorder_Median']
 ['wavelet-HLH_firstorder_Skewness']
 ['wavelet-HLH_firstorder_TotalEnergy']
 ['wavelet-HLH_glcm_ClusterProminence']
 ['wavelet-HLH_glcm_Contrast']
 ['wavelet-HLH_glcm_DifferenceEntropy']
 ['wavelet-HLH_glcm_DifferenceVariance']
 ['wavelet-HLH_glcm_Id']
 ['wavelet-HLH_glcm_Idm']
 ['wavelet-HLH_glcm_InverseVariance']
 ['wavelet-HLH_glcm_JointEntropy']
 ['wavelet-HLH_glcm_SumEntropy']
 ['wavelet-HLH_glcm_SumSquares']
 ['wavelet-HLH_gldm_DependenceVariance']
 ['wavelet-HLH_gldm_GrayLevelVariance']
 ['wavelet-HLH_gldm_LargeDependenceHighGrayLevelEmphasis']
 ['wavelet-HLH_gldm_SmallDependenceEmphasis']
 ['wavelet-HLH_gldm_SmallDependenceHighGrayLevelEmphasis']
 ['wavelet-HLH_gldm_SmallDependenceLowGrayLevelEmphasis']
 ['wavelet-HLH_glrlm_GrayLevelNonUniformity']
 ['wavelet-HLH_glrlm_GrayLevelVariance']
 ['wavelet-HLH_glrlm_HighGrayLevelRunEmphasis']
 ['wavelet-HLH_glrlm_LongRunEmphasis']
 ['wavelet-HLH_glrlm_LongRunHighGrayLevelEmphasis']
 ['wavelet-HLH_glrlm_RunPercentage']
 ['wavelet-HLH_glrlm_RunVariance']
 ['wavelet-HLH_glszm_HighGrayLevelZoneEmphasis']
 ['wavelet-HLH_glszm_LargeAreaLowGrayLevelEmphasis']
 ['wavelet-HLH_glszm_ZoneEntropy']
 ['wavelet-HLH_glszm_ZonePercentage']
 ['wavelet-HLH_glszm_ZoneVariance']
 ['wavelet-HLH_ngtdm_Coarseness']
 ['wavelet-HLH_ngtdm_Complexity']
 ['wavelet-HLH_ngtdm_Strength']
 ['wavelet-HHL_firstorder_Energy']
 ['wavelet-HHL_firstorder_Entropy']
 ['wavelet-HHL_firstorder_InterquartileRange']
 ['wavelet-HHL_firstorder_Kurtosis']
 ['wavelet-HHL_firstorder_Minimum']
 ['wavelet-HHL_firstorder_RootMeanSquared']
 ['wavelet-HHL_firstorder_TotalEnergy']
 ['wavelet-HHL_firstorder_Uniformity']
 ['wavelet-HHL_glcm_DifferenceEntropy']
 ['wavelet-HHL_glcm_Idm']
 ['wavelet-HHL_glcm_Idmn']
 ['wavelet-HHL_glcm_Idn']
 ['wavelet-HHL_glcm_InverseVariance']
 ['wavelet-HHL_glcm_JointEnergy']
 ['wavelet-HHL_glcm_JointEntropy']
 ['wavelet-HHL_glcm_MCC']
 ['wavelet-HHL_gldm_DependenceNonUniformityNormalized']
 ['wavelet-HHL_gldm_GrayLevelNonUniformity']
 ['wavelet-HHL_gldm_LargeDependenceEmphasis']
 ['wavelet-HHL_gldm_LowGrayLevelEmphasis']
 ['wavelet-HHL_gldm_SmallDependenceEmphasis']
 ['wavelet-HHL_gldm_SmallDependenceHighGrayLevelEmphasis']
 ['wavelet-HHL_gldm_SmallDependenceLowGrayLevelEmphasis']
 ['wavelet-HHL_glrlm_GrayLevelNonUniformityNormalized']
 ['wavelet-HHL_glrlm_GrayLevelVariance']
 ['wavelet-HHL_glrlm_LongRunHighGrayLevelEmphasis']
 ['wavelet-HHL_glrlm_LongRunLowGrayLevelEmphasis']
 ['wavelet-HHL_glrlm_LowGrayLevelRunEmphasis']
 ['wavelet-HHL_glrlm_ShortRunHighGrayLevelEmphasis']
 ['wavelet-HHL_glrlm_ShortRunLowGrayLevelEmphasis']
 ['wavelet-HHL_glszm_GrayLevelNonUniformityNormalized']
 ['wavelet-HHL_glszm_HighGrayLevelZoneEmphasis']
 ['wavelet-HHL_glszm_LargeAreaEmphasis']
 ['wavelet-HHL_glszm_LargeAreaHighGrayLevelEmphasis']
 ['wavelet-HHL_glszm_LowGrayLevelZoneEmphasis']
 ['wavelet-HHL_glszm_SmallAreaEmphasis']
 ['wavelet-HHL_glszm_SmallAreaHighGrayLevelEmphasis']
 ['wavelet-HHL_glszm_SmallAreaLowGrayLevelEmphasis']
 ['wavelet-HHL_glszm_ZoneEntropy']
 ['wavelet-HHL_glszm_ZoneVariance']
 ['wavelet-HHL_ngtdm_Busyness']
 ['wavelet-HHL_ngtdm_Contrast']
 ['wavelet-HHH_firstorder_10Percentile']
 ['wavelet-HHH_firstorder_90Percentile']
 ['wavelet-HHH_firstorder_Energy']
 ['wavelet-HHH_firstorder_Entropy']
 ['wavelet-HHH_firstorder_InterquartileRange']
 ['wavelet-HHH_firstorder_Maximum']
 ['wavelet-HHH_firstorder_MeanAbsoluteDeviation']
 ['wavelet-HHH_firstorder_Median']
 ['wavelet-HHH_firstorder_Minimum']
 ['wavelet-HHH_firstorder_Range']
 ['wavelet-HHH_firstorder_RootMeanSquared']
 ['wavelet-HHH_firstorder_TotalEnergy']
 ['wavelet-HHH_firstorder_Variance']
 ['wavelet-HHH_glcm_ClusterShade']
 ['wavelet-HHH_glcm_Correlation']
 ['wavelet-HHH_glcm_DifferenceEntropy']
 ['wavelet-HHH_glcm_DifferenceVariance']
 ['wavelet-HHH_glcm_Imc1']
 ['wavelet-HHH_glcm_JointEnergy']
 ['wavelet-HHH_glcm_JointEntropy']
 ['wavelet-HHH_glcm_MaximumProbability']
 ['wavelet-HHH_glcm_SumEntropy']
 ['wavelet-HHH_glcm_SumSquares']
 ['wavelet-HHH_gldm_DependenceEntropy']
 ['wavelet-HHH_gldm_DependenceNonUniformity']
 ['wavelet-HHH_gldm_DependenceNonUniformityNormalized']
 ['wavelet-HHH_gldm_GrayLevelNonUniformity']
 ['wavelet-HHH_gldm_GrayLevelVariance']
 ['wavelet-HHH_gldm_LargeDependenceHighGrayLevelEmphasis']
 ['wavelet-HHH_gldm_LargeDependenceLowGrayLevelEmphasis']
 ['wavelet-HHH_gldm_LowGrayLevelEmphasis']
 ['wavelet-HHH_gldm_SmallDependenceEmphasis']
 ['wavelet-HHH_gldm_SmallDependenceHighGrayLevelEmphasis']
 ['wavelet-HHH_gldm_SmallDependenceLowGrayLevelEmphasis']
 ['wavelet-HHH_glrlm_GrayLevelNonUniformityNormalized']
 ['wavelet-HHH_glrlm_GrayLevelVariance']
 ['wavelet-HHH_glrlm_HighGrayLevelRunEmphasis']
 ['wavelet-HHH_glrlm_LongRunHighGrayLevelEmphasis']
 ['wavelet-HHH_glrlm_LongRunLowGrayLevelEmphasis']
 ['wavelet-HHH_glrlm_LowGrayLevelRunEmphasis']
 ['wavelet-HHH_glrlm_RunEntropy']
 ['wavelet-HHH_glrlm_RunLengthNonUniformityNormalized']
 ['wavelet-HHH_glrlm_ShortRunEmphasis']
 ['wavelet-HHH_glszm_GrayLevelNonUniformity']
 ['wavelet-HHH_glszm_GrayLevelNonUniformityNormalized']
 ['wavelet-HHH_glszm_GrayLevelVariance']
 ['wavelet-HHH_glszm_LargeAreaHighGrayLevelEmphasis']
 ['wavelet-HHH_glszm_LargeAreaLowGrayLevelEmphasis']
 ['wavelet-HHH_glszm_LowGrayLevelZoneEmphasis']
 ['wavelet-HHH_glszm_SizeZoneNonUniformityNormalized']
 ['wavelet-HHH_glszm_ZonePercentage']
 ['wavelet-HHH_ngtdm_Complexity']
 ['wavelet-LLL_firstorder_90Percentile']
 ['wavelet-LLL_firstorder_Energy']
 ['wavelet-LLL_firstorder_Entropy']
 ['wavelet-LLL_firstorder_InterquartileRange']
 ['wavelet-LLL_firstorder_Kurtosis']
 ['wavelet-LLL_firstorder_MeanAbsoluteDeviation']
 ['wavelet-LLL_firstorder_Minimum']
 ['wavelet-LLL_firstorder_RootMeanSquared']
 ['wavelet-LLL_firstorder_Skewness']
 ['wavelet-LLL_firstorder_TotalEnergy']
 ['wavelet-LLL_glcm_ClusterShade']
 ['wavelet-LLL_glcm_Contrast']
 ['wavelet-LLL_glcm_Correlation']
 ['wavelet-LLL_glcm_Idmn']
 ['wavelet-LLL_glcm_MCC']
 ['wavelet-LLL_glcm_SumEntropy']
 ['wavelet-LLL_glcm_SumSquares']
 ['wavelet-LLL_gldm_GrayLevelNonUniformity']
 ['wavelet-LLL_gldm_GrayLevelVariance']
 ['wavelet-LLL_gldm_LargeDependenceEmphasis']
 ['wavelet-LLL_gldm_LargeDependenceLowGrayLevelEmphasis']
 ['wavelet-LLL_gldm_LowGrayLevelEmphasis']
 ['wavelet-LLL_gldm_SmallDependenceEmphasis']
 ['wavelet-LLL_gldm_SmallDependenceHighGrayLevelEmphasis']
 ['wavelet-LLL_glrlm_GrayLevelNonUniformity']
 ['wavelet-LLL_glrlm_GrayLevelNonUniformityNormalized']
 ['wavelet-LLL_glrlm_GrayLevelVariance']
 ['wavelet-LLL_glrlm_HighGrayLevelRunEmphasis']
 ['wavelet-LLL_glrlm_RunEntropy']
 ['wavelet-LLL_glrlm_RunLengthNonUniformity']
 ['wavelet-LLL_glrlm_RunPercentage']
 ['wavelet-LLL_glrlm_RunVariance']
 ['wavelet-LLL_glrlm_ShortRunEmphasis']
 ['wavelet-LLL_glszm_GrayLevelNonUniformity']
 ['wavelet-LLL_glszm_GrayLevelVariance']
 ['wavelet-LLL_glszm_HighGrayLevelZoneEmphasis']
 ['wavelet-LLL_glszm_LargeAreaEmphasis']
 ['wavelet-LLL_glszm_LargeAreaHighGrayLevelEmphasis']
 ['wavelet-LLL_glszm_LowGrayLevelZoneEmphasis']
 ['wavelet-LLL_glszm_SizeZoneNonUniformityNormalized']
 ['wavelet-LLL_glszm_SmallAreaEmphasis']
 ['wavelet-LLL_glszm_ZonePercentage']
 ['wavelet-LLL_ngtdm_Busyness']
 ['wavelet-LLL_ngtdm_Strength']]

Heatmap of the model in the training samples:


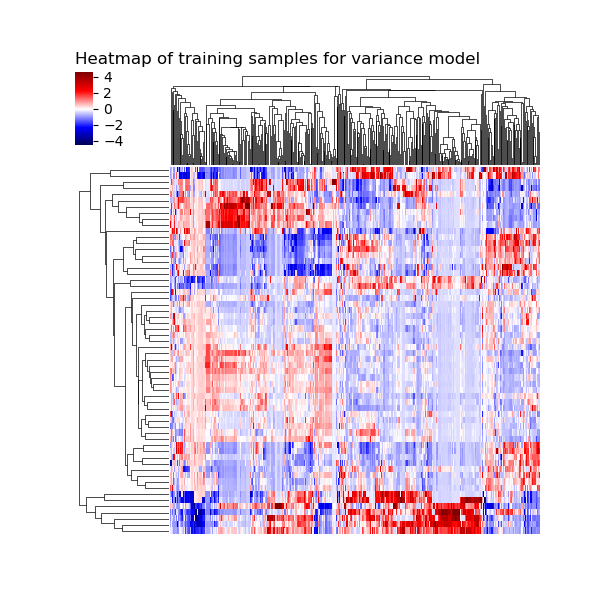


Heatmap of the model in the testing samples:


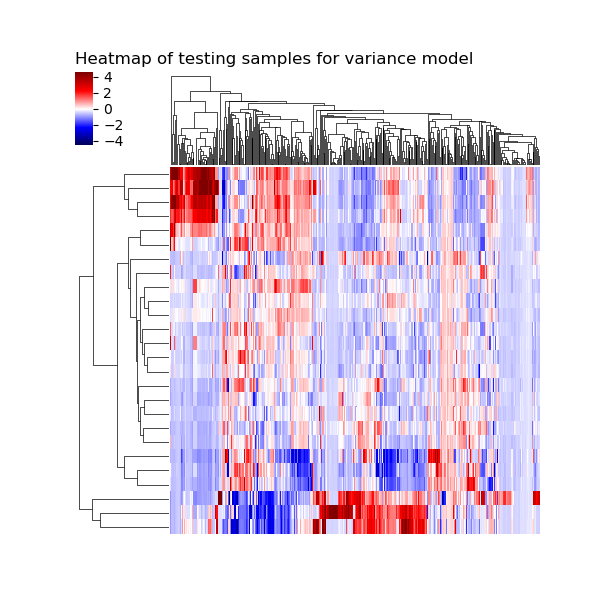


Correlation coefficient figure of the training samples

Correlation coefficient figure of the testing samples

# 7. The method for selecting features: Correlation_xx

parameters setted: {'cutoff': 0.7}
num of remained features: 85
remained features:
[['original_shape_Flatness']
 ['original_shape_Sphericity']
 ['original_glcm_ClusterShade']
 ['original_glcm_InverseVariance']
 ['original_glcm_JointEnergy']
 ['original_glszm_LargeAreaEmphasis']
 ['original_glszm_LowGrayLevelZoneEmphasis']
 ['original_ngtdm_Contrast']
 ['wavelet-LLH_firstorder_90Percentile']
 ['wavelet-LLH_firstorder_Kurtosis']
 ['wavelet-LLH_firstorder_Maximum']
 ['wavelet-LLH_glcm_Autocorrelation']
 ['wavelet-LLH_glcm_Correlation']
 ['wavelet-LLH_glcm_Imc2']
 ['wavelet-LLH_glcm_InverseVariance']
 ['wavelet-LLH_gldm_DependenceVariance']
 ['wavelet-LLH_gldm_LargeDependenceHighGrayLevelEmphasis']
 ['wavelet-LLH_gldm_LowGrayLevelEmphasis']
 ['wavelet-LLH_glrlm_LongRunLowGrayLevelEmphasis']
 ['wavelet-LLH_ngtdm_Contrast']
 ['wavelet-LHL_firstorder_90Percentile']
 ['wavelet-LHL_firstorder_Kurtosis']
 ['wavelet-LHL_glcm_Correlation']
 ['wavelet-LHL_glcm_InverseVariance']
 ['wavelet-LHL_glcm_MCC']
 ['wavelet-LHL_gldm_DependenceEntropy']
 ['wavelet-LHL_gldm_SmallDependenceHighGrayLevelEmphasis']
 ['wavelet-LHL_glrlm_LongRunLowGrayLevelEmphasis']
 ['wavelet-LHL_glszm_GrayLevelVariance']
 ['wavelet-LHL_glszm_LargeAreaHighGrayLevelEmphasis']
 ['wavelet-LHL_glszm_SmallAreaLowGrayLevelEmphasis']
 ['wavelet-LHH_firstorder_Median']
 ['wavelet-LHH_firstorder_TotalEnergy']
 ['wavelet-LHH_glcm_Correlation']
 ['wavelet-LHH_gldm_DependenceEntropy']
 ['wavelet-LHH_gldm_DependenceNonUniformityNormalized']
 ['wavelet-LHH_glrlm_LongRunLowGrayLevelEmphasis']
 ['wavelet-LHH_glszm_GrayLevelNonUniformity']
 ['wavelet-LHH_glszm_SizeZoneNonUniformityNormalized']
 ['wavelet-LHH_glszm_SmallAreaLowGrayLevelEmphasis']
 ['wavelet-LHH_ngtdm_Busyness']
 ['wavelet-LHH_ngtdm_Contrast']
 ['wavelet-HLL_firstorder_90Percentile']
 ['wavelet-HLL_glcm_ClusterProminence']
 ['wavelet-HLL_glcm_ClusterShade']
 ['wavelet-HLL_glcm_Correlation']
 ['wavelet-HLL_glrlm_LongRunHighGrayLevelEmphasis']
 ['wavelet-HLL_glrlm_RunEntropy']
 ['wavelet-HLL_glszm_LargeAreaHighGrayLevelEmphasis']
 ['wavelet-HLL_glszm_SmallAreaLowGrayLevelEmphasis']
 ['wavelet-HLL_ngtdm_Strength']
 ['wavelet-HLH_firstorder_Skewness']
 ['wavelet-HLH_glcm_InverseVariance']
 ['wavelet-HLH_gldm_DependenceVariance']
 ['wavelet-HHL_glcm_Idm']
 ['wavelet-HHL_glcm_InverseVariance']
 ['wavelet-HHL_glcm_MCC']
 ['wavelet-HHL_gldm_DependenceNonUniformityNormalized']
 ['wavelet-HHL_gldm_SmallDependenceHighGrayLevelEmphasis']
 ['wavelet-HHL_glrlm_LongRunHighGrayLevelEmphasis']
 ['wavelet-HHL_glrlm_LongRunLowGrayLevelEmphasis']
 ['wavelet-HHL_glrlm_ShortRunHighGrayLevelEmphasis']
 ['wavelet-HHL_glszm_SmallAreaEmphasis']
 ['wavelet-HHL_glszm_SmallAreaLowGrayLevelEmphasis']
 ['wavelet-HHL_ngtdm_Busyness']
 ['wavelet-HHH_firstorder_InterquartileRange']
 ['wavelet-HHH_firstorder_Median']
 ['wavelet-HHH_glcm_ClusterShade']
 ['wavelet-HHH_glcm_Correlation']
 ['wavelet-HHH_glcm_Imc1']
 ['wavelet-HHH_gldm_DependenceEntropy']
 ['wavelet-HHH_glszm_GrayLevelNonUniformity']
 ['wavelet-HHH_glszm_GrayLevelNonUniformityNormalized']
 ['wavelet-HHH_glszm_LowGrayLevelZoneEmphasis']
 ['wavelet-HHH_glszm_SizeZoneNonUniformityNormalized']
 ['wavelet-HHH_glszm_ZonePercentage']
 ['wavelet-HHH_ngtdm_Complexity']
 ['wavelet-LLL_firstorder_Kurtosis']
 ['wavelet-LLL_firstorder_Minimum']
 ['wavelet-LLL_glcm_Contrast']
 ['wavelet-LLL_glcm_MCC']
 ['wavelet-LLL_gldm_LargeDependenceLowGrayLevelEmphasis']
 ['wavelet-LLL_glszm_GrayLevelVariance']
 ['wavelet-LLL_ngtdm_Busyness']
 ['wavelet-LLL_ngtdm_Strength']]

Heatmap of the model in the training samples:


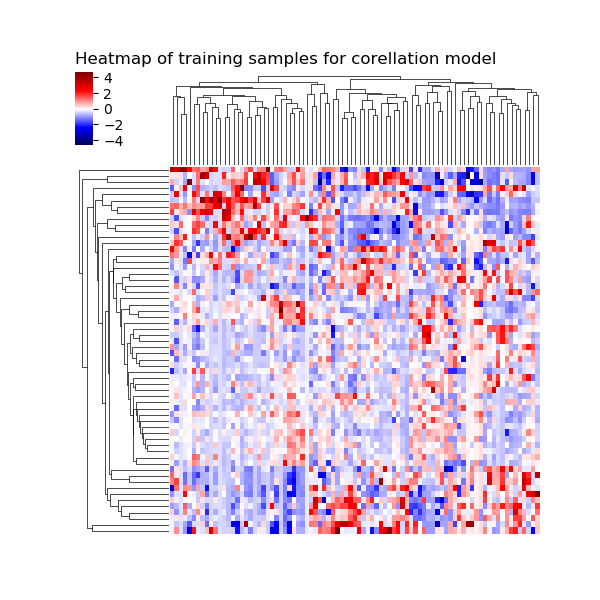


Heatmap of the model in the testing samples:


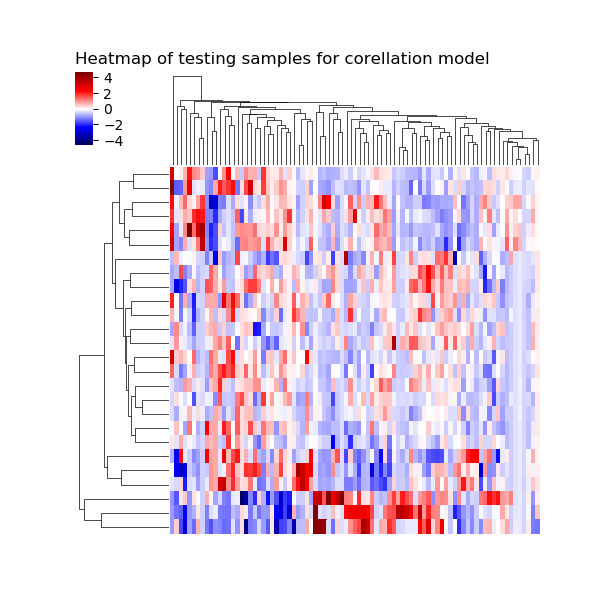


Correlation coefficient figure of the training samples

Correlation coefficient figure of the testing samples

# 8. The method for selecting features: MultiVariate_Logistic

parameters setted: {'P value for threshold in': 0.05, 'P value for threshold out': 0.1}
num of remained features: 4
remained features:
[['wavelet-HHH_glszm_SizeZoneNonUniformityNormalized']
 ['wavelet-LHH_glcm_Correlation']
 ['wavelet-HLL_glcm_ClusterShade']
 ['original_shape_Flatness']]

Statistical analysis of logistic multivariate analysis:

| feature | OR | 0.025 | 0.975 | P_value |
| --- | --- | --- | --- | --- |
| const | 0.317 | 0.145 | 0.694 | nan |
| wavelet-HHH_glszm_SizeZoneNonUniformityNormalized | 3.364 | 1.263 | 8.965 | 0.015 |
| wavelet-LHH_glcm_Correlation | 5.048 | 1.74 | 14.643 | 0.003 |
| wavelet-HLL_glcm_ClusterShade | 0.27 | 0.11 | 0.661 | 0.004 |
| original_shape_Flatness | 2.288 | 1.028 | 5.092 | 0.043 |

Heatmap of the model in the training samples:


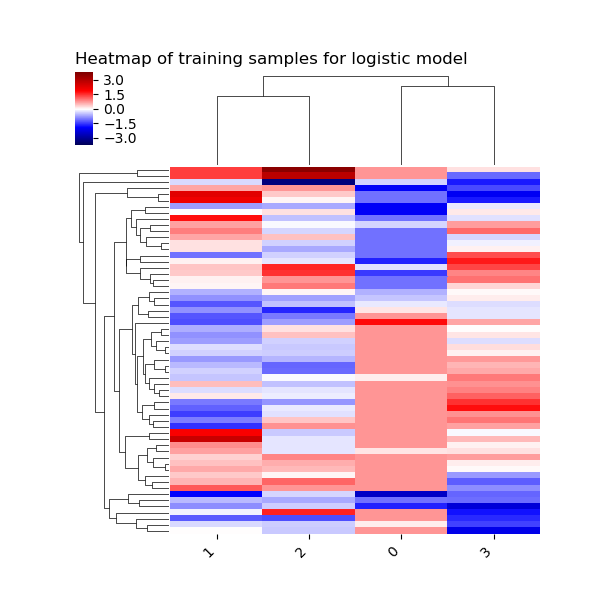


Heatmap of the model in the testing samples:


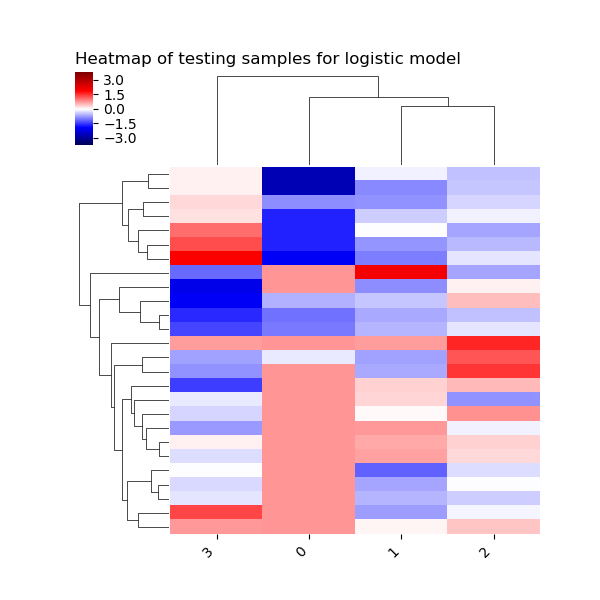


Correlation coefficient figure of the training samples


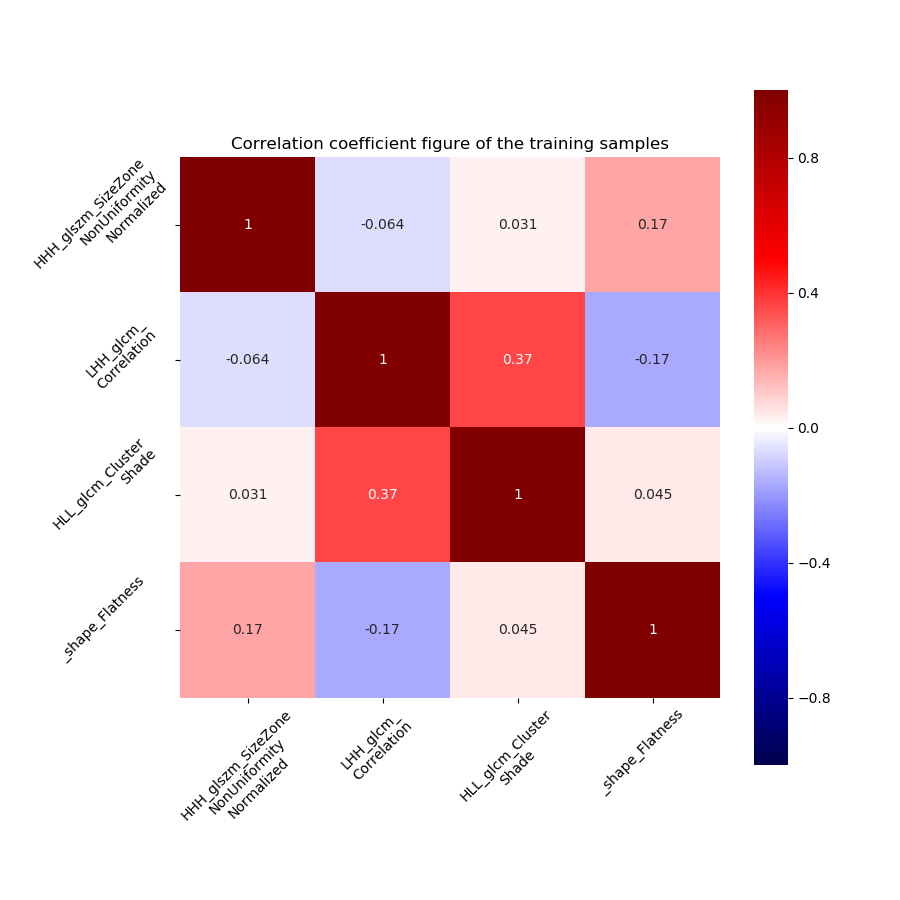


Correlation coefficient figure of the testing samples


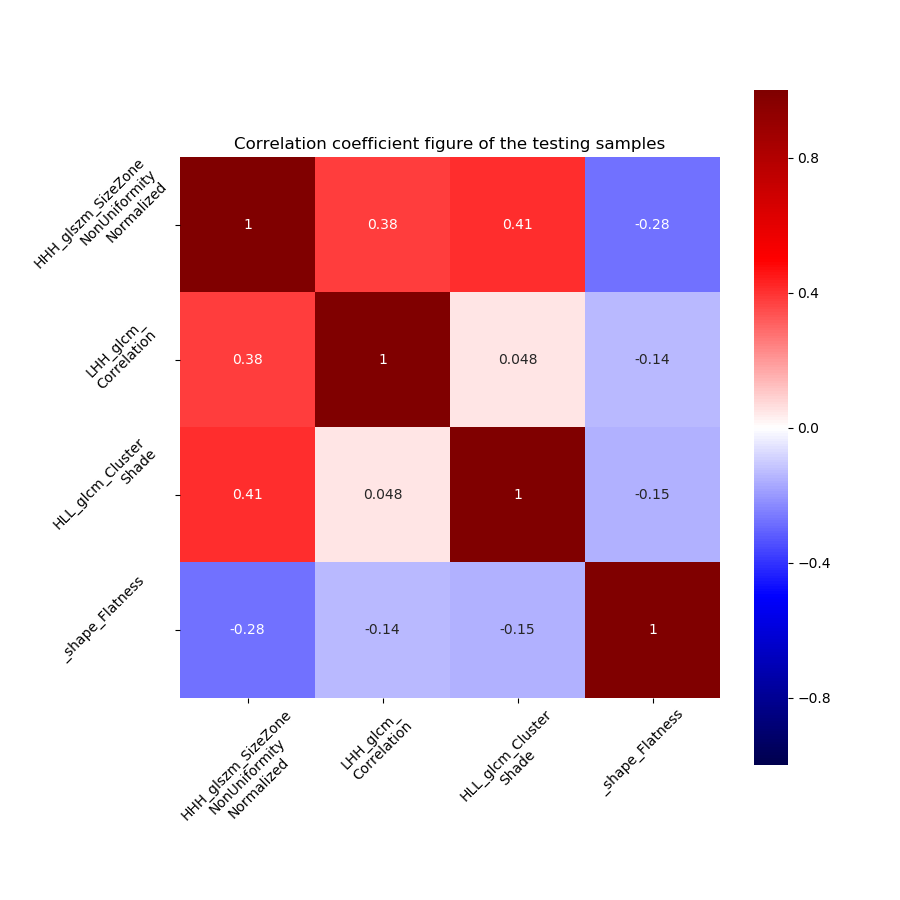


# 9. The best machine learning method: KNN

the mechine learning method: KNN

the parameters selection method and selected parameters:
 {'Method': 'auto', 'n_neighbors': 9}

the separate scores and total mean scores of model in each validation fold:
 {'scores': array([0.75 , 0.83333333, 0.75 , 0.75 , 0.75 ]), 'mean_score': 0.7666666666666667}

evaluation of the KNN model in the training and testing samples:

| Item | Train | Test |
| --- | --- | --- |
| Accuracy | 0.767 | 0.692 |
| f1_score | 0.667 | 0.556 |
| Recall | 0.7 | 0.556 |
| Precision | 0.636 | 0.556 |
| AUC | 0.817 (0.712, 0.907) | 0.748 (0.581, 0.895) |
| Sensitivity | 0.7 | 0.556 |
| Specificity | 0.8 | 0.765 |
| positive prediction | 0.636 | 0.556 |
| negative prediction | 0.842 | 0.765 |
| positive llr | 3.5 | 2.361 |
| negatice llr | 0.375 | 0.581 |

ROC of the KNN model in the training samples:


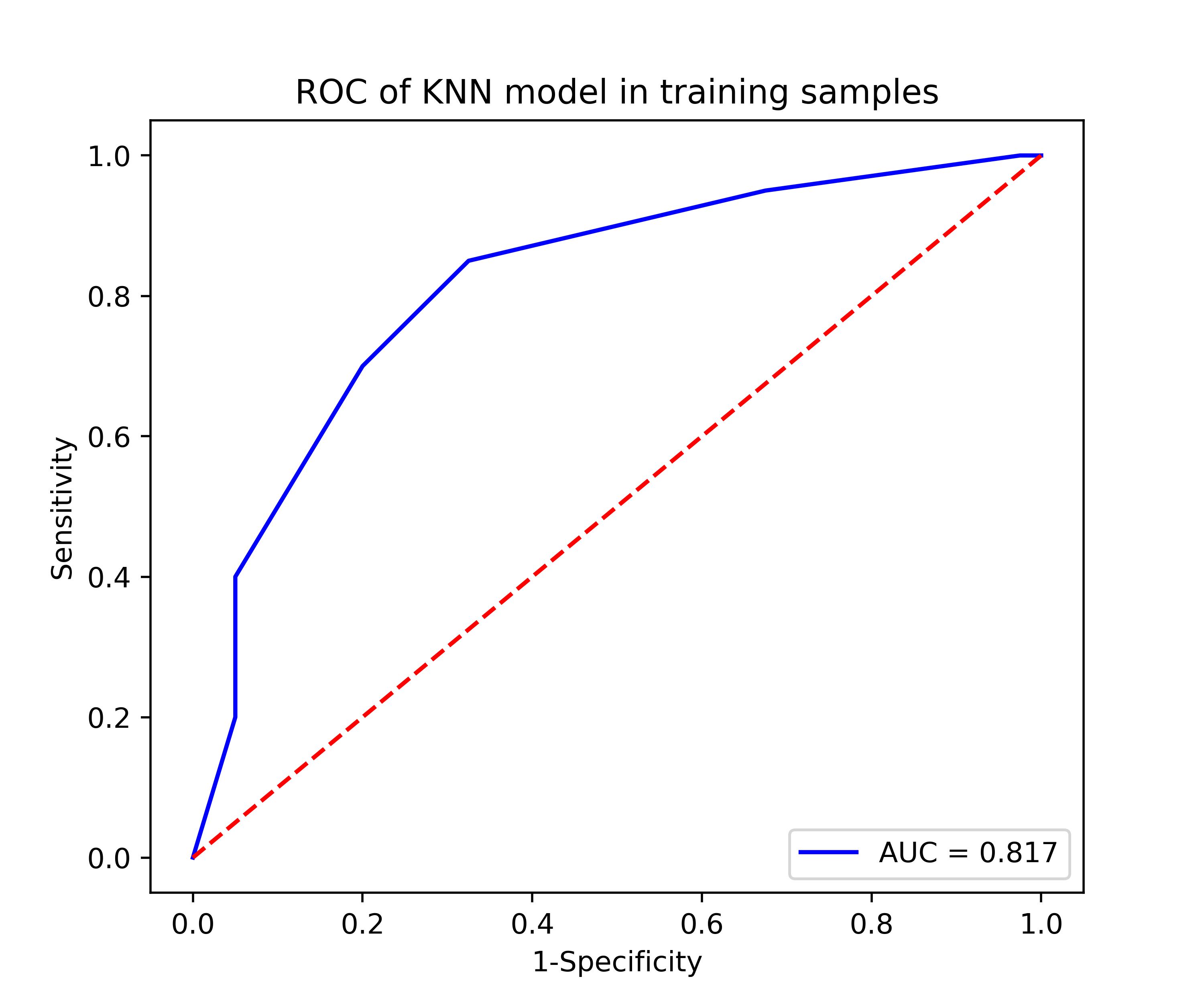


ROC of the KNN model in the testing samples:


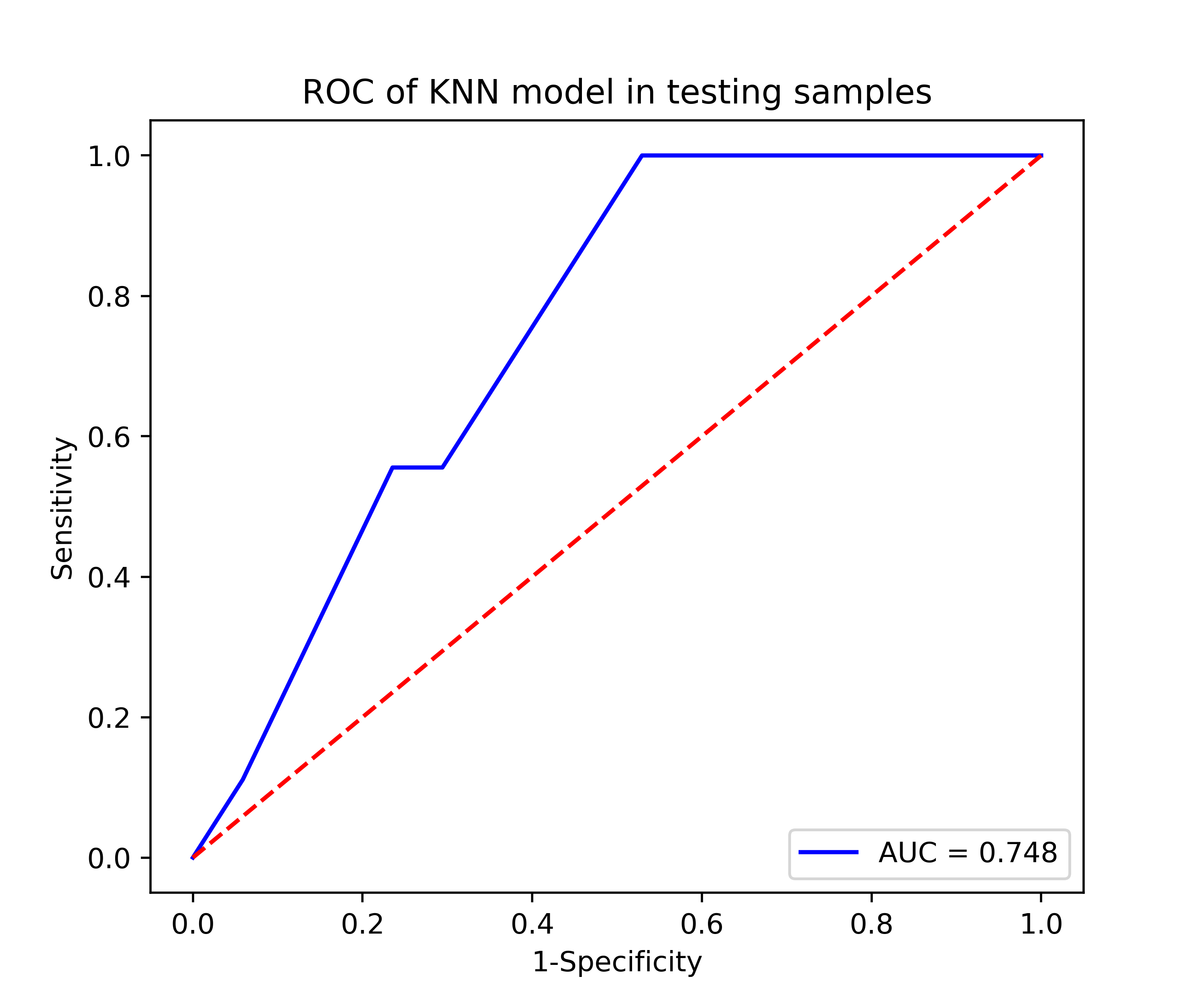


Decision Curve of KNN model in training samples:


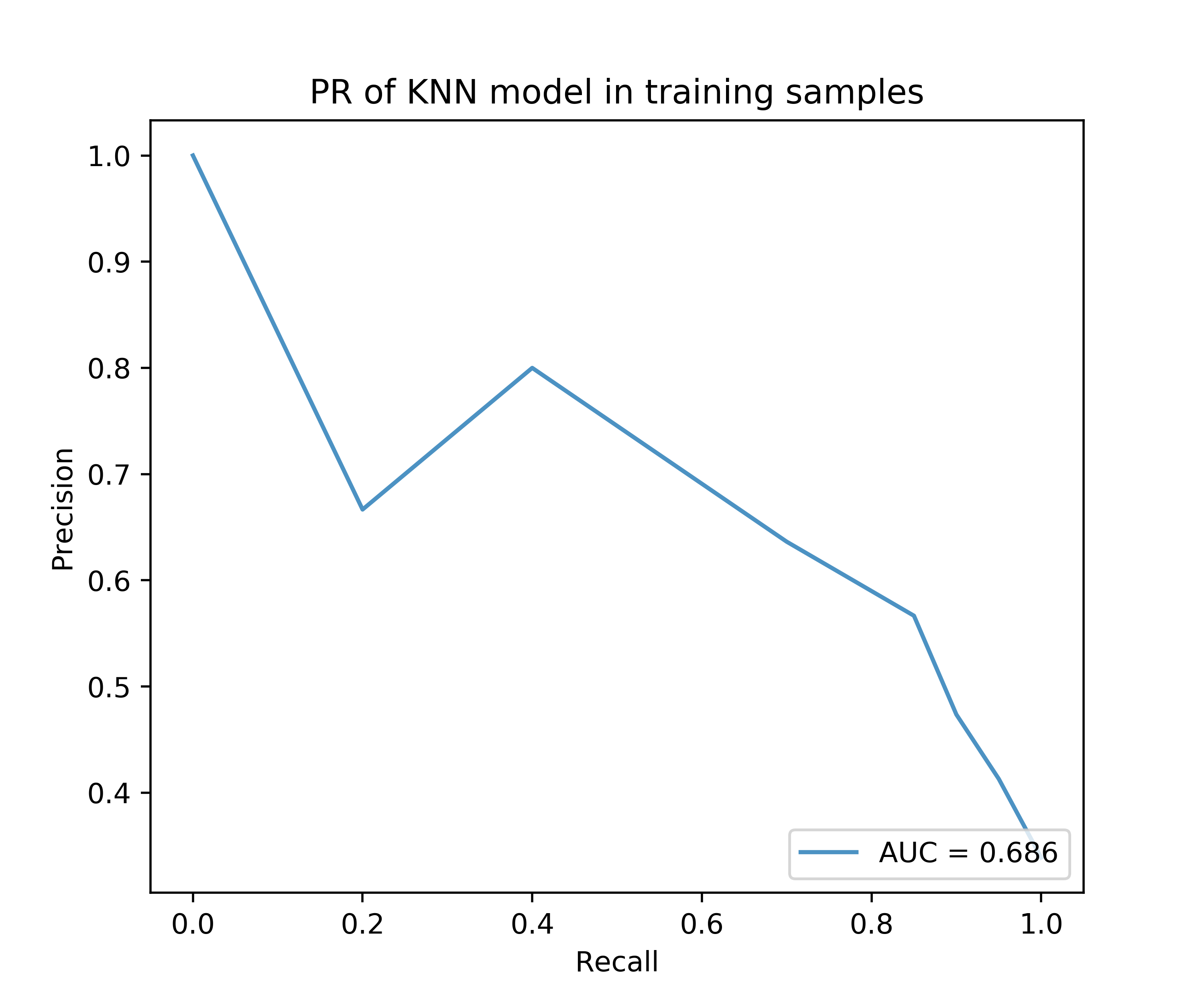


Decision Curve of KNN model in testing samples:


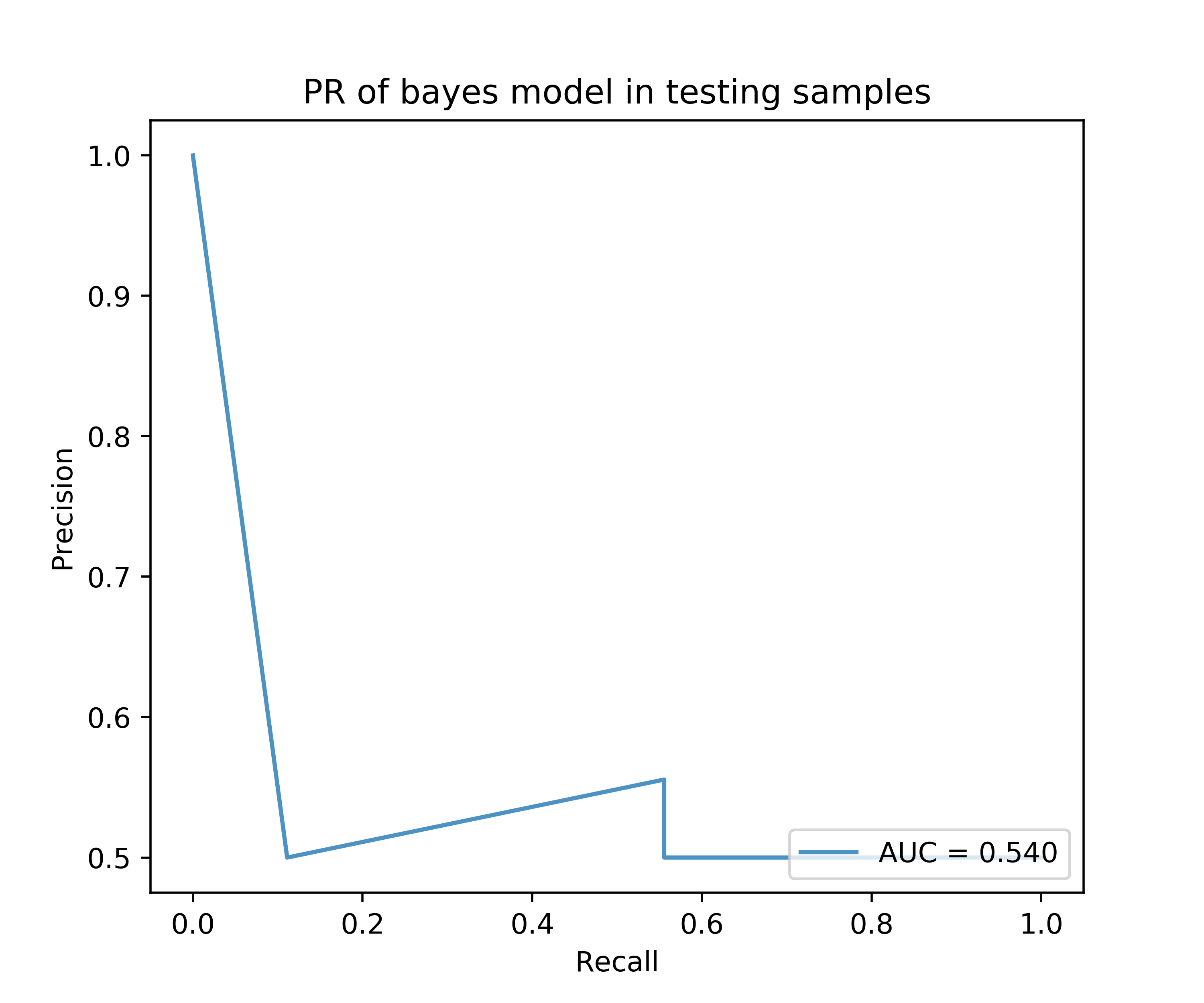

Supplement: Supplementary file 1 — Additional file 1. Supplementary Material. [file 12880_2022_859_MOESM1_ESM.docx]
